# Unexpected Rearrangement of Unsaturated Lactones to Give Tricyclic Furopyrrolidinones

**DOI:** 10.1021/acs.joc.6c01880

**Published:** 2026-09-29

**Authors:** Ebuka L. Onyeyilim, David B. C. Martin

**Affiliations:** Department of Chemistry, 4083 University of Iowa, Iowa City, Iowa 52242, United States

## Abstract

An unexpected cyanide-mediated rearrangement of conjugated lactones is described, providing access to novel tricyclic furopyrrolidinones. This reaction occurs in a stoichiometry- and substrate-dependent manner, induced by multiple additions of cyanide to α,β,γ,δ-unsaturated lactones bearing suitable aryl substituents, providing products with adjacent ether, nitrile, and lactam functionalities. Single-crystal X-ray diffraction (SCXRD) studies of two products support the stereochemical assignments, providing evidence of a dynamic equilibrium process.

## Graphical abstract

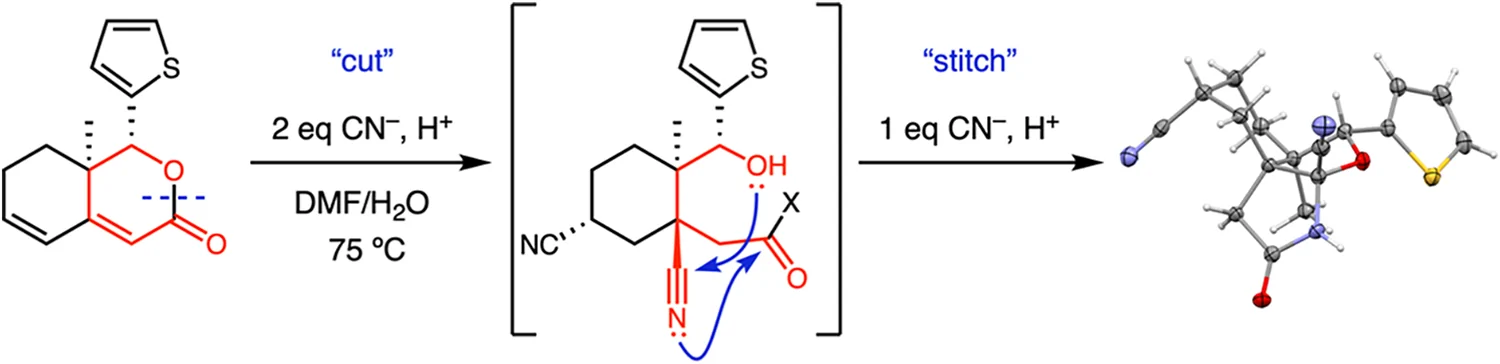

Natural products are a massive reservoir of chemical and biochemical knowledge, providing a guide to what structures are possible and how they can be made.^1,2^ The study of natural products has been a limitless source of inspiration for organic chemists, with a significant portion of what we know about structure and reactivity historically stemming from these studies.^3^ Certain substructures appear repeatedly in diverse product classes, while others are rare and may arise from the intramolecular reactions of functional groups in close proximity.^4^ One rare substructure is the fused bicyclic hexahydro-5*H*-furo­[2,3-*b*]­pyrrol-5-one (or furopyrrolidinone **1**, Figure 1), which contains an N,O-acetal. Only 13 examples of this structure have been reported in natural products, typically as part of larger polycyclic scaffolds (e.g., cytochalasans and colibactins).^5–9^ This structure is far less common than the corresponding furolactone **2** (e.g., ginkgolides, norrisolide, etc.)^10^ and has seen few synthetic studies.

**Figure 1:**
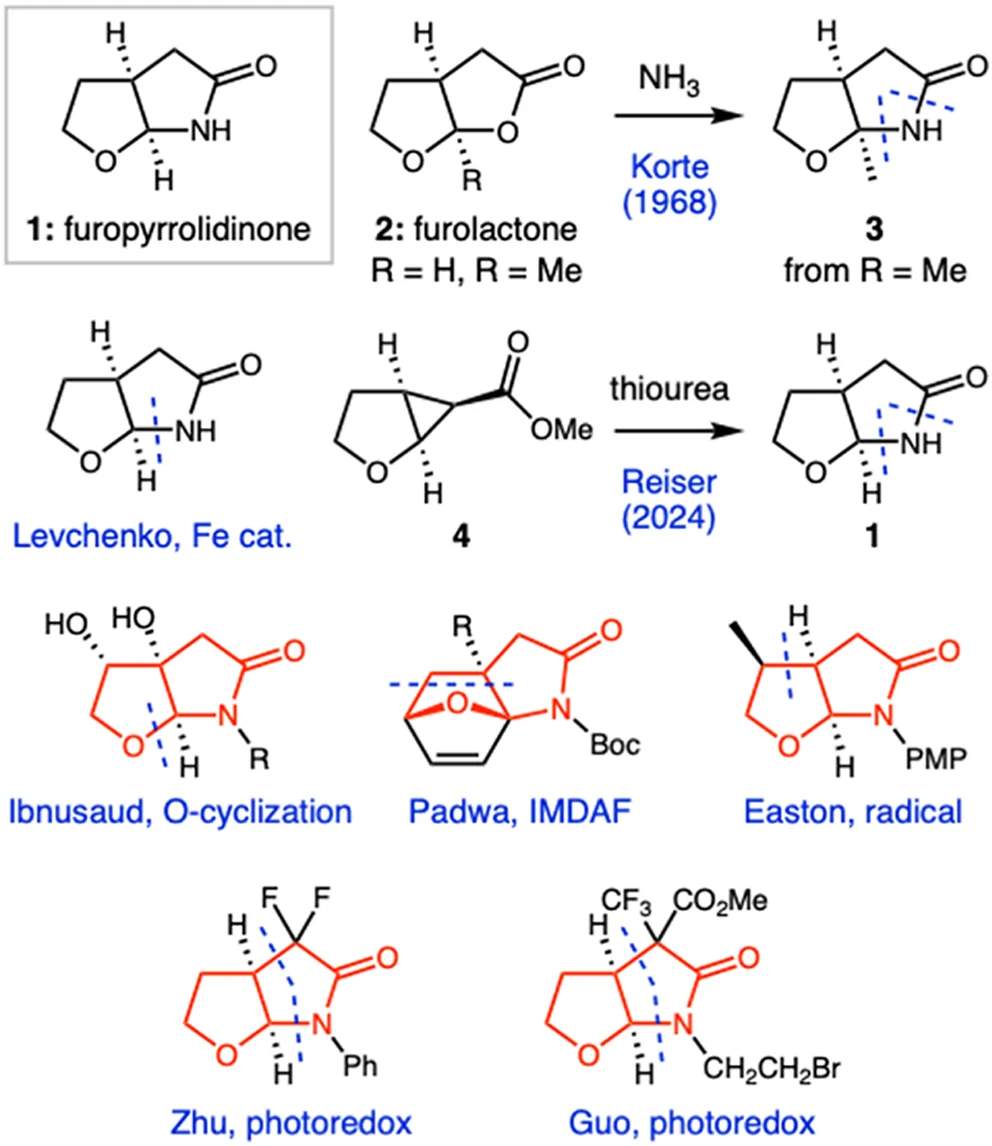
Structures of furopyrrolidinones and furolactones, and notable synthetic approaches to furopyrrolidinones.

The simple furopyrrolidinone **3** was synthesized by Korte in 1968 via treatment of the furolactone **2** with ammonia (Figure 1),^11^ which resembles a reaction reported by Barton in 1961 on a more complex clerodin-derived skeleton.^12^ A similar modification was reported by Ferland to convert a cardenolide lactone to a fused furopyrrolidinone.^13^ Notably, parent structure **1** has only been synthesized recently by Reiser and Levchenko using modern approaches.^14,15^ In particular, Reiser reported many examples using a donor–acceptor cyclopropane (e.g., **4**) opening with thiourea as an N-nucleophile. Other examples have included cyclizations of alcohols onto an N-acyl iminium ions to form the N,O-acetal, in particular by Ibnusaud.^16,17^ Approaches to more elaborate furopyrrolidinones that involve C–C bond formation include intramolecular Diels–Alder reactions of amidofurans (IMDAF),^18–20^ radical cyclizations,^21^ photoredox-catalyzed (3 + 2)-type radical approaches,^22,23^ and others.^24^ Herein, we report the unexpected synthesis of the furopyrrolidinone framework by a cascade reaction initiated by cyanide addition to unsaturated bicyclic lactones.

Our group is interested in limonoid natural products, specifically in investigating their neuroprotective properties and mechanism of action.^25,26^ Within these investigations, we explored the addition of cyanide to the α,β,γ,δ-unsaturated lactone of pyroangolensolide (**11**) and related molecules to introduce new functionality based on reports of cyanide conjugate addition reactions to α,β,γ,δ-unsaturated ketones.^27–30^ As shown in Scheme 1, we use a four-step sequence reported by Fernández-Mateos to access pyroangolensolide analogs, starting with a highly diastereoselective aldol of 6-methyl-2-cyclohexenone **6**.^31^ The aldol product (e.g., **7a**) is then acetylated, subjected to intramolecular aldol with LDA, then dehydrated using SOCl_2_ to give the unsaturated lactone **10a**. In one instance of cyanide addition with 2-thiophene substrate **10a**, we made an interesting observation. With only 2.3 equiv of KCN and NH_4_Cl at 75 °C in aqueous DMF, we observed cyanide addition at the δ-position to give nitrile **12a**.^30^ A crystal structure of **12a** confirmed the location and relative configuration of the new CN group (*trans* to the C18 methyl group), resulting from axial attack on the δ-carbon (C9, see intact limonoid numbering). However, when more forcing conditions were used (6 equiv KCN and NH_4_Cl, 5d), we observed a new product with unexpected spectroscopic features. The ^1^H NMR showed no alkene peaks, and peaks were observed consistent with a new aliphatic methylene (geminal J coupling) and a heteroatom-bound hydrogen (broad singlet). The ^13^C NMR showed the presence of two nitriles, two new fully substituted carbons at 54.6 and 91.4 ppm, and a distinct carbonyl carbon at 172.9 ppm. The HRMS pointed to a molecular formula of C_17_H_17_N_3_O_2_S, indicating that three molecules of HCN had been incorporated. 2D NMR analysis left some doubts about the rearranged skeleton, but we were able to grow crystals suitable for single-crystal X-ray diffraction. This finally revealed the tricyclic lactam structure **13a** with a furopyrrolidinone fused to the cyclohexane, sharing two vicinal quaternary centers (C13 and C14). The N,O-acetal carbon bearing the nitrile accounts for the other fully substituted carbon (91.4 ppm). Surprisingly, the stereochemistry of the nitrile-bearing C9 is observed to be opposite to that of the simple nitrile addition product **12a** (*cis* to the methyl group), presenting more questions about the pathway leading to rearranged structure **13a**.

**Scheme 1:**
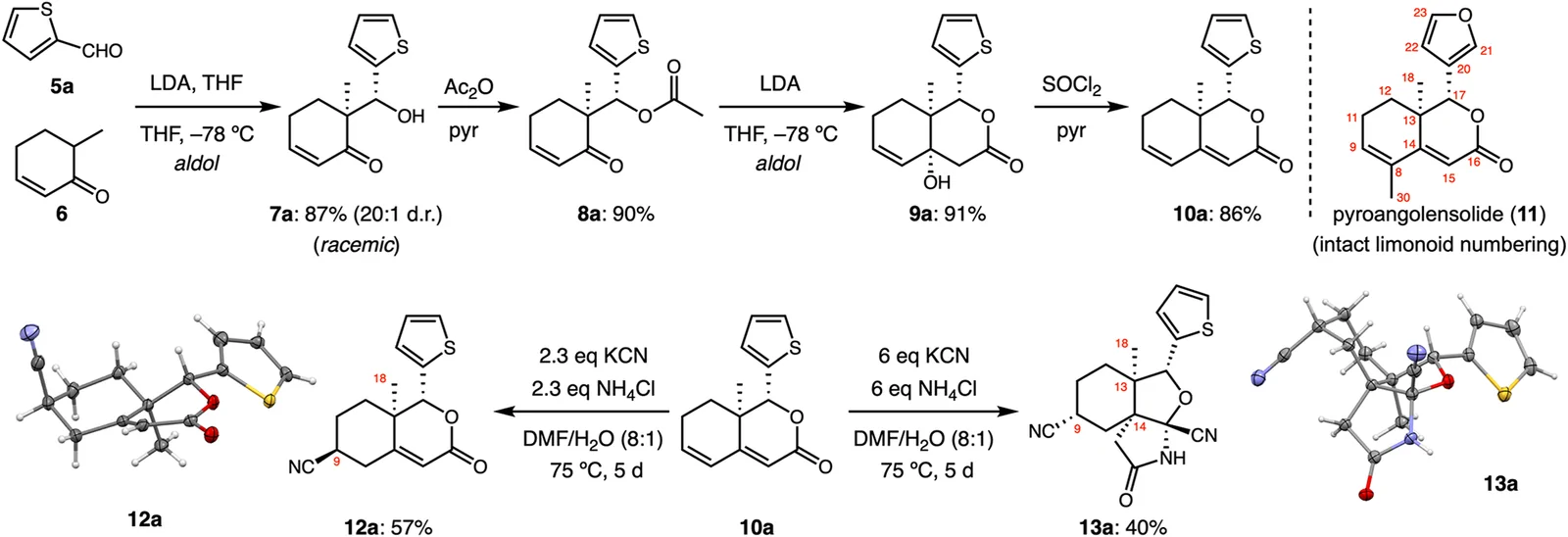
Synthesis of α,β,γ,δ-Unsaturated Lactone **10a**, Initial Studies of Cyanide Addition Reactions, and X-ray Crystal Structures of Products **12a** and **13a**

Satisfied with our structural assignment, we set out to investigate this cyanide-promoted reaction and determine the scope (Scheme 2). Lactone substrates were synthesized using the same four-step synthetic sequence with different aldehydes **5b**–**h** (see Supporting Information for details). Under the same conditions (6 equiv KCN and NH_4_Cl, 75 °C, 5d), 5-bromo-2-thiophene substrate **10b** underwent the same rearrangement to give rearranged product **13b** in 18% isolated yield. A *p*-fluorophenyl substrate **10c** gave rearrangement product **13c** (23%) as well as some single cyanide addition product **12c** (9%). We found that only single cyanide addition products were observed for 2-furyl, phenyl, and cyclohexyl substrates (58% **12d**, 58% **12e**, and 30% **12f**, respectively). Interestingly, 3-thiophene substrate **14g** and pyroangolensolide (**11**)^31^ showed no reactivity under these conditions, with only starting material recovered. Pushing substrate **14g** harder at 90 °C resulted in some decomposition but no cyanide addition products. These results show a dramatic effect of the lactone substituent on the outcome of this reaction.

**Scheme 2:**
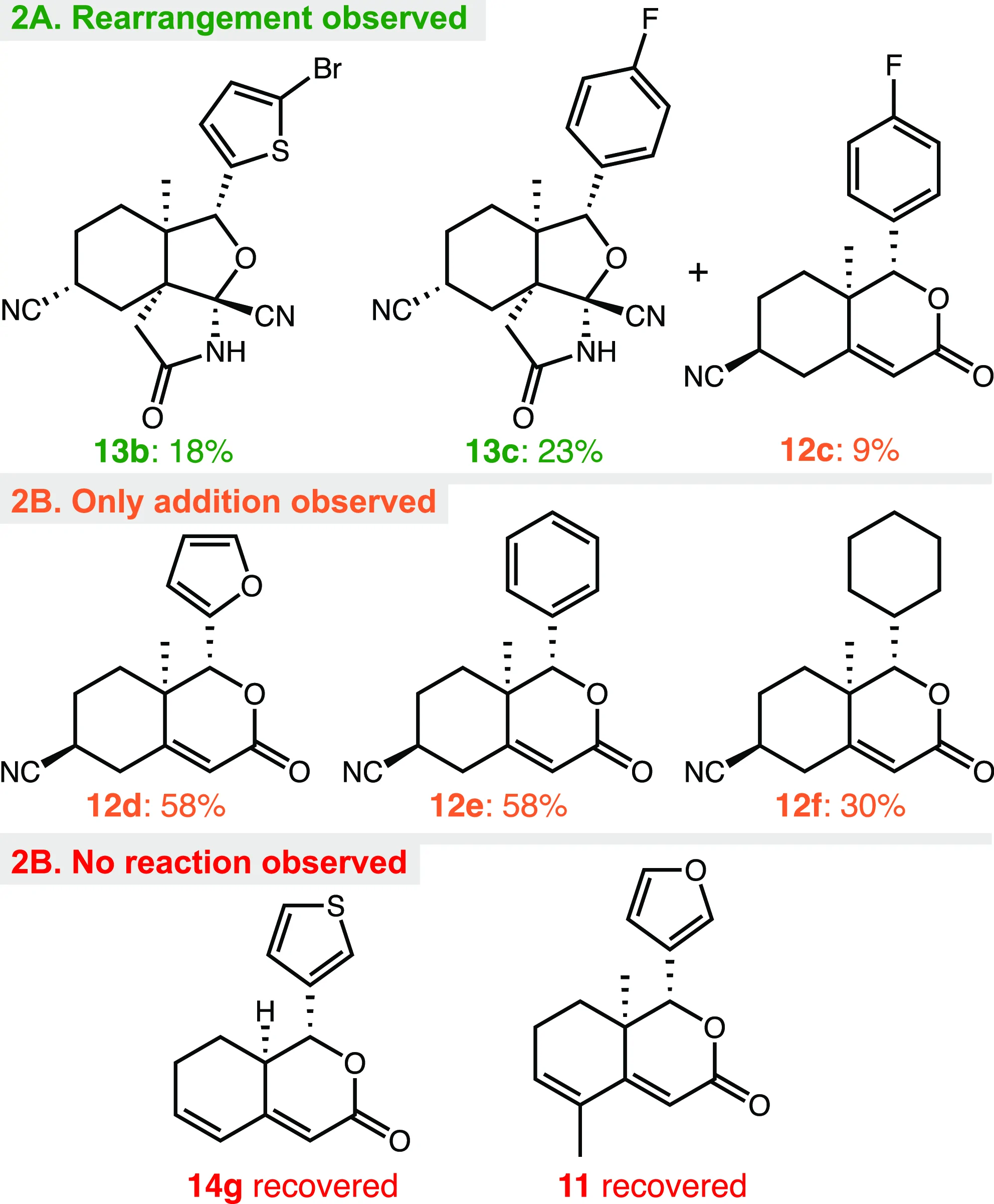
Cyanide Addition/Rearrangement Results for Various α,β,γ,δ-Unsaturated Lactones under Standard Conditions

To ascertain what other features are necessary for this rearrangement, mechanistic experiments were performed. A substrate lacking the γ,δ-unsaturation was synthesized by reduction with hydrogen and Pd/C. As shown in Scheme 3, when substrate **15h** was subjected to the same reaction conditions, the rearranged product **16h** was observed in 33% yield, indicating that the γ,δ-unsaturation is not necessary. We also isolated the β-cyanide conjugate addition product **17h** (28%), which we did not observe in other reactions. We hypothesize this is an intermediate in the rearrangement reaction to the furopyrrolidinone (see proposal below). We also resubjected nitrile addition products **12e** and **12a** to the reaction conditions. With phenyl substrate **12e**, we primarily recovered the starting material. Some decomposition was observed, but no clear evidence of rearrangement was detected. When the nitrile addition product **12a** was resubjected to the reaction conditions, we observed the formation of the previously isolated furopyrrolidinone **13a** in a comparable yield (22%), which would require inversion of the C9 stereochemistry. We also isolated smaller amounts of furopyrrolidinone **18a** with retained stereochemistry at C9 (CN *trans* to the methyl group, both equatorial), the only time this *trans* diastereomer was found in our studies. This outcome suggests that C9 epimers **13a** and **18a** are independently formed rather than resulting from equilibration of the C9 center after formation of the rearranged core. In contrast, these observations suggest that a dynamic equilibrium exists between C9 epimers **12a** and **19a** prior to a second cyanide addition leading ultimately to the rearranged core (either **13a** or **18a**).

**Scheme 3:**
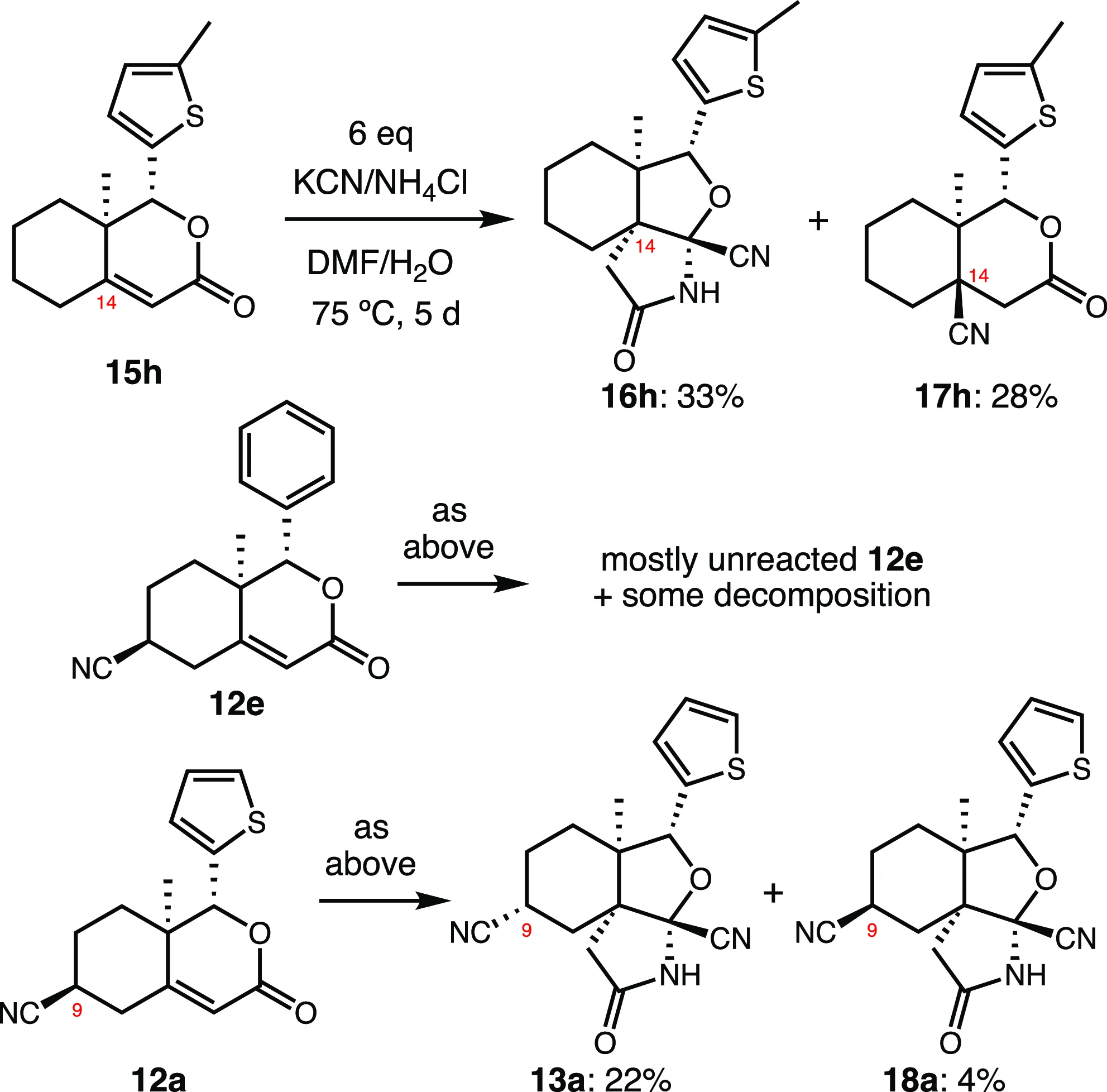
Rearrangement Studies of Monounsaturated Lactones

The proposed mechanism for this rearrangement is presented in Scheme 4A. α,β,γ,δ-Unsaturated lactone **10a** undergoes reversible cyanide addition to give either **12a** or **19a**, with reaction on the top face being favored kinetically due to axial attack, avoiding a twist-boat transition state.^32,33^ Subsequent cyanide addition to the β-carbon of **12a** is disfavored due to 1,3-diaxial interactions (see Scheme 4B), while conjugate addition on the top face of equatorial nitrile **19a** is unhindered, giving *trans*-dinitrile **20a** (not observed). The intermediacy of **20a** is supported by the isolation of β-cyanolactone **17h** (Scheme 3). We note that double cyanide addition to dienones to give β,δ-dicyano ketones has been reported, in some cases leading to further cyclizations (e.g., lactams);^27,28^ however, this is the first evidence of a β,δ-dicyano lactone to our knowledge. From dinitrile **20a**, attack by a third molecule of cyanide and ring-opening gives an alkoxide **22a** (or corresponding alcohol), which can cyclize onto the β-nitrile to form the 5-membered oxacycle in **23a**.^34,35^ Cyclization of nitrogen gives the 5-membered azacycle **25a**, and addition of cyanide to the N-acylimidate on the top face leads to the observed *cis*-fused furopyrrolidinone product **13a**. The observation of *trans*-nitrile **18a** when starting from *trans*-nitrile **12a** suggests that an analogous pathway can proceed by conjugate addition on the top face of **12a** when starting with this compound, but equilibration to the more reactive *cis*-nitrile **19a** is the dominant process. Side reactions of the intermediates in Scheme 4A with NH_3_, H_2_O, etc. likely leads to the low yields observed under the thermal conditions used (75 °C).

**Scheme 4:**
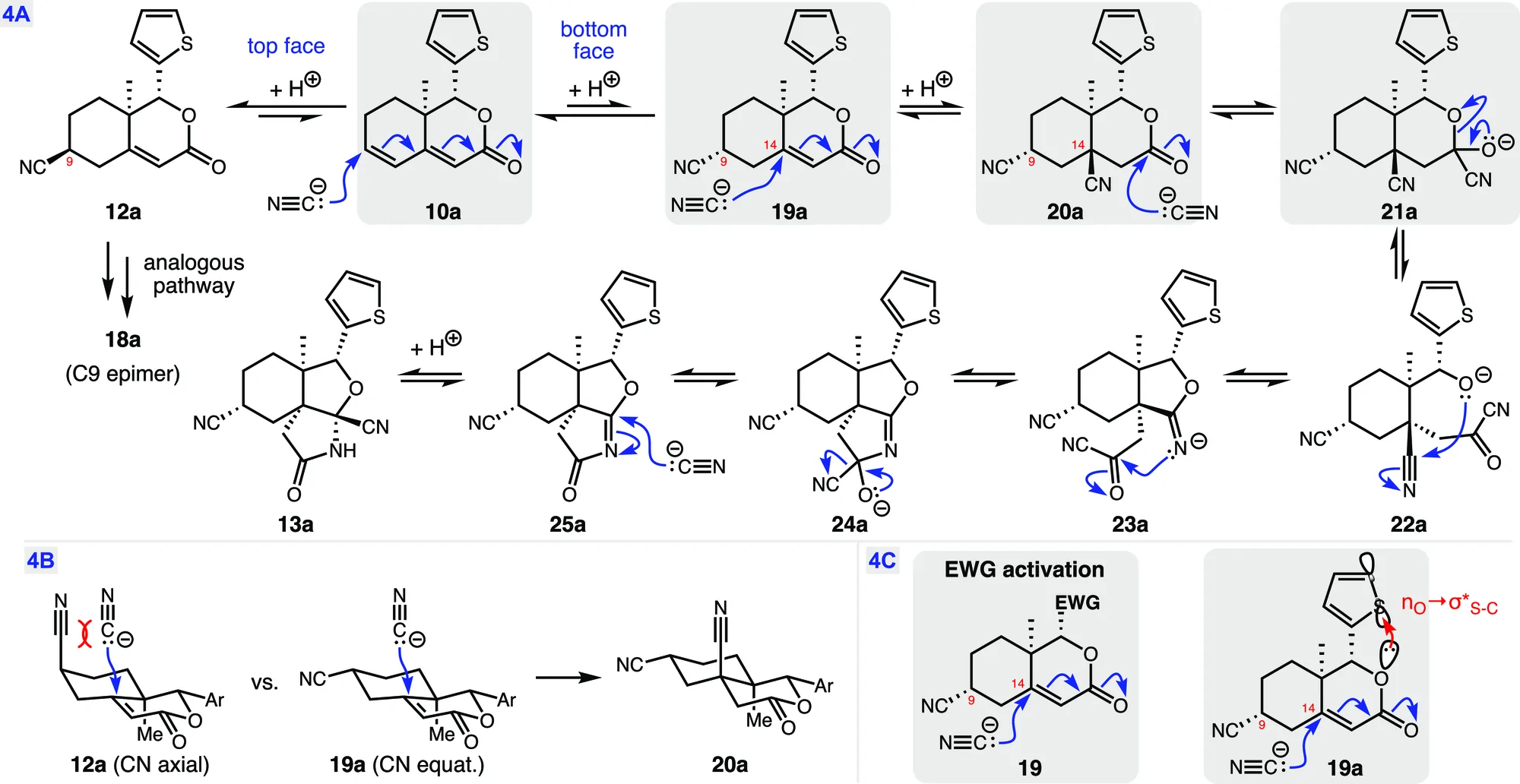
Proposed Mechanism of the Rearrangement, Conformational Analysis of Intermediates and Possible Effect of Chalcogen Bonding on Substrate Reactivity

The explanation for different reaction outcomes based on the aryl substituent is not fully clear at this point. The difference between the phenyl substrate **10e** forming addition product **12e** and the *p*-fluorophenyl substrate **10c** forming rearranged product **13c** suggests that the electron-withdrawing effect of the aryl group could play a key role. The steps highlighted in Scheme 4A with gray boxes should benefit from electron-withdrawing groups, favoring addition of the cyanide nucleophile at the δ-, β-, and carbonyl carbons sequentially. In this context, the successful rearrangement of the 2-thiophene substrates **10a**, **10b**, and **15h** and the failure of the 3-thiophene substrate **14g** and furan substrates **10d** and **11** suggest an electron-withdrawing effect of the 2-thiophene group. The 2-thiophene group is uniquely positioned to engage in chalcogen bonding with the nearby ester oxygen (see Scheme 4C), and chalcogen bonding has been invoked to explain reactivity enhancements in organocatalytic reactions (LUMO-lowering), including with S-heterocycle catalysts.^36–39^ Examination of the crystal structures in Scheme 1 shows the *syn*-coplanar arrangement of sulfur and oxygen in both **12a** and **13a**, with S–O distances of 2.77 Å and 3.00 Å, respectively. Both measured distances are shorter than the sum of van der Waals radii (3.32 Å), which is consistent with a stabilizing attractive interaction.^40^ We speculate that the n_O_ → σ*_S–C_ interaction shown in Scheme 4C could account for enhanced reactivity of the 2-thiophene substrates; however, further studies are required to test this hypothesis.

In conclusion, we have discovered an unexpected rearrangement of unsaturated lactones to yield aryl-substituted tricyclic furopyrrolidinones. This rearrangement is induced by the conjugate addition of cyanide and occurs in a substrate-dependent manner, where 2-thiophene and *p*-fluorophenyl substrates participate, and other aryl and alkyl substrates do not. We propose that the electron-withdrawing effect of the *p*-fluorophenyl group inductively and 2-thiophene group via chalcogen bonding promotes the addition of successive cyanide nucleophiles, and future studies will aim to test this hypothesis.

## Supplementary Material



## Data Availability

The data underlying this study are available in the published article and its Supporting Information.

## Notes

Experimental procedures, characterization data, and ^1^H and ^13^C NMR spectra of all new compounds (PDF)
