# Supplementary material for: Unexpected Rearrangement of Unsaturated Lactones to Give Tricyclic Furopyrrolidinones
Source: J Org Chem. 2026 Sep 29;91(41):13806–11. doi: 10.1021/acs.joc.6c01880 (PMC13682204; doi:10.1021/acs.joc.6c01880)
Supplement: Supplementary file 1 [file jo6c01880_si_001.pdf]

Supporting Information

Unexpected Rearrangement of Unsaturated Lactones to give Tricycylic Furopyrrolidinones

Ebuka L. Onyeyilim and David B.C. Martin*
Corresponding Author: david-martin@uiowa.edu

Department of Chemistry, University of Iowa, Iowa City, Iowa 52242, United States.

Contents

General Methods - - - - - - - - S2

Safety Statement - - - - - - - - S4

Experimental Procedures - - - - - - - S4

General Synthetic Procedures- - - - - - - S5

1. General Procedure A: Aldol reaction - - - - - S5
2. General Procedure B: Acetylation reaction- - - - S5
3. General Procedure C: Intramolecular aldol reaction- - S6
4. General Procedure D: Dehydration reaction- - - - S6
5. General Procedure E: CN conjugate addition reaction- - S7
6. General Procedure F: CN polyaddition reaction- - - S7

Characterization Data of Products - - - - - S8-31

NMR Spectra - - - - - - - - S32-76

References - - - - - - - - - S77

General Methods:

All reactions were carried out in a round-bottom flask charged with a magnetic stir bar under nitrogen. All aldehydes were freshly distilled and stored under nitrogen. All intermediates were prepared according to known literature procedures unless otherwise indicated. Reactions were monitored by thin-layer chromatography (TLC) and carried out on 0.25 mm coated commercial silica gel plates (Analtech TLC Uniplates, F254 precoated glass plates) using UV light as the visualizing agent. Unless otherwise indicated, silica gel chromatography was performed using flash chromatography on P60 silica. ^{1}H and ^{13}C NMR spectra were recorded on a Bruker Neo 400.30 MHz, DRX 500.30 MHz and 600.21 MHz spectrometer and were internally referenced to

residual protio solvent signal (note: $CDCl_3$ referenced at δ 7.26 ppm for ^{1}H NMR and δ 77.16 ppm for ^{13}C NMR, respectively). Data for 1H NMR are reported as follows: chemical shift (δ ppm), multiplicity (s = singlet, d = doublet, t = triplet, q = quartet, m = multiplet, app=apparent), coupling constant (Hz), and integration. Data for $^{13}C\{^{1}H\}$ NMR are reported in terms of chemical shift; no special nomenclature is used for equivalent carbons. High-resolution mass spectrometry (HRMS) data were recorded on a Thermo Q-Exactive instrument (hybrid quadrupole Orbitrap) using direct injection of samples in dichloromethane diluted into acetonitrile/water (1:1) into the electrospray source (ESI) with positive ionization or negative ionization (as noted) or on an Agilent 7250 GCMS Q-TOF instrument with electron ionization (EI) using direct injection of samples in dichloromethane, listed as GC-HRMS.

The crystal data were collected on a Bruker D8 VENTURE DUO diffractometer equipped with a IμS 3.0 microfocus source operated at 75 W (50kV, 1.5 mA) to generate Mo Kα radiation (λ = 0.71073 Å) and a PHOTON III detector. Crystals were transferred from the vial and placed on a glass slide in type NVH immersion oil by Cargille. A Zeiss Stemi 305 microscope was used to identify a suitable specimen for X-ray diffraction from a representative sample of the material. The crystal and a small amount of the oil were collected on a MiTeGen 100-micron MicroLoop and transferred to the instrument, where it was placed under a cold nitrogen stream (Oxford 800 series) at 100K. The sample was optically centered with the aid of a video camera to ensure that no translations were observed as the crystal was rotated through all positions. A unit cell collection was then carried out. After it was determined that the unit cell was not present in the CCDC database, a data collection strategy was calculated by *APEX6*.[1] The crystal was measured for size, morphology, and color. These values are reported in the accompanying mar25_12_tables files. After data collection, the unit cell was re-determined using a subset of the full data collection. Intensity data were corrected for Lorentz, polarization, and background effects using the *APEX6*.[1] A numerical absorption correction was applied based on a Gaussian integration over a multifaceted crystal and followed by a semi-empirical correction for adsorption applied using *SADABS*[1]. The program *SHELXT*[2] was used for the initial structure solution and *SHELXL*[3] was used for refinement of the structure. Both programs were utilized within the OLEX2 software.[4] In this structure, one of the thiophene moieties was positionally disordered over 2 sites with refined occupancies of 0.87 and 0.13 for the major and minor sites, respectively. To help maintain reasonable bond lengths and ADP values, SADI, RIGU, and SIMU restraints were applied to the disordered sites. Hydrogen

atoms bound to carbon and oxygen atoms were located in the difference Fourier map and were geometrically constrained using the appropriate AFIX commands. Hydrogen atoms bound to N1 and N4 were allowed to freely refine their positions.

Safety Statement

Caution: n-BuLi is a pyrophoric reagent and should be handled under an inert atmosphere (nitrogen) using air-free Schlenk techniques. Diisopropylamine (DIPA) is an irritant amine that may cause eye and skin irritation upon exposure. Lithium diisopropylamide (LDA) generated from n-BuLi and DIPA should also be handled under an inert atmosphere (nitrogen) using air-free Schlenk techniques. Methyl iodide (CH_3I) is a known volatile alkylating agent, toxic and a suspected carcinogen. Potassium cyanide (KCN) is highly toxic and readily releases poisonous HCN gas when it comes in contact with acids. Thionyl chloride ($SOCl_2$) is corrosive, moisture-sensitive, and releases toxic SO_2 and HCl gases upon decomposition or hydrolysis. All experiments were conducted under an inert atmosphere and in a properly functioning fume hood while wearing appropriate personal protective equipment (PPE).

Experimental Procedures

Procedure for Methylation Reaction to afford Intermediate **6**

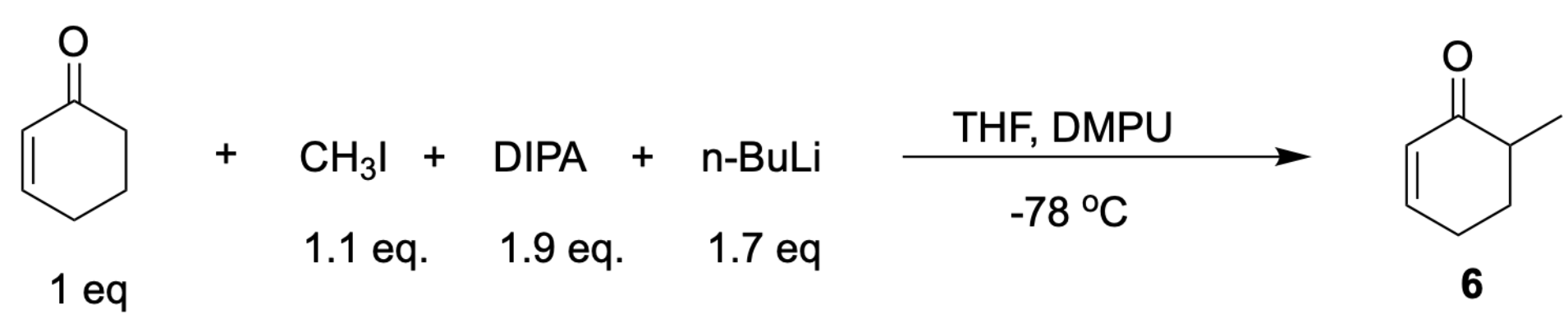

Product **6** was synthesized according to a previously reported procedure.[5] A round-bottom flask containing a magnetic stir bar is charged with diisopropylamine (DIPA) (5.6 mL, 39.5mmol, 1.9 eq) and anhydrous tetrahydrofuran (THF) (25 mL). The solution was cooled down to 0 °C, then n-BuLi (2.5 M in hexane, 14 mL, 35.2 mmol) was added dropwise. The resulting solution was allowed to stir at 0 °C for 30 min. Then, the reaction mixture was cooled down to -78 °C, and a solution of 2-cyclohexen-1-one (2 mL, 20.8 mmol, 1 eq) in THF was added dropwise. After 30 minutes, iodomethane (3.24 mL, 51.3 mmol) was added dropwise at -78 °C and stirred for 30 minutes. N,N-dimethylpropyleneurea (DMPU) (15 mL) was then added, and the reaction mixture was allowed to gradually warm to rt. After 12 h, the reaction mixture was cooled to 0 °C saturated NH_4Cl solution (30 mL) was slowly added. After warming to rt, the reaction mixture was extracted

with diethyl ether (2 x 15 ml). The combined organic layers were washed with brine, dried with Na_2SO_4, and concentrated under reduced pressure (Note: due to the volatility of the product, high vacuum was avoided). The resulting residue was purified by column chromatography using silica gel and hexanes/ethyl ether (90:10) as the eluent to afford 6-methyl-2-cyclohexen-1-one as a light-yellow liquid (1.04 g, 51%). NMR data are in accordance with literature values: ^{1}H NMR (600.21 MHz, $CDCl_3$): δ 6.88-6.85 (m, 1H), 5.92-5.90 (d, J = 9.2 Hz, 1H), 2.34-2.31 (m, 3H), 2.03-1.98 (m, 1H), 1.70-1.63 (m, 1H), 1.08-1.06 (d, J = 6.9 Hz, 3H). ^{13}C{^{1}H} (150.94 MHz, $CDCl_3$): δ = 202.4, 149.8, 129.3, 41.6, 30.8, 25.5, 15.0.

General Procedures.

General Procedure A: Adol reaction of cyclohexenones with various aldehydes (**5a-h**)

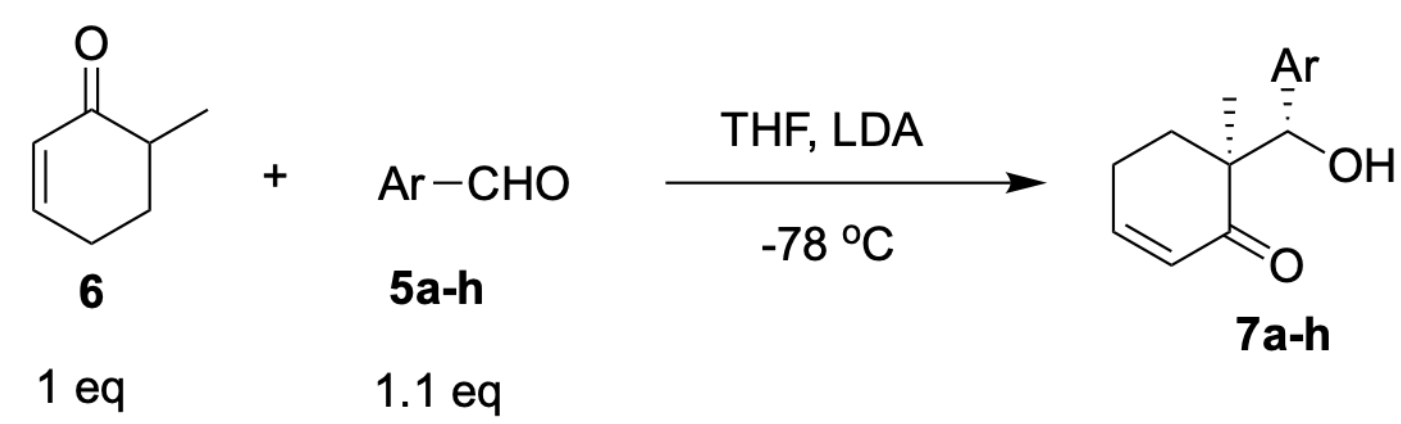

Prepared using a known procedure in the literature.[6] A round-bottom flask containing a magnetic stir bar is charged with DIPA (1.1 eq) and anhydrous THF (2-3 M). The solution was cooled down to 0 °C, then n-BuLi (2.5 M in hexane, 1.1 eq) was added dropwise. The resulting solution was allowed to stir at 0 °C for 30 min. Then, the reaction mixture was cooled down to -78 °C, and a solution of **6** (1 eq, 4.5 mmol) in THF (0.1 M) was added dropwise. After 30 min, freshly distilled aldehydes **5a-h** (1.1 eq) were added, and the solution was stirred at this temperature for 1 h. At reaction completion, NH_4Cl solution was slowly added, and was allowed to warm up to rt before the mixture was extracted with diethyl ether three times. The combined organic layers were washed with brine, dried with Na_2SO_4, and concentrated under reduced pressure. The resulting residue was purified by column chromatography using silica gel and hexanes/ethyl acetate as the eluent to afford the aldol products **7a-h**.

General Procedure B: Acetylation reaction to afford products **8a-h**

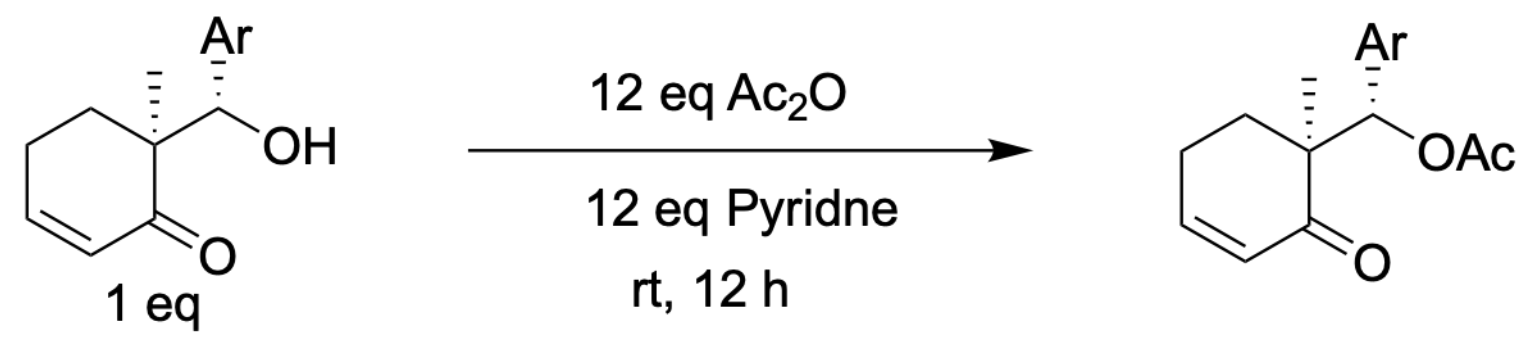

7a-h **8a-h**

The aldol intermediates **(7a-h)** (1 eq) were added to a round-bottom flask containing pyridine (12 eq), a magnetic stir bar, and stirred for 5 min at rt. Acetic anhydride (12 eq) was added, and the solution was allowed to stir overnight at rt. At reaction completion, ice was added to the solution and allowed to stir for an additional 15 min before extracting the solution with DCM (3 times), and the layers were separated. After extraction, the combined organic layers were washed with 2M HCl, saturated Na_2CO_3, brine, dried with Na_2SO_4, and concentrated under reduced pressure. The resulting residue was purified by column chromatography using silica gel and hexane/ethyl acetate as the eluent to the acetylated Intermediates **8a-h.**

General Procedure C: Intramolecular aldol reaction to afford products **9a-h**

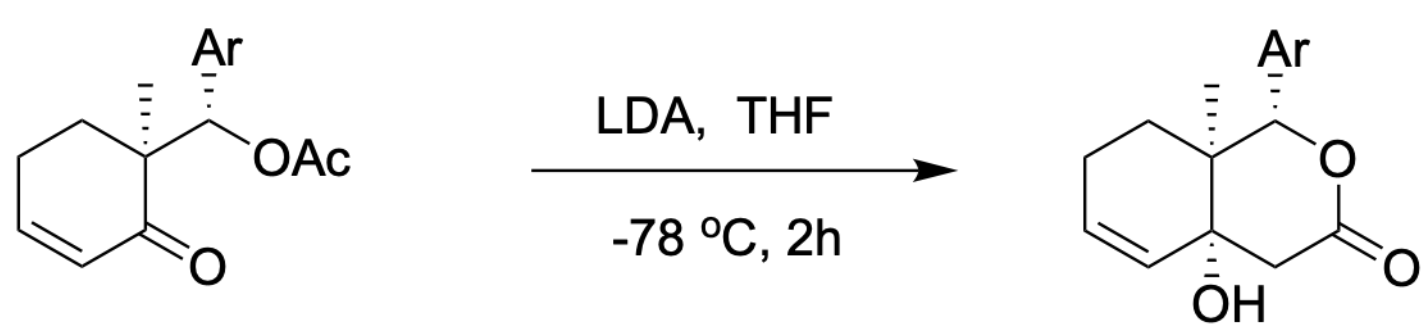

8a-h **9a-h**

Prepared using a known procedure in the literature.[6] A round-bottom flask containing a magnetic stir bar is charged with DIPA (1.1 eq) and anhydrous THF (0.3-0.5 M). The solution was cooled down to 0 °C, then n-BuLi (2.5 M in hexane, 1.1 eq) was added dropwise. The resulting solution was stirred at 0 °C for 30 min. Then, the reaction mixture was cooled down to -78 °C, and a solution of **8a-h** (1 eq) in THF was added dropwise. The solution was stirred at this temperature for 2 h. At reaction completion, saturated NH_4Cl solution was slowly added, and the reaction mixture was extracted with diethyl ether (3 times). The combined organic layers were washed with brine, dried with Na_2SO_4, and concentrated under reduced pressure. The resulting residue was purified by column chromatography using silica gel and hexanes/ethyl acetate as the eluent to afford the aldol products **9a-h.**

General Procedure D: Dehydration reaction to afford products **10a-h**

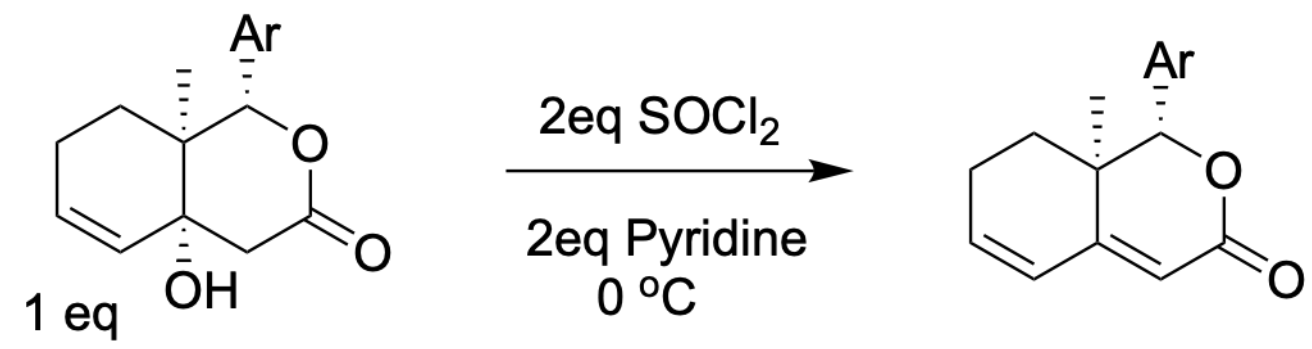

9a-h **10a-h**

Prepared using a known procedure in the literature.[6] A round-bottom flask containing a magnetic stir bar is charged with pyridine (2 eq) and the starting material **9a-h** (1 eq) in THF (0.4-0.7 M). The solution was cooled down to 0 °C, then $SOCl_2$ (2 eq) was added dropwise. The resulting solution was stirred at 0 °C for 20 min. At reaction completion, ice was added to the solution and allowed to stir for an additional 15 min before extracting the solution with DCM (3 times). After extraction, the combined organic layers were washed with 2 M HCl, Na_2CO_3, brine, dried with Na_2SO_4, and concentrated under reduced pressure to obtain the products **10a-h**. The crude products **10a-h** were used in the next step of the synthesis without purification, unless otherwise noted.

General Procedure E: CN conjugate addition reaction to afford product **12a-h**

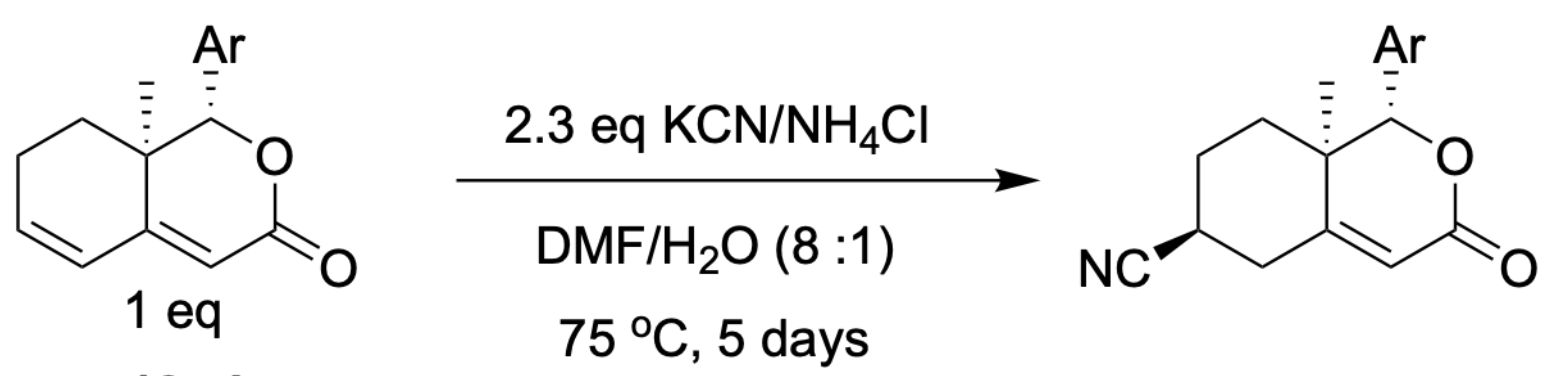

10a-h **12a-h**

Prepared based on related cyanide conjugate addition in the literature.[7] A round-bottom flask containing a magnetic stir bar is charged with KCN (2.3 eq), NH_4Cl (2.3 eq), and the starting material **10a-h** (1 eq) in DMF/H_2O (8 :1 ratio, 0.2 M). The solution was heated to 75 °C in an oil bath under nitrogen for 5 days in a sealed round-bottom flask. At reaction completion, the reaction mixture was cooled to rt, then ice was added to the solution and allowed to stir for an additional 15 min before extracting the solution with DCM (3 times). After extraction, the combined organic layers were washed with H_2O, washed with brine, dried with sodium sulfate, and then concentrated under reduced pressure to obtain the crude products **12a-h**. The resulting crude mixtures was purified by column chromatography using silica gel and hexanes/ethyl acetate as the eluent to afford the pure products **12a-h.**

General Procedure F: CN polyaddition addition reaction to afford product **13a-h**

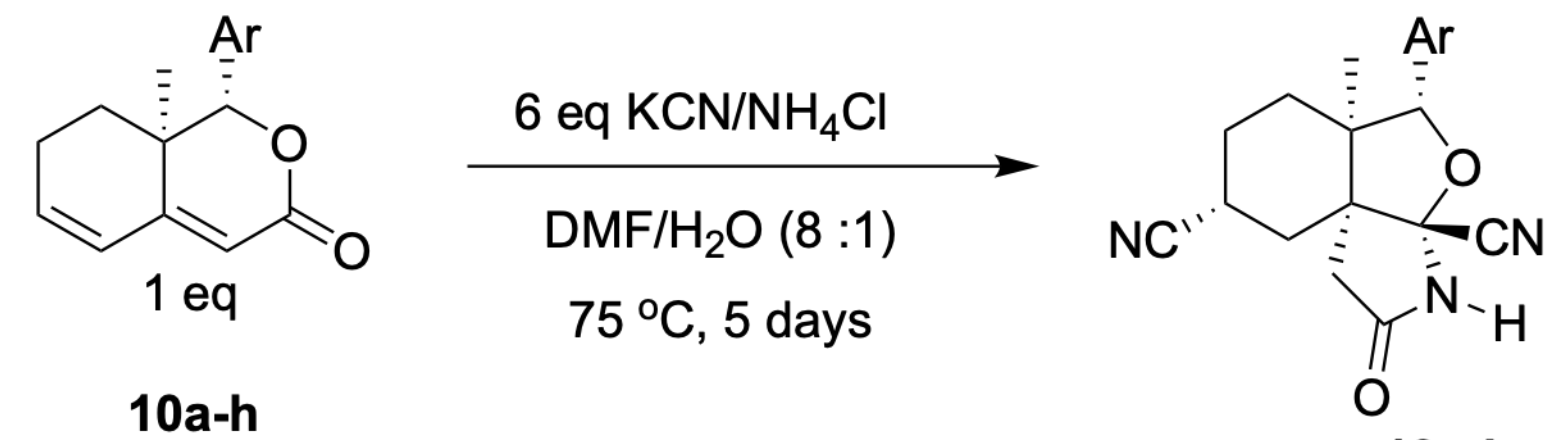

A round-bottom flask containing a magnetic stir bar is charged with KCN (6.0 eq), NH_4Cl (6.0 eq) and the starting material **10a-h** (1 eq) in DMF/H_2O (8 :1 ratio, 0.2-0.6 M). The solution was heated to 75 °C in an oil bath for 5 days. Upon reaction completion, ice was added to the solution, and the mixture was stirred for an additional 15 min before the solution was extracted with DCM (3 times). After extraction, the combined organic layers were washed with H_2O, washed with brine, dried over sodium sulfate, and concentrated under reduced pressure to obtain the crude products **13a-h**. The resulting crude mixtures was purified by column chromatography using silica gel and hexanes/ethyl acetate as the eluent to afford the pure products **13a-h.**

Characterization Data of Products

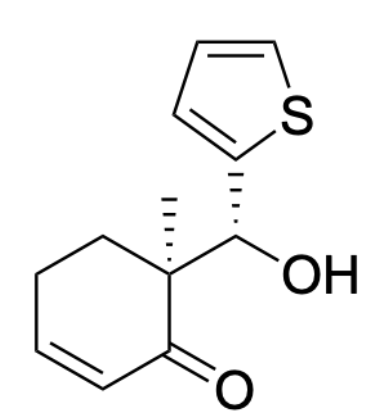

7a

Prepared using general procedure A, using 2-thiophenecarbaldehyde **5a** (0.51 mL, 5.0 mmol) and **6** (0.50 mL, 4.5 mmol) in THF (10 mL), DIPA (1.00 mL, 6.8 mmol) and n-BuLi (1.6 M in hexane, 4.3mL, 6.8 mmol) at -78 °C for 2 h. The crude product was purified by column chromatography using silica gel and hexane/ethyl acetate (90:10) as the eluent to afford aldol intermediate **7a** as a white solid (876 mg, 87%). ^1H NMR (600.21 MHz in $CDCl_3$): δ = 7.25-7.24 (d, J = 5.7 Hz, 1H), 6.99-6.94 (m, 2H), 6.92-6.92 (d, J = 5.5 Hz, 1H), 5.97-5.96 (d, J = 7.9 Hz, 1H), 5.19 (s, 1 H), 4.68 (s, 1H), 2.36-2.34 (m, 2H), 1.80-1.75 (m, 1H), 1.51-1.47 (m, 1H), 1.25 (s, 3H). $^{13}C\{^1H\}$ NMR (150.94 MHz, $CDCl_3$): δ = 206.8, 150.7, 142.3 128.2, 126.0, 125.3, 124.9, 74.7, 47.9, 31.2, 23.0, 14.5. HRMS (ESI-TOF) m/z: $[M+Na]^+$ calcd for $C_{12}H_{14}O_2SNa$ 245.0607; found 245.0604.

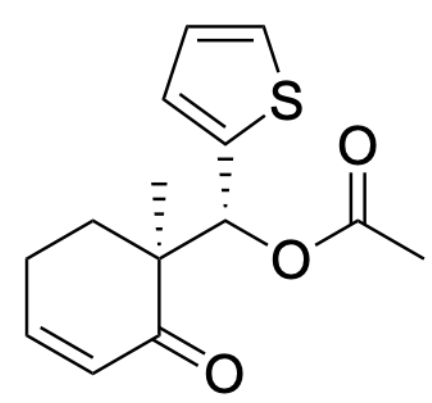

8a

Prepared using general procedure B with **7a** (85.1 mg, 0.38 mmol) in pyridine (0.5 mL) and acetic anhydride (0.5 mL) at rt for 5 h. The crude product was purified by column chromatography using silica gel and hexane/ethyl acetate (90:10) as the eluent to afford Intermediate **8a** as a white solid (91.1 mg, 90 %). ^{1}H NMR (600.21 MHz, $CDCl_3$): δ = 7.20-7.19 (d, J = 5.7 Hz, 1H), 6.96-6.95 (d, J = 5.5 Hz, 1H), 6.93-6.92 (dd, J = 5.7, 1.3 Hz, 1H), 6.88-6.84 (m, 1H), 6.61 (s, 1H), 5.92-5.90 (d, J = 9.6 Hz, 1H), 2.45-2.34 (m, 2H), 2.07 (s, 3H), 1.92-1.82 (m, 2H), 1.21 (s, 3H). $^{13}C\{^{1}H\}$ (150.94 MHz, $CDCl_3$): δ = 200.9, 169.6, 148.8, 140.0, 128.6, 126.6,126.5 125.0, 73.7, 49.4, 29.5, 22.8, 20.9, 18.6. HRMS (ESI-TOF) m/z: $[M+Na]^+$ calcd for $C_{14}H_{16}O_3SNa$ 287.0712; found 287.0710.

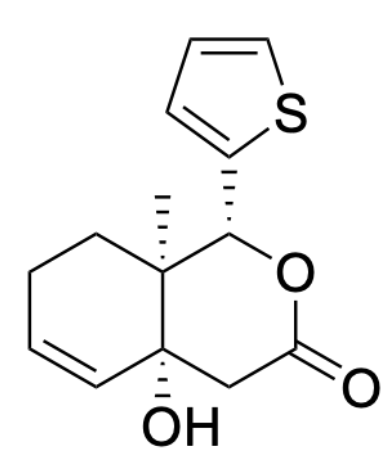

9a

Prepared using general procedure C using intermediate **8a** (694 mg, 2.9 mmol) in THF (20 mL), DIPA (0.4 mL, 4.2 mmol), and n-BuLi (1.6 M in hexane, 2.8 mL, 4.2 mmol) at -78 °C for 3 h. The crude product was purified by column chromatography using silica gel and hexane/ethyl acetate (70:30) as the eluent to afford intermediate **9a** as a white solid (629.3 mg, 91 %). ^{1}H NMR (600.21 MHz, $CDCl_3$): δ 7.33-7.32 (d, J = 6.1 Hz, 1H), 7.07-7.07 (d, J = 3.4 Hz, 1H), 7.03-7.01 (dd, J = 6.1, 3.4 Hz, 1H), 6.03-6.00 (m, 1H), 5.80-5.78 (d, J = 16.8 Hz, 1H), 5.57 (s, 1H), 2.96-2.93 (d, J = 18.0 Hz, 1H), 2.83-2.80 (d, J = 18.0 Hz, 1H), 2.30-2.16 (m, 2H), 1.84-1.79 (m, 1H), 1.50-1.46 (m, 1H), 1.08 (s, 3H). $^{13}C\{^{1}H\}$ NMR (150.94 MHz, $CDCl_3$): δ = 169.5, 138.2, 131.7, 131.4, 126.7, 126.3, 125.6, 80.7, 69.9, 42.6, 40.7, 27.1, 22.6, 15.3. HRMS (ESI-TOF) m/z: $[M+H]^+$ calcd for $C_{14}H_{17}O_3S$ 265.0898; found 265.0891, $[M+Na]^+$ calcd for $C_{14}H_{16}O_3SNa$ 287.0712; found 287.0709.

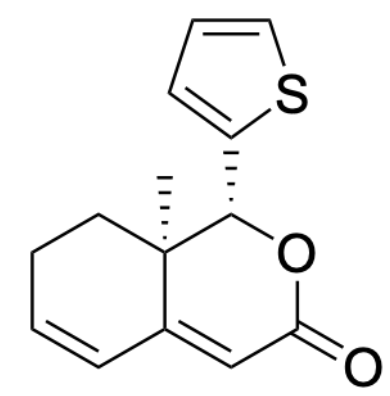

10a

Prepared using general procedure D using intermediate **9a** (75.5 mg, 0.3 mmol) in THF (4 mL), pyridine (0.1 mL, 1.2 mmol), and $SOCl_2$ (0.1 mL, 1.2 mmol) at 0 °C for 20 min. The crude product was washed with 2 M HCl, saturated Na_2CO_3, and brine to afford intermediate **10a** as a brown solid (60 mg, 86 %). The compound was used without purification. ^{1}H NMR (500.30 MHz, $CDCl_3$): δ 7.35-7.33 (d, J = 6.2 Hz, 1H), 7.07-7.06 (d, J = 3.4 Hz 1H), 7.03-7.01 (dd, J = 6.2, 3.4 Hz 1H), 6.35-6.32 (m, 1H), 6.26-6.24 (d, J = 11.8 Hz, 1H), 5.72 (s, 1H), 5.41 (s, 1H), 2.37-2.24 (m, 2H), 1.58-1.49 (m, 2H), 1.12 (s, 3H). ^{13}C{^{1}H} (125.82 MHz, $CDCl_3$): δ = 165.4, 158.8, 139.0, 137.0, 127.1, 126.3, 125.8, 125.3, 83.5, 37.5, 29.9, 22.6, 163. HRMS (EI-QTOF) m/z: $[M]^{+\cdot}$ calcd for $C_{14}H_{14}O_2S$ 246.0709; found 246.0715.

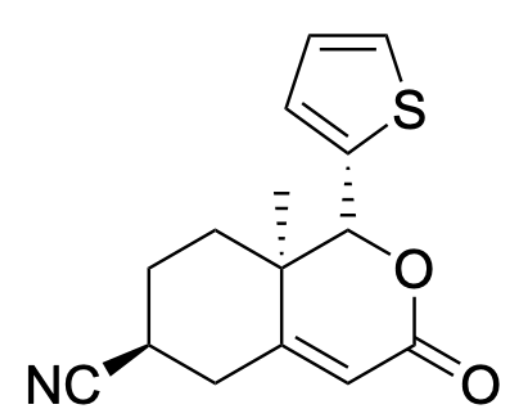

12a

Prepared using general procedure E using intermediate **10a (**38 mg, 0.15 mmol) in DMF/H_2O (4:0.5 ratio, 4.5 mL)**,** KCN (2.3 eq, 23 mg, 0.36 mmol), and NH_4Cl (2.3 eq, 19 mg, 0.36 mmol) at 75 ° C for 5 days. The resulting crude product was purified by column chromatography using silica gel and hexanes/ethyl acetate (60:40) as the eluent to afford **12a** as a light yellow solid (23.8 mg, 57 %). ^{1}H NMR (500.30 MHz, $CDCl_3$): δ 7.36-7.35 (d, J = 5.0 Hz, 1H), 7.11-7.10 (d, J = 3.3 Hz, 1H), 7.04-7.02 (t, J = 4.4 Hz, 1H), 5.96 (s, 1H), 5.49 (s, 1H), 3.20 (m, 1H), 2.74-2.72 (d, J = 15.8 Hz, 1H), 2.66-2.63 (dd, J = 15.7 Hz, 2.1 Hz, 1H), 2.05-2.03 (m, 1H), 1.87-1.77 (m, 2H), 1.62-1.60 (d, J = 13.11 Hz, 1H), 1.15 (s, 3H). ^{13}C{^{1}H} (125.82 MHz, $CDCl_3$): δ = 163.5, 160.3, 136.2, 127.4, 126.4, 126.1, 120.0, 117.9, 83.7, 39.2, 33.0, 31.0, 27.3,23.5, 17.1. HRMS (ESI-TOF) m/z: $[M+H]^+$ calcd for $C_{15}H_{16}NO_2S$ 274.0896; found 274.0897.

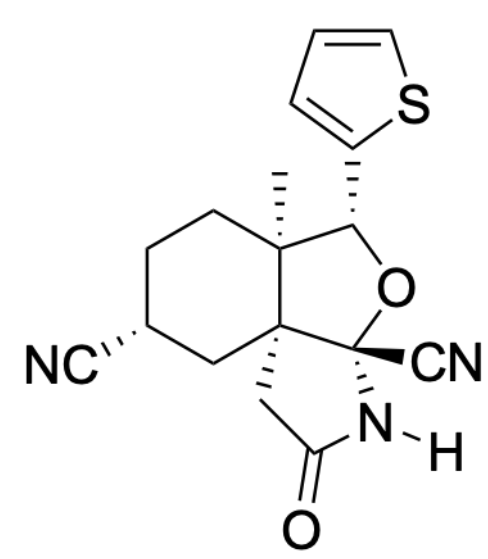

13a

Prepared using general procedure F, using intermediate **10a (**50 mg, 0.2 mmol) in DMF/H_2O (8:1 ratio,9 mL)**,** KCN (6.0 eq, 136 mg, 1.2 mmol), and NH_4Cl (6.0 eq, 74.5 mg, 1.2 mmol) at 75 ^{o}C for 5 days. The resulting crude product was purified by column chromatography using silica gel and hexanes/ethyl acetate (60:40) as the eluent to afford **13a** as a white solid (26.6 mg, 40 %). ^{1}H NMR (500.30 MHz, $CDCl_3$): δ 7.33-7.32 (d, J = 5.1 Hz, 1H), 7.04-7.02 (d, J = 8.7 Hz, 1H), 6.93 (dd, J = 8.6, 5.1 Hz, 1H), 6.50 (s, 1H), 5.55 (s, 1H), 3.33-3.30 (m, 1H), 3.05-3.302 (d, J = 18.0 Hz, 1H), 2.47 – 2.41 (m, 2H), 2.31-2.27 (dd, J = 14.7, 5.2 Hz, 1H), 1.99-1.95 (m, 2H), 1.85-1.82 (m, 2H), 0.97 (s, 3H). ^{13}C{^{1}H} NMR (125.82 MHz, $CDCl_3$): δ = 172.9, 137.6, 127.0, 125.7, 125.3, 120.2, 115.9, 91.4, 85.9, 54.6, 45.8, 40.4, 35.1, 29.0, 24.8, 23.7, 19.8. HRMS (ESI-TOF) m/z: $[M+H]^+$ calcd for $C_{17}H_{18}N_3O_2S$ 328.1114, found 328.1117.

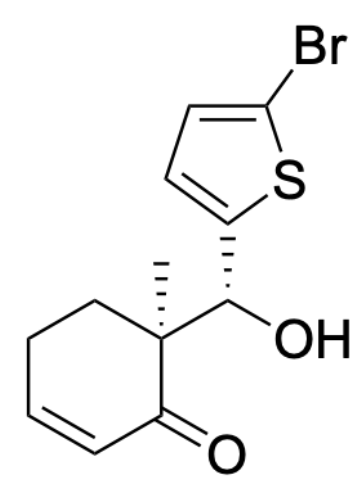

7b

Prepared using general procedure A, using 5-methyl-2-thiophenecarbaldehyde **5b** (0.47 mL, 3.96 mmol) and **6** (0.4 mL g, 3.6 mmol) in THF (8 mL), DIPA (0.6 mL, 3.96 mmol) and n-BuLi (2.5 M in hexane, 1.54 mL, 3.96 mmol) at -78 ^{o}C for 2 h. The crude product was purified by column chromatography using silica gel and hexane/ethyl acetate (90:10) as the eluent to afford aldol intermediate **7b** as a white solid (0.76 g, 70 %). ^{1}H NMR (600.21 MHz, $CDCl_3$) : δ 6.99-6.95 (m, 1H), 6.90-6.88 (d, J = 4.2 Hz, 1H), 6.67-6.65 (d, J = 4.0 Hz, 1H), 5.98-5.96 (d, J = 8.8 Hz, 1H), 5.11 (s, 1H), 4.67 (s, 1H), 2.43-2.40 (m, 2H), 1.84-1.80 (m, 1H), 1.59-1.56 (m, 1H), 1.24 (s, 3H). ^{13}C{^{1}H} NMR (150.94 MHz, $CDCl_3$): δ = 206.41, 150.8, 144.2, 128.8, 128.2, 126.0, 111.8, 75.03,

47.7, 30.9, 23.0, 14.6. HRMS (ESI-TOF) m/z: $[M+Na]^+$ calcd for $C_{12}H_{13}{}^{79}BrO_2SNa$ 322.9712; found 322.9710

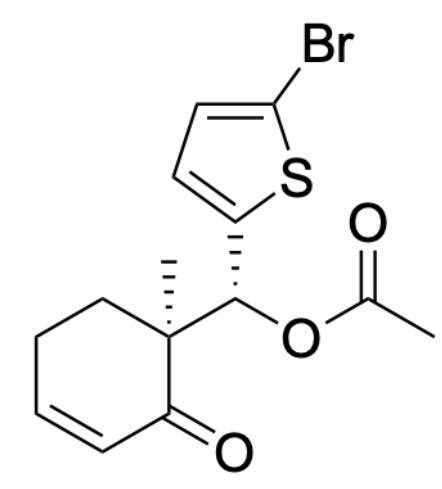

8b

Prepared using general procedure B, **7b** (552 mg, 1.83 mmol) in pyridine (4 mL) and acetic anhydride (4 mL) at rt for 5 h. The crude product was purified by column chromatography using silica gel and hexane/ethyl acetate (90:10) as the eluent to afford intermediate **8b** as a white solid (450 mg, 88 %). ^{1}H NMR (500.30 MHz, $CDCl_3$): δ 6.91-6.87 (m, 2H), 6.72-6.71 (d, J = 4.5 Hz, 1H), 6.52 (s, 1H), 5.95-5.93 (d, J = 9.0 Hz 1H), 2.41 (m, 2H), 2.09 (s, 3H), 1.99-1.94 (m, 1H), 1.89-1.84 (m, 1H), 1.21 (s, 3H). $^{13}C\{^1H\}$ (125.82 MHz, $CDCl_3$): δ = 200.5, 169.5, 148.8, 141.7, 129.3, 128.7, 127.1, 112.0, 74.1, 49.4, 29.1, 22.8, 20.8, 18.8. HRMS (EI-QTOF) m/z: $[M]^{+\cdot}$ calcd for $C_{14}H_{15}{}^{79}BrO_3S$ 341.9920; found 341.9927.

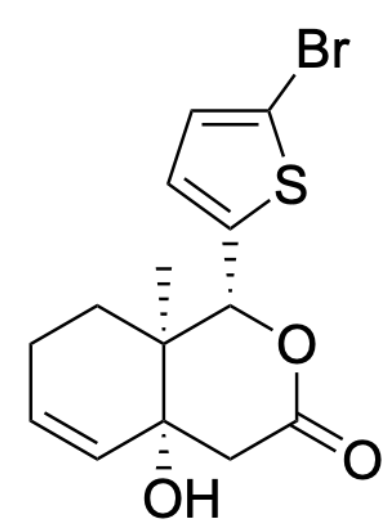

9b

Prepared using general procedure C, using intermediate **8b** (203 mg, 0.6 mmol) in THF (4 mL), DIPA (0.1 mL, 0.6 mmol), and n-BuLi (2.5 M in hexane, 0.25 mL, 0.6 mmol) at -78 °C for 3 h. The crude product was purified by column chromatography using silica gel and hexane/ethyl acetate (7:3) as the eluent to afford intermediate **9b** as a white solid (138.5 mg, 68 %). ^{1}H NMR (500.30 MHz, $CDCl_3$) : 6.97-6.96 (d, J = 3.8 Hz, 1H), 6.82-6.81 (d, J = 4.4 Hz, 1H), 6.01-5.98 (d, J = 17.1 Hz, 1H), 5.78-5.76 (d, J = 14.2 Hz, 1H), 5.45 (s, 1H), 2.94-2.92 (d, J = 18.0 Hz, 1H), 2.81-2.77 (d, J = 18.0 Hz, 1H), 2.21-2.17 (m, 2H), 1.86-1.80 (m, 1H), 1.52-1.47 (m, 1H), 1.07 (s, 3H).

$^{13}C\{^1H\}$ (125.82 MHz, $CDCl_3$): δ = 169.1, 139.9, 129.2, 126.9112.6, 80.4, 69.4, 41.8, 40.0, 27.0, 22.2, 15.0. HRMS (EI-QTOF) m/z: $[M]^{+\cdot}$ calcd for $C_{14}H_{15}{}^{79}BrO_3S$ 341.9920; found 341.9909.

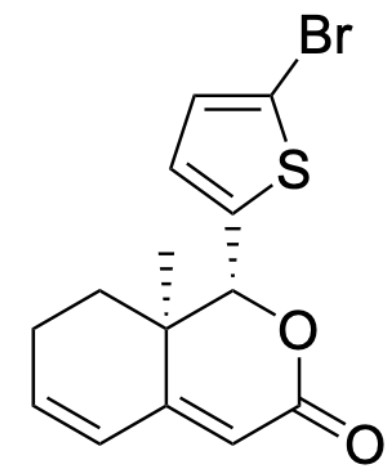

10b

Prepared using general procedure D, using intermediate **9b** (89.1 mg, 0.23 mmol) in THF (3 mL), pyridine (0.07 mL, 0.92 mmol), and $SOCl_2$ (0.07 mL, 0.92 mmol) at 0 °C for 20 min. The crude product was washed with 2M HCl, saturated Na_2CO_3 and brine to afford the intermediate **10b** as a light brown solid (47.3 mg, 56 %) without further purification. ^1H NMR (500.30 MHz, $CDCl_3$) : δ 6.97-6.96 (d, J = 3.8 Hz, 1H), 6.81-6.80 (d, J = 3.7 Hz, 1H), 6.36-6.32 (m, 1H), 6.24-6.22 (d, J = 11.3 Hz, 1H), 5.70 (s, 1H), 5.29 (s, 1H), 2.38-2.29 (m, 2H), 1.59-1.56 (m, 1H), 1.52-1.46 (m, 1H), 1.10 (s, 3H). $^{13}C\{^1H\}$ NMR (125.82 MHz, $CDCl_3$): δ = 165.1, 158.7, 139.2, 138.5, 129.2, 127.4, 125.1, 112.9, 112.3, 83.4, 37.4, 29.9, 22.6, 16.1. HRMS (ESI-TOF) m/z: $[M+H]^+$ calcd for $C_{12}H_{14}{}^{79}BrO_2SNa$ 324.9892; found 324.9897.

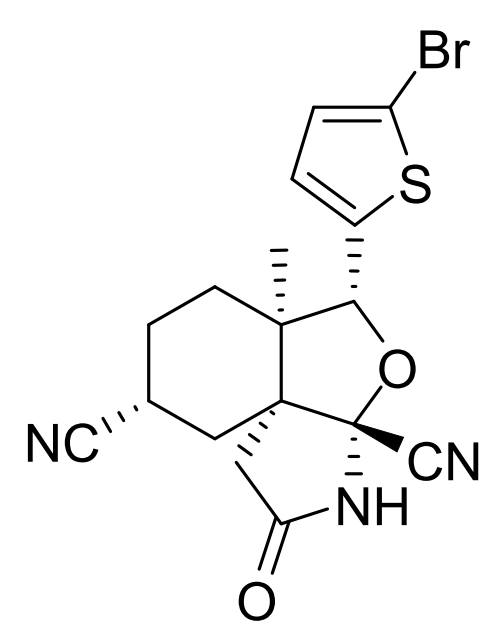

13b

Prepared using general procedure F, using intermediate **10b (**47 mg, 0.15 mmol) in DMF/H_2O (4:0.5 ratio, 4.5 mL)**,** KCN (6.0 eq, 59 mg, 0.9 mmol), and NH_4Cl (6.0 eq, 47 mg, 0.9 mmol) at 75 ° C for 5 days. The resulting crude product was purified by column chromatography using silica gel and hexanes/ethyl acetate (60:40) as the eluent to afford the **13b** as a white solid (10.5 mg, 18 %) , ^1H NMR (500.30 MHz, $CDCl_3$): δ 6.98 (d, J = 4.8 Hz, 1H), 6.68 (d, J = 4.9 Hz, 1H), 6.49 (s, 1H), 5.43 (s, 1H), 3.31-3.29 (m, 1H), 3.05-3.01 (d, J = 18.0 Hz, 1H), 2.45-2.40 (m, 2H), 2.31-2.27

(m, 1H), 2.01-1.90 (m, 2H), 1.83-1.81 (m, 2H), 0.99 (s, 3H). $^{13}C\{^1H\}$ NMR (125.82 MHz, $CDCl_3$): δ =172.6, 139.2, 129.8, 125.6, 120.1, 115.7, 112.8, 91.4, 85.6, 54.5, 45.8, 40.3, 35.1, 28.9, 24.7, 23.719.2. HRMS (ESI-TOF) m/z: $[M+H]^+$ calcd for $C_{17}H_{17}{}^{79}BrN_3O_2S$ 406.0219; found 406.0225.

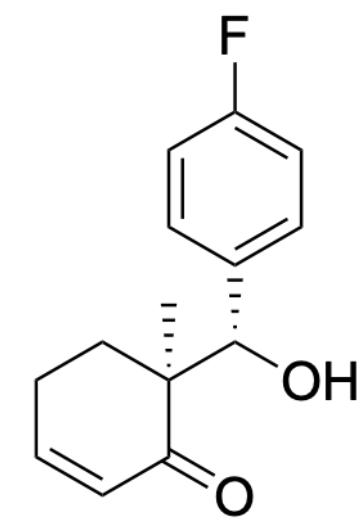

7c

Prepared using general procedure A, using 4-fluorobenzaldehyde **5c** (0.33 mL, 3.00 mmol) and **6** (300 mL, 2.72 mmol) in THF (5 mL), DIPA (0.42 mL, 3.0 mmol) and n-BuLi (1.6 M in hexane, 2.0 mL, 3.0 mmol) at -78 °C for 2 h. The crude product was purified by column chromatography using silica gel and hexane/ethyl acetate (90:10) as the eluent to afford aldol intermediate **7c** as a white solid (335 mg, 53 %). ^1H NMR (400.30 MHz, $CDCl_3$): δ 7.31 – 7.26 (m, 2H), 7.00-6.93 (m, 3H), 5.96-5.94 (d, J = 10.8 Hz, 1H), 4.88 (s, 1H), 2.32-2.30 (m, 2H), 1.79-1.72 (m, 1H), 1.30-1.25 (dt, J = 13.5 Hz, 3.9Hz, 1H), 1.13 (s, 3H). $^{13}C\{^1H\}$ NMR (100.67 MHz, $CDCl_3$): δ = 207.1, 163.5, 161.0, 150.9, 134.8, 129.7, 129.6, 128.2, 114.6, 114.4, 77.4, 47.7, 31.4, 23.0, 14.0. HRMS (ESI-TOF) m/z: $[M+Na]^+$ calcd for $C_{14}H_{15}FO_2Na$ 257.0948; found 257.0946.

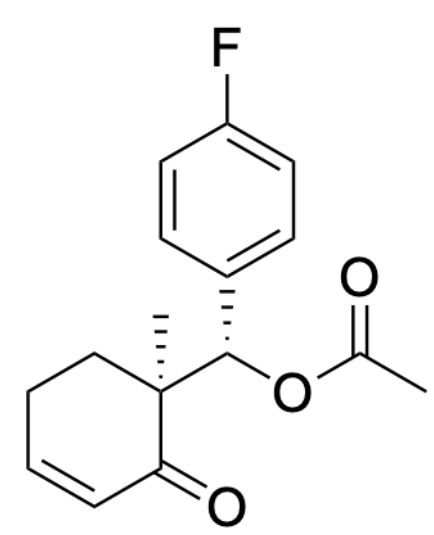

8c

Prepared using general procedure B, **7c** (335 mg, 1.40 mmol) in pyridine (2 mL) and acetic anhydride (2 mL) at rt for 5 h. The crude product was purified by column chromatography using silica gel and hexane/ethyl acetate (90:10) as the eluent to afford intermediate **8c** as a white solid

(320 mg, 81 %).^{1}H NMR (400.30 MHz, $CDCl_3$): δ 7.32-7.28 (m, 2H), 7.01-6.96 (m, 2H), 6.87-6.83 (m, 1H), 6.30 (s, 1H), 5.92-5.88 (d, J = 14.1 Hz, 1H), 2.48-2.34 (m, 2H), 2.08 (s, 3H), 1.87-1.75 (m, 2H), 1.18 (s, 3H). $^{13}C\{^{1}H\}$ (100.67 MHz, $CDCl_3$): δ =200.7, 169.7, 163.5, 161.1, 148.5, 133.1, 129.7, 129.6, 128.3, 115.0, 114.8, 76.3, 49.2, 29.3, 22.9, 21.1, 19.0. HRMS (ESI-TOF) m/z: $[M+Na]^+$ calcd for $C_{16}H_{17}FO_3Na$ 299.1054; found 299.1052.

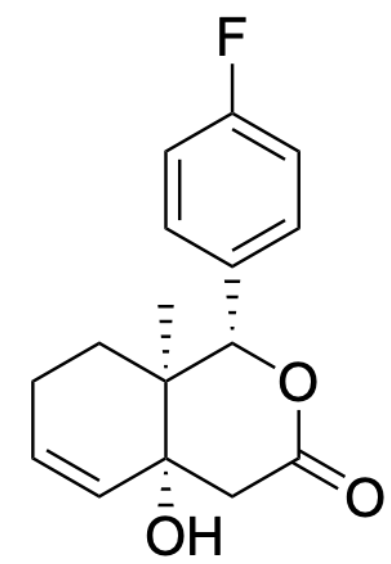

9c

Prepared using general procedure C, using intermediate **8c** (272 mg, 1.00 mmol) in THF (10 mL), DIPA (0.20 mL, 1.5 mmol), and n-BuLi (1.6 M in hexane, 1.0 mL, 1.5 mmol) at -78 ºC for 3 h. The crude product was purified by column chromatography on silica gel using hexanes/ethyl acetate (60:40) as the eluent to afford intermediate **9c** as a white solid (158 mg, 58 %). ^{1}H NMR (400.30 MHz, $CDCl_3$): δ 7.33-7.30 (m, 2H), 7.08-7.04 (m, 2H), 6.03-5.98 (d, J = 17.0 Hz, 1H), 5.78-5.76 (d, J = 9.9 Hz, 1H), 5.24 (s, 1H), 2.95-2.90 (d, J = 17.4 Hz, 1H), 2.84-2.29 (d, J = 17.5 Hz, 1H), 2.31-2.19 (m, 2H), 1.81-1.73 (m, 2H), 1.39-1.33 (m, 1H), 0.88 (s, 3H). $^{13}C\{^{1}H\}$ (100.67 MHz, $CDCl_3$): δ = 170.1, 163.8, 161.3, 133.2, 133.84, 131.6, 130.1, 129.24, 129.16, 115.1, 114.8, 83.6, 69.8, 47.8, 39.8, 27.6, 22.3, 15.1. HRMS (ESI-TOF) m/z: $[M+H]^+$ calcd for $C_{16}H_{18}FO_3$ 277.1234, found 277.1231.

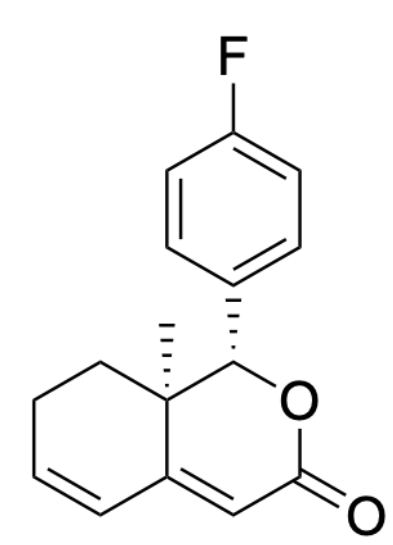

10c

Prepared using general procedure D, using intermediate **9c** (158 mg, 0.57 mmol) in THF (5 mL), pyridine (0.1 mL, 1.15 mmol), and $SOCl_2$ (0.1 mL, 1.15 mmol) at 0 ºC for 20 min. The crude product was washed with 2M HCl, saturated Na_2CO_3, and brine to afford the intermediate **10c** as a brown solid (129 mg, 88 %) without further purification. ^1H NMR (400.30 MHz, $CDCl_3$): δ 7.37-7.33 (m, 2H), 7.09-7.04 (m, 2H), 6.34-6.30 (m, 1H), 6.27-6.24 (d, J = 12.2 Hz, 1H), 5.74 (s, 1H), 5.14 (s, 1H), 2.35-2.21 (m, 2H), 1.60-1.52 (m, 1H), 1.37-1.32 (m, 1H), 0.98 (s, 3H). $^{13}C\{^1H\}$ NMR (1006.7 MHz, $CDCl_3$): δ = 165.7, 164.0, 161.5, 158.6, 138.8, 130.6, 129.6, 125.4, 115.0, 114.8, 112.6, 86.1, 37.4, 30.0, 22.5, 15.8. HRMS (ESI-TOF) m/z: $[M+H]^+$ calcd for $C_{16}H_{16}FO_2$ 259.1129; found 259.1126.

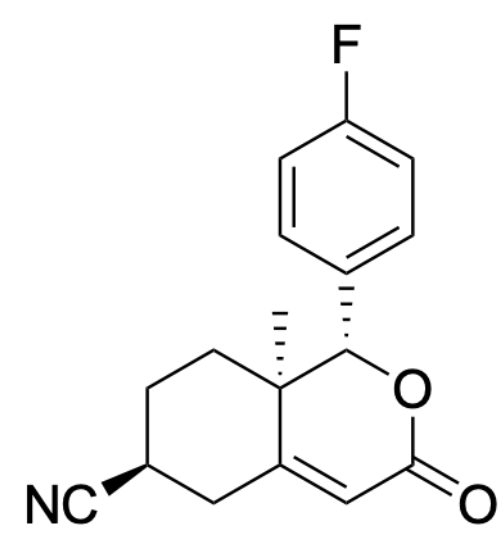

12c

Prepared using general procedure F, using intermediate **10c (**107.8 mg, 0.40 mmol) in DMF/H_2O (8:1 ratio, 9 mL)**,** KCN (6.0 eq, 162 mg, 2.5 mmol), and NH_4Cl (6.0 eq, 133 mg, 2.5 mmol) at 75 º C for 5 days. The resulting crude product was purified by column chromatography using silica gel and hexanes/ethyl acetate (60:40) as the eluent to afford the **12c** (12 mg, 9 %) and **13c** (32 mg, 23 %) as white solids. For **12c :** ^1H NMR (500.30 MHz, $CDCl_3$): δ 7.39-7.36 (m, 2H), 7.10-7.06 (m, 2H), 5.99 (s, 1H), 5.23 (s, 1H), 3.20-3.19 (m, 1H), 2.75-2.72 (dt, J = 18.0 Hz, 4.5 Hz, 1H), 2.67-2.63 (m, 1H), 2.03-1.98 (m, 1H), 1.87-1.80 (m, 2H), 1.43-1.39 (m, 1H), 1.02 (s, 3H). $^{13}C\{^1H\}$ NMR (125.82 MHz, $CDCl_3$): δ = 163.9, 163.8, 161.9, 159.9, 129.81, 129.75, 120.0, 118.0, 115.2, 115.0, 86.3, 38.9, 33.0, 31.0, 27.4, 23.3, 16.9. HRMS (ESI-TOF) m/z: $[M+H]^+$ calcd for $C_{17}H_{17}FNO_2$ 286.1238; found 286.1234.

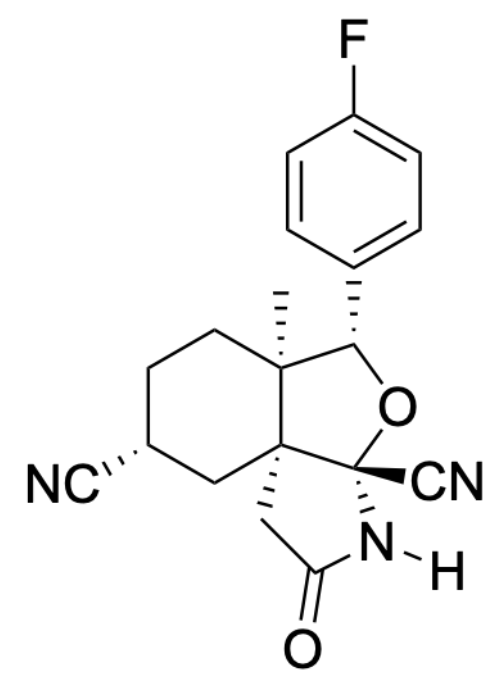

13c

For **13c**: ^{1}H NMR (500.30 MHz, $CDCl_3$): δ 7.19-7.17 (m, 2H), 7.10-7.06 (m, 2H), 6.63 (s, 1H), 5.32 (s, 1H), 3.31-3.29 (m, 1H), 3.04-3.00 (d, J = 17.9 Hz, 1H), 2.52-2.47 (m, 2H), 2.37-2.33 (d, J = 14.3 Hz, 1H), 2.03-1.95 (m, 2H), 1.81-1.69 (m, 2H), 0.78 (s, 3H). $^{13}C\{^{1}H\}$ NMR (100.67 MHz, $CDCl_3$): δ = 173.3, 164.4, 161.9, 130.8, 128.24, 128.15, 120.4, 116.2, 116.0, 115.7, 91.9, 87.5, 54.7 46.1, 40.3, 35.5, 25.2, 24.0, 20.1. HRMS (ESI-TOF) m/z: $[M+H]^+$ calcd for $C_{19}H_{19}FN_3O_2$ 340.1456; found 340.1450.

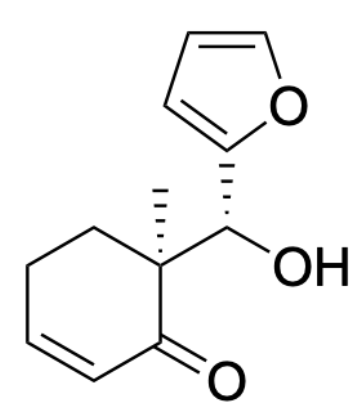

7d

Prepared using general procedure A, using 2-furaldehyde **5d** (0.25 mL, 3.0 mmol) and **6** (0.30 mL, 2.7 mmol) in THF (4 mL), DIPA (0.42 mL, 3.0 mmol) and n-BuLi (2.5 M in hexane, 1.2 mL, 3.0 mmol) at -78 ºC for 2 h. The crude product was purified by column chromatography using silica gel and hexane/ethyl acetate (90:10) as the eluent to afford aldol intermediate **7d** as a white solid (0.30 mg, 5 %). ^{1}H NMR (500.30 MHz, $CDCl_3$) : δ 7.36 (d, J = 5.1 Hz, 1H) , 6.99 -6.96 (m, 1H), 6.35-6.34 (d, J = 4.9 Hz, 1H), 6.31 (dd, J = 4.7 Hz, 5.0 Hz 1H), 5.98-5.96 (d, J = 13.8 Hz, 1H), 4.95 (s, 1H), 4.40 (s, 1H), 2.40-2.37 (m, 2H), 1.81-1.76 (m, 1H), 1.42-1.39 (dt, J = 13.7 Hz, 3.6 Hz 1H), 1.28 (s, 3H). $^{13}C\{^{1}H\}$ NMR (125.82 MHz, $CDCl_3$): δ =206.5, 153.2, 150.7, 141.8, 128.1, 110.2, 108.4, 72.3, 48.0, 30.8, 22.9, 14.8 HRMS (ESI-TOF) m/z: $[M+Na]^+$ calcd for $C_{12}H_{14}O_3Na$ 229.0835; found 229.0837.

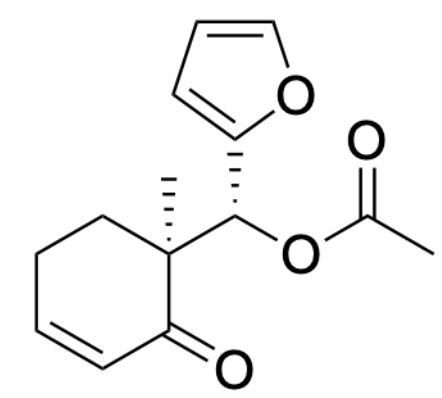

8d

Prepared using general procedure B, **7d** (19.5 mg, 0.09 mmol) in pyridine (0.2 mL) and acetic anhydride (0.2 mL) at rt for 5 h. The crude product was purified by column chromatography using silica gel and hexane/ethyl acetate (90:10) as the eluent to afford intermediate **8d** as a white solid (21 mg, 91 %).^{1}H NMR (500.30 MHz, $CDCl_3$): δ 7.36 (d, J = 3.8 Hz, 1H), 6.90 -6.87 (m, 1H), 6.33-6.32 (m, 2H), 6.29 (s, 1H), 5.94-5.92 (d, J = 10.1 Hz, 1H), 2.54-2.29 (m, 1H), 2.36-2.31 (m, 1H), 2.06 (s, 3H), 1.86-1.84 (t, J = 6.1 Hz, 2H), 1.24 (s, 3H). ^{13}C{^{1}H} NMR (125.82 MHz, $CDCl_3$): δ = 200.6, 169.7, 150.7, 148.5, 142.2, 128.4, 110.3, 109.3, 70.9, 48.8, 30.7, 22.9, 20.8, 18.0. HRMS (ESI-TOF) m/z: $[M+Na]^+$ calcd for $C_{14}H_{16}O_4Na$ 271.0941; found 271.0940.

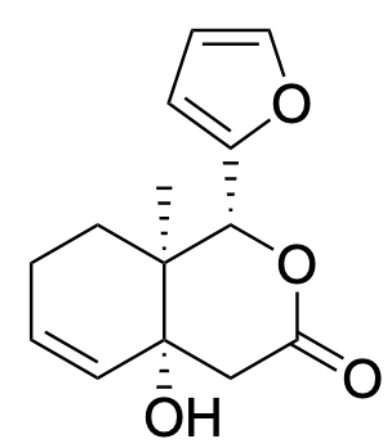

9d

Prepared using general procedure C, using intermediate **8d** (27 mg, 0.1 mmol) in THF (1 mL), DIPA (0.02 mL, 0.12 mmol) and n-BuLi (2.5 M in hexane, 0.08 mL, 0.12 mmol) at -78 ºC for 3 h. The crude product was purified by column chromatography to afford the intermediate **9d** as a white solid (17 mg, 65 %). ^{1}H NMR (600.21 MHz, $CDCl_3$) : δ 7.41 (d, J = 3.0 Hz, 1H), 6.42 (d, J = 3.2 Hz, 1H), 6.40-6.39 (d, J = 4.7 Hz, 1H), 5.99-5.96 (d, J = 16.9 Hz, 1H), 5.76-5.74 (d, J = 9.8 Hz, 1H), 5.33 (s, 1H), 2.95-2.92 (d, J = 18.2 Hz, 1H), 2.82-2.79 (d, J = 18.2 Hz, 1H), 2.30-2.24 (m, 1H), 2.16-2.10 (dd, J = 14.6 Hz, 4.3Hz 1H), 1.82-1.77 (m, 2H), 1.45-1.41 (dd, J = 14.2 Hz, 6.2 Hz 1H), 1.08 (s, 3H). ^{13}C{^{1}H} NMR (150.94 MHz, $CDCl_3$): δ = 169.7, 149.8, 142.4, 131.5, 131.3, 110.4, 109.5, 78.1, 69.4, 42.0, 40.0, 27.2, 22.1, 15.2. HRMS (ESI-TOF) m/z: $[M+Na]^+$ calcd for $C_{14}H_{16}O_4Na$ 271.0941; found 271.0941.

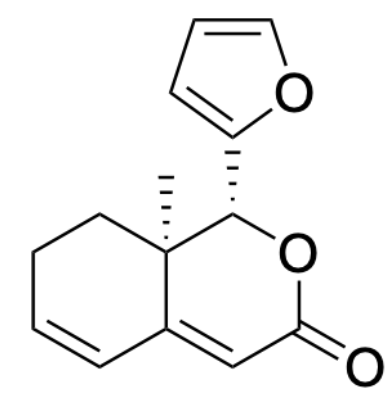

10d

Prepared using general procedure D, using intermediate **9d** (17.5 mg, 0.07 mmol) in THF (1 mL), pyridine (0.01 mL, 0.14 mmol), and $SOCl_2$ (0.01 mL, 0.14 mmol) at 0 °C for 20 min. The crude product was washed with 2M HCl, saturated Na_2CO_3, and brine to afford the intermediate **10d** as a light brown solid (6.9 mg, 43 %) without further purification. ^{1}H NMR (500.30 MHz, $CDCl_3$) : δ 7.42 (d, J = 2.6 Hz, 1H), 6.48-6.47 (d, J = 3.3 Hz, 1H), 6.41-6.40 (d, J = 5.0 Hz, 1H), 6.35-6.31 (m, 1H), 6.24-6.22 (d, J = 9.8 Hz, 1H), 5.70 (s, 1H), 5.17 (s, 1H), 2.34-2.29 (m, 2H), 1.55-1.53 (m, 2H), 1.15 (s, 3H). ^{13}C{^{1}H} NMR (125.82 MHz, $CDCl_3$): δ = 165.3, 158.6, 149.1, 142.6, 139.0, 125.1, 112.3, 110.4, 110.0, 80.9, 37.4, 29.8, 22.5, 16.5 HRMS (ESI-TOF) m/z: $[M+Na]^+$ calcd for $C_{14}H_{14}O_3Na$ 253.0835, found 253.0831.

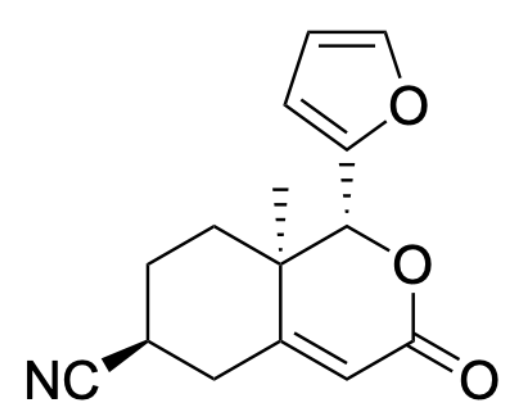

12d

Prepared using general procedure F, using intermediate **10d (**6.9 mg, 0.03 mmol) in DMF/H_2O (1.6:0.2 ratio, 1.8 mL)**,** KCN (6.0 eq, 12 mg, 0.18 mmol), and NH_4Cl (6.0 eq, 10 mg, 0.18 mmol) at 75 °C for 5 days. The resulting crude product was purified by column chromatography on silica gel using hexanes/ethyl acetate (60:40) as the eluent to afford **12d** as a white solid (4.5 mg, 58 %). ^{1}H NMR (500.30 MHz, $CDCl_3$) : δ 7.44-7.43 (d, J = 2.6 Hz, 1H), 6.50-6.49 (d, J = 3.3 Hz, 1H), 6.42-6.41 (d, J = 5.1 Hz, 1H), 5.95 (s, 1H), 5.25 (s, 1H), 3.19-2.74 (m, 1H), 2.69-2.63 (m, 2H), 2.05-2.01 (m, 1H), 1.87-1.83 (m, 2H), 1.69-1.59 (m, 1H) 1.20 (s, 3H). ^{13}C{^{1}H} NMR (125.82 MHz, $CDCl_3$): δ = 163.4, 160.1, 148.3, 142.9, 119.9, 117.8, 110.5, 110.4, 81.0, 38.9, 32.8, 30.7, 27.4, 23.4, 17.5 HRMS (ESI-TOF) m/z: $[M+H]^+$ calcd for $C_{15}H_{16}NO_3$ 258.1125, found 258.1123.

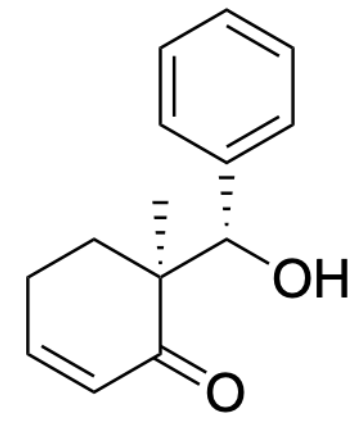

7e

Prepared using general procedure A, using benzaldehyde **5e** (0.5 mL, 5.0 mmol) and **6** (0.5 mL, 4.5 mmol) in THF (8 mL), DIPA (0.7 mL, 5.0 mmol) and n-BuLi (1.6 M in hexane, 3.12 mL, 5.0 mmol) at -78 ºC for 2 h. The crude product was purified by column chromatography using silica gel and hexane/ethyl acetate (90:10) as the eluent to afford aldol intermediate **7e** as a light-yellow solid (578.2 mg, 60 %). ^{1}H NMR (500.30 MHz, $CDCl_3$) : δ 7.36-7.28 (m, 5H), 6.98-6.94 (m, 1H), 6.00-5.98 (d, J = 10.0 Hz, 1H), 4.92 (s, 1H), 4.73 (s, 1H), 2.34-2.31 (m, 2H), 1.84-1.78 (m, 1H), 1.34-1.30 (d, J = 16.3 Hz, 1H), 1.18 (s, 3H). $^{13}C\{^{1}H\}$ NMR (125.82 MHz, $CDCl_3$): δ = 207.3, 150.5, 138.9, 128.3, 128.1, 127.70, 127.69, 77.7, 47.8, 31.6, 23.01, 14.1. HRMS (ESI-TOF) m/z: $[M+Na]^+$ calcd for $C_{14}H_{16}O_2Na$ 239.1043; found 239.1039.

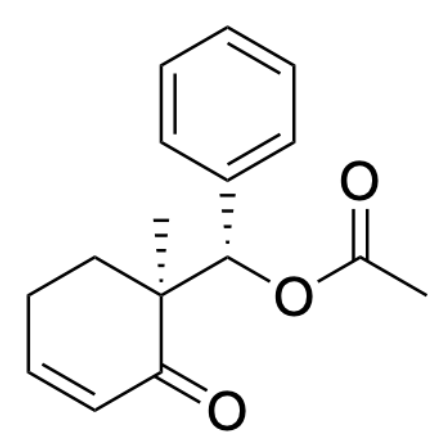

8e

Prepared using general procedure B, **7e** (578.7 mg, 2.67 mmol) in pyridine (3 mL) and acetic anhydride (3 mL) at rt for 5 h. The crude product was purified by column chromatography using silica gel and hexane/ethyl acetate (90:10) as the eluent to afford intermediate **8e** as a white solid (423 mg, 61 %). ^{1}H NMR (500.30 MHz, $CDCl_3$): δ 7.30-7.28(m, 3H), 7.25-7.22 (m, 2H), 6.83-6.79 (m, 1H), 6.27 (s, 1H), 5.89-5.57 (d, J = 12.1 Hz, 1H), 2.47-2.40 (m, 1H), 2.35-2.29 (m, 1H), 2.04 (s, 3H), 1.78-1.75 (m, 2H), 1.14 (s, 3H). $^{13}C\{^{1}H\}$ NMR (125.82 MHz, $CDCl_3$): δ = 200.9, 169.7, 148.3, 137.2, 128.8, 127.93, 127.89, 127.8, 49.1, 29.8, 23.0, 21.1, 18.8. HRMS (ESI-TOF) m/z: $[M+Na]^+$ calcd for $C_{16}H_{18}O_3Na$ 281.1148, found 281.1142.

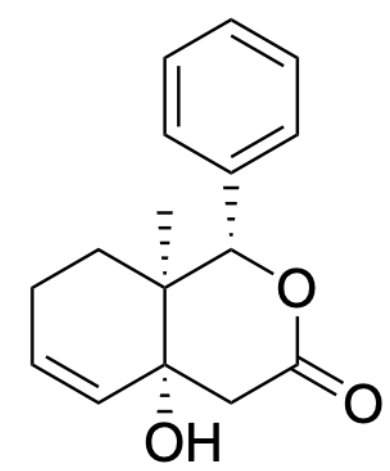

9e

Prepared using general procedure C, using intermediate **8e** (323.1 mg, 1.25 mmol) in THF (8 mL), DIPA (0.2 mL, 1.38 mmol) and n-BuLi (1.6 M in hexane, 0.9 mL, 1.38 mmol) at -78 ^{o}C for 3 h. The crude product was purified by column chromatography to afford the intermediate **9e** as a light yellow solid (116.2 mg, 45 %). ^{1}H NMR (500.30 MHz, $CDCl_3$): δ 7.38-7.33 (m, 5H), 6.02-65.99 (m, 1H), 5.78-5.76 (d, J = 11.5 Hz, 1H), 5.26 (s, 1H), 2.95-2.91 (d, J = 17.4 Hz, 1H), 2.84-2.82 (d, J = 17.4 Hz, 1H), 2.36-2.29 (m, 1H), 2.24-2.15 (m, 1H), 1.80-1.74 (m, 2H), 1.42-1.36 (m, 1H), 0.89 (s, 3H). ^{13}C{^{1}H} NMR (125.82 MHz, $CDCl_3$): δ =170.2, 135.9, 131.6, 130.9, 128.3, 127.9, 127.5, 84.3, 69.8, 41.8, 39.8, 27.6, 22.4, 15.2. HRMS (ESI-TOF) m/z: $[M+Na]^+$ calcd for $C_{16}H_{18}O_3Na$ 281.1148, found 281.1143.

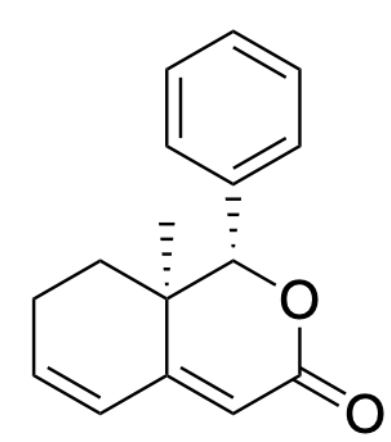

10e

Prepared using general procedure D, using intermediate **9e** (116.2 mg, 0.45 mmol) in THF (5.5 mL), pyridine (0.07 mL, 0.9 mmol), and $SOCl_2$ (0.07 mL, 0.9. mmol) at 0 ^{o}C for 20 min. The crude product was washed with 2M HCl, saturated Na_2CO_3, and brine to afford the intermediate **10e** as a brown solid (100 mg, 92 %) without further purification. ^{1}H NMR (500.30 MHz, $CDCl_3$): δ 7.36 – 7.33 (m, 5H), 6.32-6.29 (m, 1H), 6.24-6.22 (d, J = 9.8 Hz, 1H), 5.71 (s, 1H), 5.13 (s, 1H), 2.33-2.26 (m, 1H), 2.23-2.18 (m, 1H), 1.59-1.53 (m, 1H), 1.38-1.33 (m, 1H), 0.97 (s, 3H). ^{13}C{^{1}H} NMR (125.82 MHz, $CDCl_3$): δ = 165.95, 158.8, 138.9, 134.7, 128.5, 128.0, 127.9, 125.3, 112.6, 37.4, 30.0, 22.6, 15.9. HRMS (ESI-TOF) m/z: $[M+Na]^+$ calcd for $C_{16}H_{16}O_2Na$ 263.1043; found 263.1038.

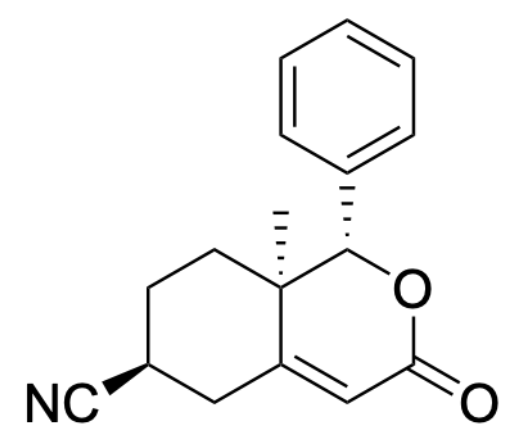

12e

Prepared using general procedure F, using intermediate **10e (**100 mg, 0.42 mmol) in DMF/H_2O (8:1 ratio, 9 mL)**,** KCN (6.0 eq, 172.75 mg, 2.5 mmol), and NH_4Cl (6.0 eq, 133.75 mg, 2.5 mmol) at 75 °C for 5 days. The resulting crude product was purified by column chromatography on silica gel using hexanes/ethyl acetate (60:40) as the eluent to afford **12e** as a white solid (40.5 mg, 58 %). ^{1}H NMR (500.30 MHz, $CDCl_3$): δ 7.26 (m, 5H), 5.98 (s, 1H), 5.23 (s, 1H), 3.19 (m, 1H), 2.74-2.62 (m, 2H), 2.03-1.99 (dd, J = 13.3 Hz, 2.7 Hz 1H), 1.92 – 1.76 (m, 2H), 1.44-1.42 (d, J = 13.5 Hz, 1H), 1.03 (s, 3H). ^{13}C{^{1}H} NMR (125.82 MHz, $CDCl_3$): δ = 164.07, 160.10, 134.1, 128.8, 128.1, 128.0, 120.1, 118.0, 87.0, 39.0, 33.0, 31.0, 27.3, 17.0. HRMS (ESI-TOF) m/z: $[M+H]^+$ calcd for $C_{17}H_{18}O_2$ 268.1332; found 268.1327.

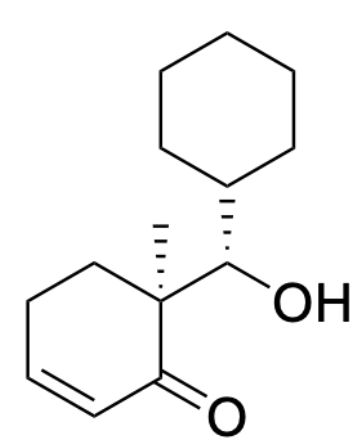

7f

Prepared using general procedure A, using cyclohexane carbaldehyde **5f** (0.5 mL, 4.0 mmol) and **6** (400 mg, 3.6 mmol) in THF (8 mL), DIPA (0.6 mL, 4.0 mmol) and n-BuLi (1.6 M in hexane, 2.5 mL, 4.0 mmol) at -78 °C for 2 h. The crude product was purified by column chromatography using silica gel and hexane/ethyl acetate (90:10) as the eluent to afford aldol intermediate **7f** as a light-yellow solid (310 mg, 38 %). ^{1}H NMR (500.30 MHz, $CDCl_3$): δ 6.89-6.86 (m, 1H), 5.87-5.85 (d, J = 10.1 Hz, 1H), 3.66-3.65 (d, J = 4.3 Hz, 1H), 2.43-2.31 (m, 2H), 1.84-1.77 (m, 3H), 1.71-1.67 (m, 2H), 1.59-157 (m, 1H), 1.39-1.38 (m, 2H), 1.25-1.21 (m, 3H), 1.15 (m, 3H), 1.11 (s, 3H). ^{13}C{^{1}H} NMR (125.82 MHz, $CDCl_3$): δ = 205.3, 149.2, 128.3, 78.1, 48.5, 40.0, 32.2, 29.2, 28.3, 26.8, 26.4, 26.2, 22.9, 17.7. HRMS (ESI-TOF) m/z: $[M+H]^+$ calcd for $C_{14}H_{23}O_2$ 223.1693; found 223.1690.

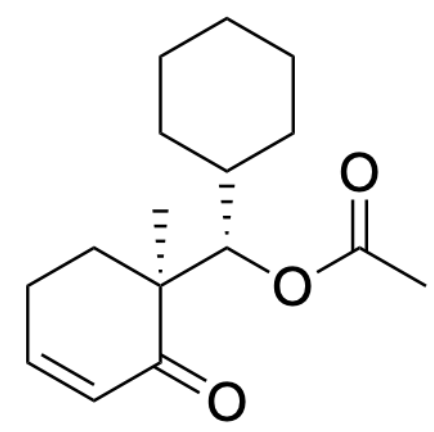

8f

Prepared using general procedure B, **7f** (310 mg, 1.4 mmol) in pyridine (2 mL) and acetic anhydride (2 mL) at rt for 5 h. The crude product was purified by column chromatography using silica gel and hexane/ethyl acetate (90:10) as the eluent to afford intermediate **8f** as a light yellow oil (266.7 mg, 72 %).^{1}H NMR (500.30 MHz, $CDCl_3$): δ 6.87-8.83 (m, 1H), 5.91-5.89 (d, J = 10.1 Hz, 1H), 5.35-5.33 (d, J = 7.1 Hz, 1H), 2.36-2.35 (m, 2H), 2.03 (s, 3H), 1.83-1.78 (m, 1H), 1.69-1.55 (s, 5H), 1.49-1.39 (m, 2H), 1.18-1.05 (m, 4H), 1.03 (s, 3H), 1.00 (m, 1H). ^{13}C{^{1}H} NMR (125.82 MHz, $CDCl_3$): δ = 200.7, 170.6, 148.2, 128.8, 79.3, 48.2, 39.7, 31.4, 29.6, 27.8, 26.5, 26.13, 26.11, 22.6, 20.8, 20.0. HRMS (ESI-TOF) m/z: $[M+H]^+$ calcd for $C_{16}H_{25}O_3$ 265.1798; found 265.1797.

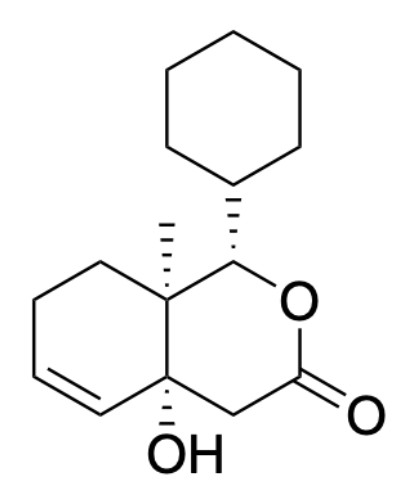

9f

Prepared using general procedure C, using intermediate **8f** (101 mg, 0.38 mmol) in THF (4 mL), DIPA (0.88 mL, 0.57 mmol) and n-BuLi (1.6 M in hexane, 1.0 mL, 0.57 mmol) at -78 °C for 3 h to afford the intermediate **9f** as a yellow solid (85 mg, 84 %). The intermediate (**9f**) was used in the subsequent step without purification because in we luckily observed 100 % conversion of starting material to product when ^{1}H NMR of the crude product was taken. ^{1}H NMR (500.30 MHz, $CDCl_3$): δ 5.88-5.84 (m, 1H), 5.62-5.60 (d, J = 9.9 Hz, 1H), 3.92-3.91 (s, 1H), 2.75-2.71 (d, J = 17.4 Hz, 1H), 2.67-2.63 (d, J = 17.4 Hz, 1H), 2.11-2.00 (m, 1H), 1.9-1.69 (m, 8H), 1.51–1.33 (m, 4H), 1.29-1.16 (m, 3H), 1.10 (s, 3H). ^{13}C{^{1}H} NMR (125.82 MHz, $CDCl_3$): δ = 171.1, 131.9,

130.4, 86.4, 70.2, 41.9, 39.7, 38.6, 33.4, 28.6, 27.8, 26.8, 26.2, 26.0, 22.2, 15.1. HRMS (EI-QTOF) m/z: $[M]^{+\cdot}$ calcd for $C_{16}H_{24}O_3$ 264.1720; found 264.1715.

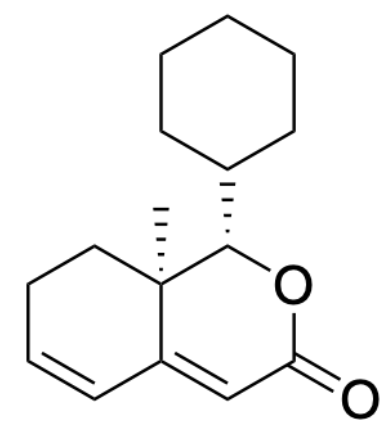

10f

Prepared using general procedure D, using intermediate **9f** (85 mg, 0.32 mmol) in THF (4 mL), pyridine (0.06 mL, 0.64 mmol), and $SOCl_2$ (0.06 mL, 0.64 mmol) at 0 °C for 20 min. The crude product was washed with 2M HCl, saturated Na_2CO_3, and brine to afford the intermediate **10f** as a dark brown solid (75 mg, 95 %) without further purification. ^{1}H NMR (500.30 MHz, $CDCl_3$): δ 6.29-6.25 (m, 1H), 6.15-6.13 (d, J = 9.8 Hz, 1H), 5.59 (s, 1H), 3.86 (s, 1H), 2.37-2.34 (m, 2H), 1.91-1.87 (m, 1H), 1.80-1.74 (m, 3H), 1.70-1.68 (m, 3H), 1.62-1.57 (m, 2H), 1.54-1.51 (m, 2H), 1.42-1.36 (m, 1H), 1.22-1.20 (m, 1H), 1.13 (s, 3H). $^{13}C\{^1H\}$ NMR (125.82 MHz, $CDCl_3$): δ = 166.6, 159.2, 138.0, 125.2, 89.0, 37.5, 37.4, 33.2, 30.2, 27.6, 26.8, 26.4, 26.0, 22.6, 16.7. HRMS (EI-QTOF) m/z: $[M]^{+\cdot}$ calcd for $C_{16}H_{22}O_2$ 246.1614; found 246.1601.

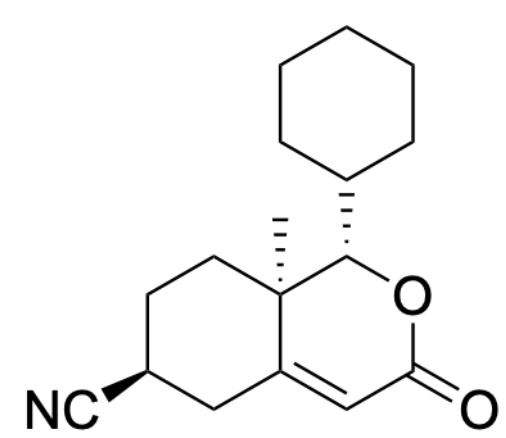

12f

Prepared using general procedure F, using intermediate **10f (**100 mg, 0.42 mmol) in DMF/H_2O (8:1 ratio, 9 mL)**,** KCN (6.0 eq, 172.75 mg, 2.5 mmol), and NH_4Cl (6.0 eq, 133.75 mg, 2.5 mmol) at 75 °C for 5 days. The resulting crude product was purified by column chromatography using silica gel and hexanes/ethyl acetate (60:40) as the eluent to afford **12f** as a yellow solid (25 mg, 30 %). ^{1}H NMR (500.30 MHz, $CDCl_3$): δ 5.83 (s, 1H), 3.92 (s, 1H), 3.16-3.15 (d, J = 7.0 Hz, 1H), 2.61-2.60 (d, J = 5.1 Hz, 2H), 2.06-2.02 (d, J = 17.9 Hz, 1H), 1.80-1.77 (m, 6H), 1.65-1.63 (m, 4H), 1.58-1.56 (m, 2H), 1.33 – 1.23 (m, 2H), 1.17 (s, 3H). $^{13}C\{^1H\}$ NMR (125.82 MHz, $CDCl_3$):

δ = 164.6, 160.2, 120.0, 118.1, 89.3, 39.1, 37.3, 33.3, 32.6, 31.2, 27.7, 27.4, 26.8, 26.4, 25.9, 23.5, 17.5 HRMS (ESI-TOF) m/z: $[M+H]^+$ calcd for $C_{17}H_{24}NO_2$ 274.1802; found 274.1797.

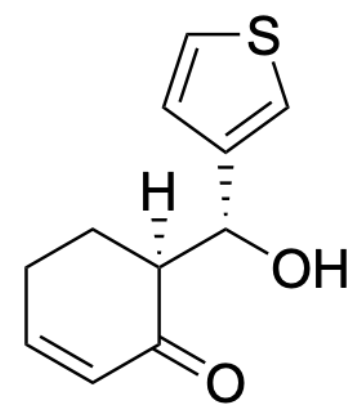

SI1

Prepared using general procedure A, using 3-thiophene carbaldehyde **5g** (0.6 mL, 5.7 mmol) and 2-cyclohexen-1-one (0.5 mL, 5.2 mmol) in THF (10 mL), DIPA (0.8 mL, 5.7 mmol) and n-BuLi (2.5 M in hexane, 2.3 mL, 5.7 mmol) at -78 ºC for 2 h. The crude product was purified by column chromatography using silica gel and hexane/ethyl acetate (90:10) as the eluent to afford aldol Intermediate **SI1** as a light-yellow solid (700 mg, 47 %). ^{1}H NMR (600.21 MHz, $CDCl_3$): δ 7.30-7.28 (d, J = 7.9 Hz, 1H), 7.20 (s, 1H), 7.09-7.08 (d, J = 8.0 Hz, 1H), 7.02-6.99 (m, 1H), 6.05-6.04 (d, J = 10.1 Hz, 1H), 5.00-4.98 (d, J = 8.6 Hz, 1H), 2.60-2.56 (m, 1H), 2.33-2.31 (m, 2H), 1.65-1.61 (m, 1H), 1.58-1.50 (s, 1H). $^{13}C\{^1H\}$ NMR (150.94 MHz, $CDCl_3$): δ = 203.2, 151.9, 142.5, 129.5, 126.1, 126.0, 122.4, 71.1, 52.5, 25.7, 25.1. HRMS (ESI-TOF) m/z: $[M+Na]^+$ calcd for $C_{11}H_{12}SO_2Na$ 231.0450, found 231.0450.

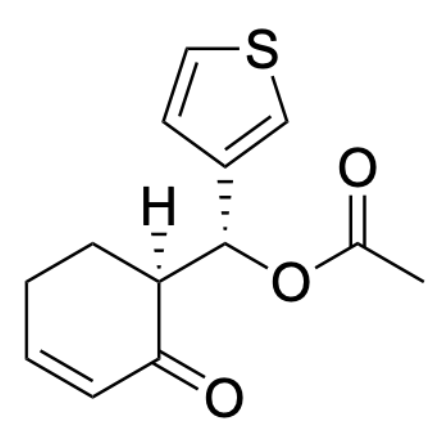

SI2

Prepared using general procedure B, **SI1** (207.2 mg, 1.00 mmol) in pyridine (1.5 mL) and acetic anhydride (1.5 mL) at rt for 5 h. The crude product was purified by column chromatography using silica gel and hexane/ethyl acetate (90:10) as the eluent to afford intermediate **SI2** as a light yellow solid (169 mg, 68 %).^{1}H NMR (500.30 MHz, $CDCl_3$): δ 7.20-7.18 (m, 2H), 7.00-6.98 (d, J = 6.2 Hz, 1H), 6.86-6.83 (m, 1H), 6.50-.49 (d, J = 5.9 Hz, 1H), 5.92-5.89 (d, J = 14.0 Hz, 1H), 2.86-2.82 (m, 1H), 2.31-2.27 (m, 2H), 2.01 (s, 3H), 1.54-1.46 (m, 1H). $^{13}C\{^1H\}$ NMR (125.82 MHz, $CDCl_3$):

δ = 197.8, 169.7, 149.8, 138.7, 129.5, 126.5, 125.7, 122.9, 70.4, 51.2, 24.9, 23.7, 21.1. HRMS (ESI-TOF) m/z: $[M+Na]^+$ calcd for $C_{13}H_{14}O_3SNa$ 273.0556; found 273.0554.

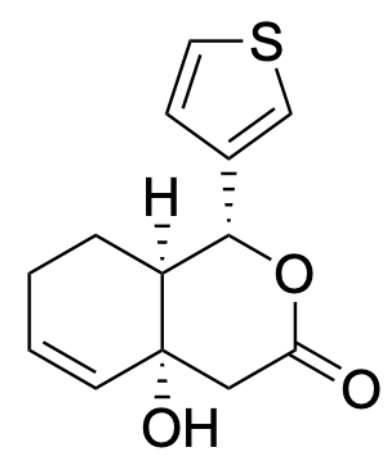

SI3

Prepared using general procedure C, using intermediate **SI2** (169 mg, 0.67 mmol) in THF (4 mL), DIPA (0.1 mL, 0.74 mmol), and n-BuLi (2.5 M in hexane, 0.3 mL, 0.74 mmol) at -78 °C for 3 h. The crude product was purified by column chromatography using silica gel and hexanes/ethyl acetate (60:40) as the eluent to afford the intermediate **SI3** as a white solid (32 mg, 19 %). ^{1}H NMR (600.21 MHz, $CDCl_3$): δ 7.37 (d, J = 7.9 Hz, 1H), 7.36 (s, 1H), 7.17 (d, J = 8.1 Hz, 1H), 6.00-5.98 (d, J = 9.9 Hz, 1H), 5.70-5.69 (d, J = 9.9 Hz, 1H), 5.04-5.03 (d, J = 13.2 Hz, 1H) , 2.81-2.78 (d, J = 16.0 Hz, 1H), 2.76-2.73 (d, J = 16.0 Hz, 1H), 2.34-2.30 (m, 1H), 2.04-2.20 (m, 2H), 1.89-1.82 (m, 1H), 1.39 (m, 1H), 1.43-1.36 (m, 1H). ^{13}C{^{1}H} NMR (150.94 MHz, $CDCl_3$): δ = 170.5, 139.0, 130.9, 130.4, 126.8, 126.1, 123.8, 78.5, 68.6, 46.6, 42.9, 22.6, 22.2. HRMS (ESI-TOF) m/z: $[M+Na]^+$calcd for $C_{13}H_{14}SO_3Na$ 273.0556; found 273.0555.

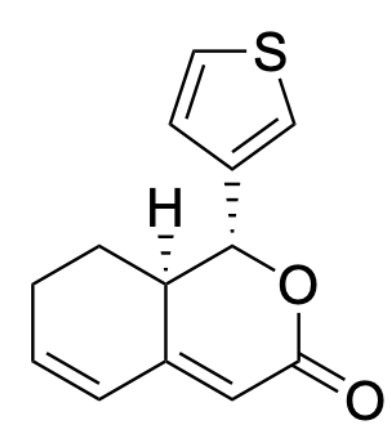

14g

Prepared using general procedure D, using intermediate **SI3** (32 mg, 0.13 mmol) in THF (1.5 mL), pyridine (0.02 mL, 0.26 mmol), and $SOCl_2$ (0.02 mL, 0.26 mmol) at 0 °C for 20 min. The crude product was washed with 2M HCl, saturated Na_2CO_3, and brine to afford the intermediate **14g** as a brown solid (8 mg, 28 %) without further purification. ^{1}H NMR (500.30 MHz, $CDCl_3$): δ 7.37-7.35 (d, J = 11.0 Hz, 2H), 7.18-7.16 (d, J = 6.3 Hz, 1H), 6.40-6.36 (m, 1H), 6.32-6.30 (d, J = 12.3 Hz, 1H), 5.80 (s, 1H), 5.14-5.11 (d, J = 11.6 Hz, 1H), 2.82-2.76 (m, 1H), 2.35 (m, 1H), 2.21 (m,

1H), 1.66-1.62 (m, 1H), 1.36-1.30 (m, 1H). $^{13}C\{^{1}H\}$ NMR (125.82 MHz, $CDCl_3$): δ = 165.5, 153.2, 140.3, 138.4, 126.6, 126.09, 126.05, 124.3, 113.2, 80.1, 39.27, 25.3, 24.0. HRMS (ESI-TOF) m/z: $[M+H]^+$calcd for $C_{13}H_{13}O_2S$ 233.0631; found 233.0629.

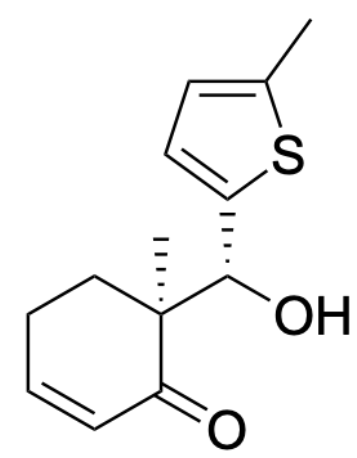

7h

Prepared using general procedure A, using 5-methyl-2-thiophenecarbaldehyde **5h** (0.5 mL, 5.0 mmol) and **6** (0.50 mL, 4.5 mmol) in THF (10 mL), DIPA (0.80 mL, 5.7 mmol) and n-BuLi (2.5 M in hexane, 2.3mL, 5.7 mmol) at -78 °C for 2 h. The crude product was purified by column chromatography using silica gel and hexane/ethyl acetate (90:10) as the eluent to afford aldol intermediate **7h** as a white solid (0.60 g, 56%). ^{1}H NMR (600.21 MHz, $CDCl_3$): 6.98-6.95 (m, 1H), 6.71-6.70 (d, J = 3.4 Hz, 1H), 6.60-6.59 (d, J = 3.1 Hz, 1H), 5.97-5.95 (d, J = 10.0 Hz, 1H), 5.09 (s, 1H), 4.58 (s, 1H), 2.44 (s, 3H), 2.38-2.34 (m, 2H), 1.79-1.74 (m, 1H), 1.53 (dt, J = 13.7, 3.5 Hz, 1H), 1.26 (s, 3H). $^{13}C\{^{1}H\}$ NMR (125.82 MHz, $CDCl_3$): δ = 206.8, 150.7, 139.7, 139.4, 128.2, 125.8, 124.1, 74.8, 47.9, 31.2, 23.0, 15.3, 14.6. HRMS (ESI-TOF) m/z: $[M+Na]^+$calcd for $C_{13}H_{16}O_2SNa$ 259.07763; found 259.0760.

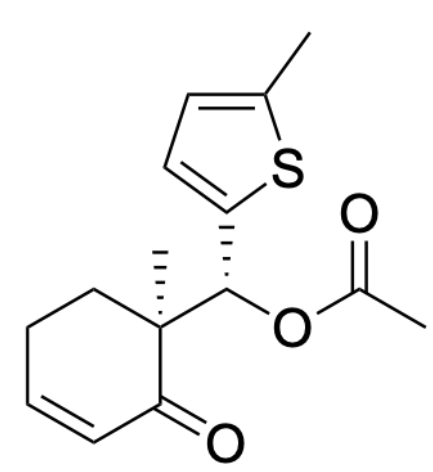

8h

Prepared using general procedure B, **7h** (225.2 mg, 0.95 mmol) in pyridine (4 mL) and acetic anhydride (3 mL) at rt for 5 h. The crude product was purified by column chromatography using silica gel and hexane/ethyl acetate (90:10) as the eluent to afford intermediate **8h** as a white solid (184 mg, 69 %). ^{1}H NMR (500 MHz, $CDCl_3$) : δ 6.88-6.85 (m, 1H), 6.75-6.74 (d, J = 3.5 Hz, 1H), 6.58-6.57 (dd, J = 3.5 Hz, 1.2 Hz 1H), 6.50 (s, 1H), 5.93-5.91 (dt, J = 10.1 Hz, 2.0 Hz 1H), 2.49-

2.47 (m, 1H), 2.42 (s, 3H), 2.42– 2.41 (m, 1H), 2.05 (s, 3H), 1.98-1.93 (m, 1H), 1.86-1.81 (m, 1H), 1.21 (s, 3H). $^{13}C\{^{1}H\}$ NMR (125.82 MHz, $CDCl_3$): δ = 200.9, 169.6, 148.6, 139.6, 137.2, 128.6, 126.7, 124.5, 73.8, 49.3, 29.8, 22.9, 20.9, 18.4, 15.1. HRMS (ESI-TOF) m/z: $[M+ Na]^+$calcd for $C_{15}H_{18}O_3SNa$ 301.0869; found 301.0869.

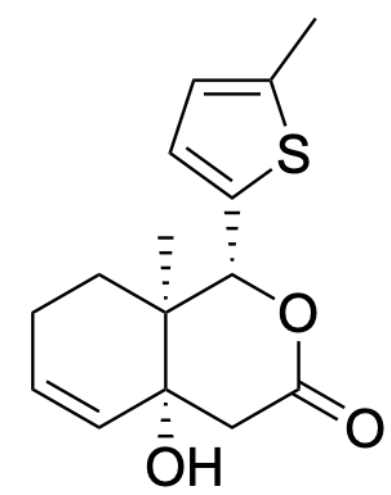

9h

Prepared using general procedure C, using intermediate **8h** (184 mg, 0.66 mmol) in THF (3 mL), DIPA (0.1 mL, 0.73 mmol) and n-BuLi (2.5 M in hexane, 0.3 mL, 0.73 mmol) at -78 ºC for 3 h. The crude product was purified by column chromatography to afford the intermediate **9h** as a white solid (270 mg, 40 %). ^{1}H NMR (500 MHz, $CDCl_3$) : δ 6.85-6.84 (d, J = 3.5 Hz, 1H), 6.66-6.65 (dd, J = 6 Hz, 1.1Hz 1H), 6.01-5.97 (m, 1H), 5.78-5.76 (dt, J = 9.8 Hz, 2.2 Hz 1H), 5.46 (s, 1H), 2.94-2.91 (d, J = 18.0 Hz, 1H), 2.81-2.77 (d, J = 18.1 Hz, 1H), 2.47 (s, 3H), 2.27-2.13 (m, 2H), 1.82-1.76 (m, 1H), 1.65 (s, 1H), 1.51-1.47 (m, 1H), 1.10 (s, 3H). $^{13}C\{^{1}H\}$ NMR (125.82 MHz, $CDCl_3$): δ =1.69.6, 140.3, 135.5, 131.8, 131.1, 126.9, 124.4, 80.6, 69.5, 41.9, 40.1, 27.1, 22.2, 15.2, 15.0. HRMS (ESI-TOF) m/z: $[M+ H]^+$calcd for $C_{15}H_{19}O_3S$ 279.1049; found = 279.1050.

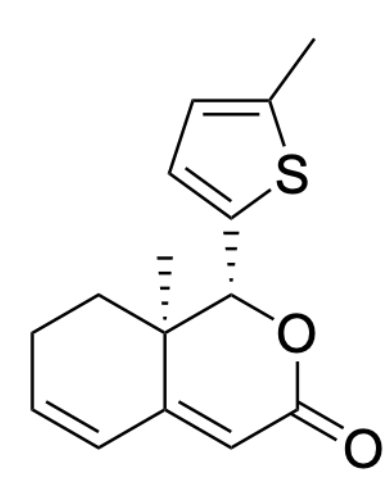

10h

Prepared using general procedure D, using intermediate **9h** (55.7 mg, 0.2 mmol) in THF (2.5 mL), pyridine (0.06 mL, 0.8 mmol), and $SOCl_2$ (0.06 mL, 0.8 mmol) at 0 °C for 20 min. The crude product was washed with 2M HCl, saturated Na_2CO_3, and brine to afford the intermediate **10h** as a dark brown solid without further purification (50 mg, 98 %). ^{1}H NMR (500 MHz, $CDCl_3$) : δ 6.84-6.84 (d, J = 3.5 Hz, 1H), 6.65-6.65 (dd, J = 3.4 Hz, 1.0 Hz, 1H), 6.34-6.30 (dt, J = 9.2 Hz, 2.9 Hz, 1H), 6.24-6.22 (d, J = 10.1 Hz, 1H), 5.69 (s, 1H), 5.29 (s, 1H), 2.47 (s, 3H), 2.33-2.26 (m, 2H), 1.58-1.55 (d, J = 12.7 Hz, 1H), 1.50-1.43 (m, 1H), 1.13 (s, 3H). ^{13}C{^{1}H} NMR (125.82 MHz, $CDCl_3$): δ = 165.6, 158.9, 140.5, 139.0, 134.3, 127.3, 125.3, 124.4, 112.5, 83.7, 37.5, 30.0, 22.6, 16.1, 15.5. HRMS (ESI-TOF) m/z: $[M+H]^+$calcd for $C_{15}H_{17}O_2S$ 261.0944; found 261.0943.

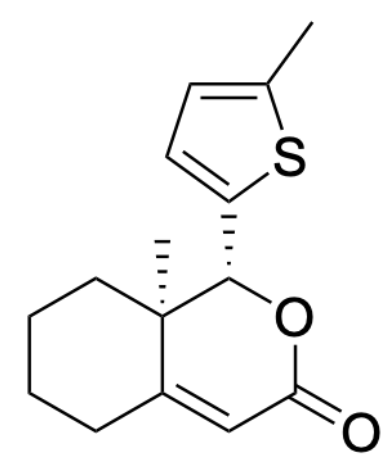

15h

A round-bottom flask containing a magnetic stir bar is charged with **10h (**60.7 mg, 0.23 mmol) and Pd/C (30 % w/w, 18.21 mg) in an inert atmosphere. Dry methanol (3 mL) was added, and the solution was allowed to stir for 10 min before introducing H_2 (g). After 2 h, the reaction was diluted with DCM and filtered using Celite to remove Pd/C from the crude product. The crude product was purified by column chromatography on silica gel using hexanes/ethyl acetate (70:30) as the eluent to afford intermediate **15h** as a white solid (23.2 mg, 38 %). ^{1}H NMR (500.30 MHz, $CDCl_3$): δ 6.84 (d, J = 3.5 Hz, 1H), 6.65-6.64 (dd, J = 3.5 Hz, 1.1 Hz, 1H), 5.76 (s, 1H), 5.23 (s, 1H), 2.47 (s, 3H), 2.46 – 2.41 (m, 1H), 2.40-2.32 (m, 1H), 1.93-1.89 (m, 1H), 1.75-1.70 (d, J = 13.5 Hz, 1H), 1.60 (d, J = 13.5 Hz, 1H), 1.54-1.47 (m, 1H), 1.35-1.28 (m, 2H), 1.14 (s, 3H). ^{13}C{^{1}H} NMR (125.82 MHz, $CDCl_3$): δ = 167.8, 165.0, 140.5, 134.6, 127.3, 124.6, 114.5, 84.7, 39.7, 35.6, 31.2, 25.8, 21.2, 17.4, 15.4. HRMS (ESI-TOF) m/z: $[M+H]^+$ calcd for $C_{15}H_{19}O_2S$ 263.1100; found 263.1100.

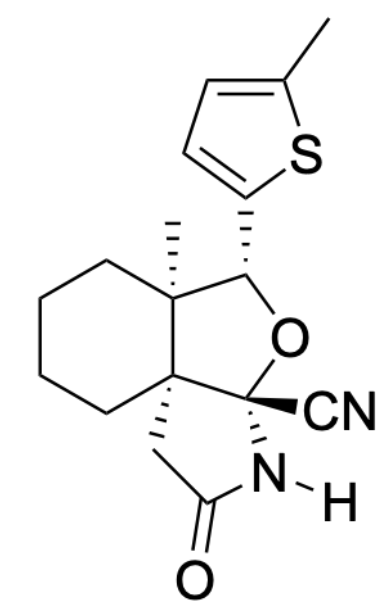

16h

Prepared using general procedure F, using intermediate **15h (**23.3 mg, 0.09 mmol) in DMF/H_2O (2.7:0.3 ratio, 3 mL)**,** KCN (6.0 eq, 35.2 mg, 0.54 mmol), and NH_4Cl (6.0 eq, 29 mg, 0.54 mmol) at 75 ^oC for 5 days. The resulting crude product was purified by column chromatography on silica gel using hexanes/ethyl acetate (60:40) as the eluent to afford **16h and 17h** as white solids. For **16h** (9.1 mg, 33 %) , ^1H NMR (600.21 MHz, $CDCl_3$) : δ 6.72 (d, J = 4.5 Hz, 1H), 6.65 (d, J = 4.1 Hz, 1H), 6.60 (s, 1H), 5.54 (s, 1H), 2.77-2.74 (d, J = 17.6 Hz, 1H), 2.45 (s, 3H), 2.25-2.22 (d, J = 17.7 Hz, 1H), 2.16-2.11 (m, 1H), 2.00-1.93 (m, 2H), 1.75-1.72 (m, 1H), 1.67-1.64 (m, 1H), 1.56-1.49 (m, 1H), 1.37-1.31 (m, 2H), 0.87 (s, 3H). $^{13}C\{^1H\}$ NMR (150.94 MHz, $CDCl_3$): δ =173.9, 140.0, 135.3, 124.8, 116.3, 91.6, 85.8, 55.4, 45.9, 39.7, 34.1, 31.9, 22.6, 21.2, 20.3, 15.3. HRMS (ESI-TOF) m/z: $[M+H]^+$ calcd for $C_{17}H_{21}N_2O_2S$ 317.1318; found = 317.1318.

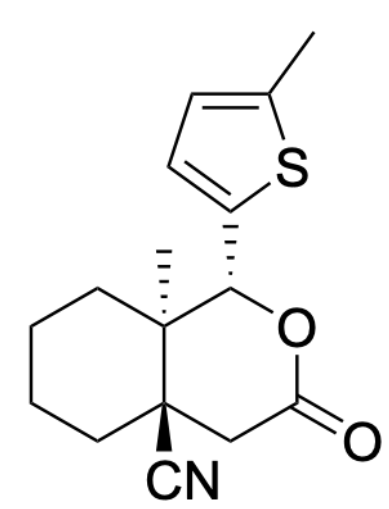

17h

For **17h** (7.7 mg, 28 %) , ^1H NMR (500 MHz, $CDCl_3$) : δ 6.83-6.83 (d, J = 3.3 Hz, 1H), 6.66-6.65 (d, J = 3.1 Hz, 1H), 5.81 (s, 1H), 2.98-2.95 (d, J = 18.5 Hz, 1H), 2.62-2.58 (d, J = 18.6 Hz, 1H), 2.47 (s, 3H), 1.90–1.87 (m, 1H), 1.82-1.81 (m, IH), 1.79-1.77 (m, 1H), 1.74-1.70 (m, 1H), 1.67-1.58 (m, 2H), 1.47–1.41 (m, 2H), 1.08 (s, 3H). $^{13}C\{^1H\}$ NMR (125.82 MHz, $CDCl_3$): δ = 166.0, 141.0, 133.8, 127.6, 124.5, 121.5, 85.5, 42.2, 38.23, 38.17, 31.5, 31.0, 22.0, 19.8, 15.2, 11.8. HRMS (ESI-TOF) m/z: $[M+H]^+$ calcd for $C_{16}H_{20}NO_2S$ 290.1209; found 290.1210.

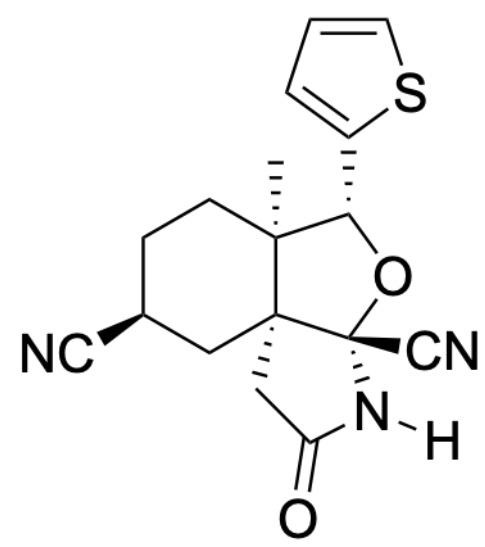

18a

A round-bottom flask containing a magnetic stir bar is charged with KCN (35.1 mg, 0.54 mmol), NH_4Cl (28.9 mg, 0.54 mmol), and the starting material **12a** (23.8 mg, 0.09 mmol) in DMF/H_2O (4:0.5 ratio, 4.5 mL). The solution was heated to 75 °C, for 5 days. At reaction completion, the reaction mixture was cooled to rt, then ice was added to the solution and allowed to stir for an additional 15 min before extracting the solution with DCM (3 times). After extraction, the combined organic layers were washed with H_2O and brine, dried over sodium sulfate, and concentrated under reduced pressure to obtain the crude mixtures. The resulting crude mixtures was purified by column chromatography using silica gel and hexanes/ethyl acetate (80:20) as the eluent to afford the pure products **13a** (6.3 mg, 22 %) and traces of diastereomer **18a** (1 mg, 4%). **For 18a:** ^{1}H NMR (500.30 MHz, $CDCl_3$): δ 7.35-7.34 (d, J = 5.1 Hz, 1H), 7.05-7.04 (d, J = 8.7 Hz, 1H), 6.96-6.95 (dd, J = 5.1 Hz, 8.3 Hz, 1H), 6.49 (s, 1H), 5.66 (s, 1H), 2.75-2.71 (d, J = 17.4 Hz, 1H), 2.53-2.49 (tt, J = 14.0 Hz, 5.0 Hz, 1H), 2.49-2.43 (m, 1H), 2.36-2.33 (d, J = 17.4 Hz, 1H), 2.17-2.14 (d, J = 16.9 Hz, 2H), 1.92 – 1.85 (m, 2H), 1.44-1.43 (d, J = 7.7 Hz, 1H), 0.85 (s, 3H). ^{13}C{^{1}H} NMR (125.82 MHz, $CDCl_3$): δ = 172.0, 136.4, 126.9, 125.9, 125.5, 120.3, 115.3, 90.3, 84.7, 54.8, 45.4, 38.8, 36.7, 29.9, 25.9, 25.2, 20.2. HRMS (ESI-TOF) m/z: $[M+H]^+$ calcd for $C_{17}H_{18}N_3O_2S$ 328.1114; found = 328.1116.

NMR Spectra

^{1}H NMR (600.21 MHz) and $^{13}C\{^{1}H\}$ (150.94 MHz) for Compound **6** in CDCl3:

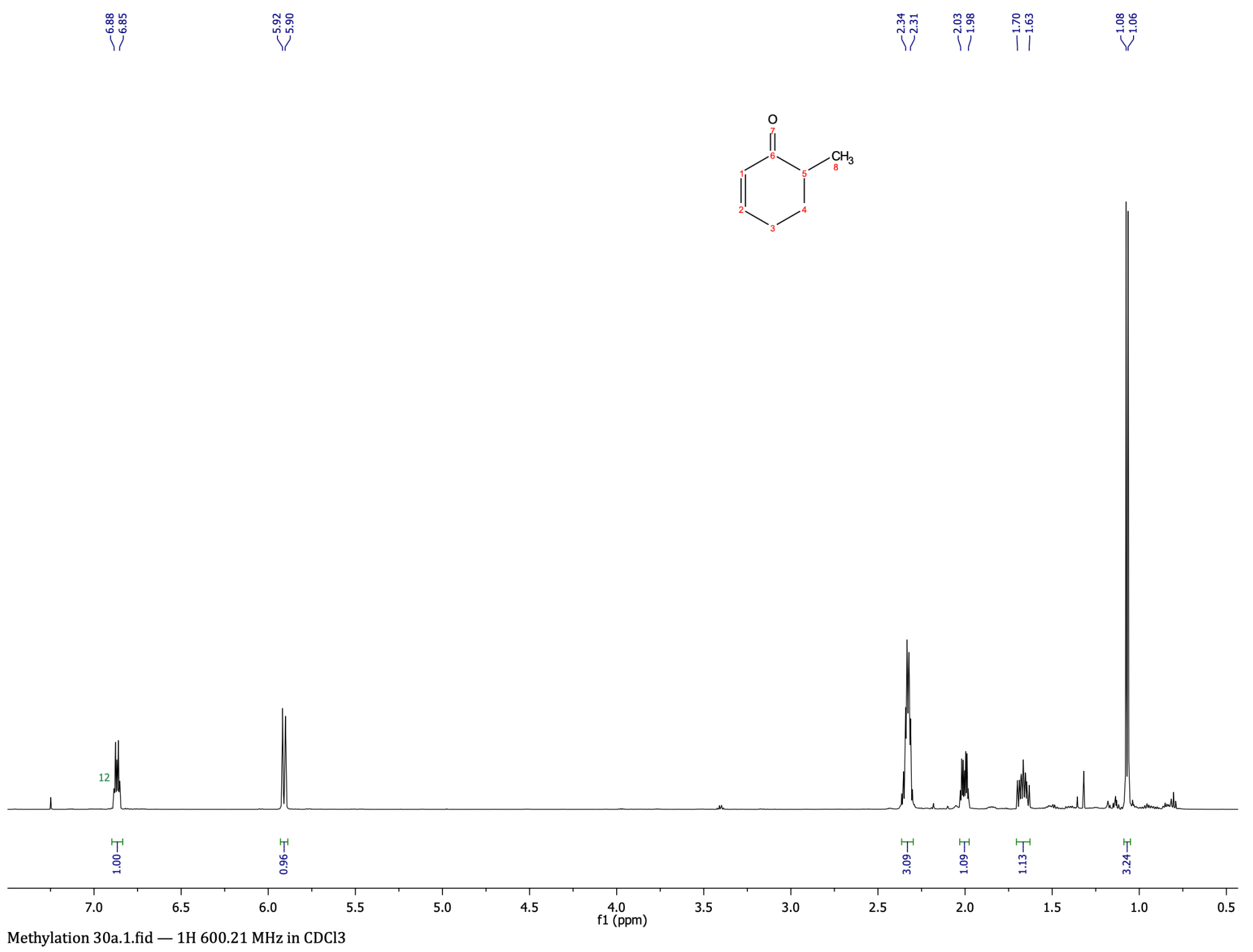

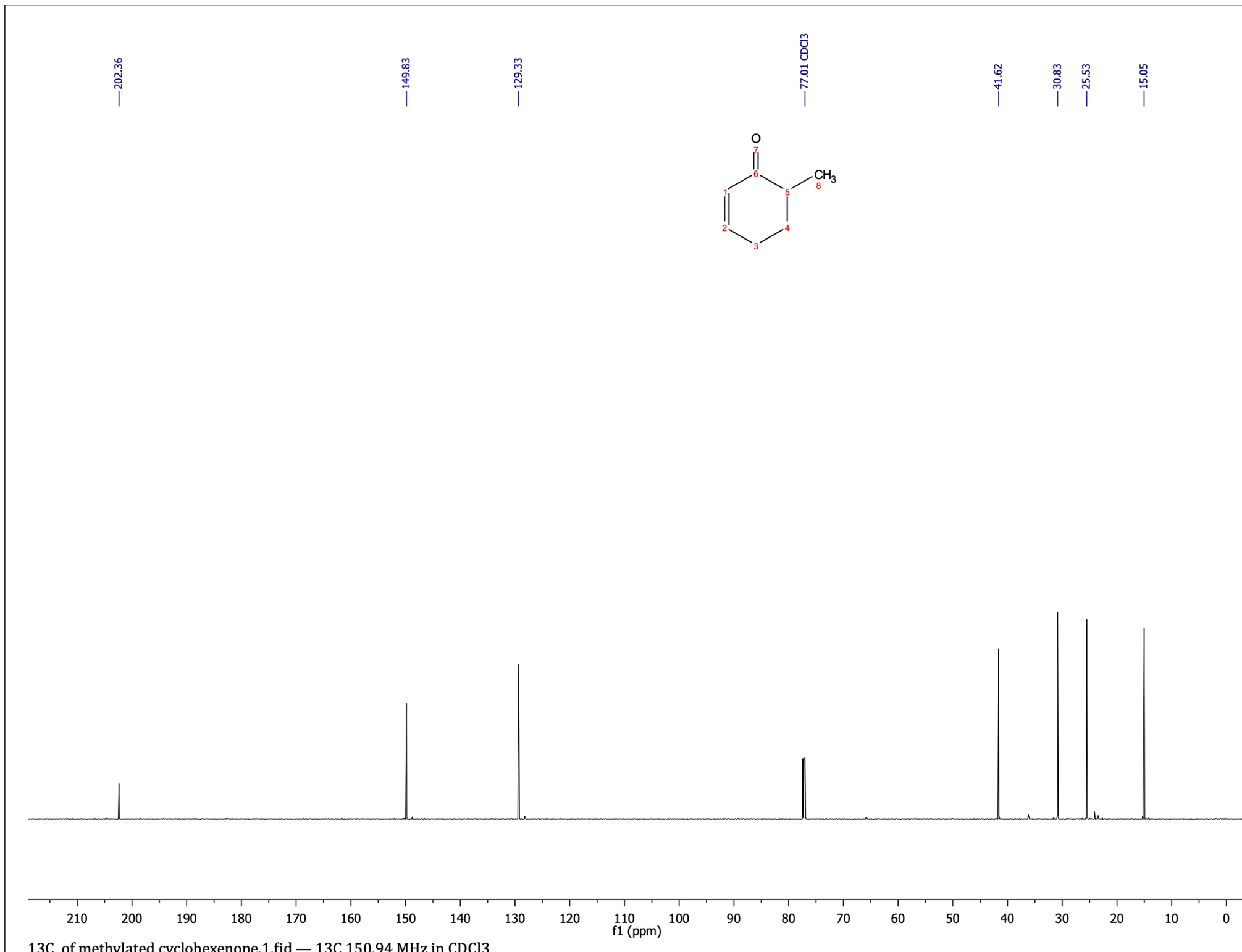

^{1}H NMR (600.21 MHz) and ^{13}C{^{1}H} NMR (150.94 MHz) for Compound **7a** in CDCl3:

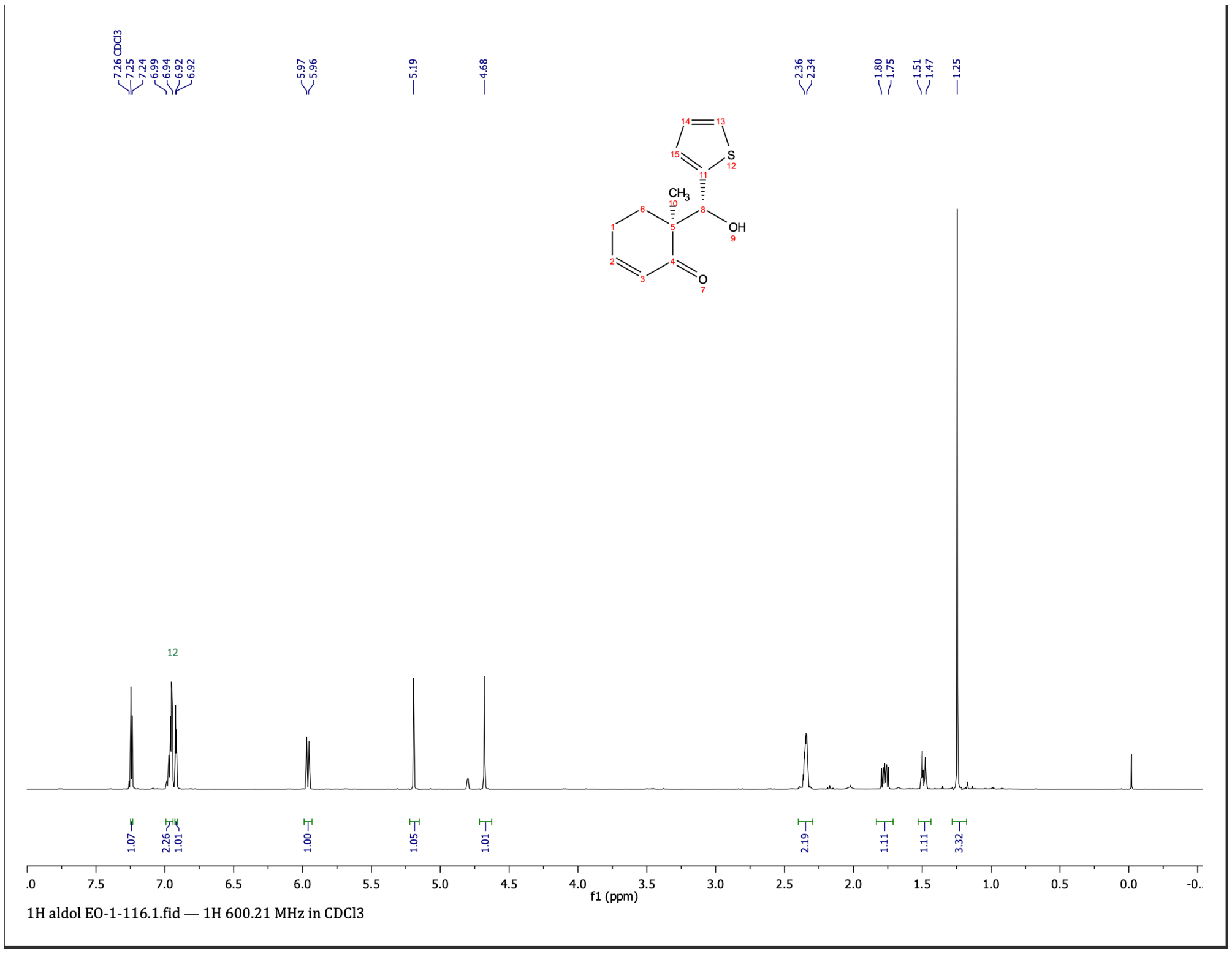

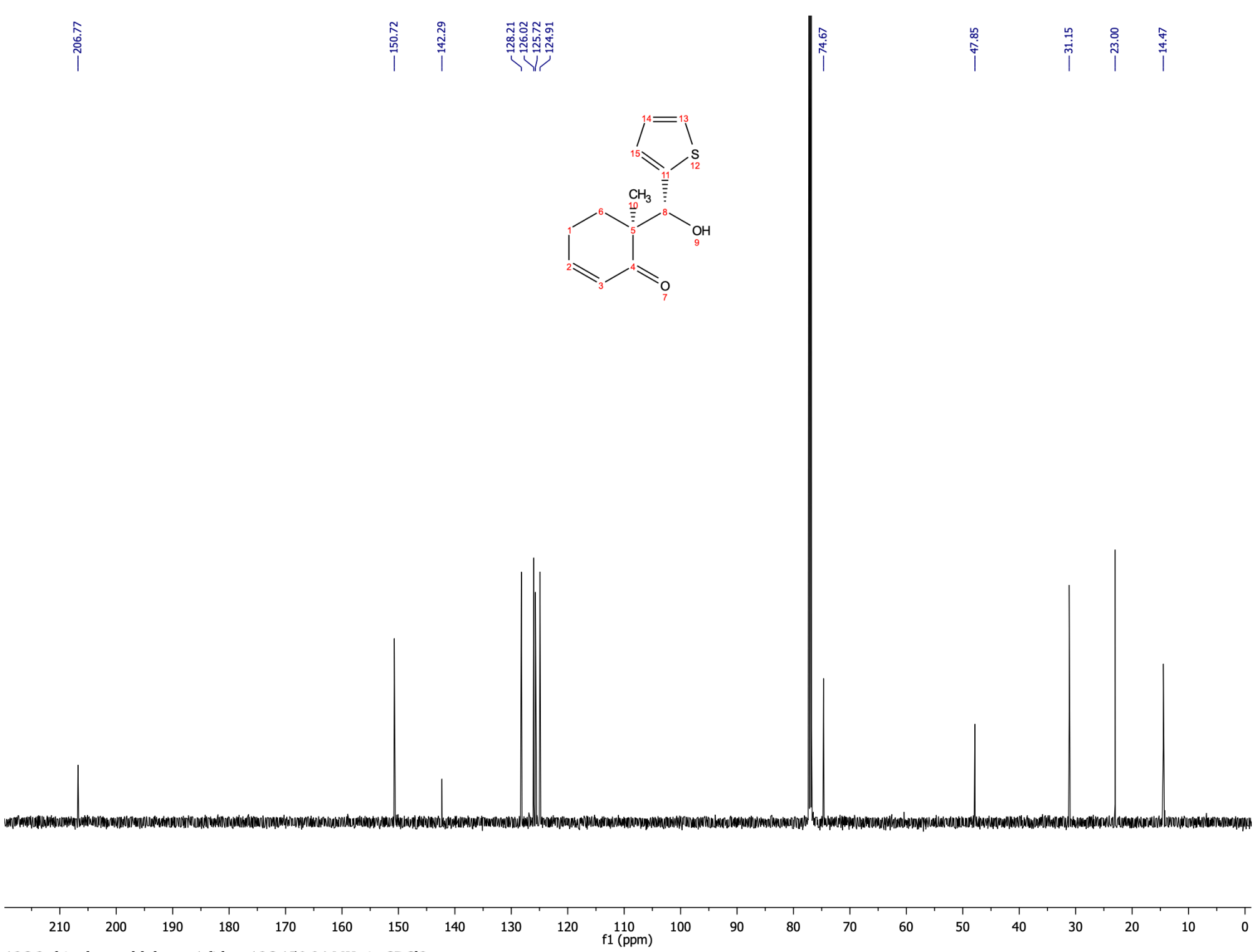

^{1}H NMR (600.21 MHz) and ^{13}C{^{1}H} NMR (150.94 MHz) for Compound **8a** in CDCl3:

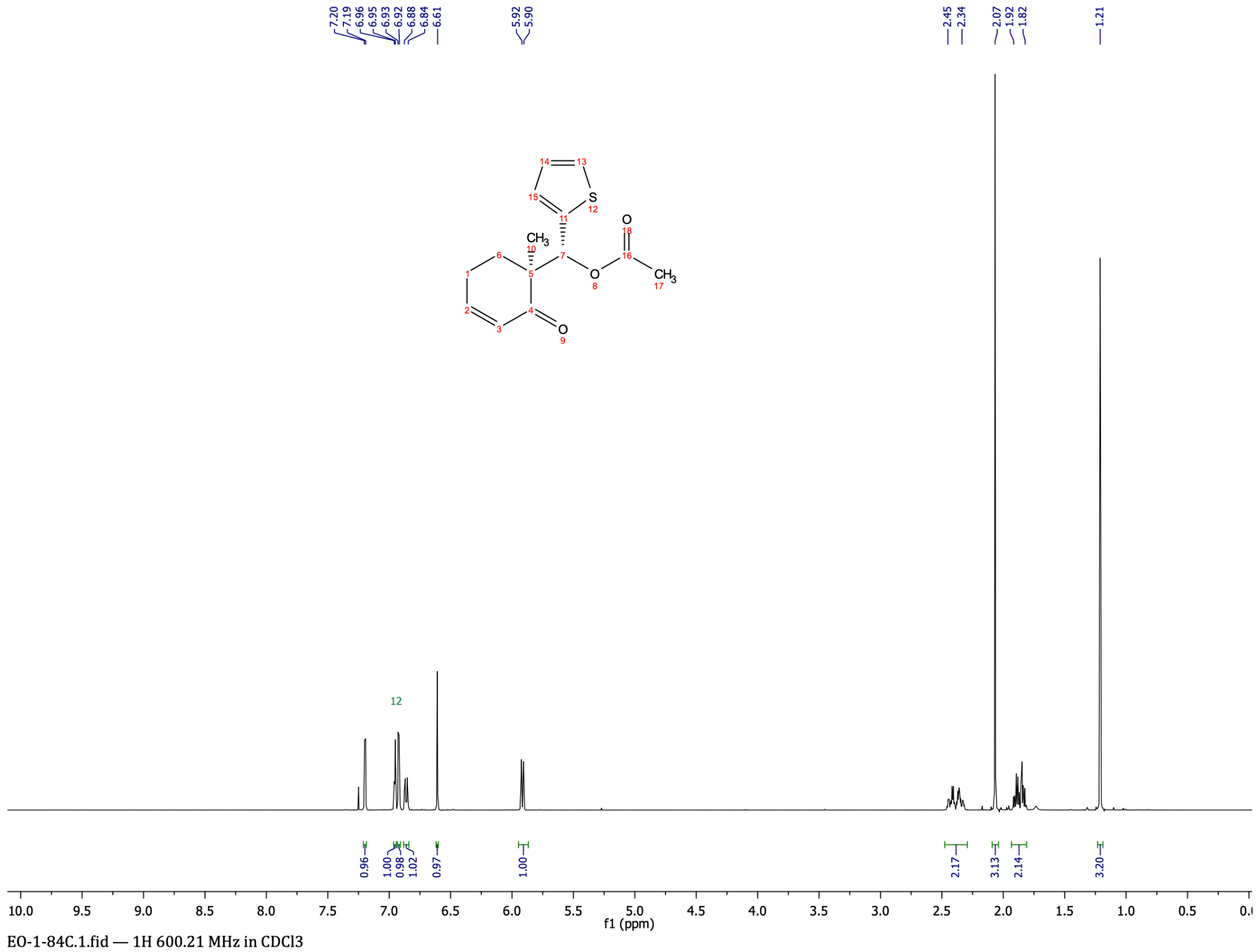

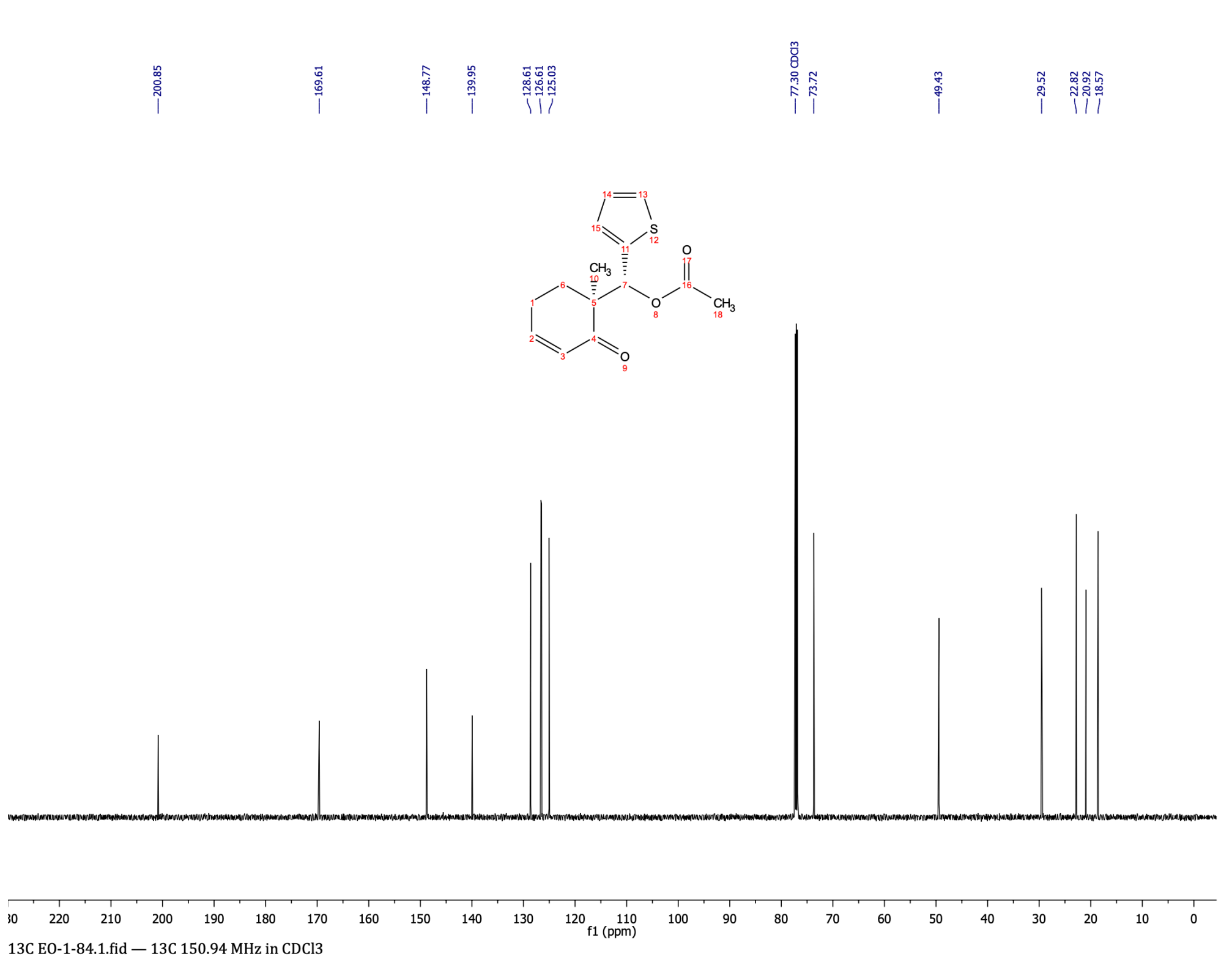

^1H NMR (600.21 MHz) and $^{13}C\{^1H\}$ NMR (150.94 MHz) for Compound **9a** in CDCl3:

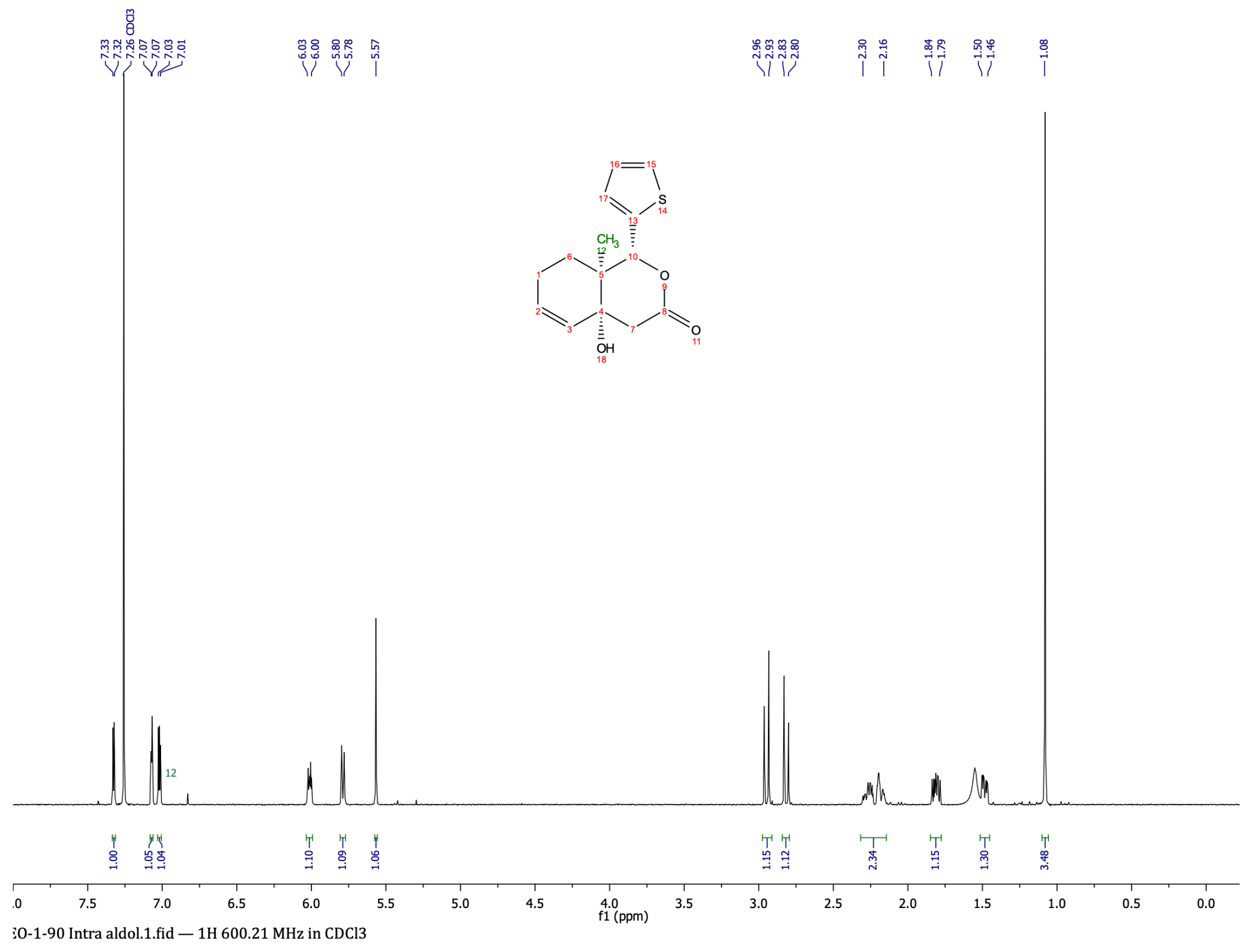

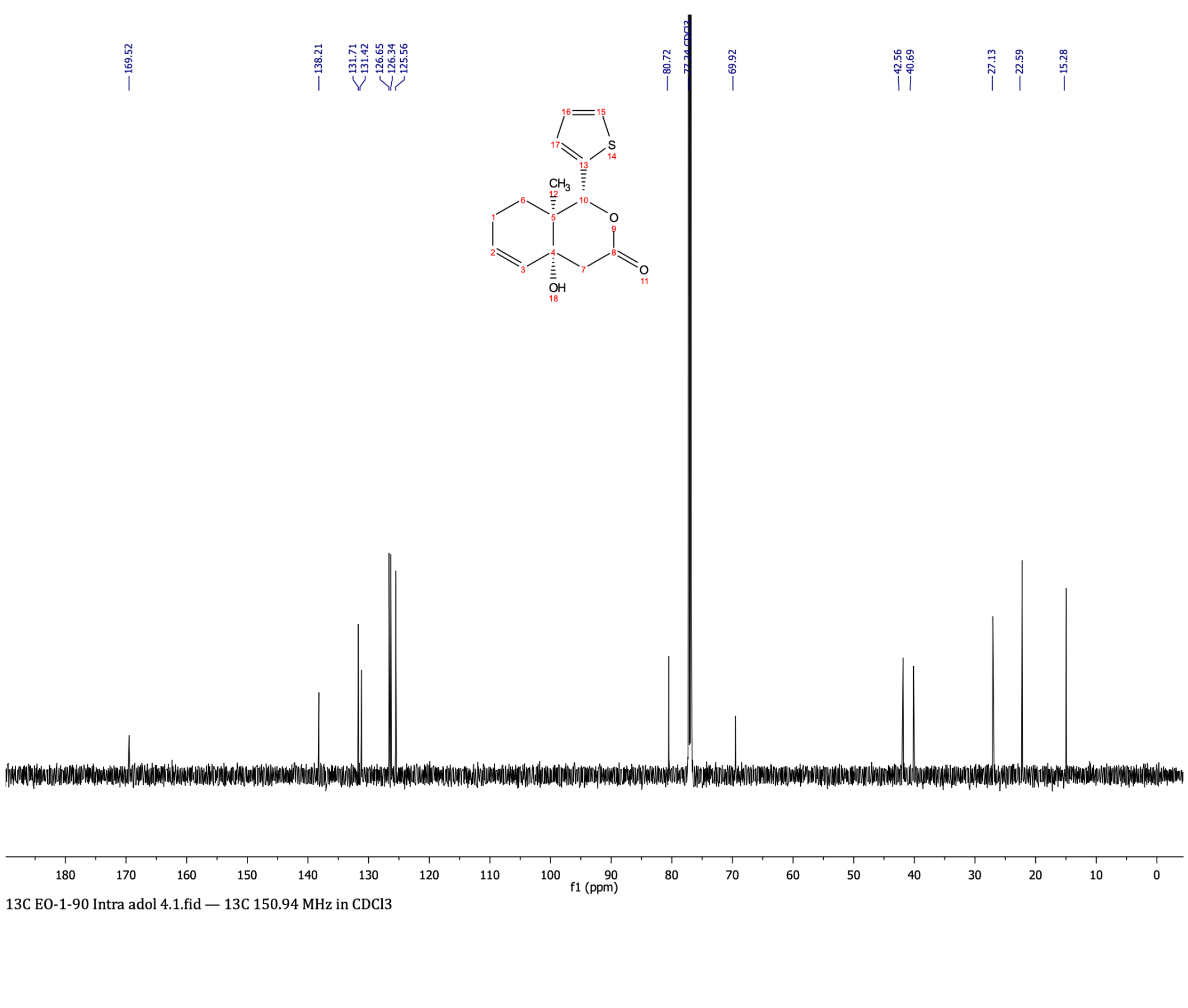

^{1}H NMR (500.30 MHz) and $^{13}C\{^{1}H\}$ NMR (125.82 MHz) for Compound **10a** in CDCl3:

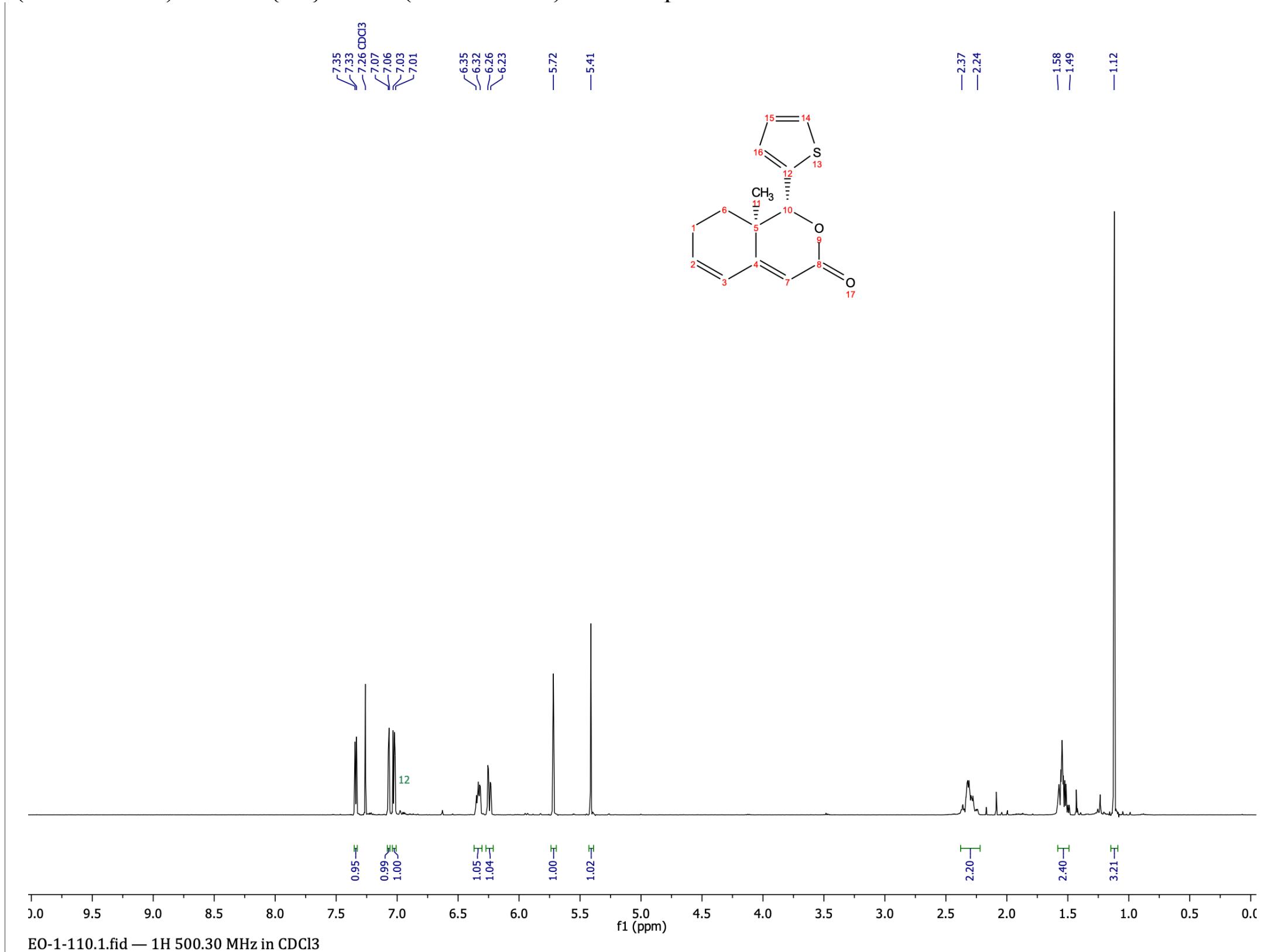

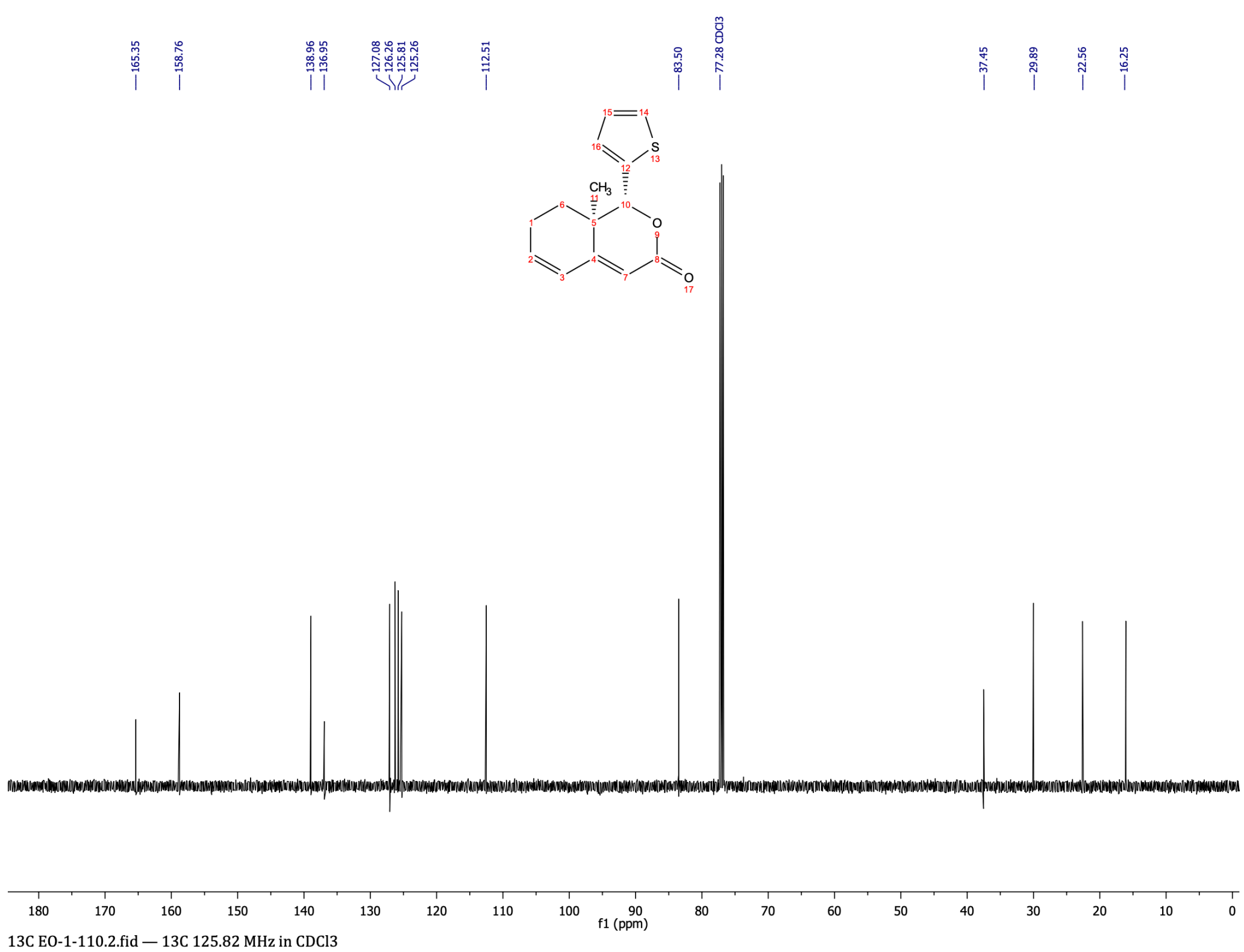

^{1}H NMR (500.30 MHz) and ^{13}C{^{1}H} NMR (125.82 MHz) for Compound **12a** in CDCl3:

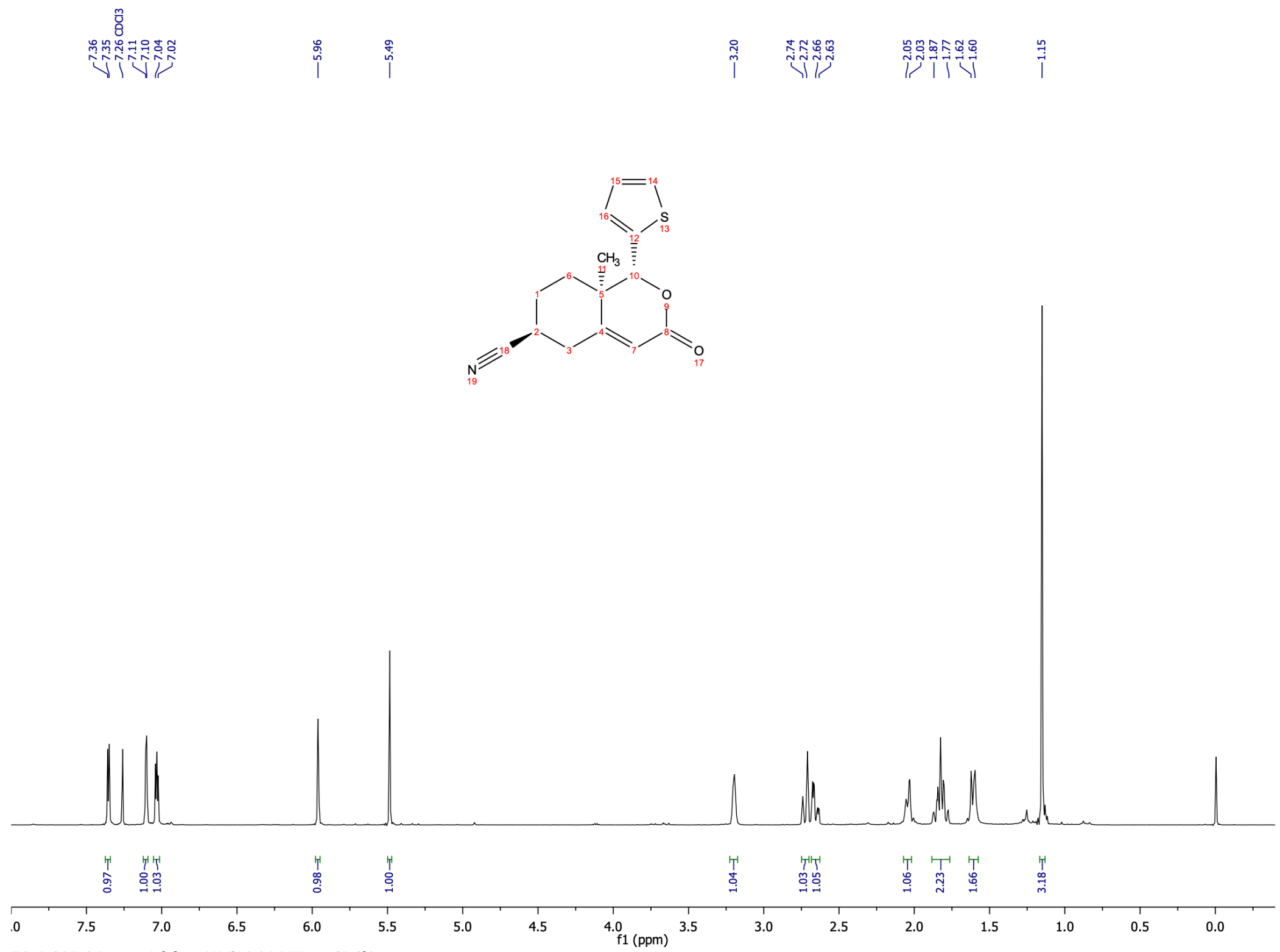

EO-1-287-2 Purest.1.fid — 1H 500.30 MHz in CDCl3

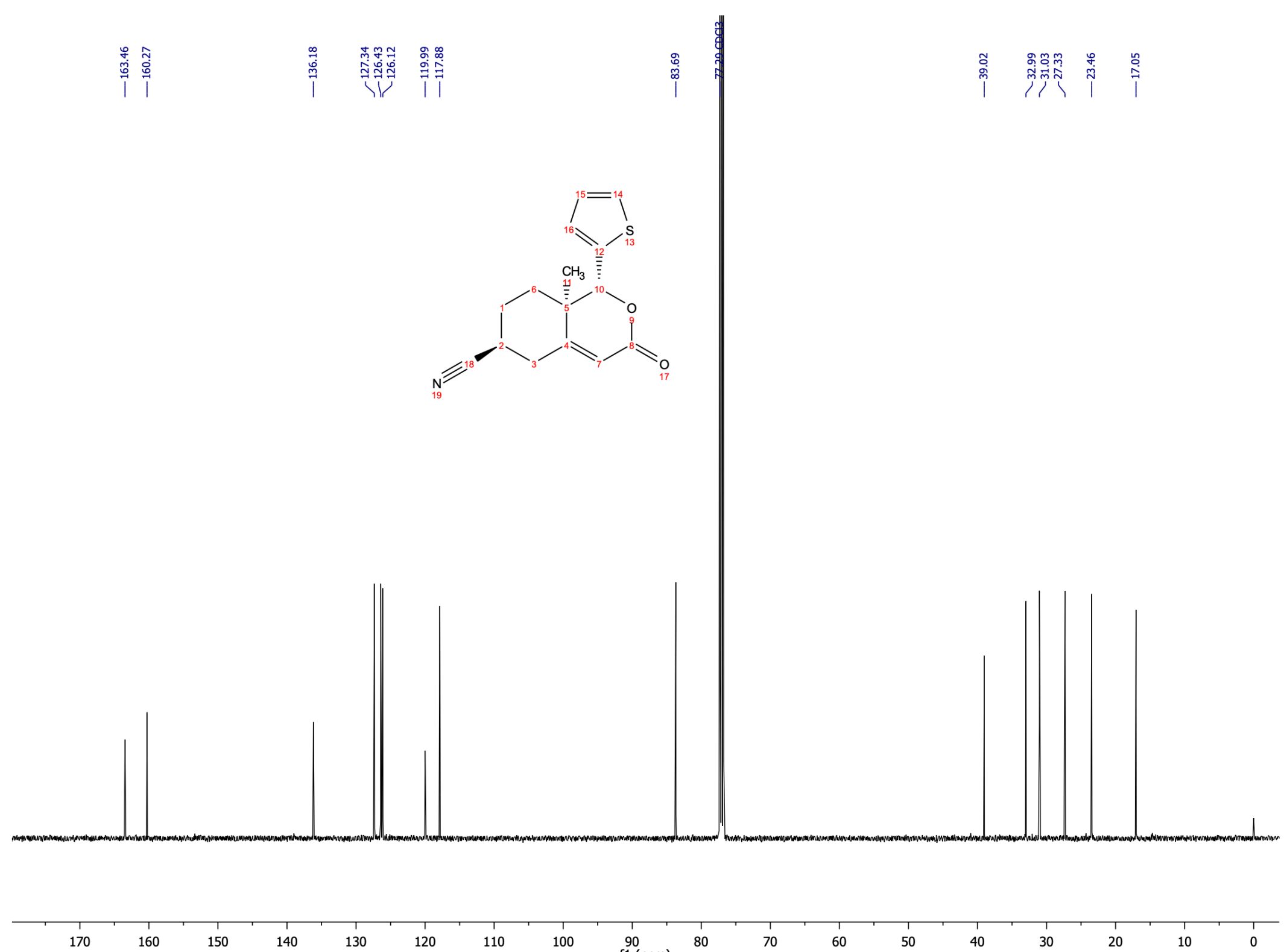

13C EO-1-287 Purest (CN Addition).2.fid — 13C 125.82 MHz in CDCl3

^{1}H NMR (500.30 MHz) and ^{13}C{^{1}H} NMR (125.82 MHz) for Compound **13a** in CDCl3:

Page 82 of 148

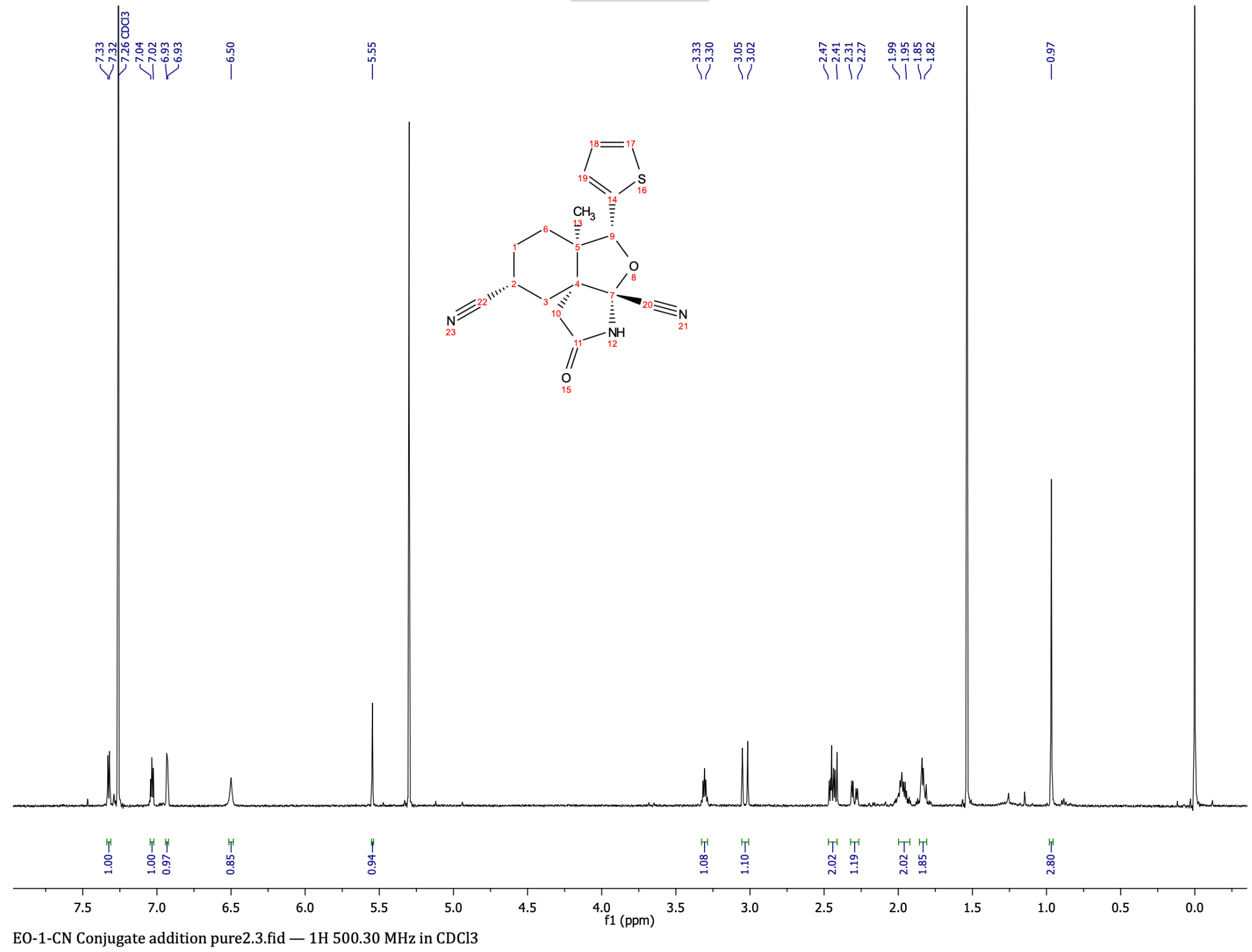

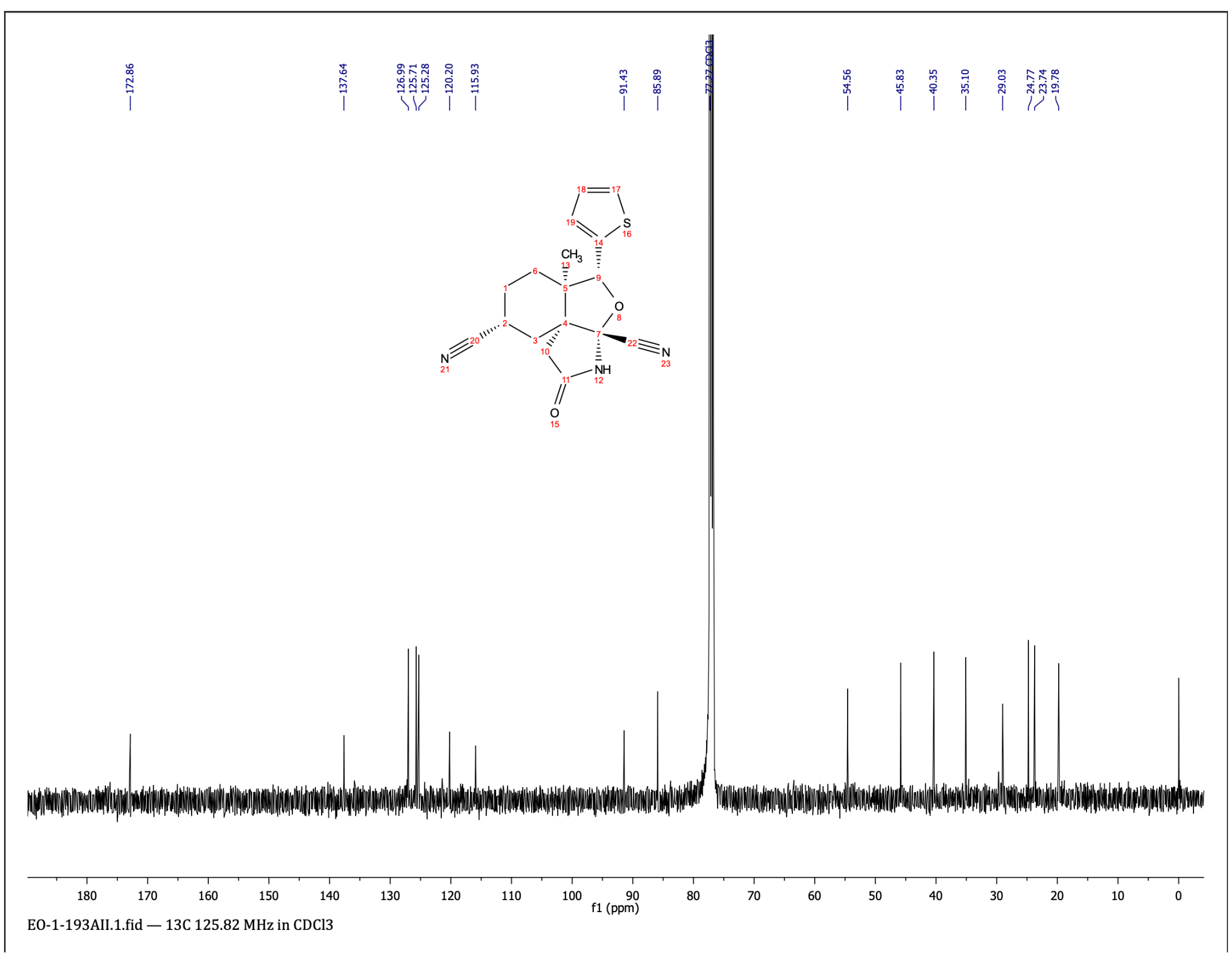

^{1}H NMR (600.21 MHz) and ^{13}C{^{1}H} NMR (150.94 MHz) for Compound **7b** in CDCl3:

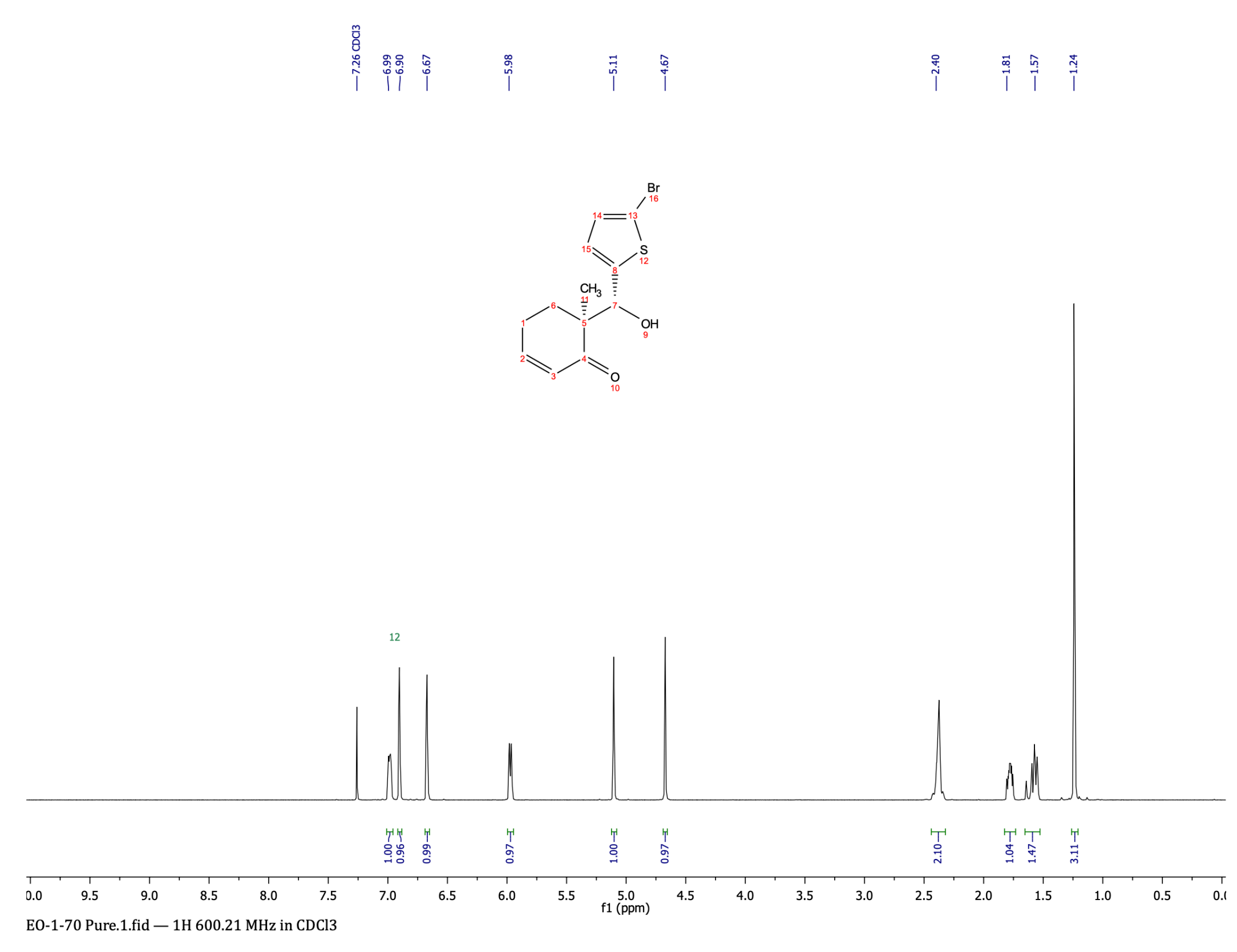

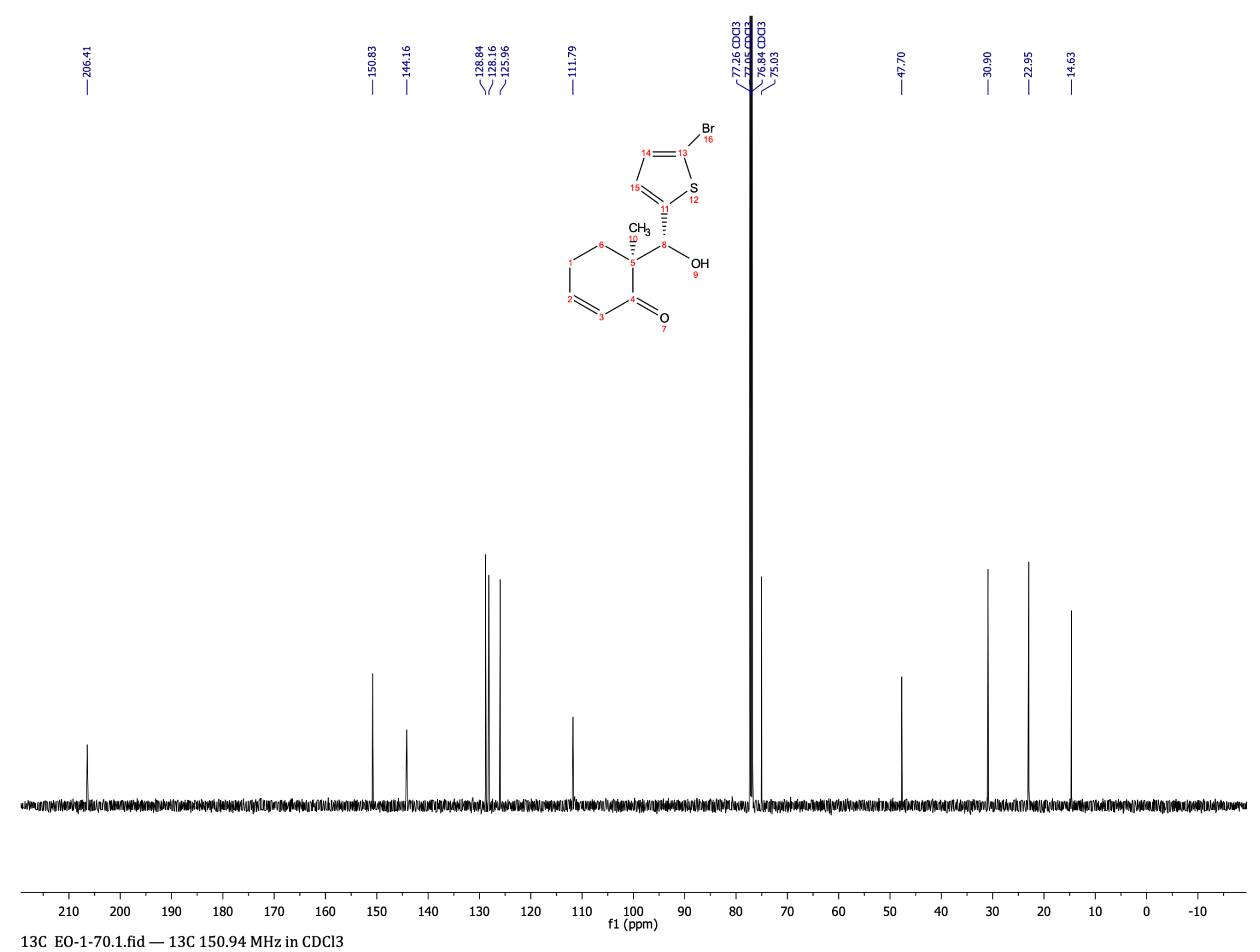

^{1}H NMR (500.30 MHz) and ^{13}C{^{1}H} NMR (125.82 MHz) for Compound **8b** in CDCl3:

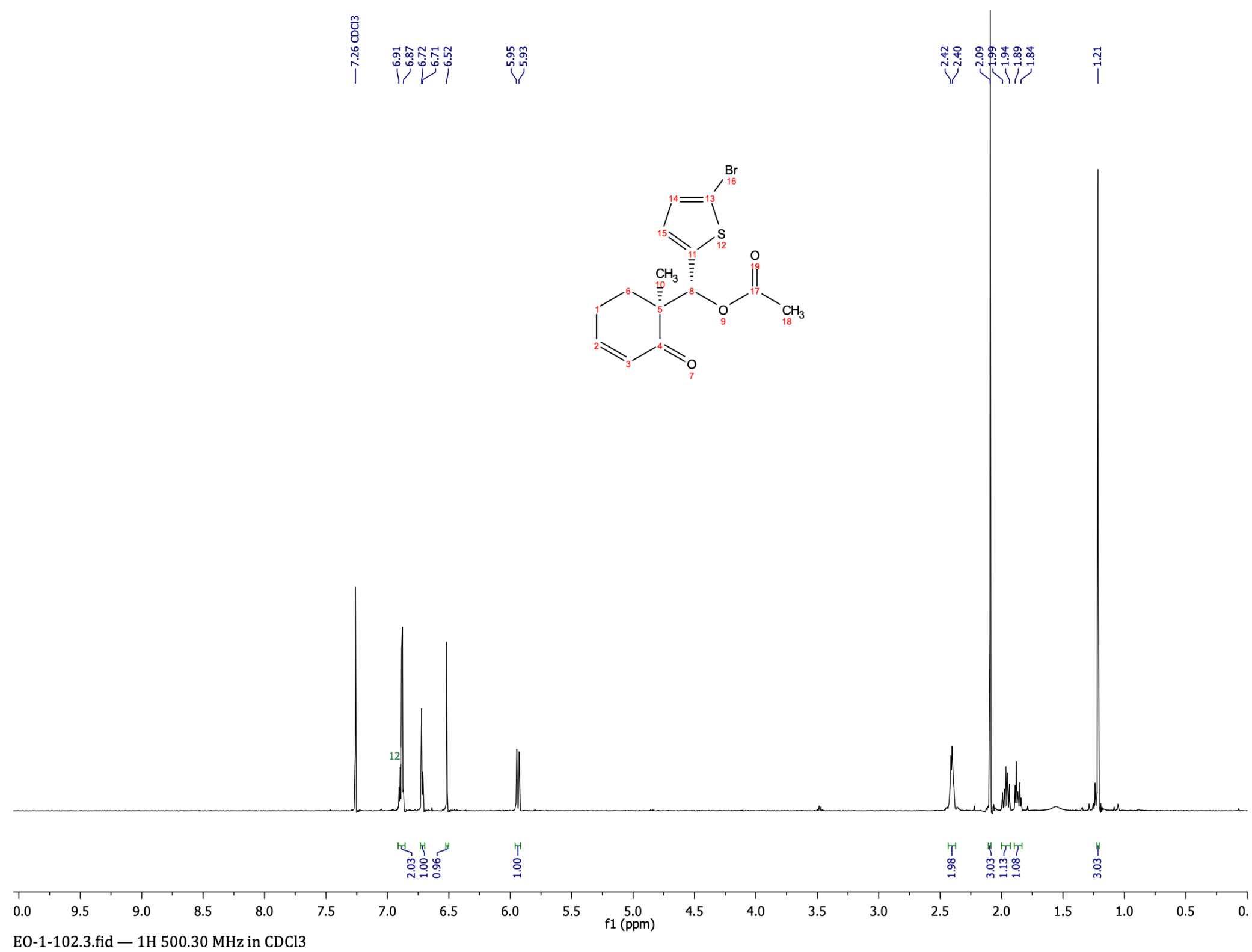

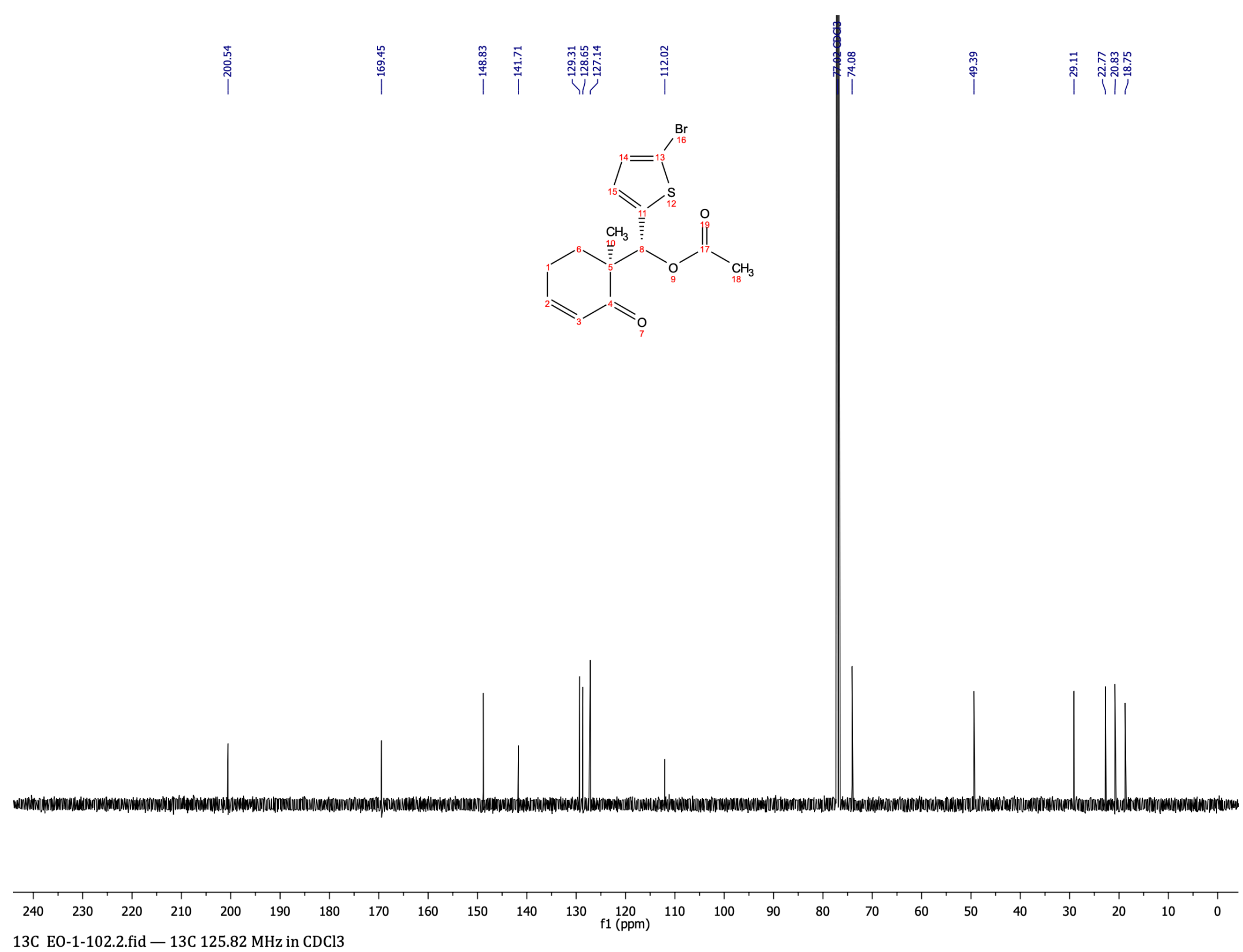

^{1}H NMR (500.30 MHz) and $^{13}C\{^{1}H\}$ NMR (125.82 MHz) for Compound **9b** in CDCl3:

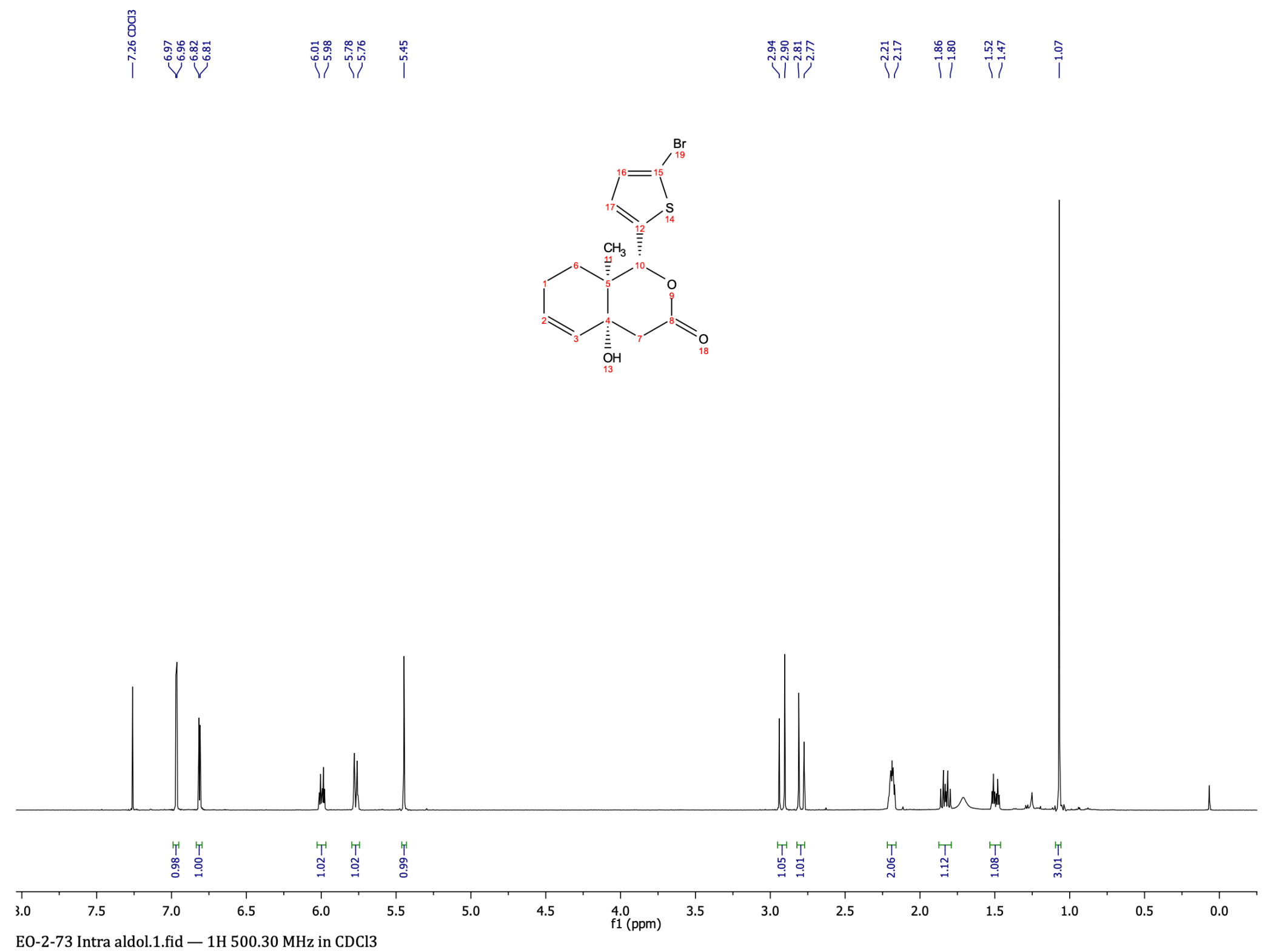

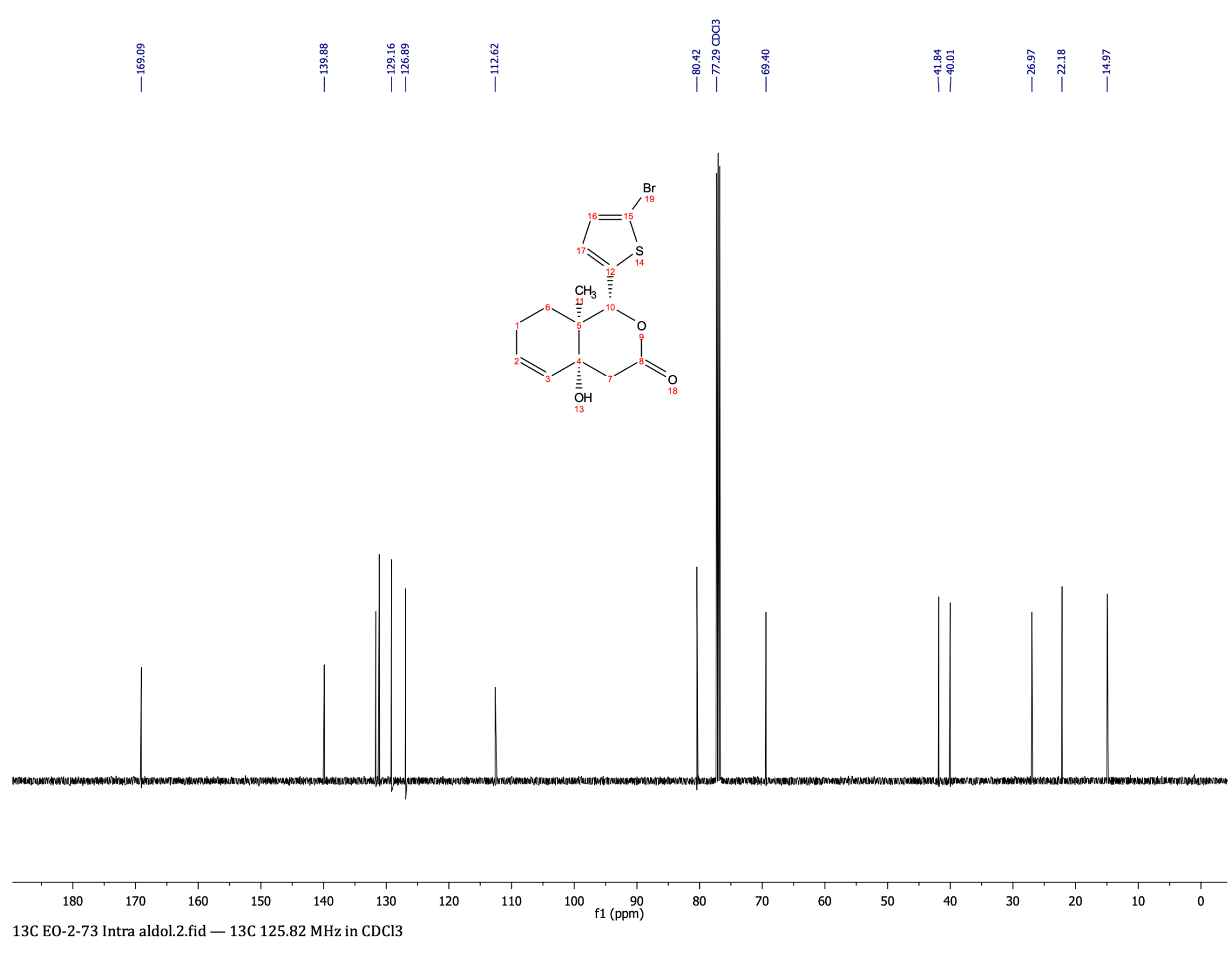

^{1}H NMR (500.30 MHz) and $^{13}C\{^1H\}$ NMR (125.82 MHz) for Compound **10b** in CDCl3:

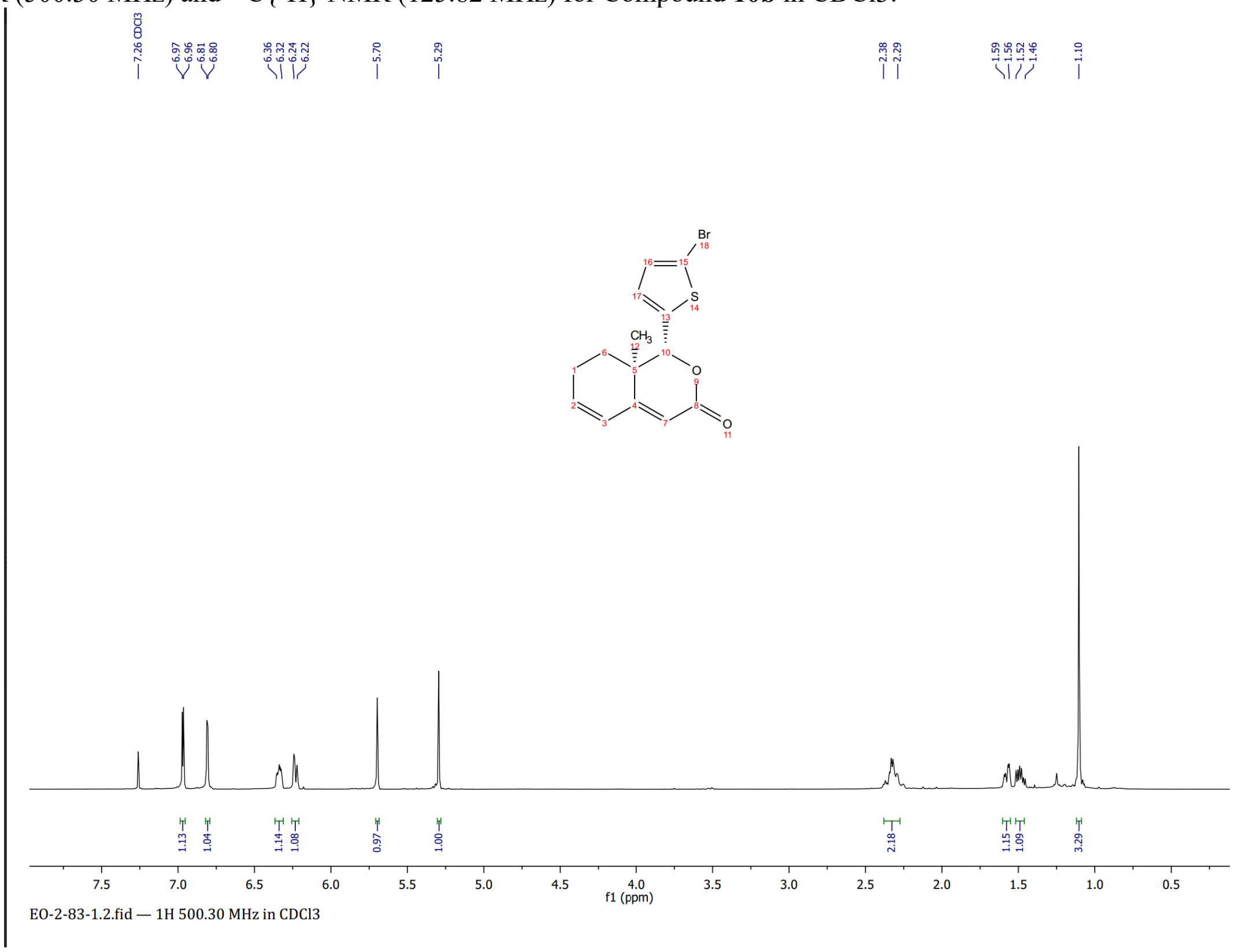

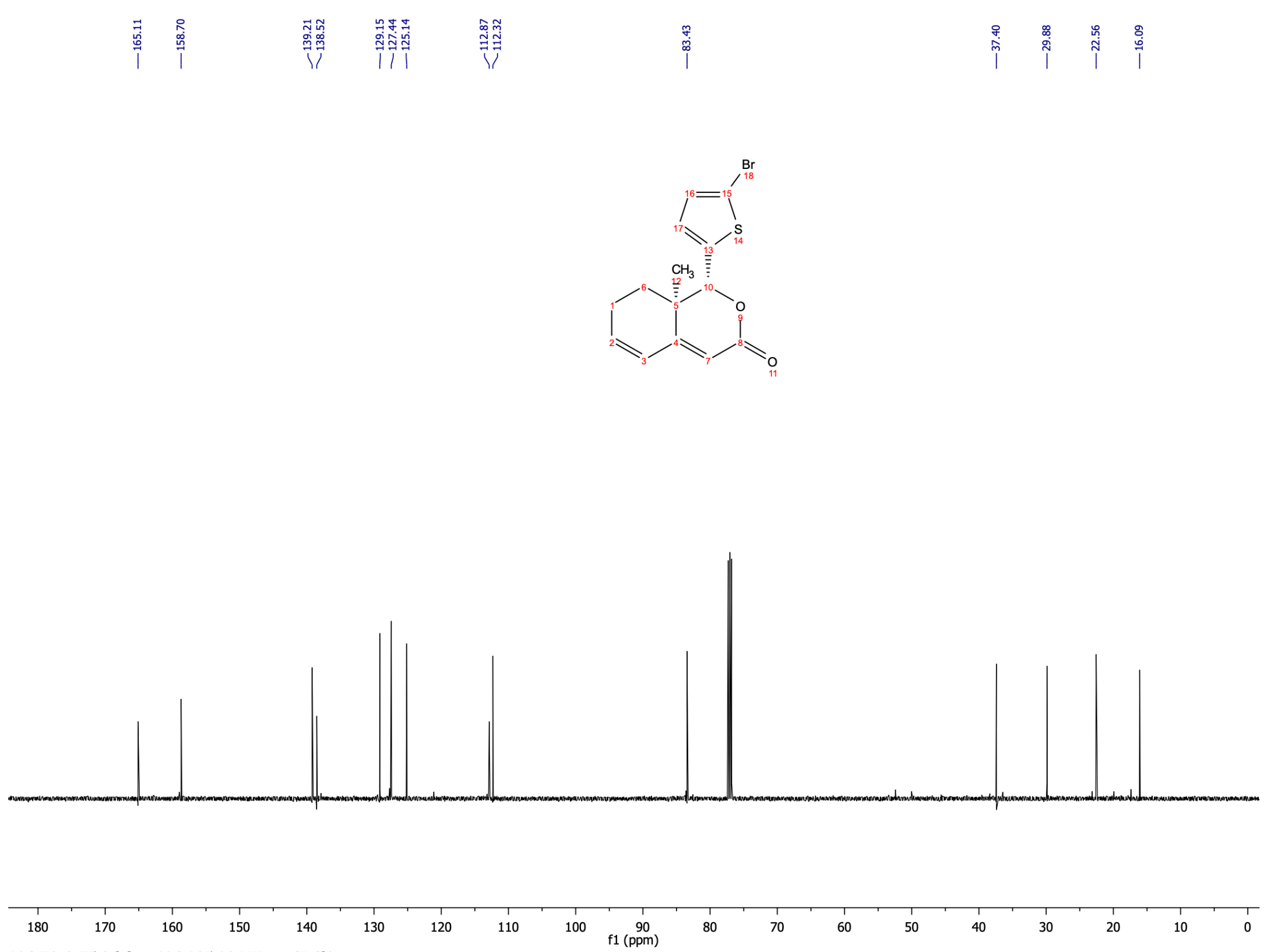

^{1}H NMR (500.30 MHz) and ^{13}C{^{1}H} NMR (125.82 MHz) for Compound **13b** in CDCl3:

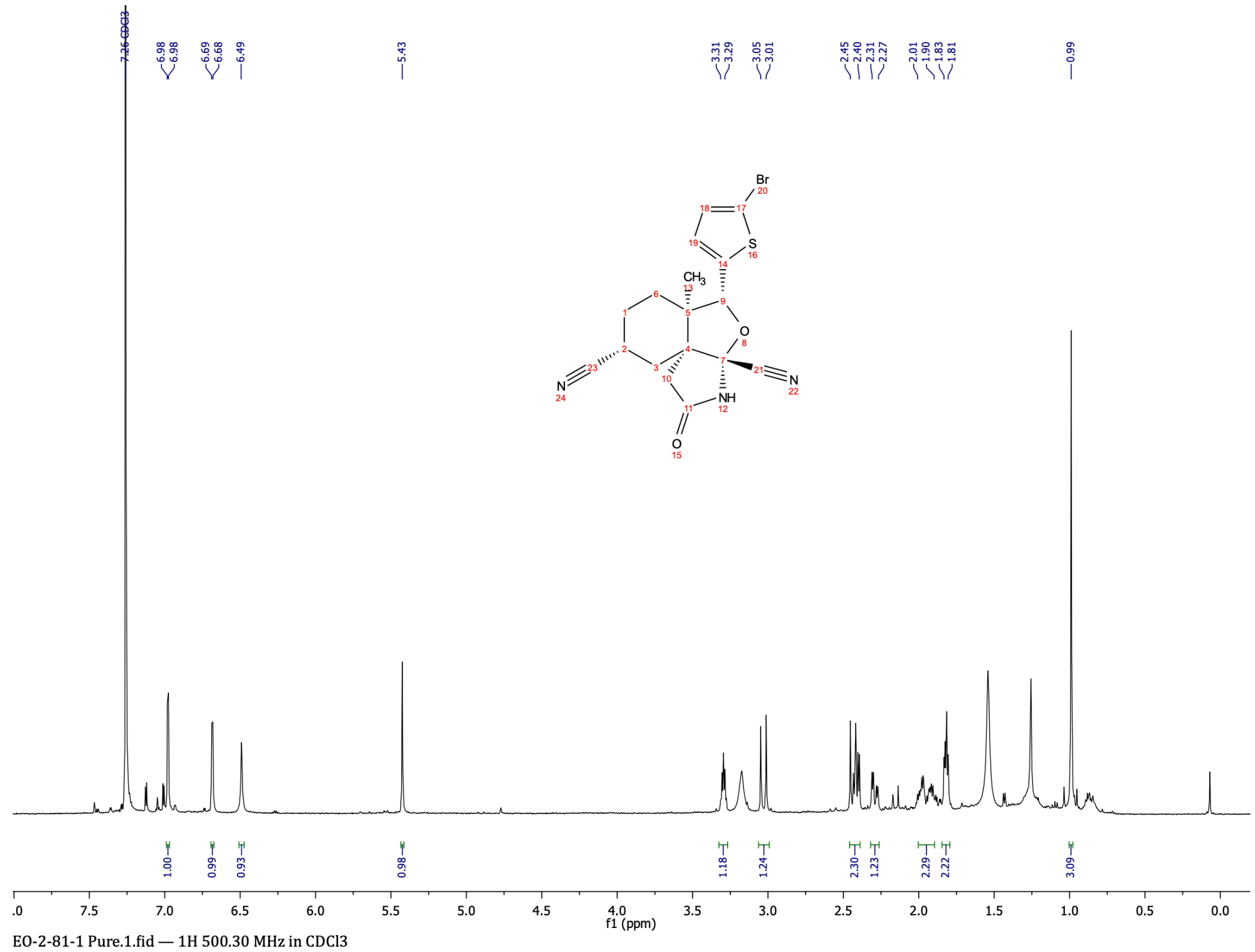

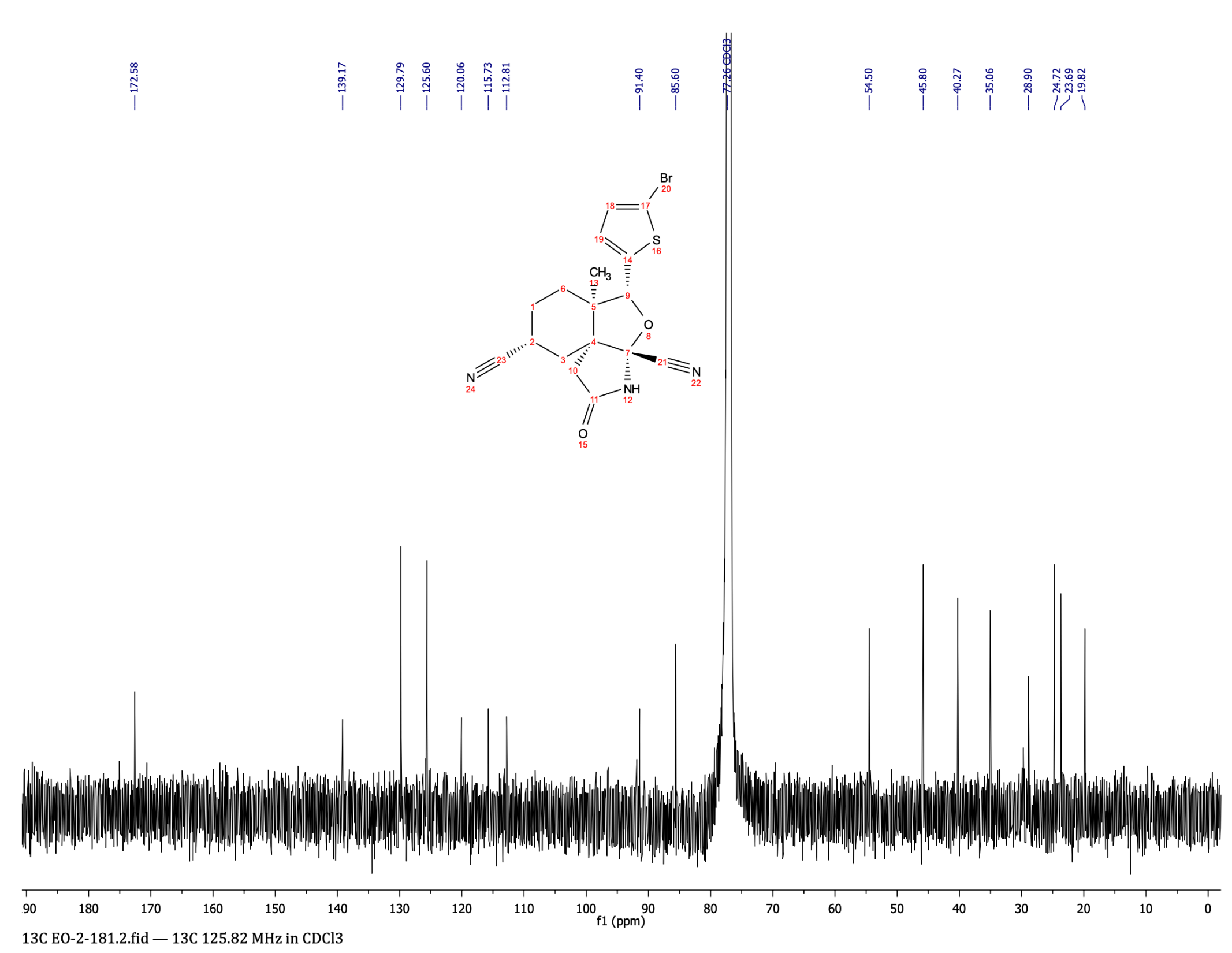

^{1}H NMR (400.30 MHz) and $^{13}C\{^{1}H\}$ NMR (100.67 MHz) for Compound **7c** in CDCl3:

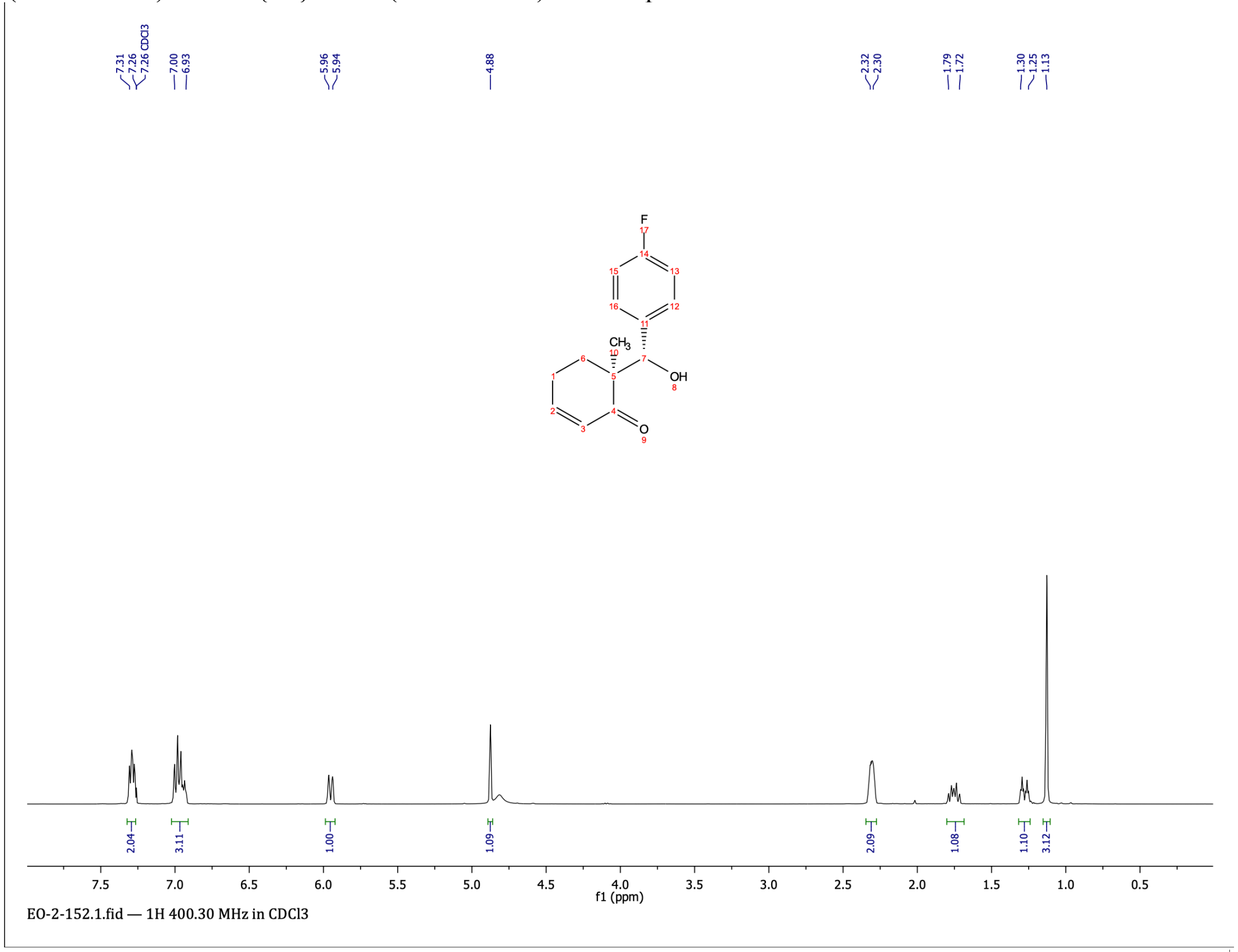

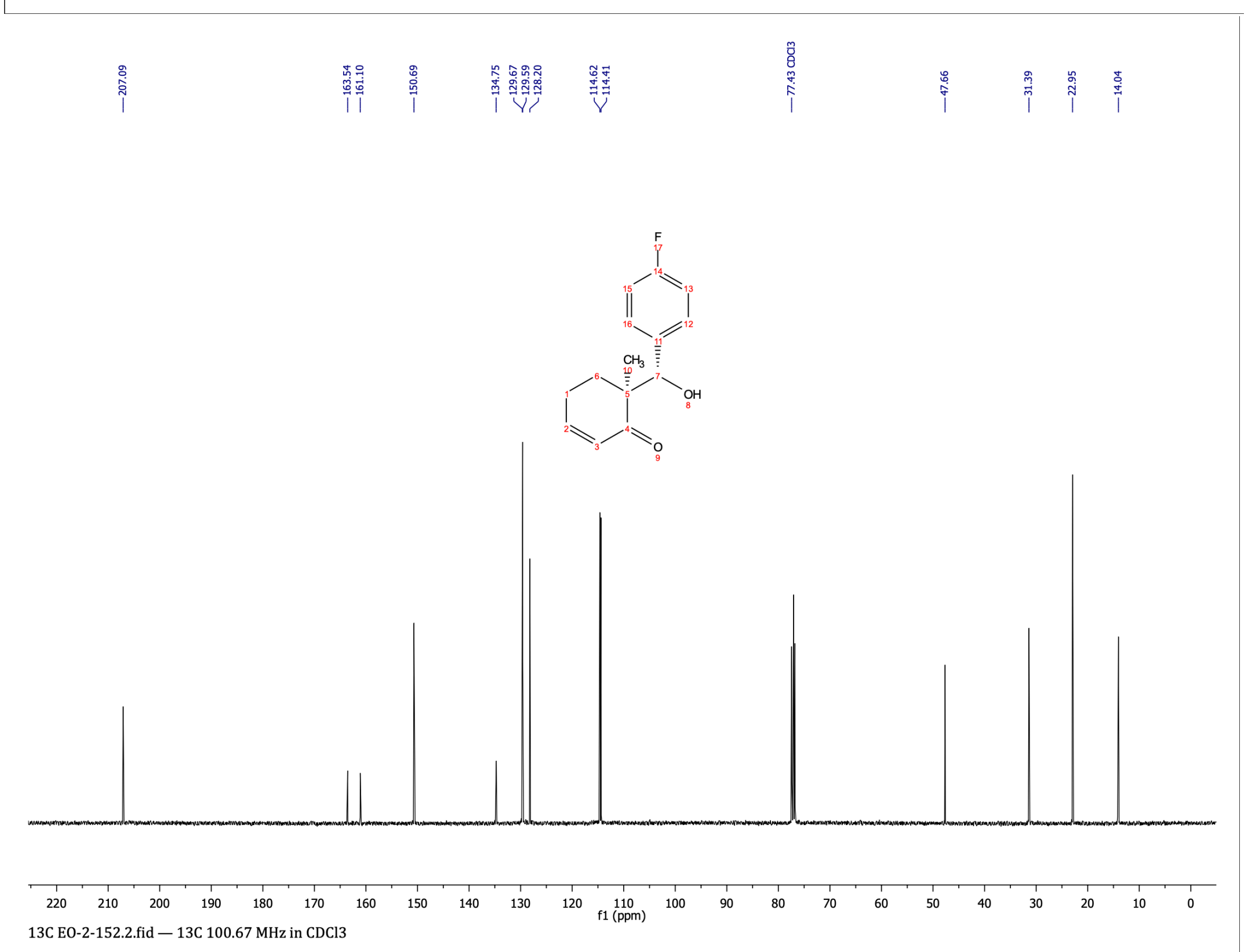

^{1}H NMR (400.30 MHz) and ^{13}C{^{1}H} NMR (100.67 MHz) for Compound **8c** in CDCl3:

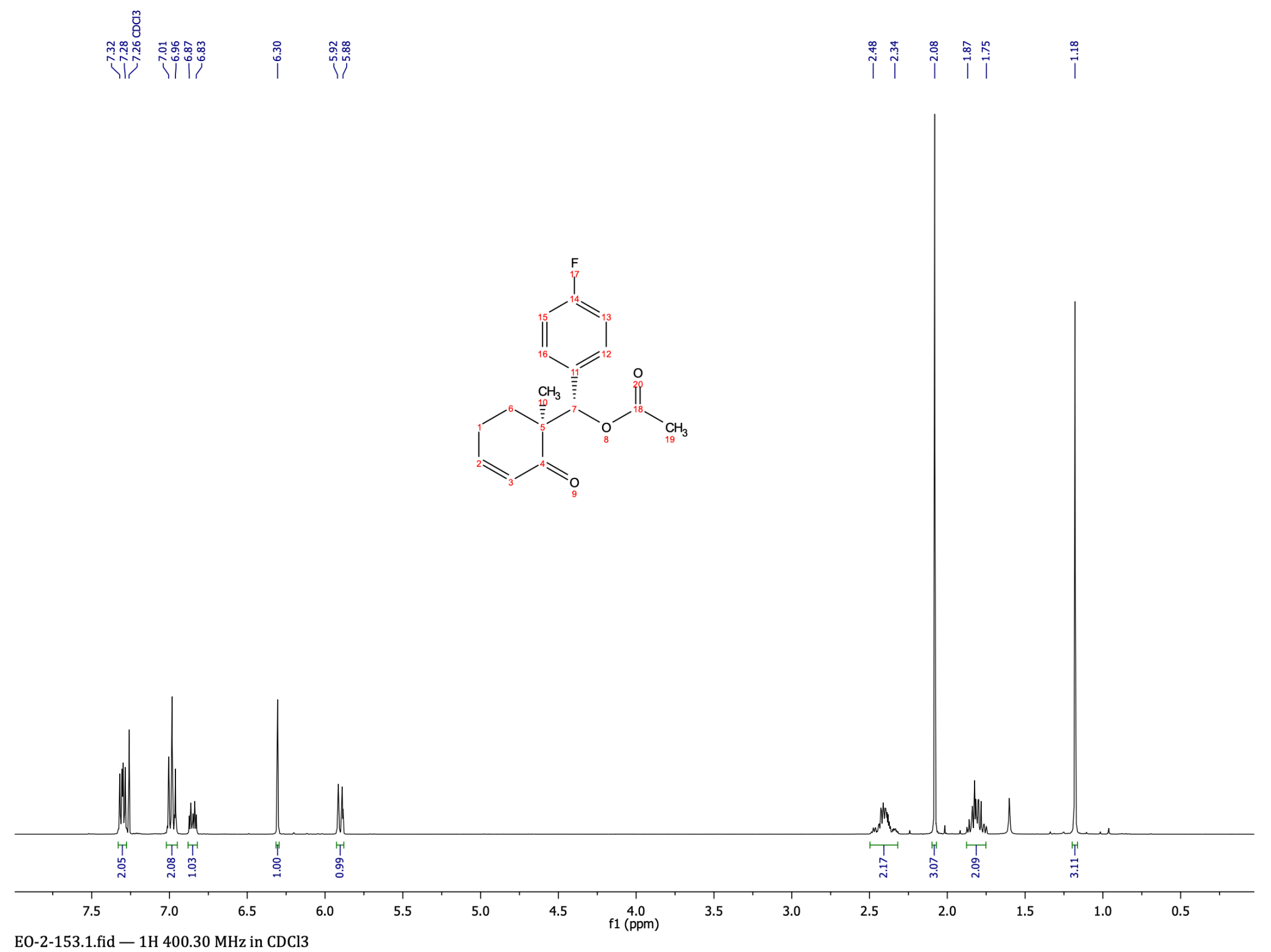

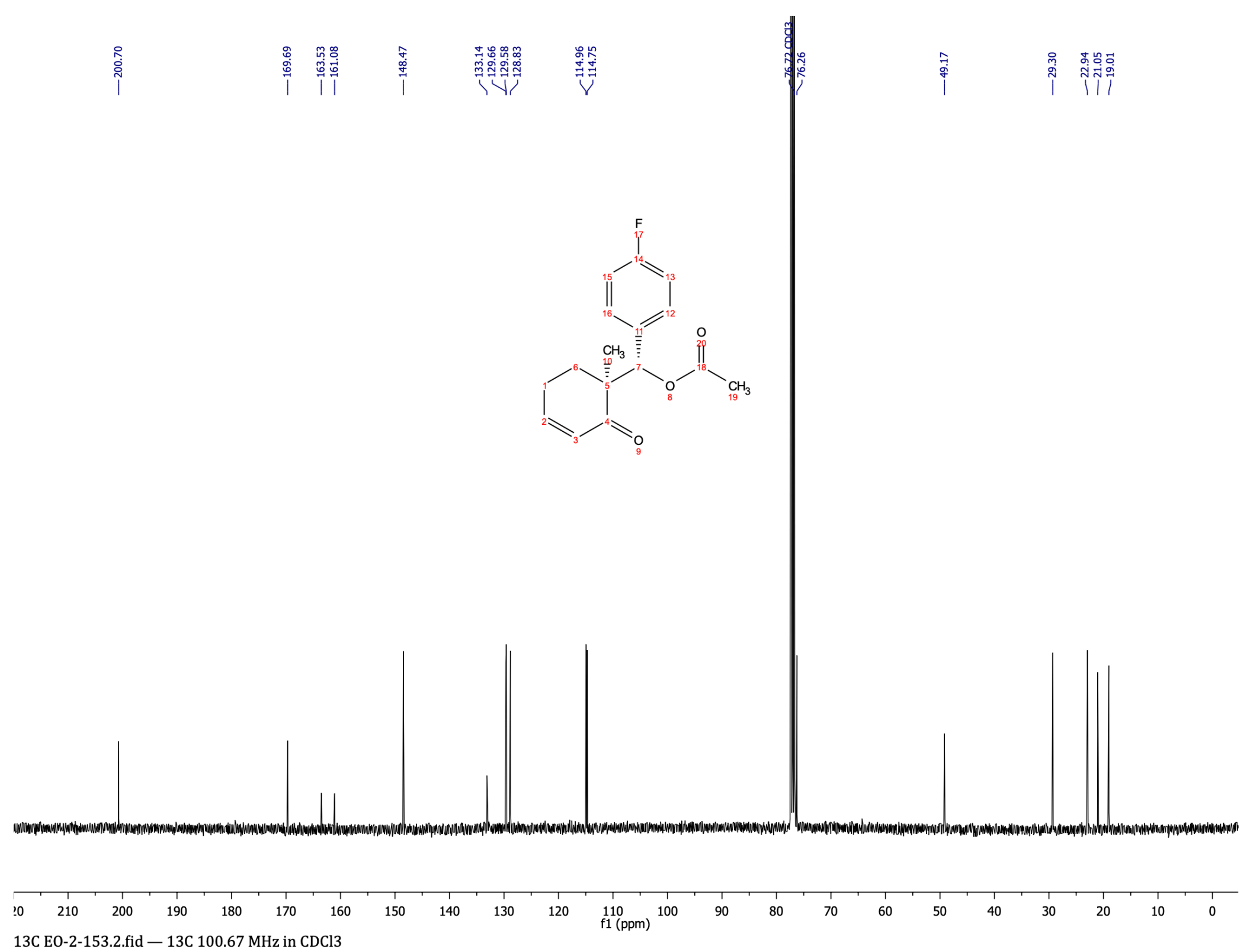

^{1}H NMR (400.30 MHz) and ^{13}C{^{1}H} NMR (100.67 MHz) for Compound **9c** in CDCl3:

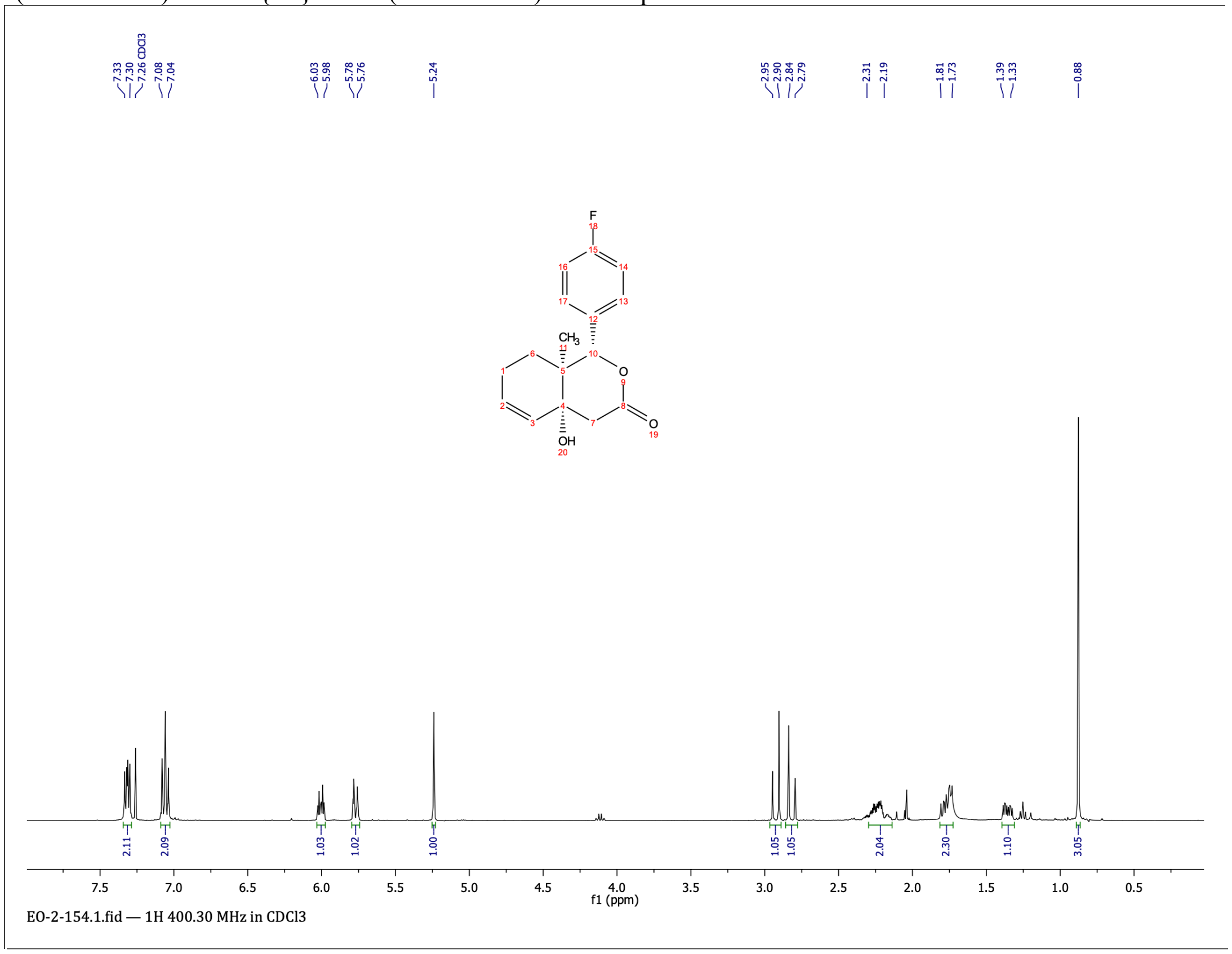

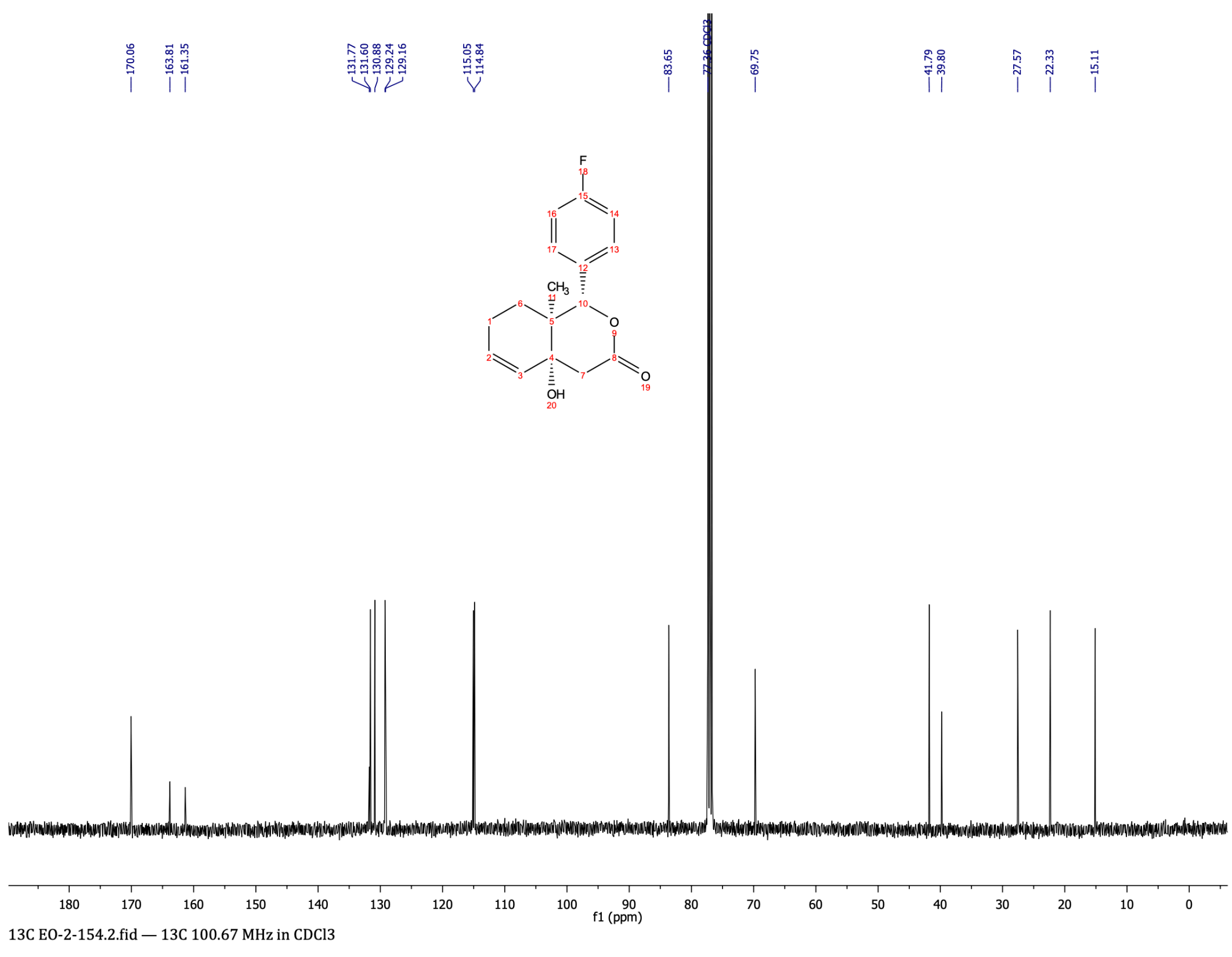

^{1}H NMR (400.30 MHz) and ^{13}C{^{1}H} NMR (100.67 MHz) for Compound **10c** in CDCl3:

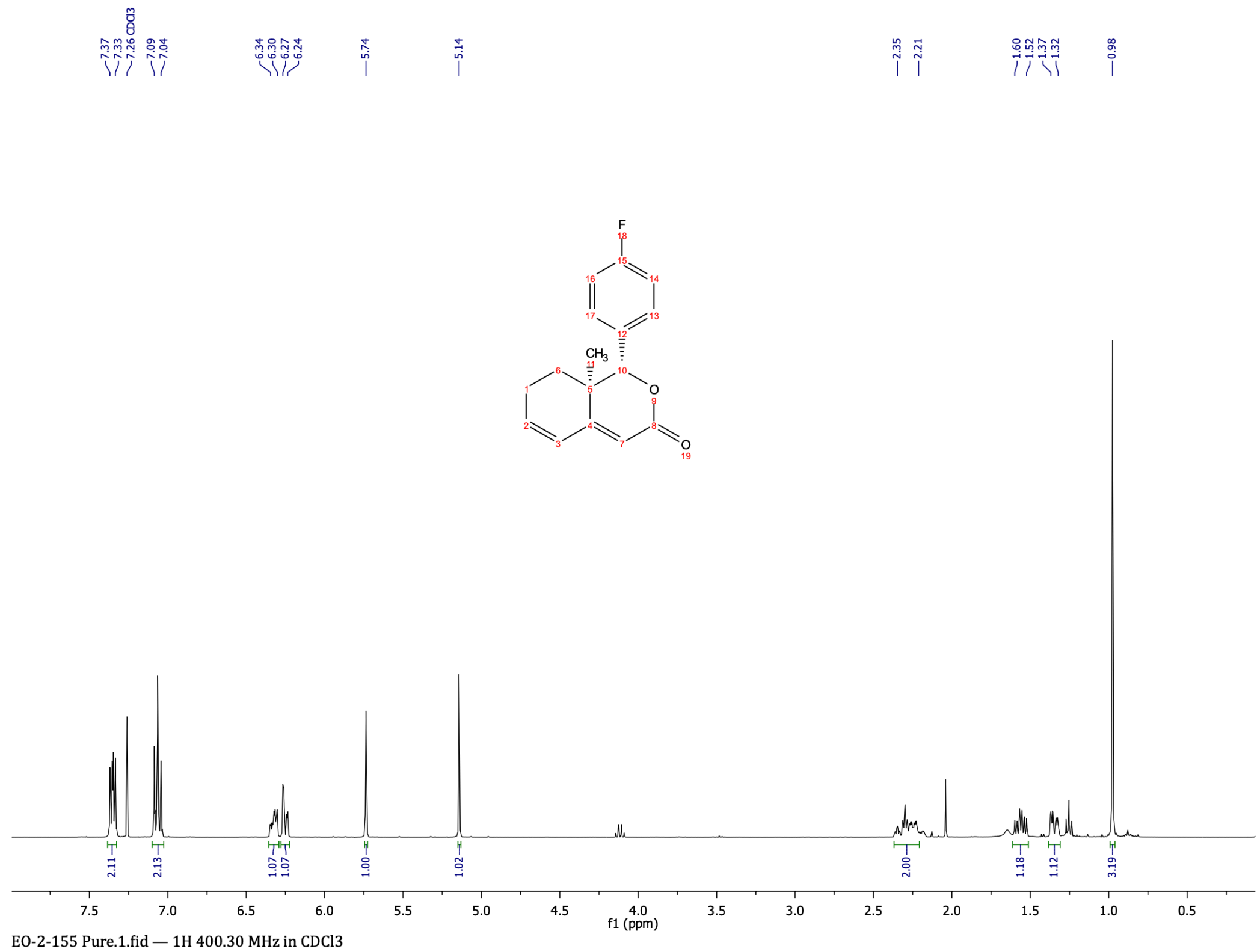

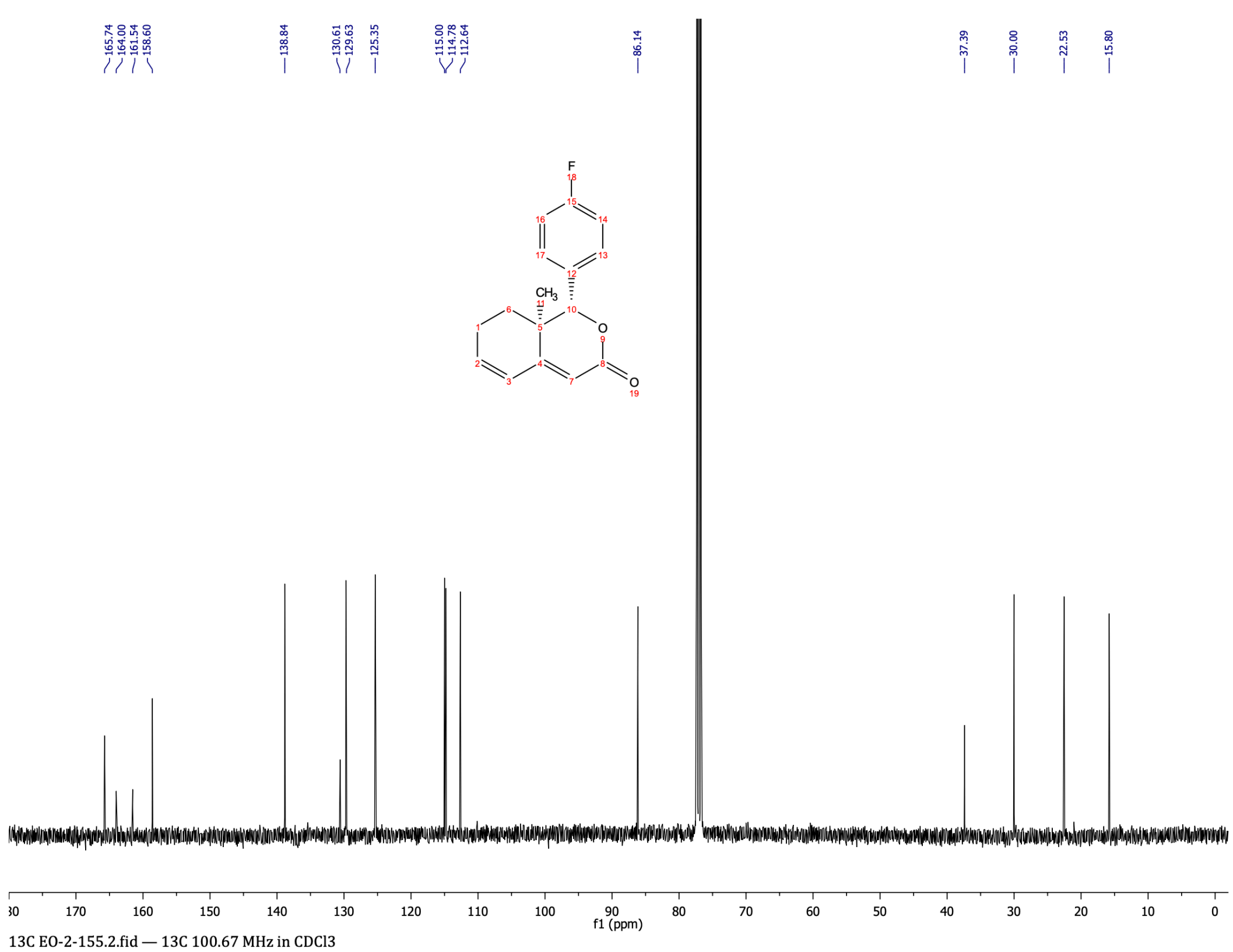

^{1}H NMR (500.30 MHz) and ^{13}C{^{1}H} NMR (125.82 MHz) for Compound **12c** in CDCl3

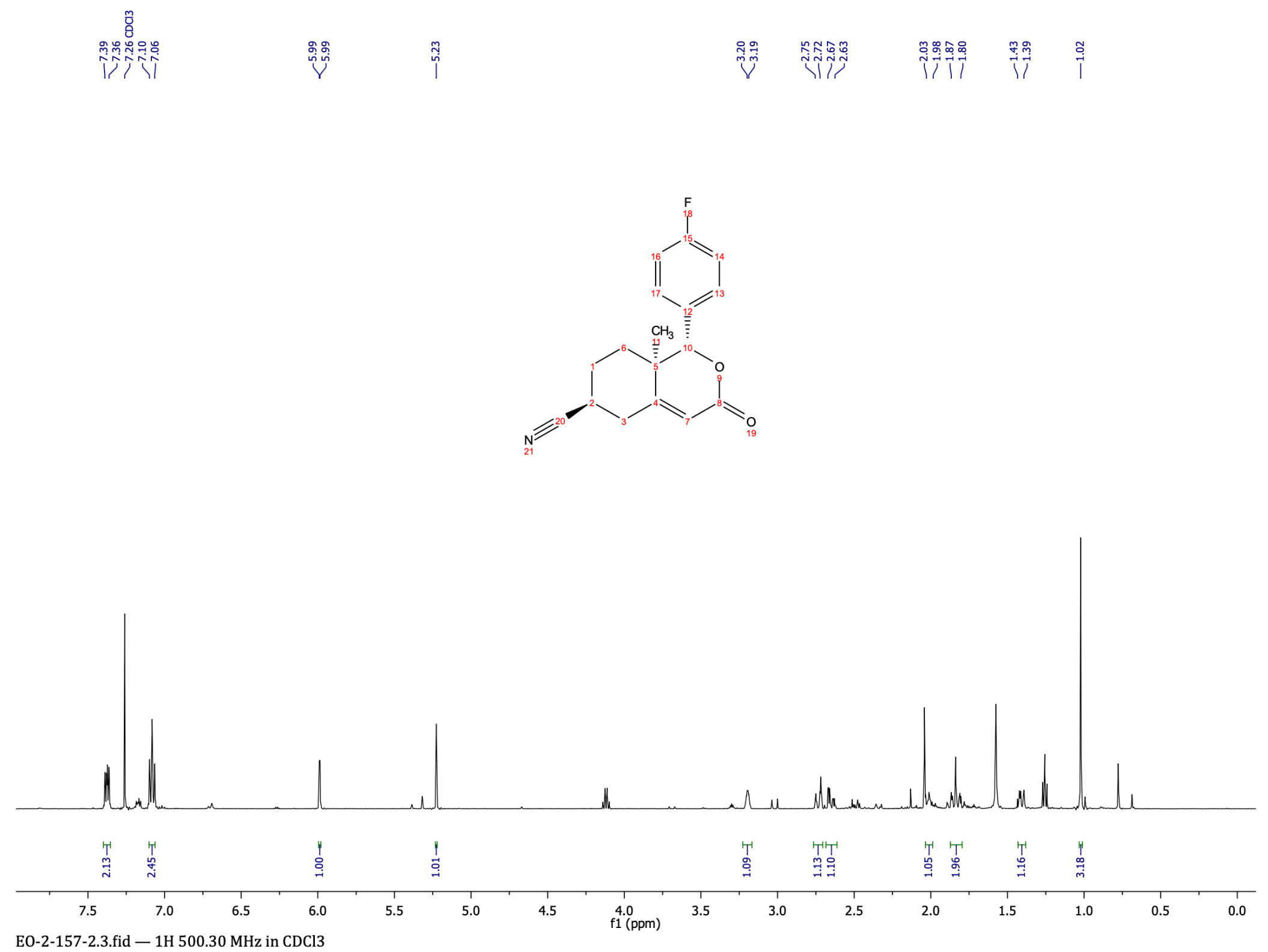

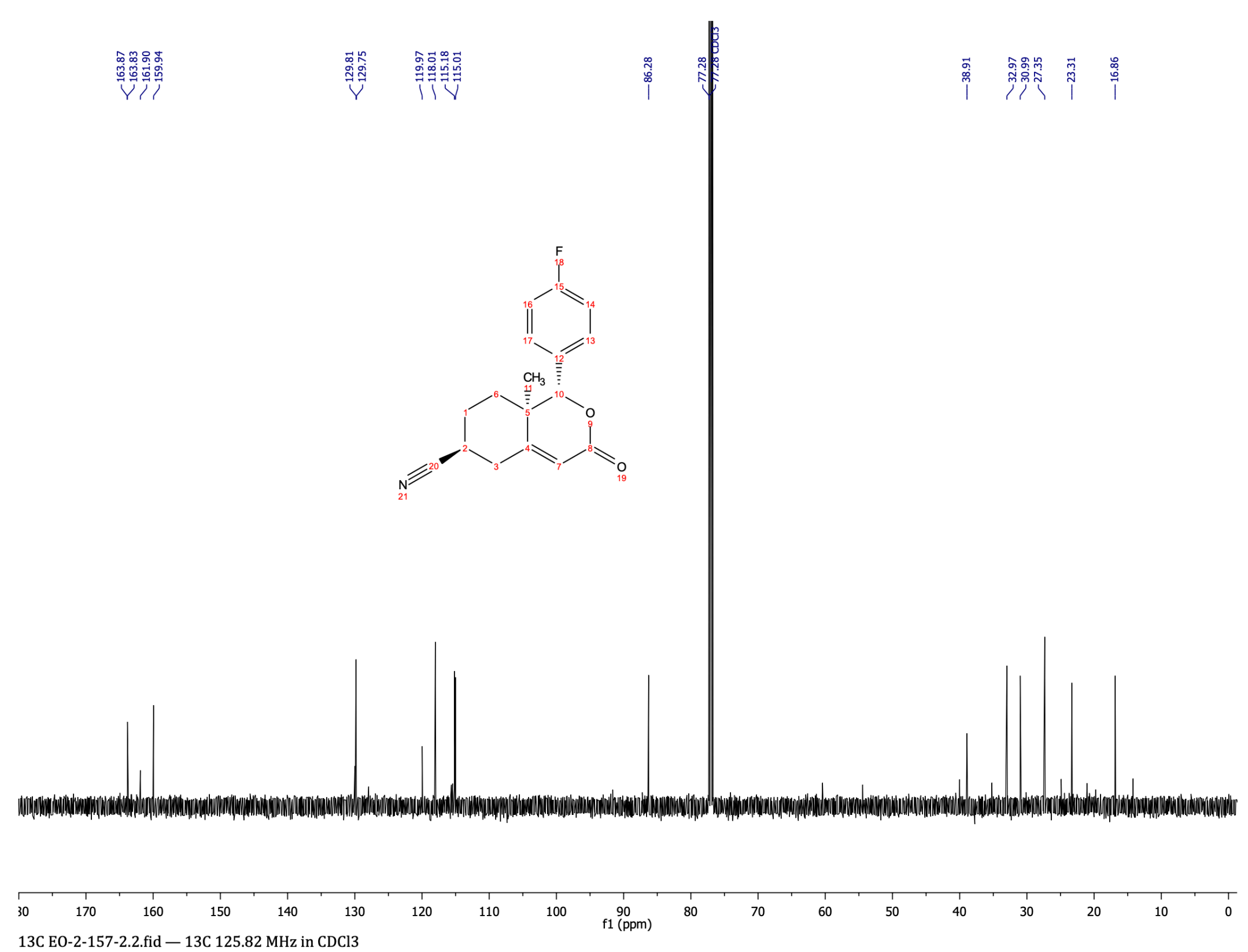

^{1}H NMR (500.30 MHz) and $^{13}C\{^{1}H\}$ NMR (100.67MHz) for Compound **13c** in CDCl3

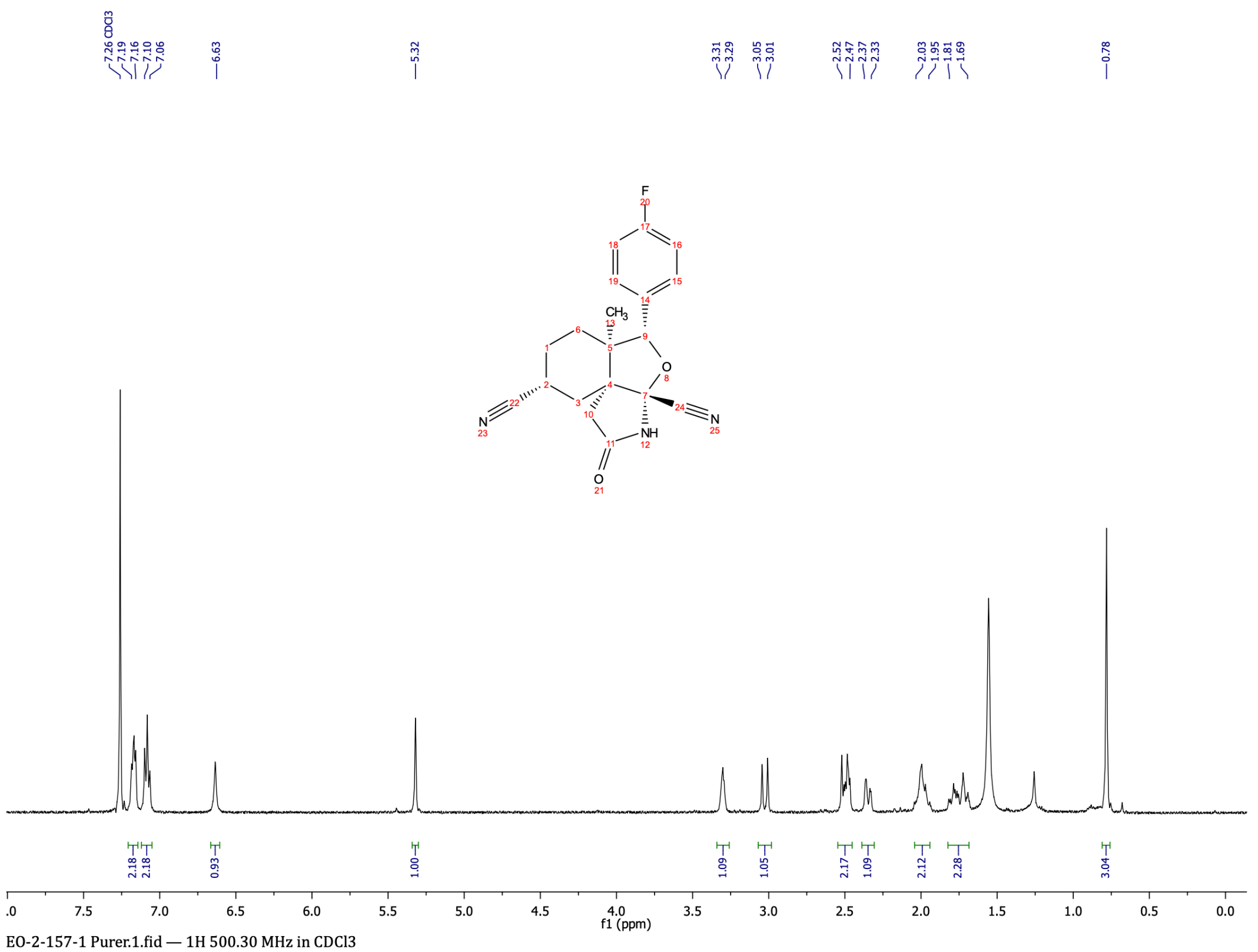

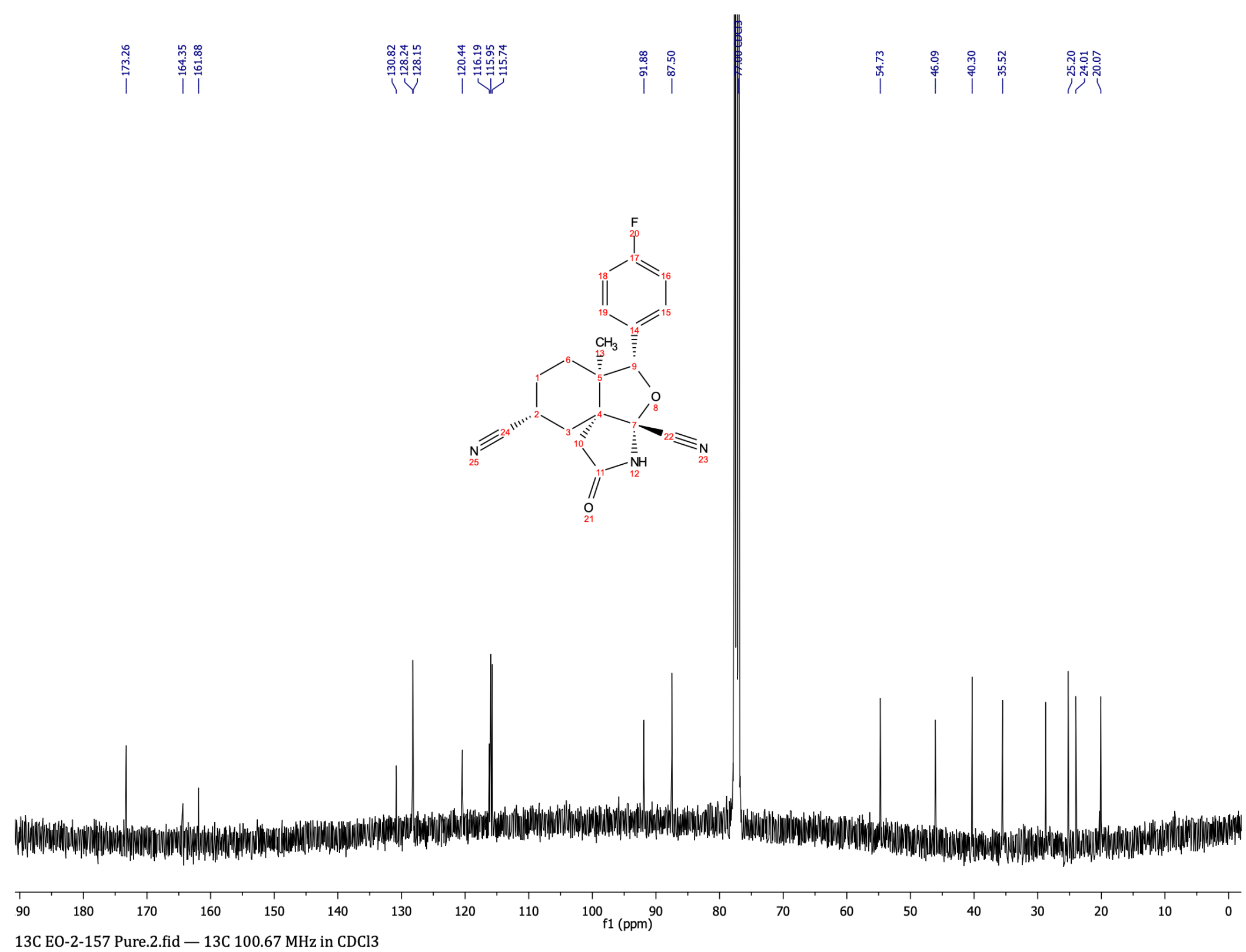

^{1}H NMR (600.21 MHz) and $^{13}C\{^{1}H\}$ (150.94 MHz) for Compound **7d** in CDCl3:

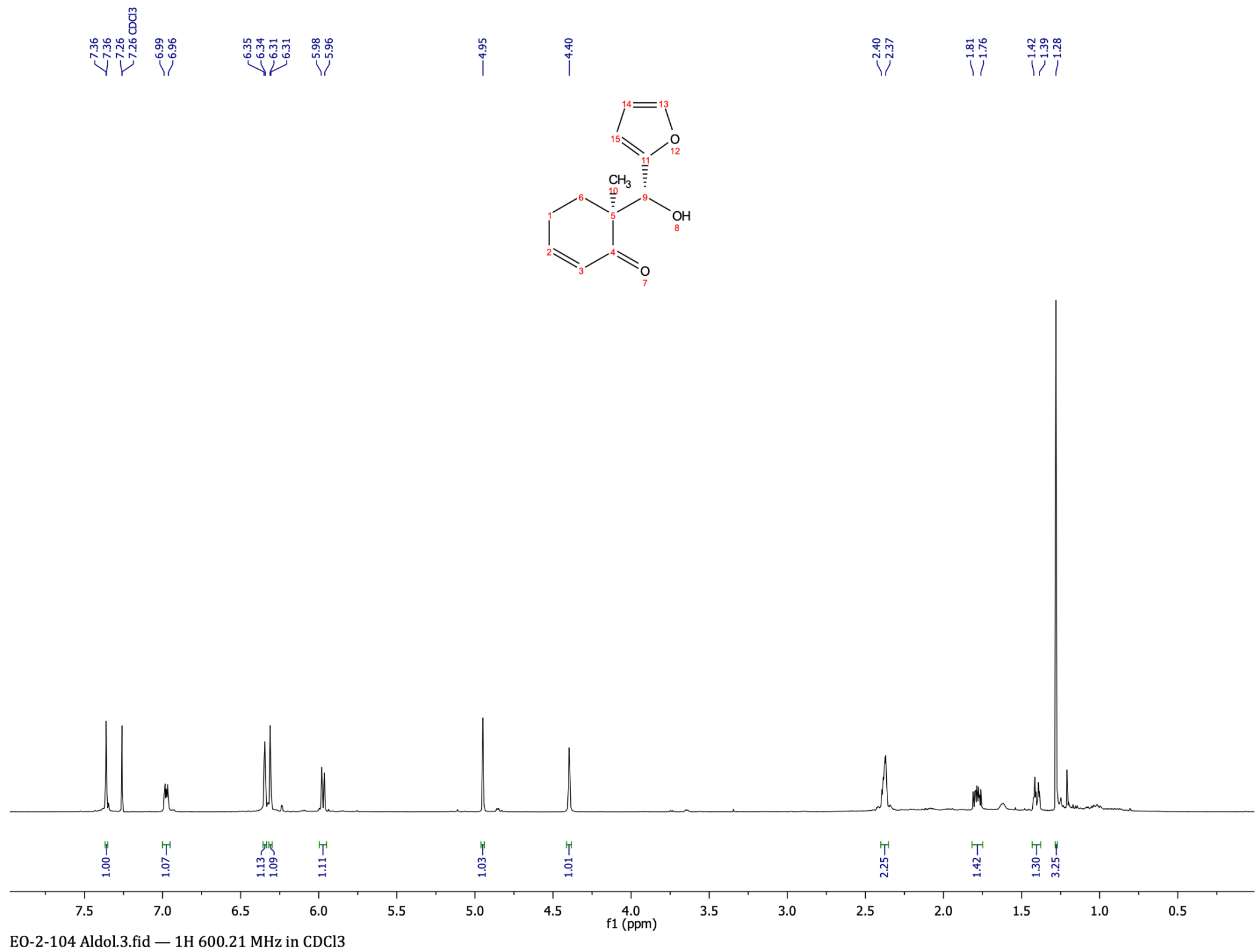

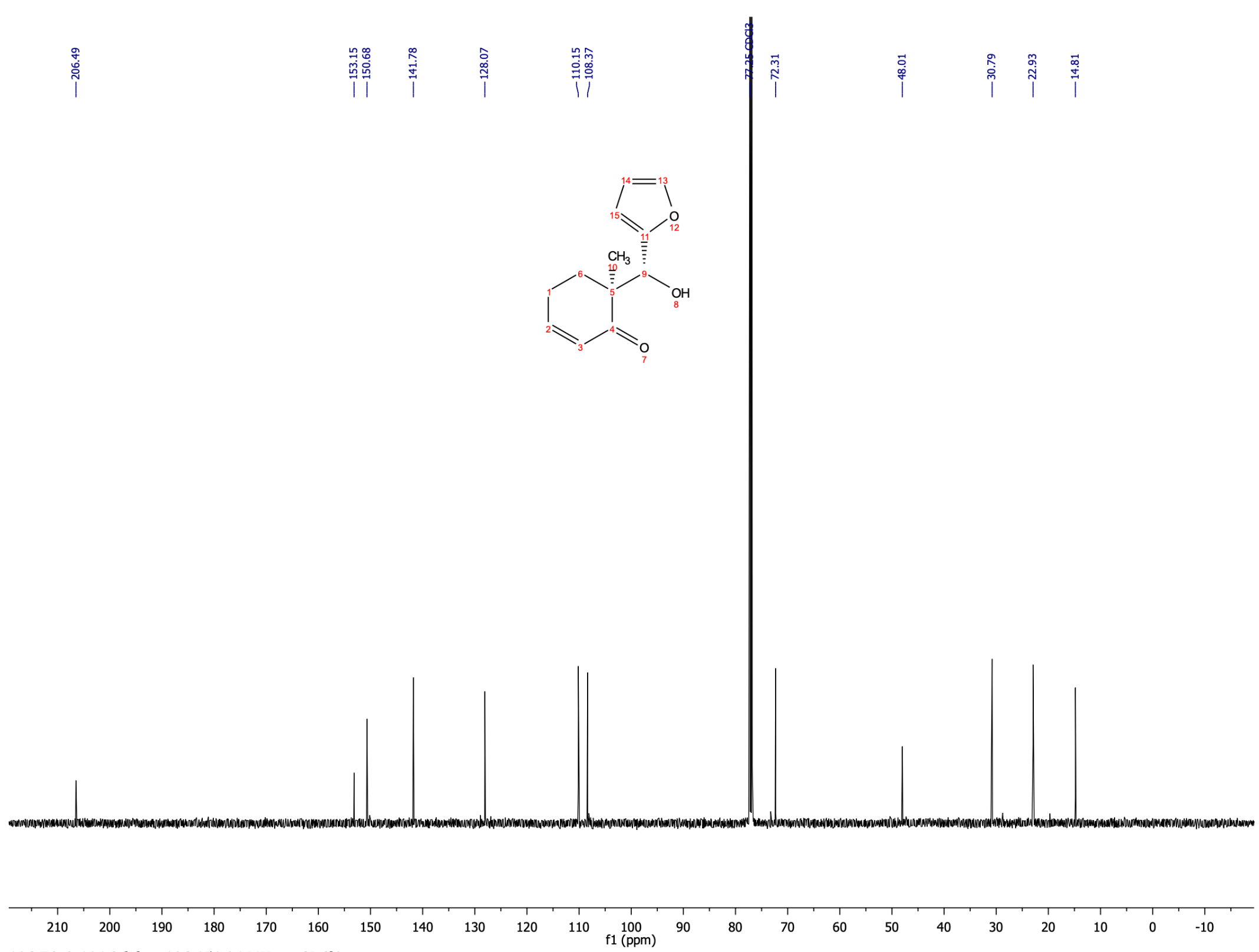

^{1}H NMR (500.30 MHz) and ^{13}C{^{1}H} (125.82 MHz) for Compound **8d** in CDCl3:

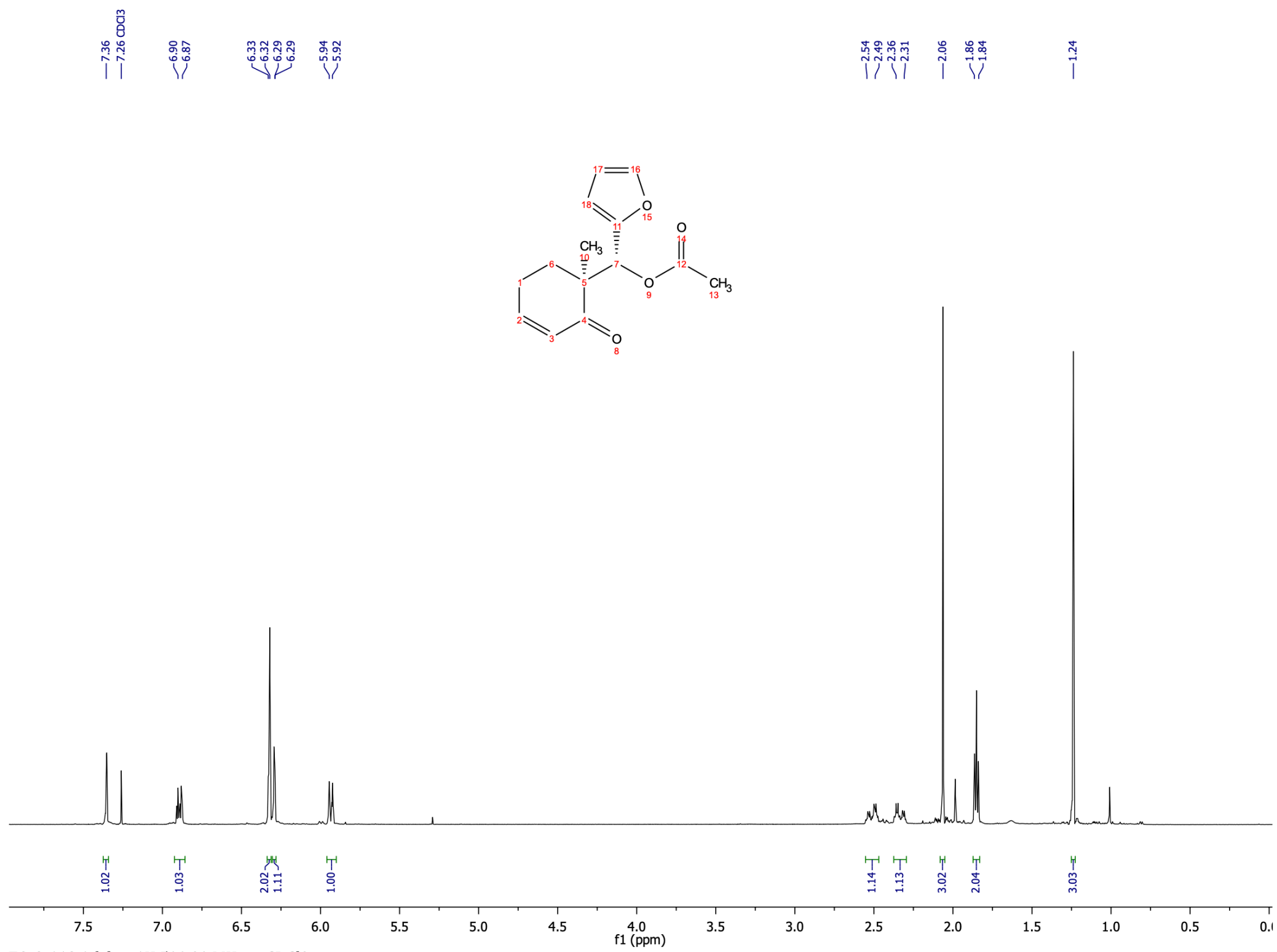

EO-2-112.1.fid — 1H 500.30 MHz in CDCl3

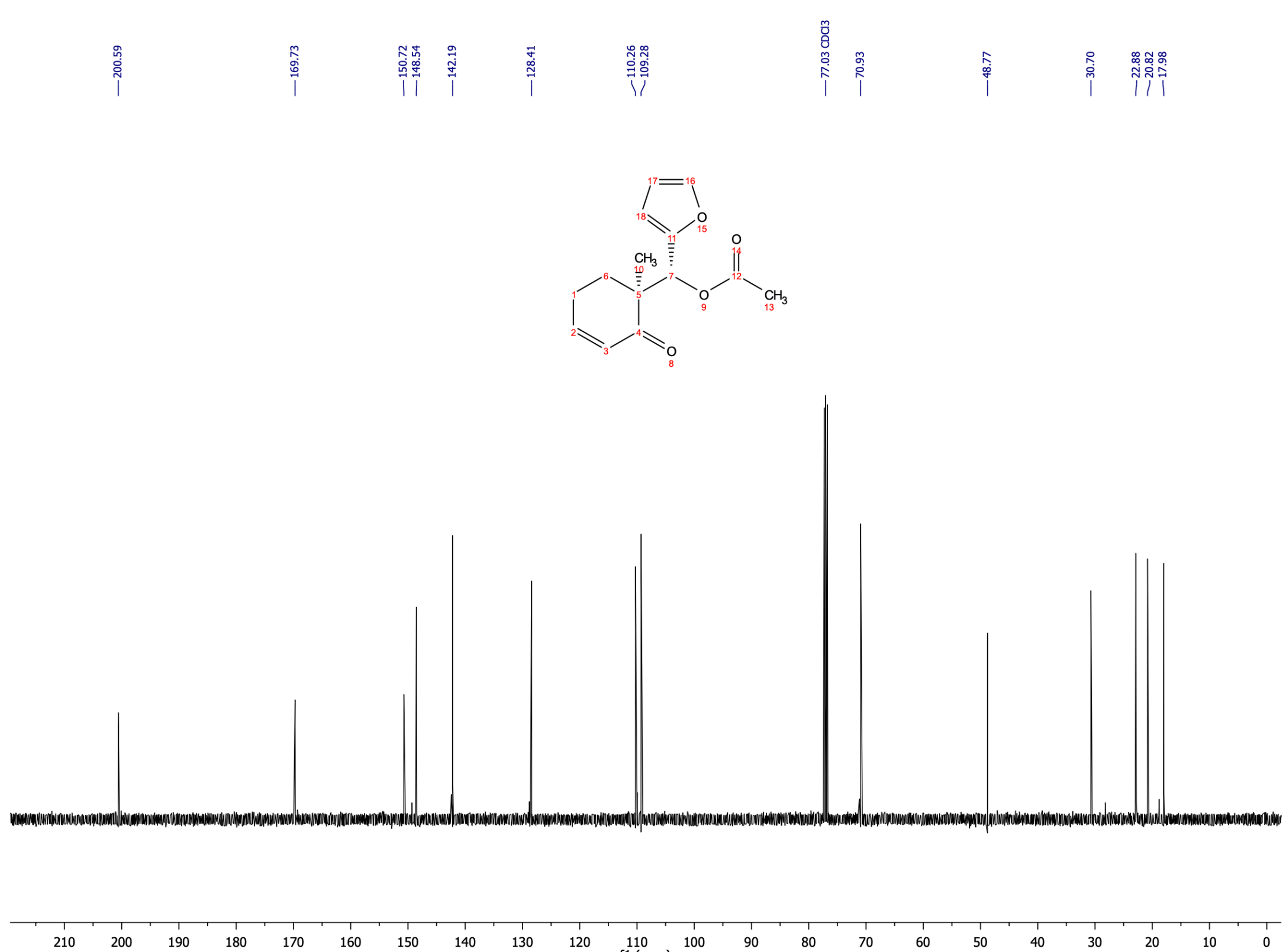

13C EO-2-112.2.fid — 13C 125.82 MHz in CDCl3

^{1}H NMR (600.21MHz) and $^{13}C\{^{1}H\}$ (150.94 MHz) for Compound **9d** in CDCl3:

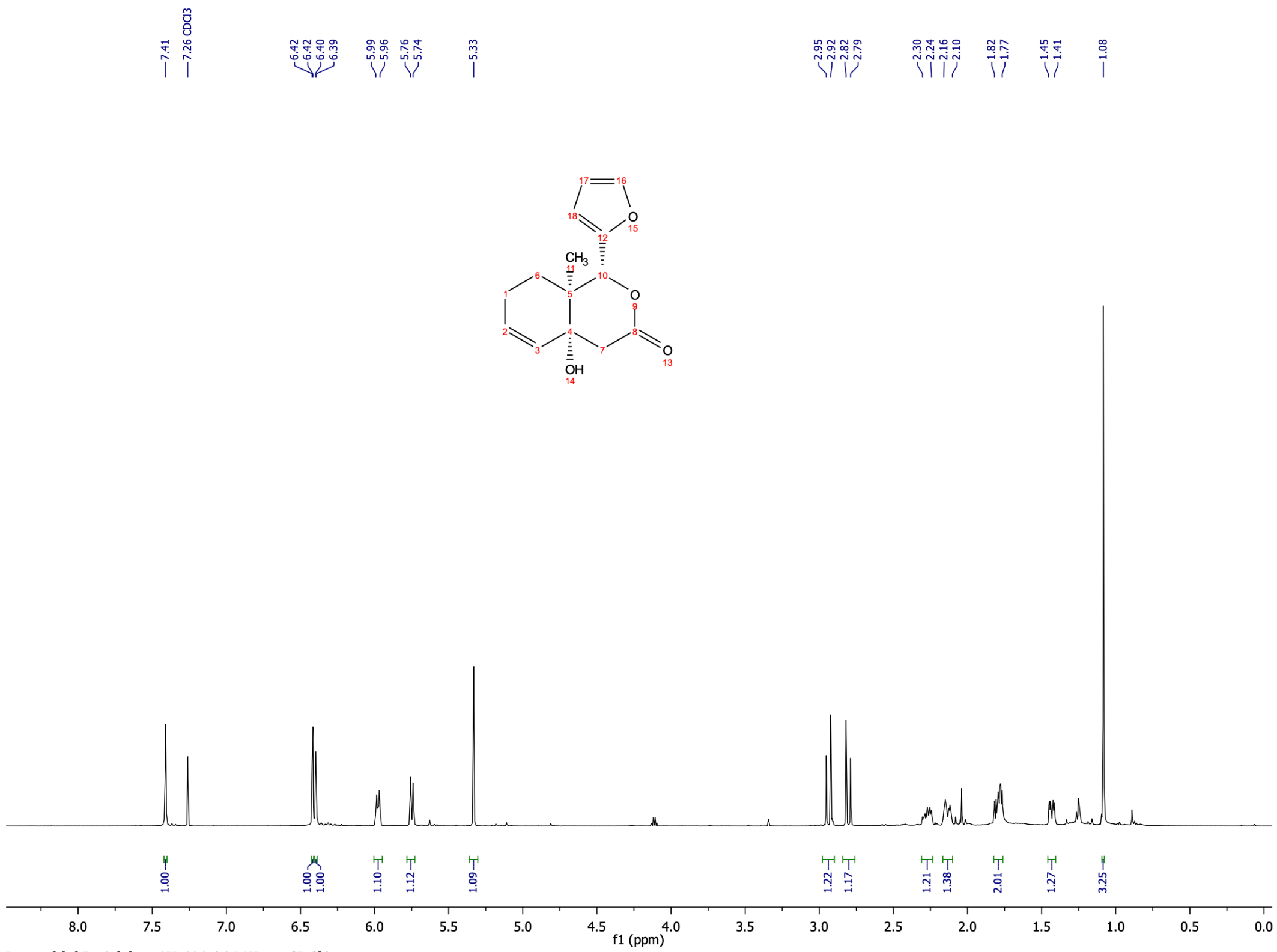

Intra aldol Bi.1.fid — 1H 600.21 MHz in CDCl3

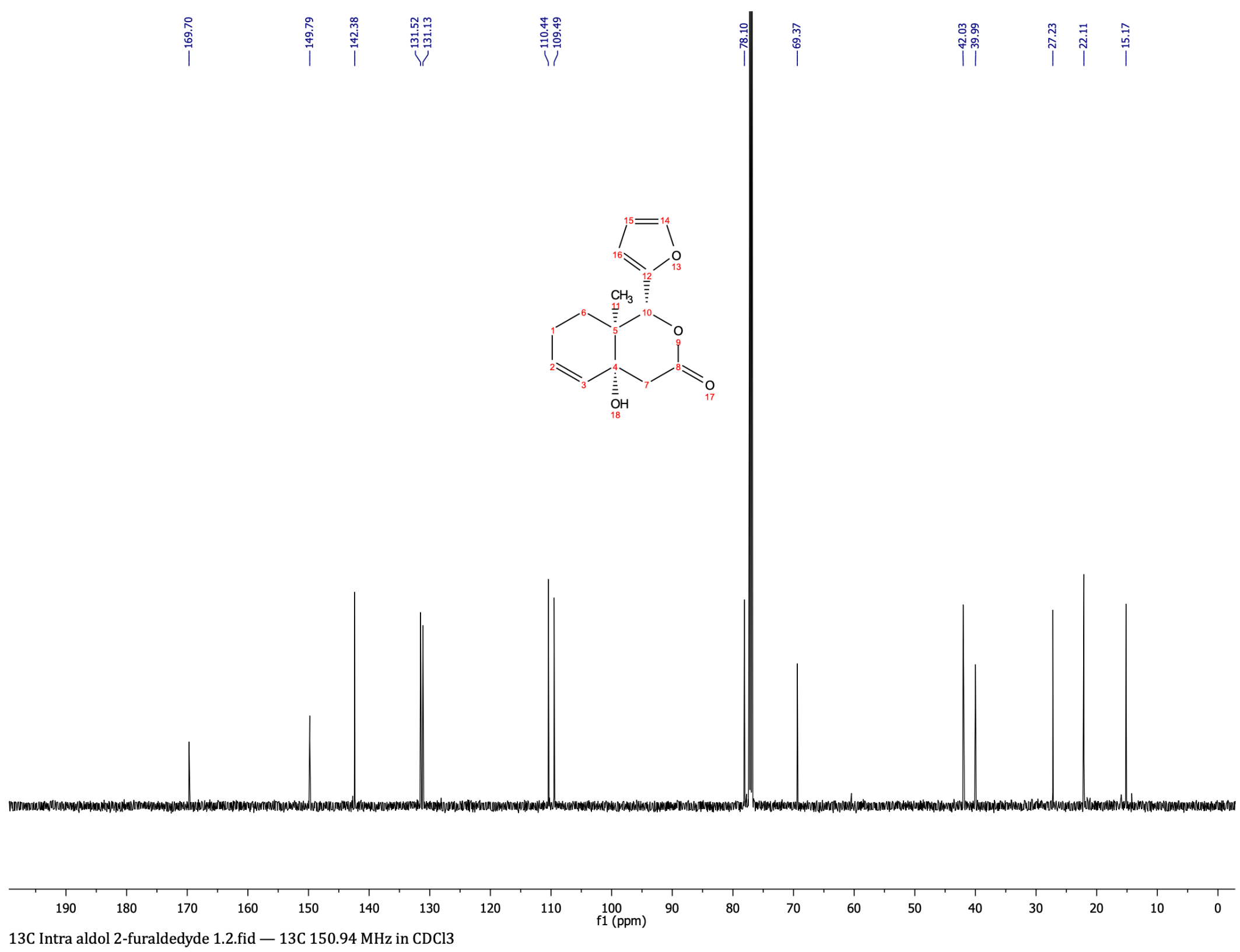

13C Intra aldol 2-furaldedyde 1.2.fid — 13C 150.94 MHz in CDCl3

^{1}H NMR (500.30 MHz) and $^{13}C\{^{1}H\}$ NMR (125.82 MHz) for Compound **10d** in CDCl3:

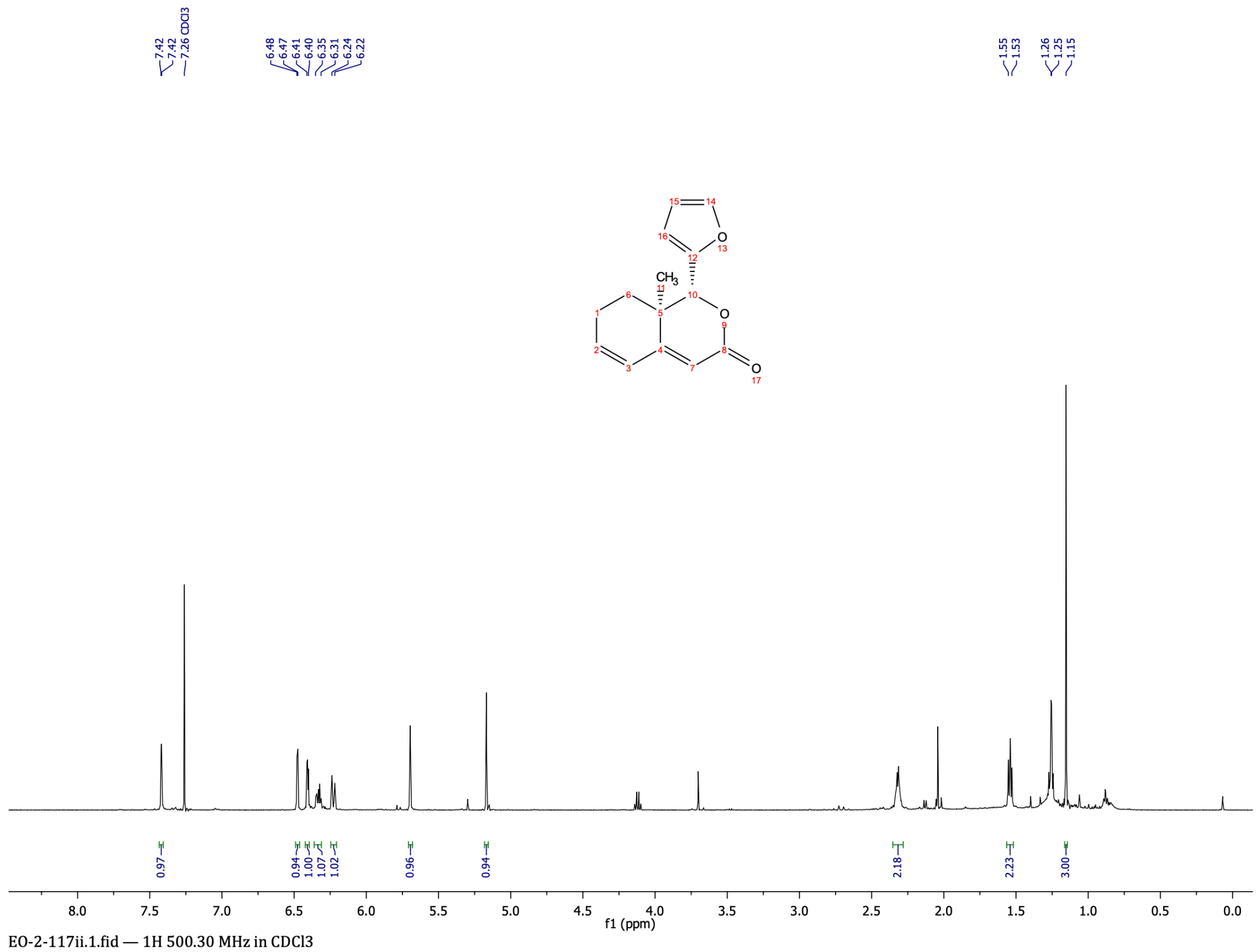

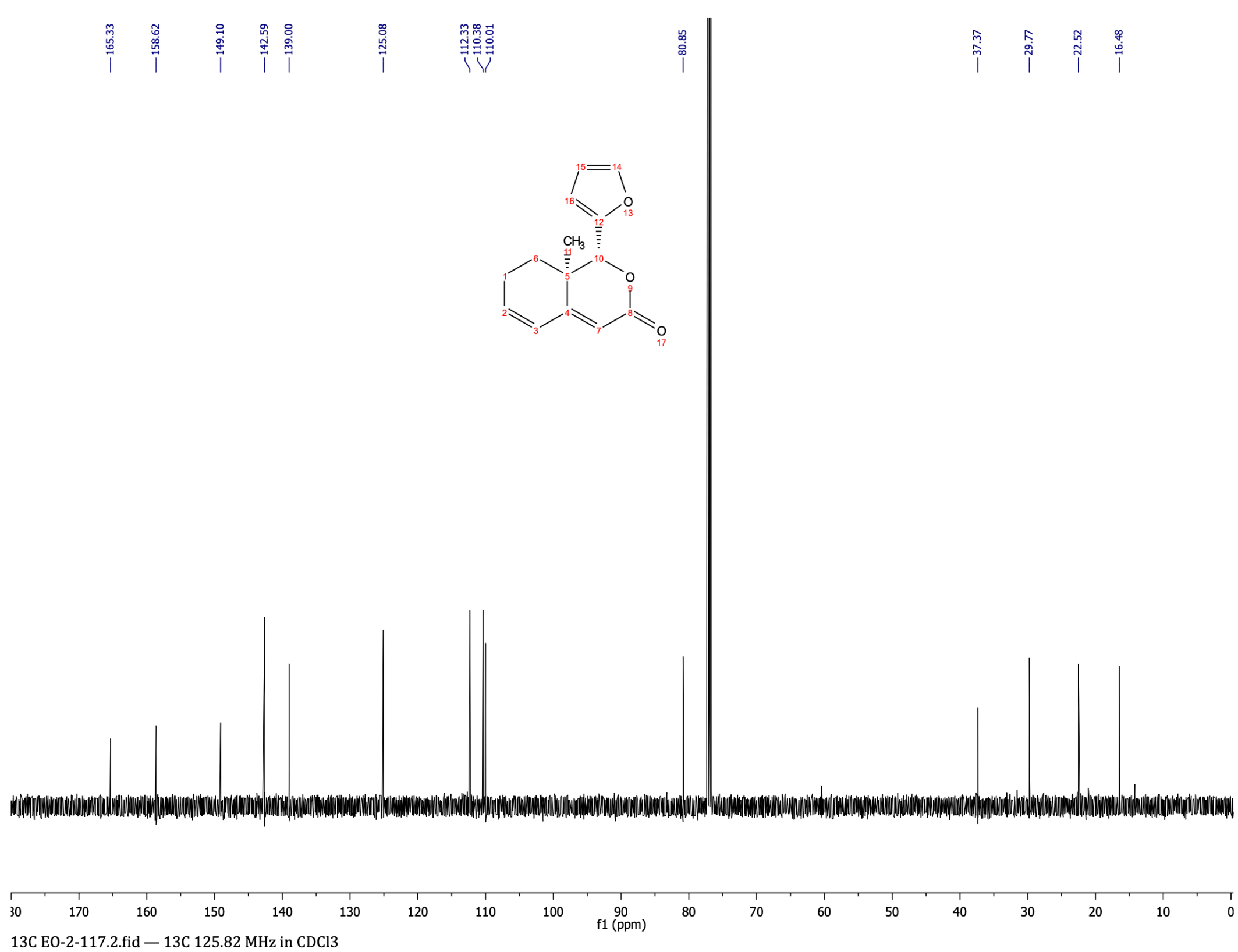

^{1}H NMR (500.30 MHz) and ^{13}C{^{1}H} NMR (125.82 MHz) for Compound **12d** in CDCl3:

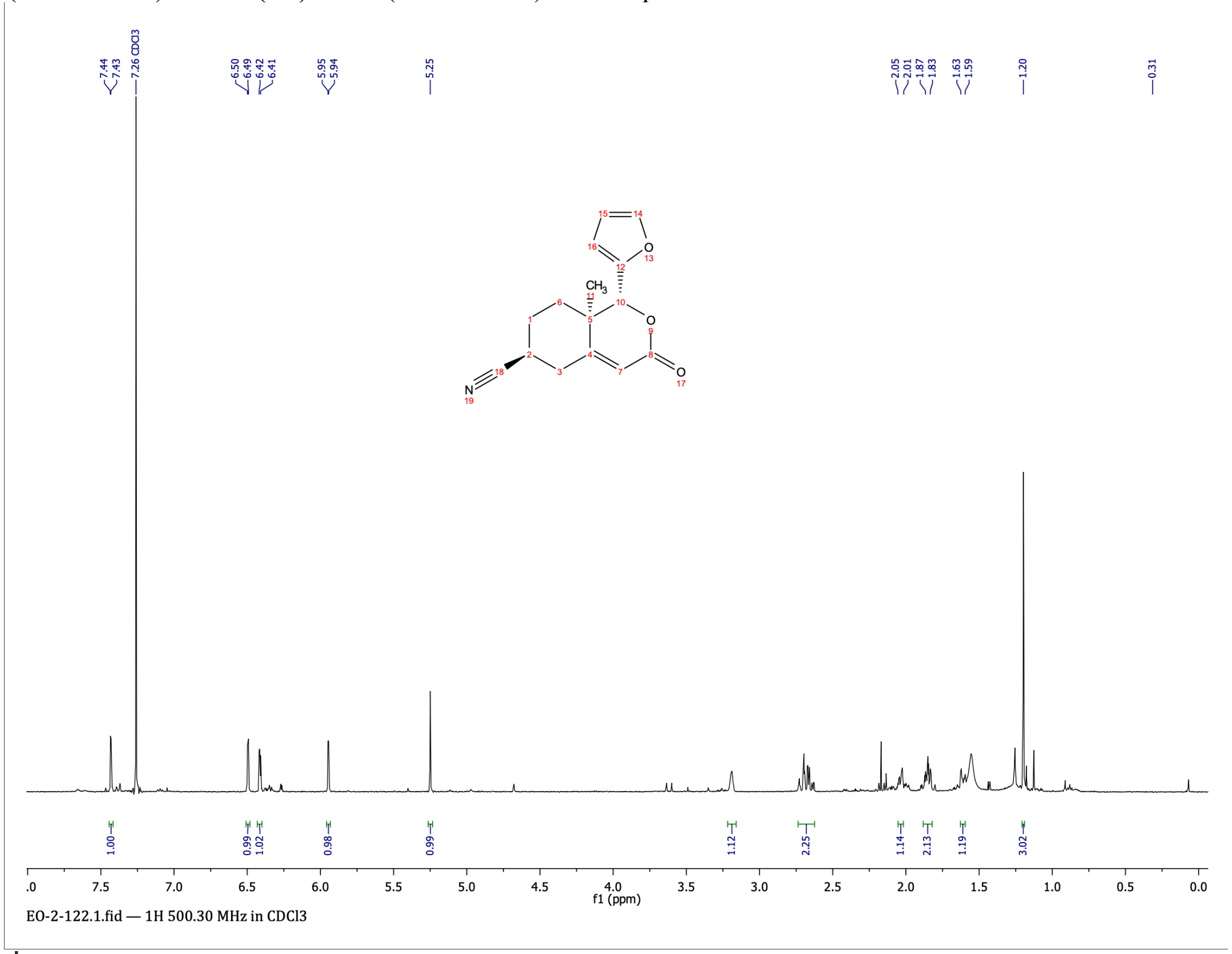

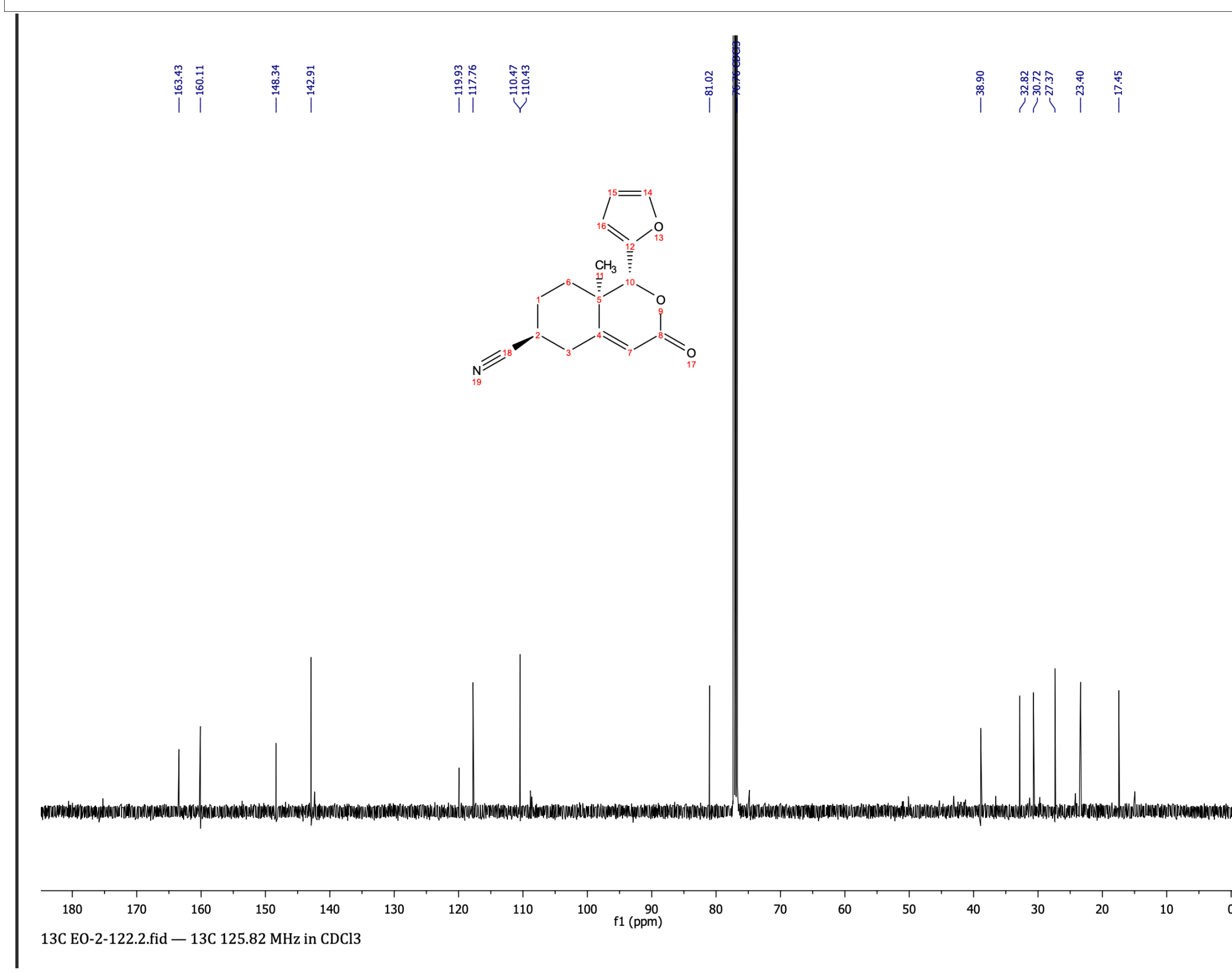

^{1}H NMR (500.30 MHz) and ^{13}C{^{1}H} NMR (125.82 MHz) for Compound **7e** in CDCl3:

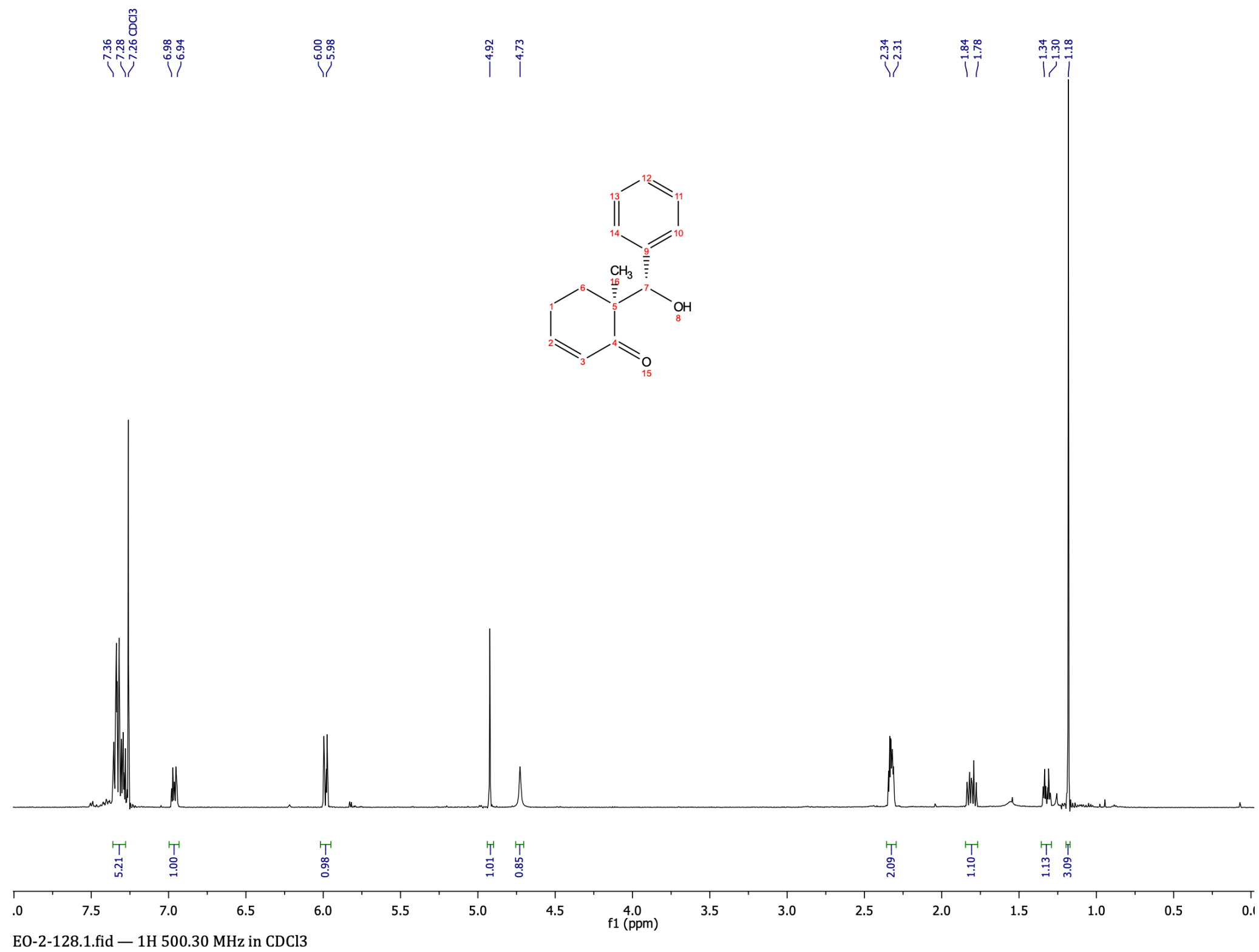

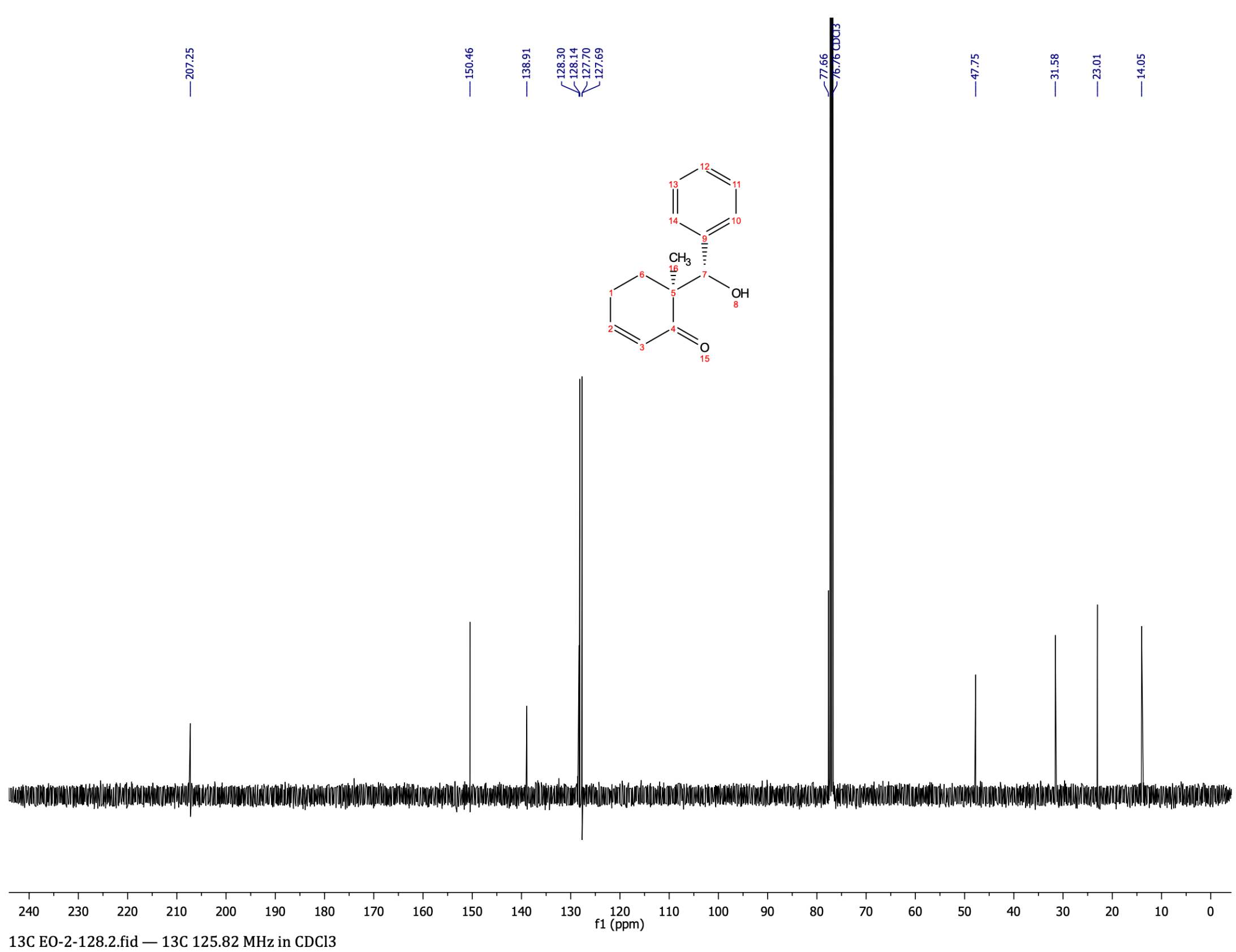

^{1}H NMR (500.30 MHz) and $^{13}C\{^{1}H\}$ NMR (125.82 MHz) for Compound **8e** in CDCl3:

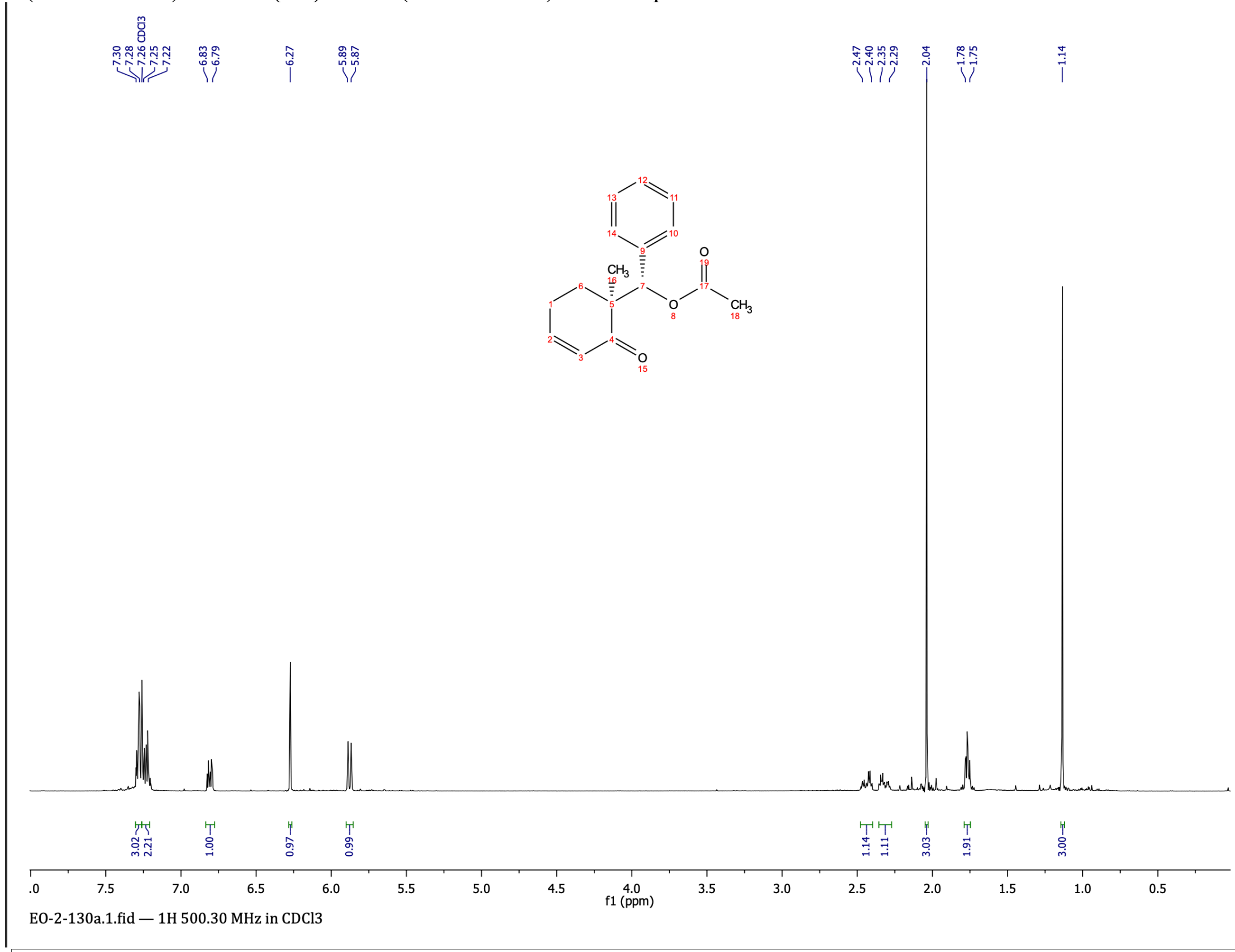

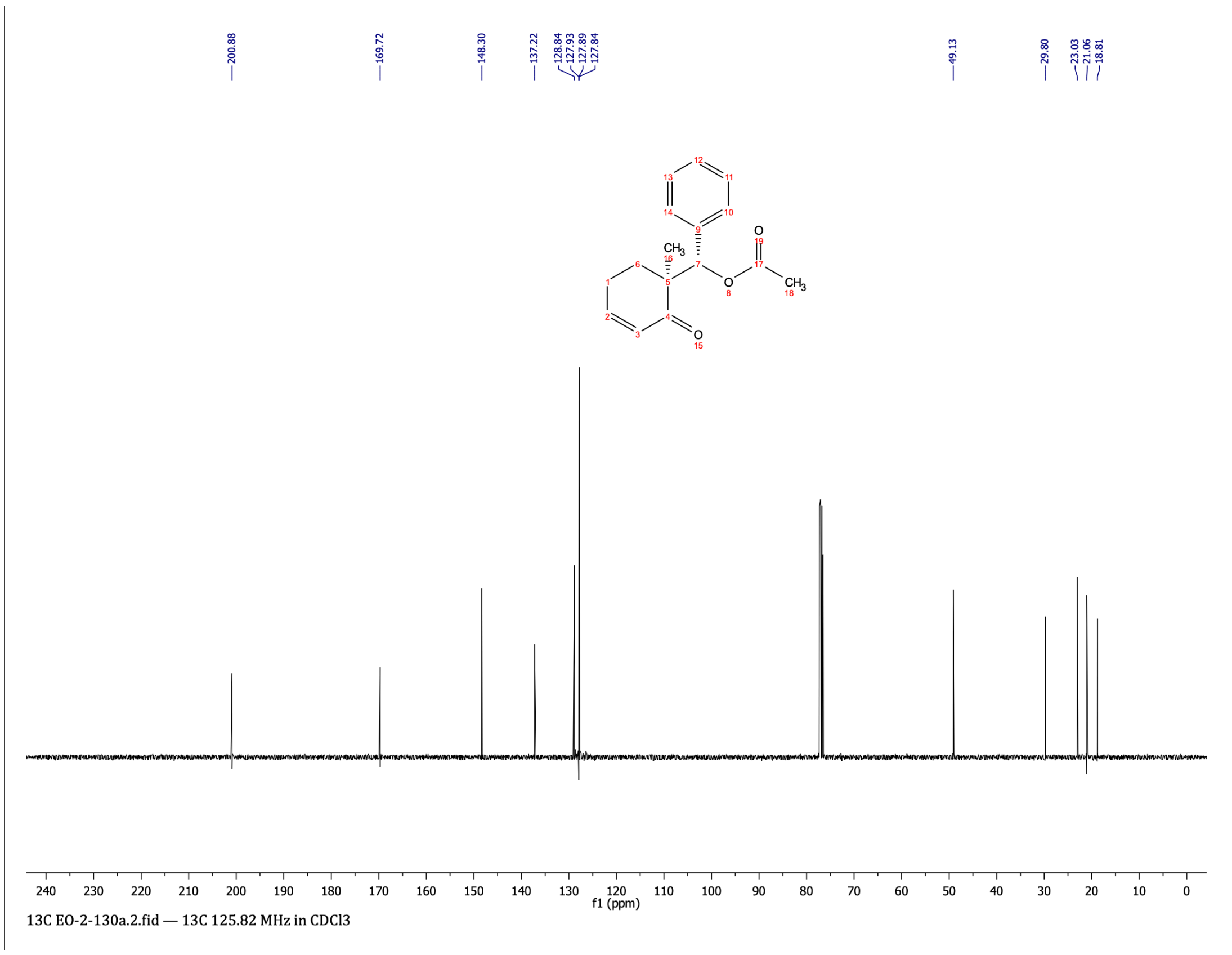

^{1}H NMR (500.30 MHz) and ^{13}C{^{1}H} NMR (125.82 MHz) for Compound **9e** in CDCl3:

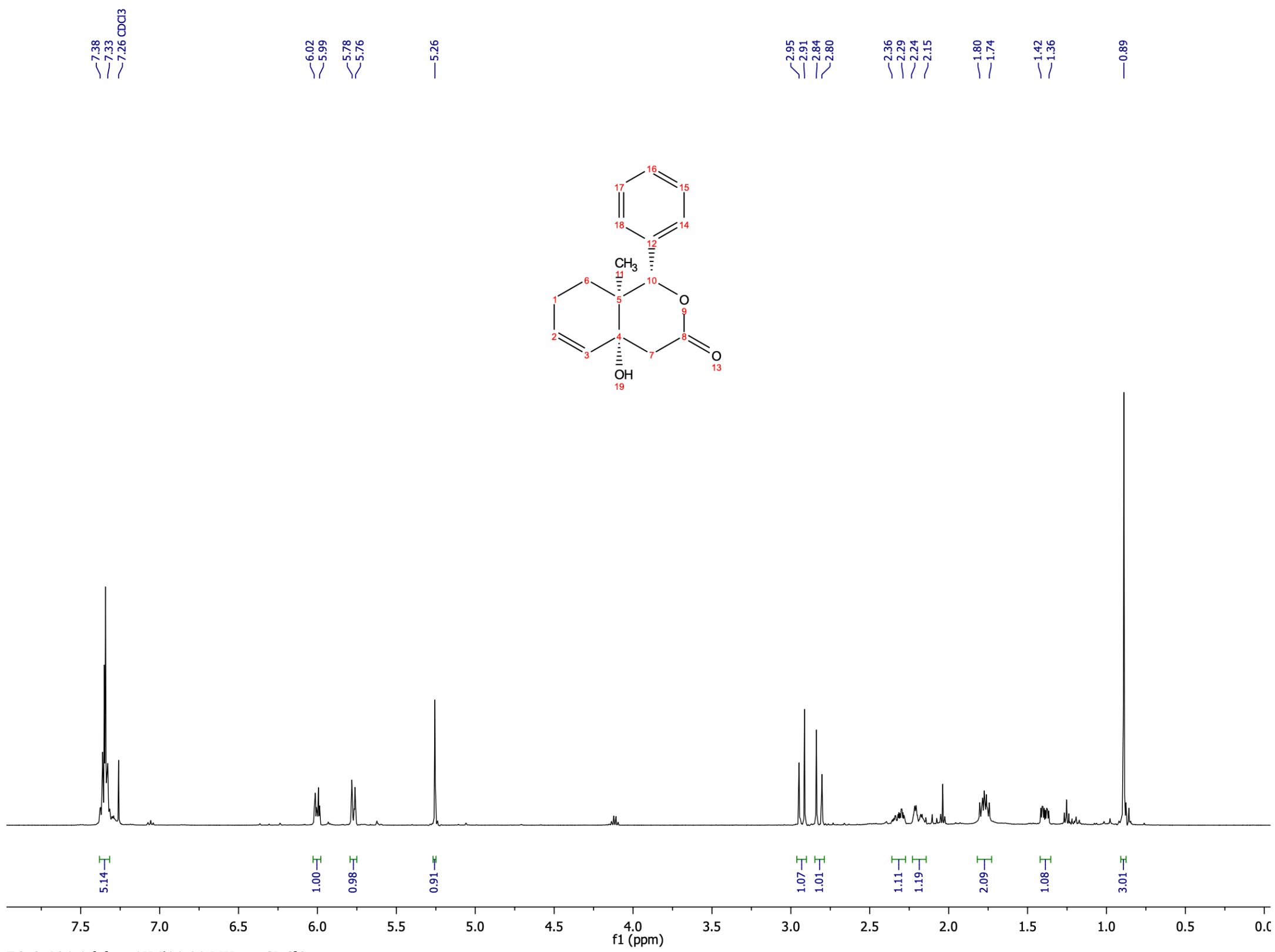

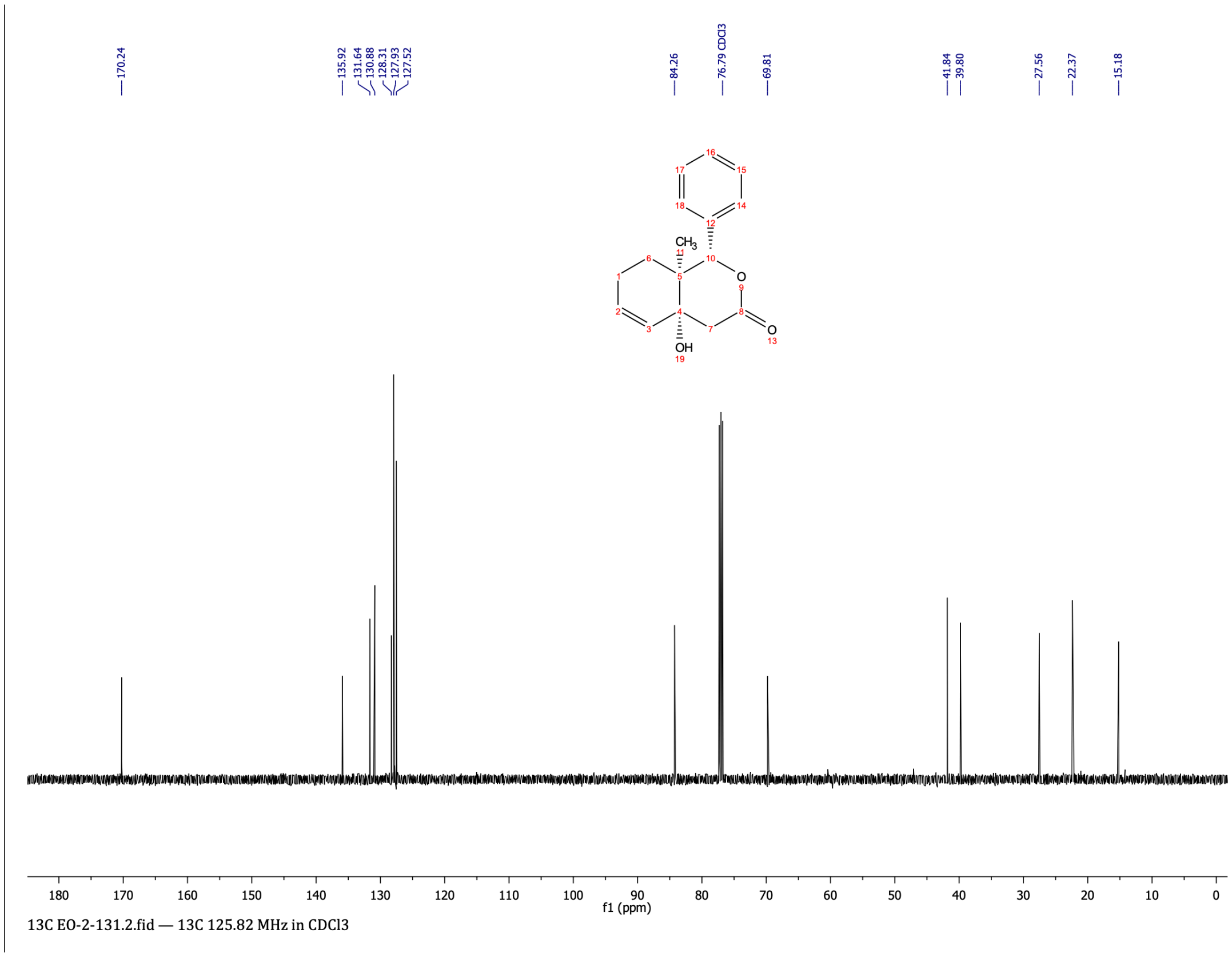

^{1}H NMR (500.30 MHz) and $^{13}C\{^1H\}$ NMR (125.82 MHz) for Compound **10e** in CDCl3:

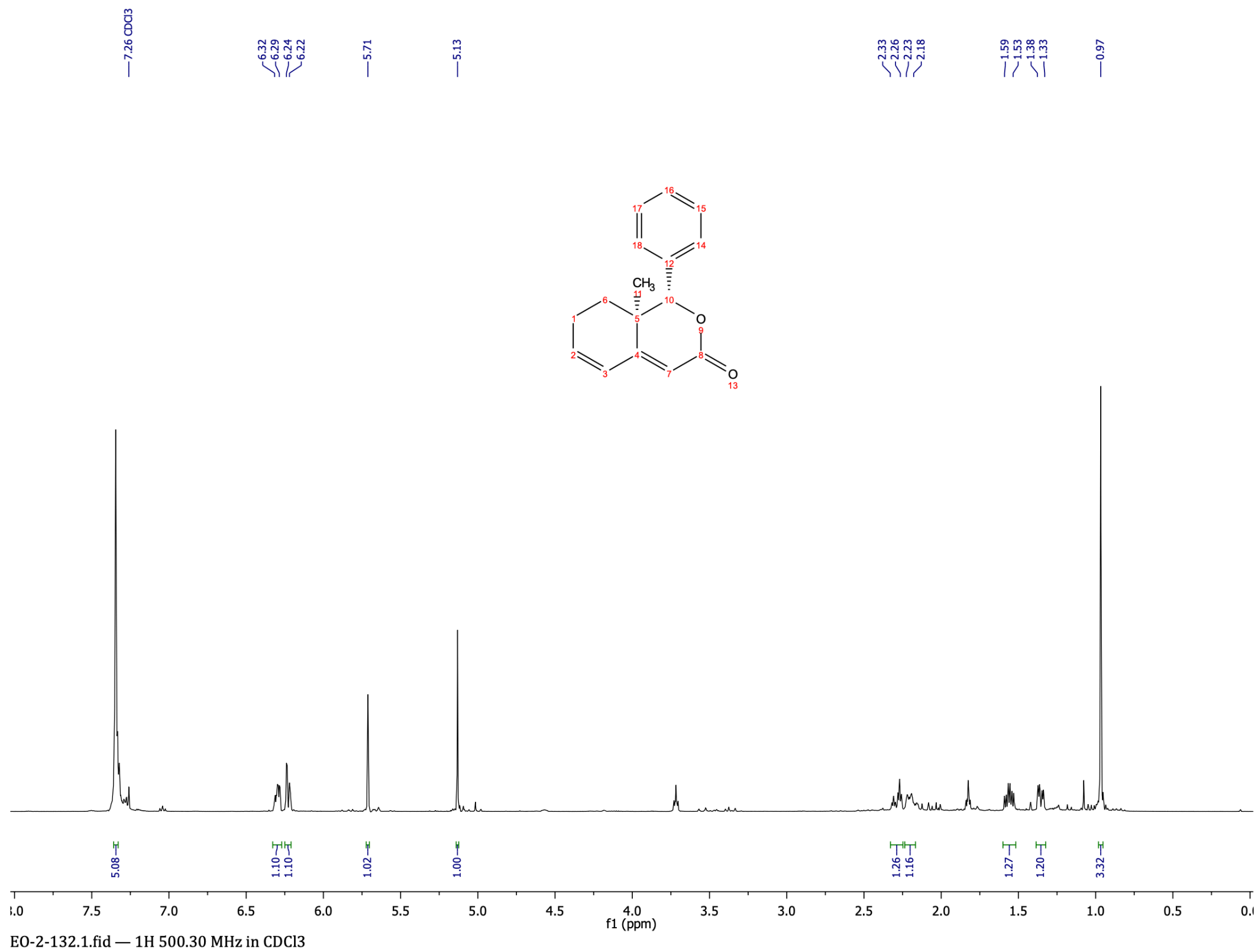

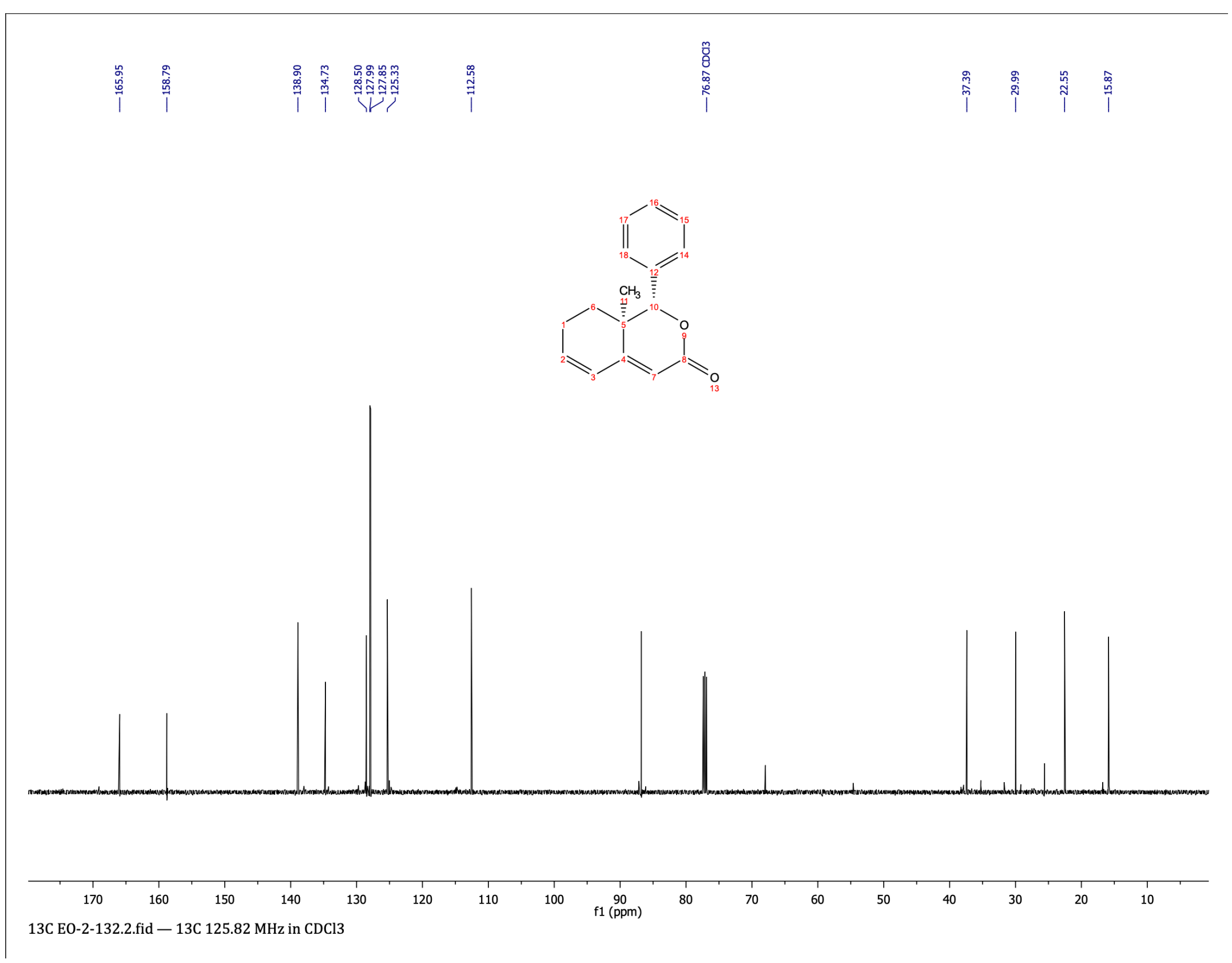

^{1}H NMR (500.30 MHz) and $^{13}C\{^{1}H\}$ NMR (125.82 MHz) for Compound **12e** in CDCl3:

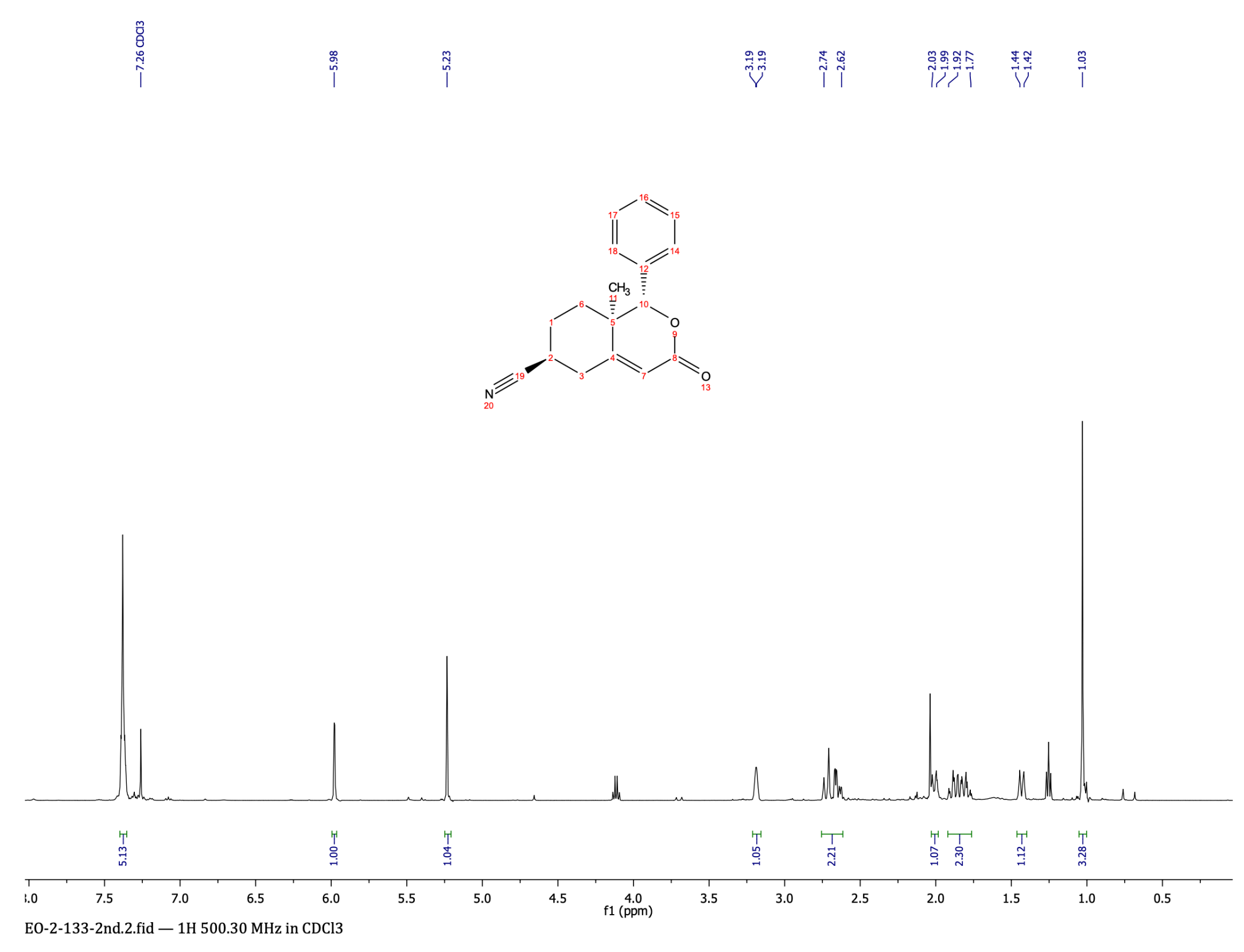

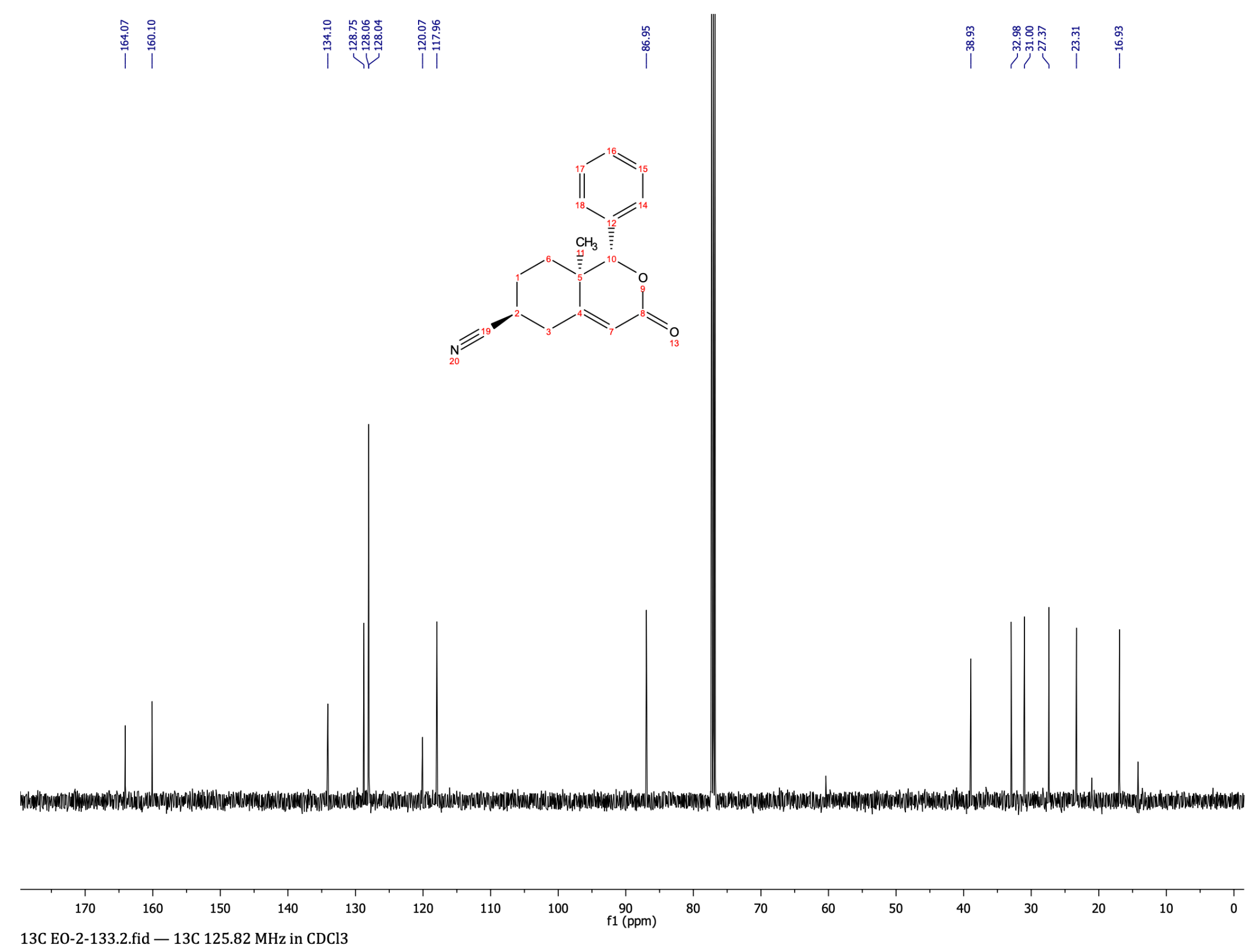

^{1}H NMR (500.30 MHz) and ^{13}C{^{1}H} NMR (125.82 MHz) for Compound **7f** in CDCl3

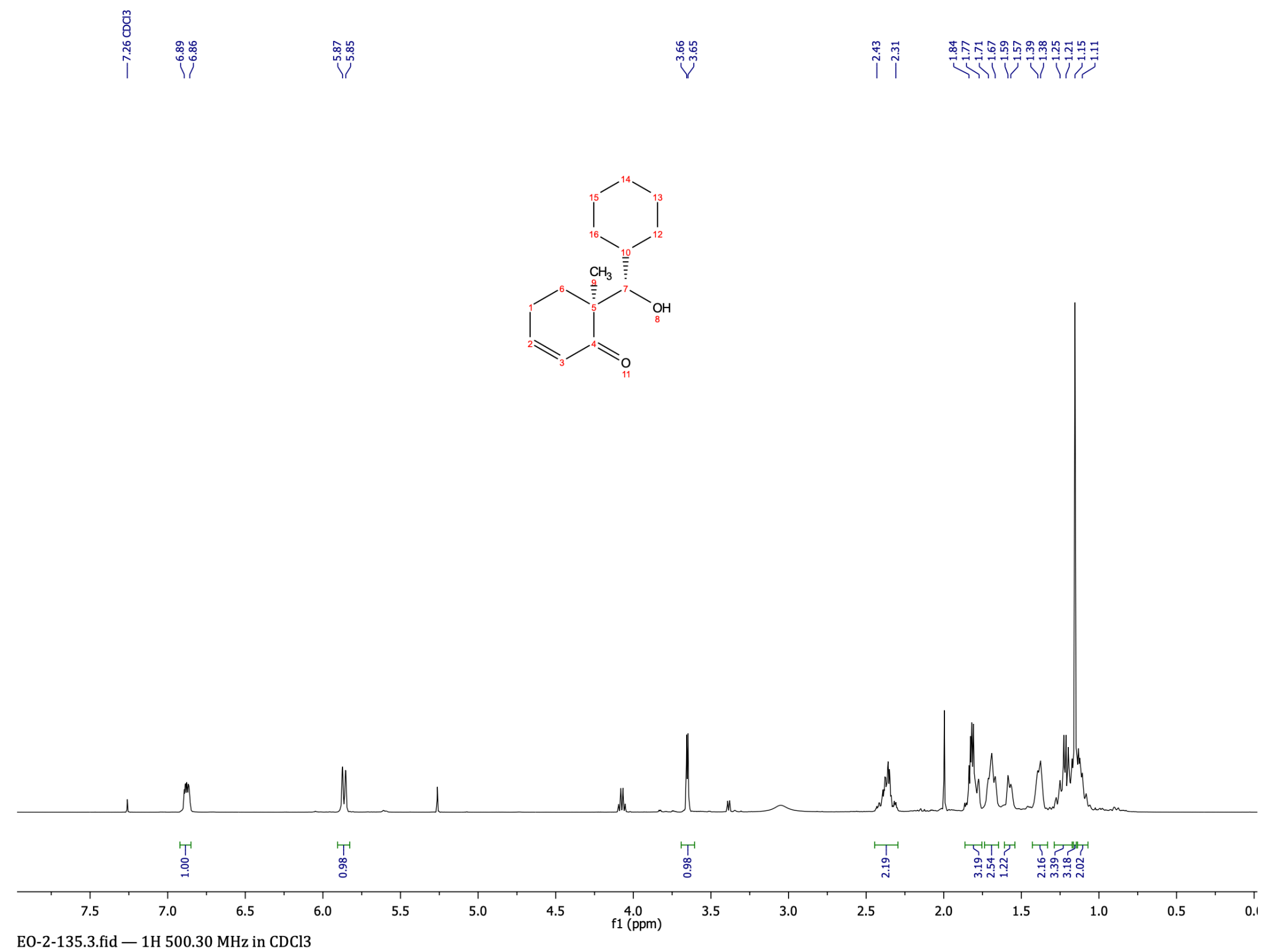

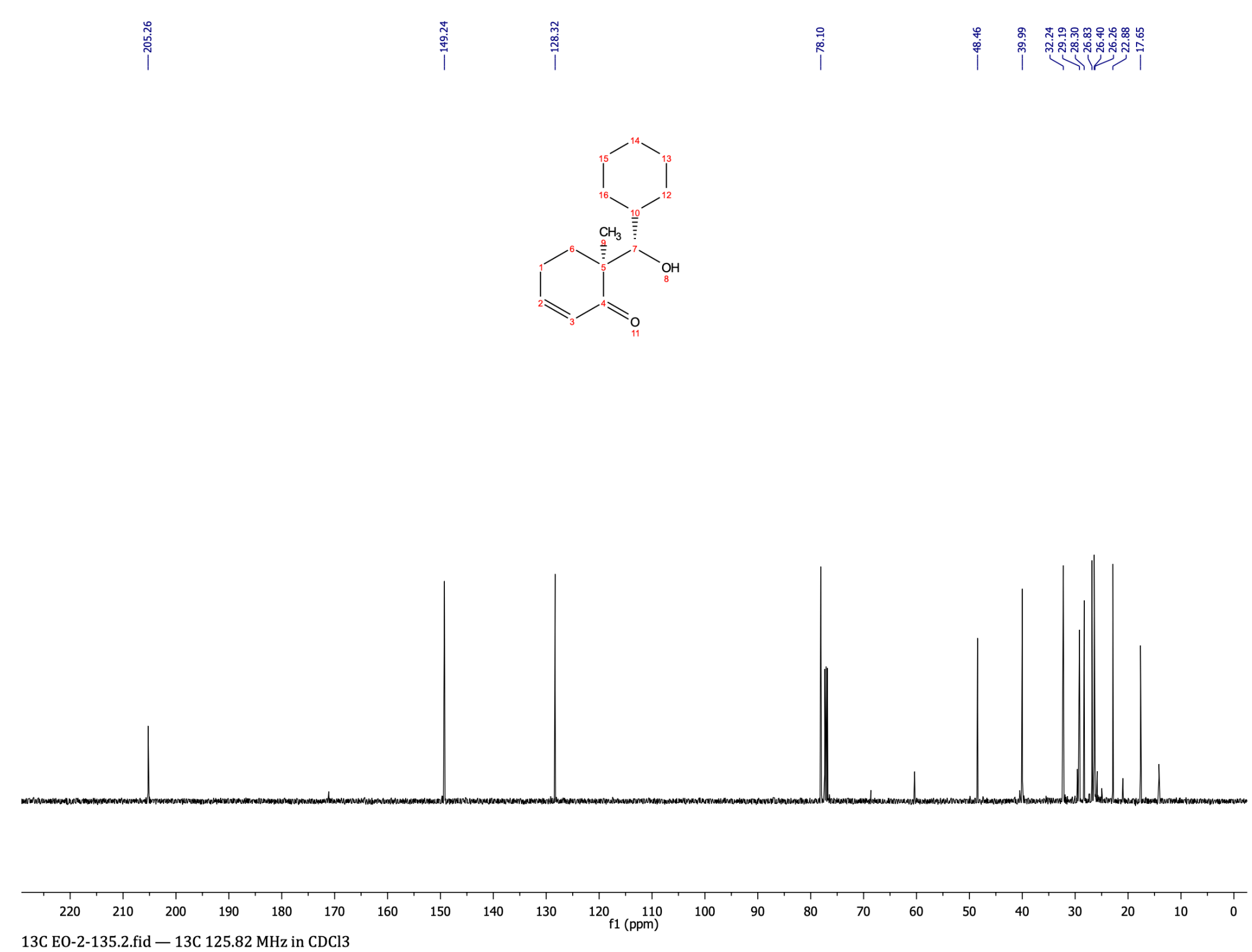

^{1}H NMR (500.30 MHz) and ^{13}C{^{1}H} NMR (125.82 MHz) for Compound **8f** in CDCl3

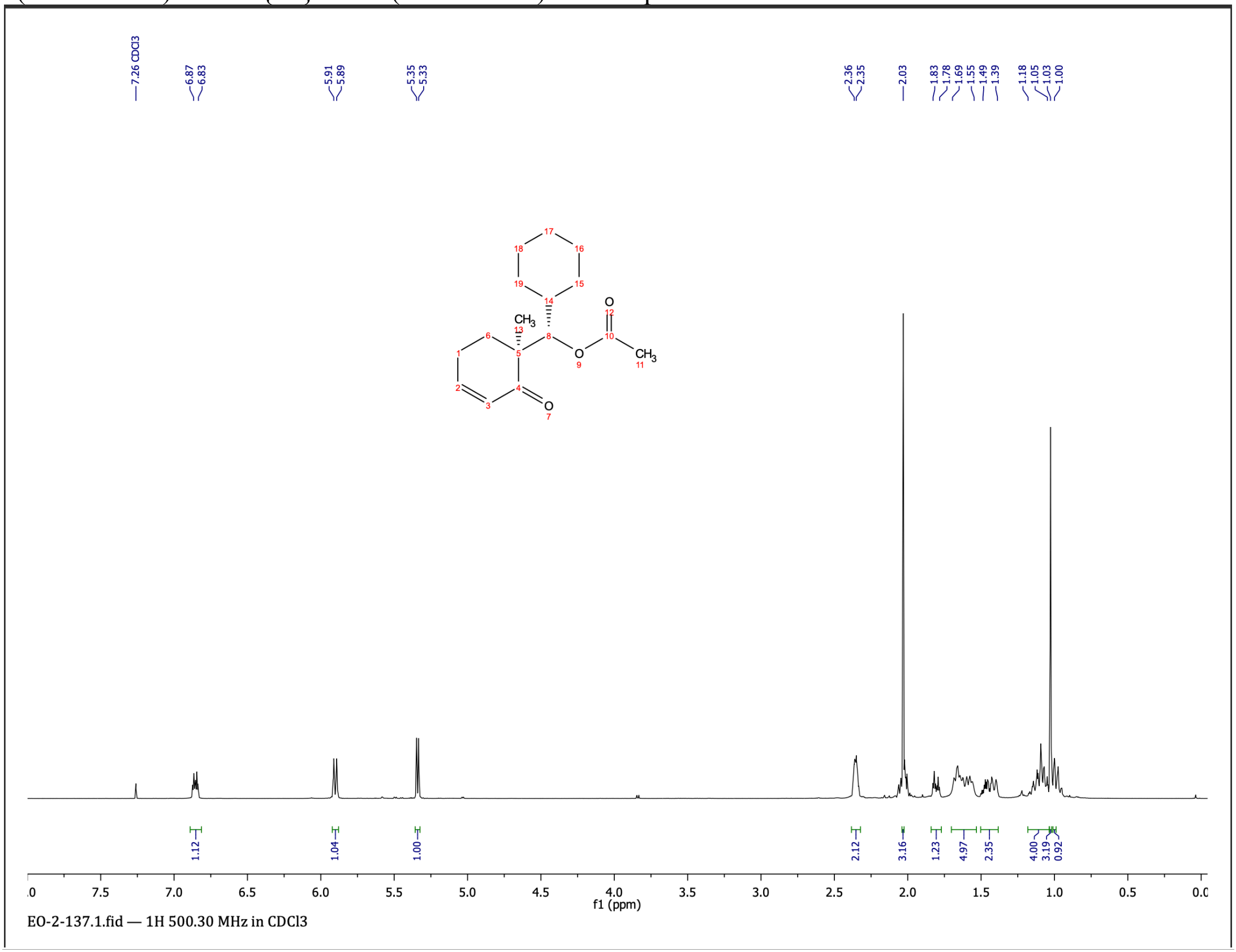

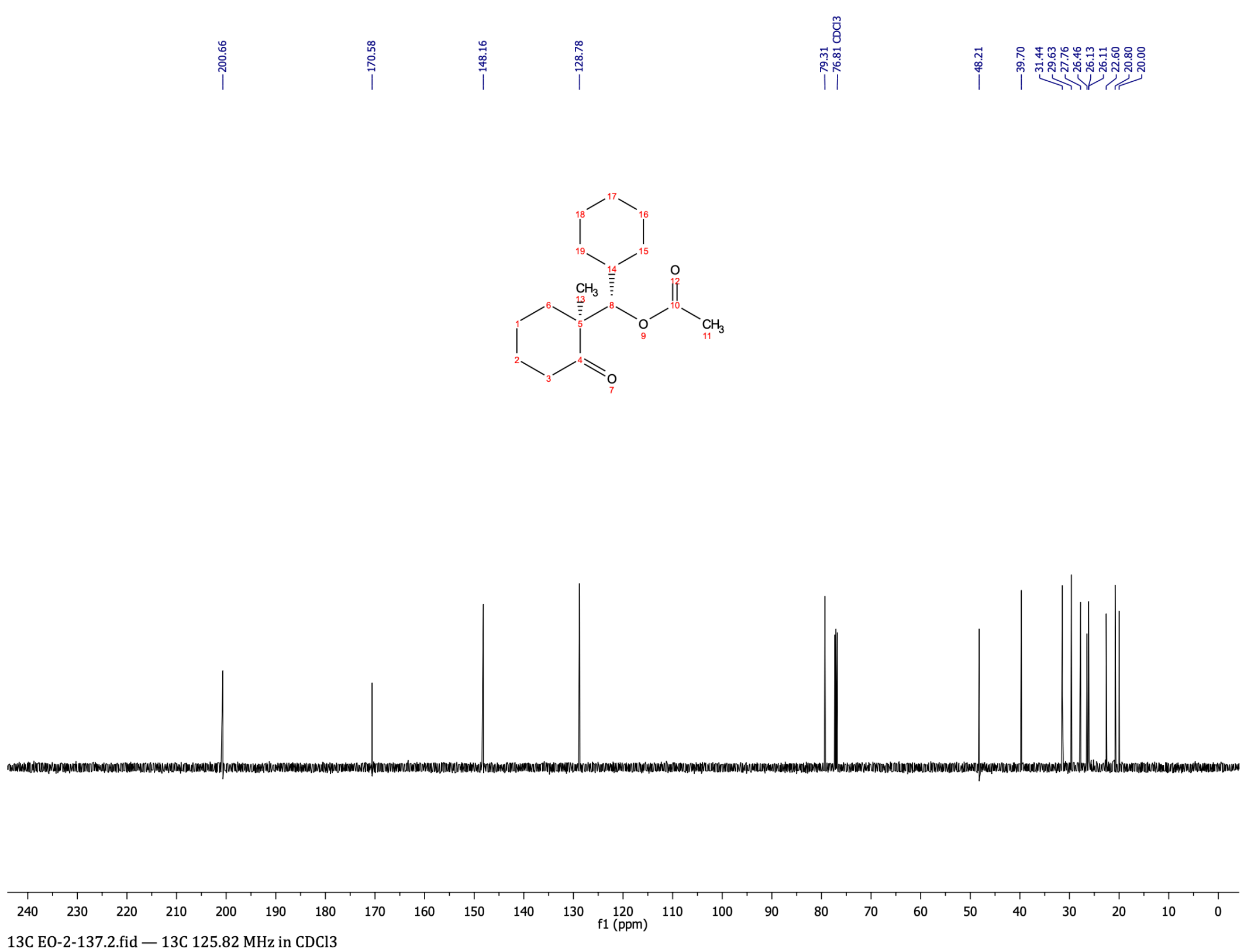

^{1}H NMR (500.30 MHz) and $^{13}C\{^{1}H\}$ NMR (125.82 MHz) for Compound **9f** in CDCl3

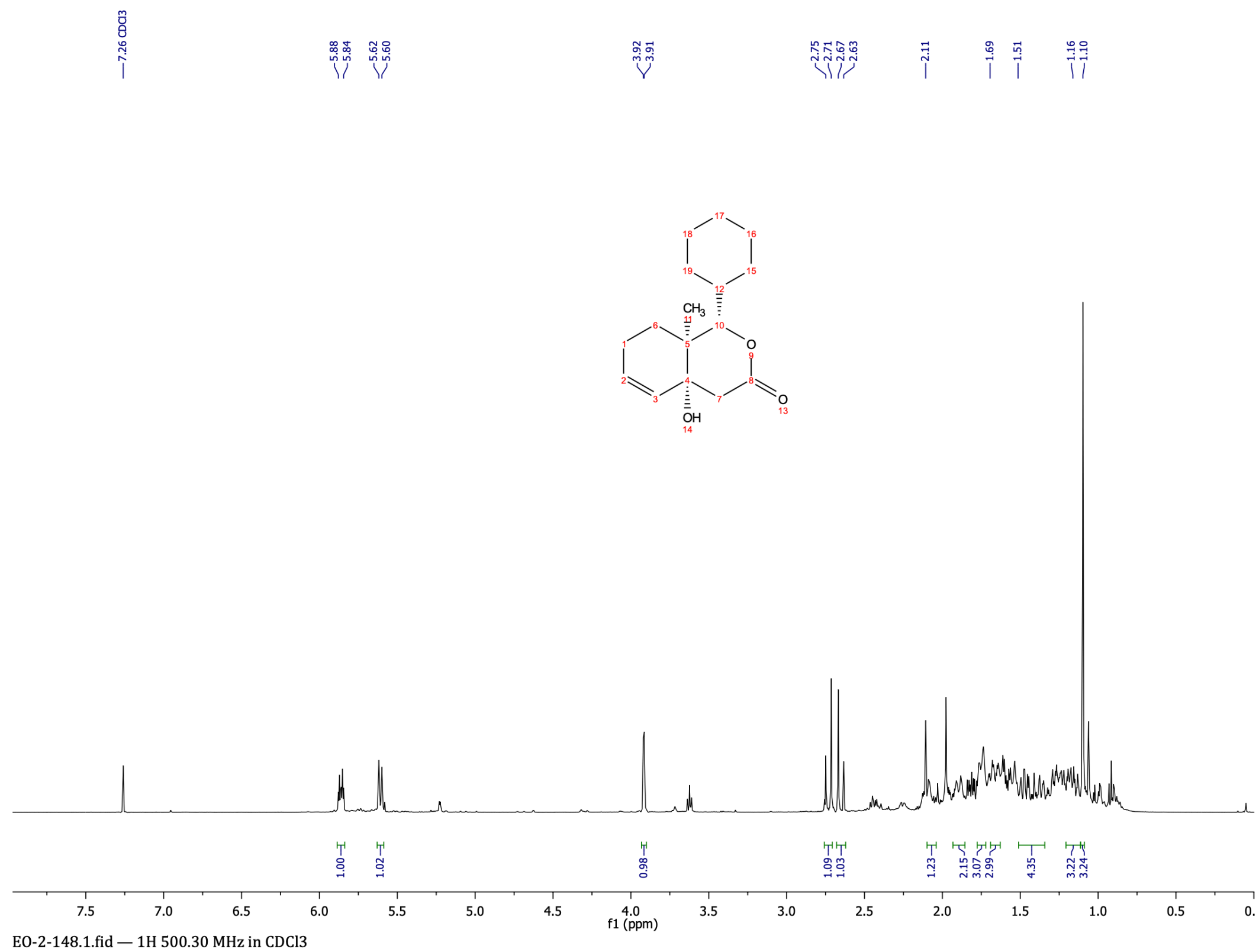

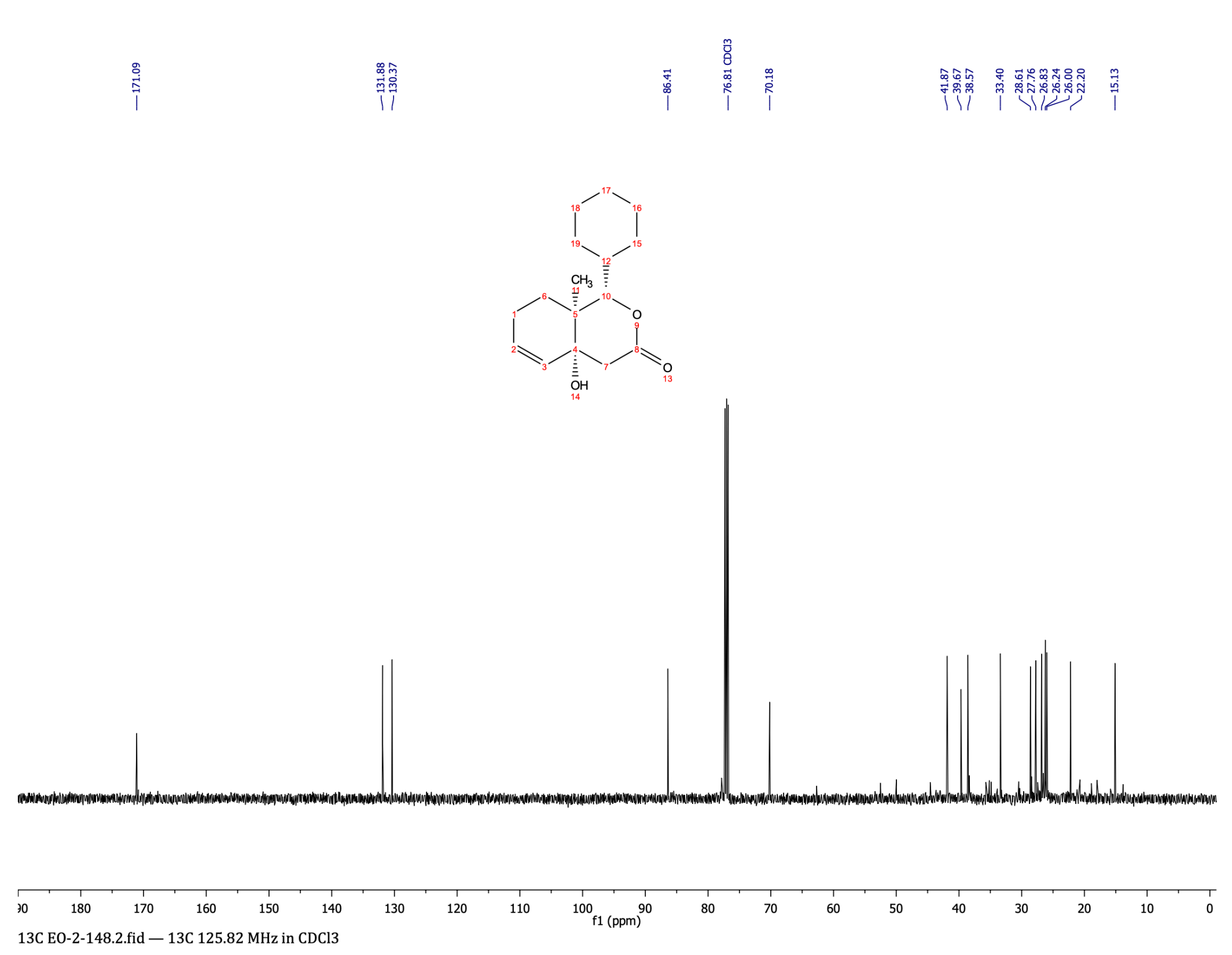

^{1}H NMR (500.30 MHz) and $^{13}C\{^{1}H\}$ NMR (125.82 MHz) for Compound **10f** in CDCl3

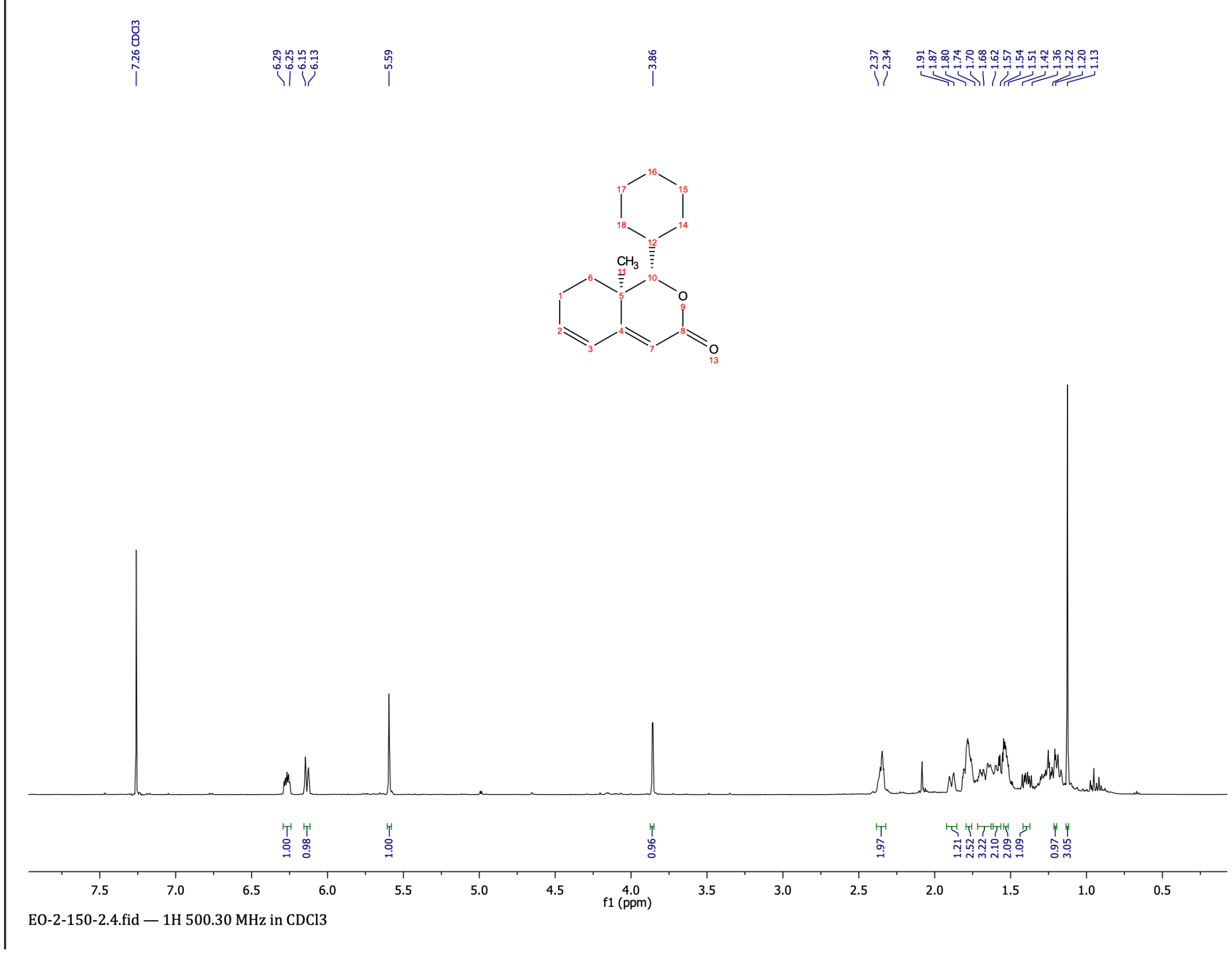

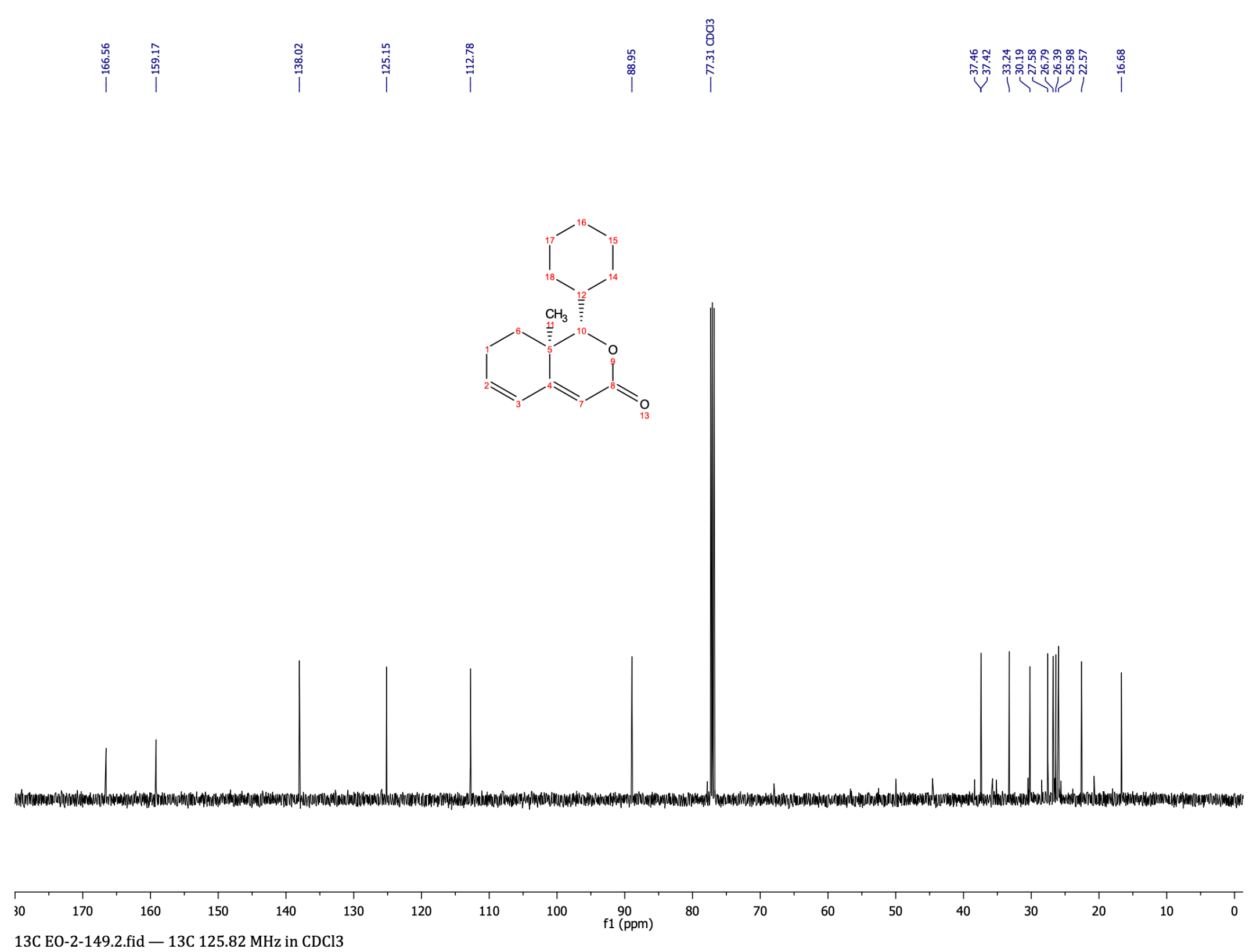

^{1}H NMR (500.30 MHz) and ^{13}C{^{1}H} NMR (125.82 MHz) for Compound **12f** in CDCl3

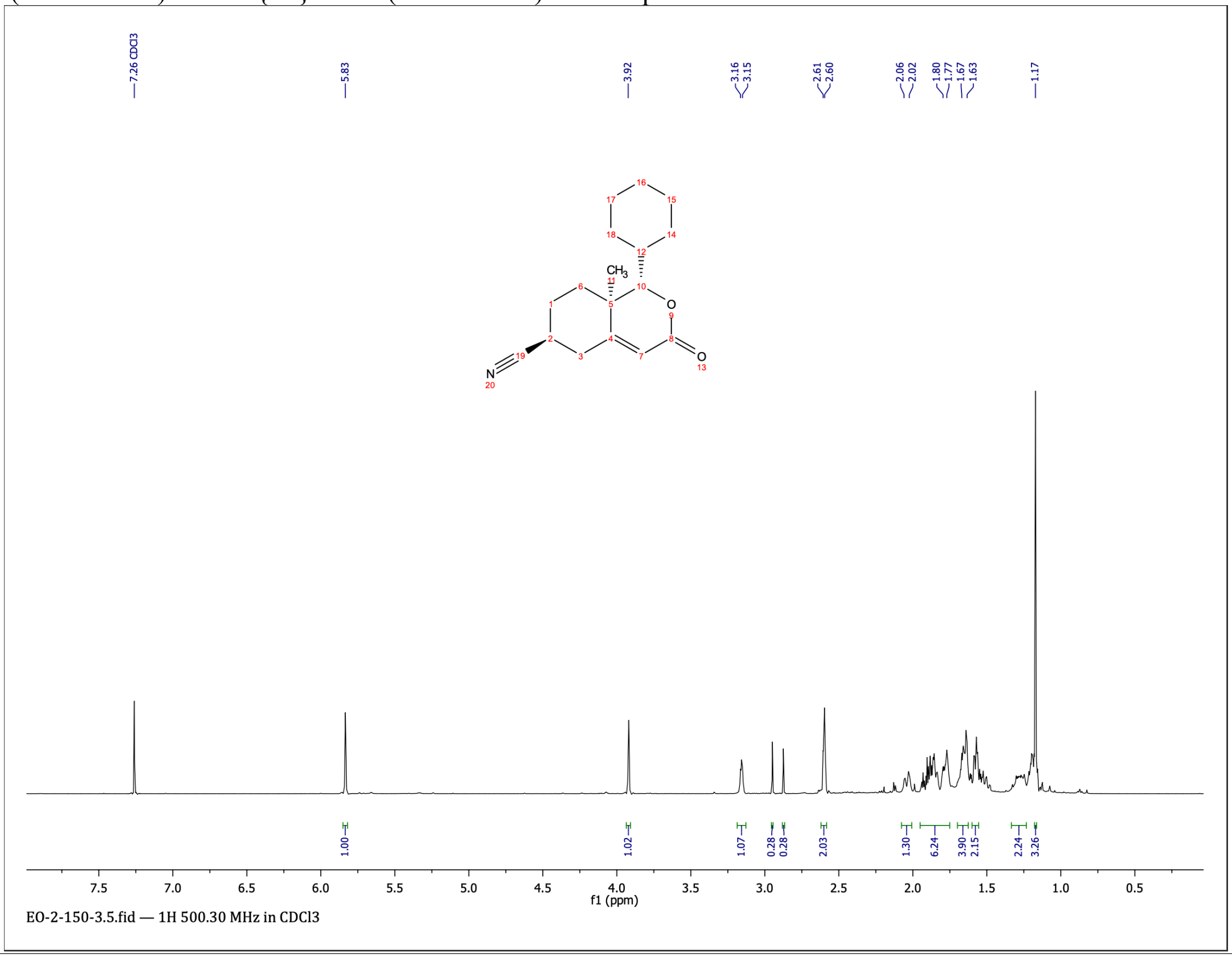

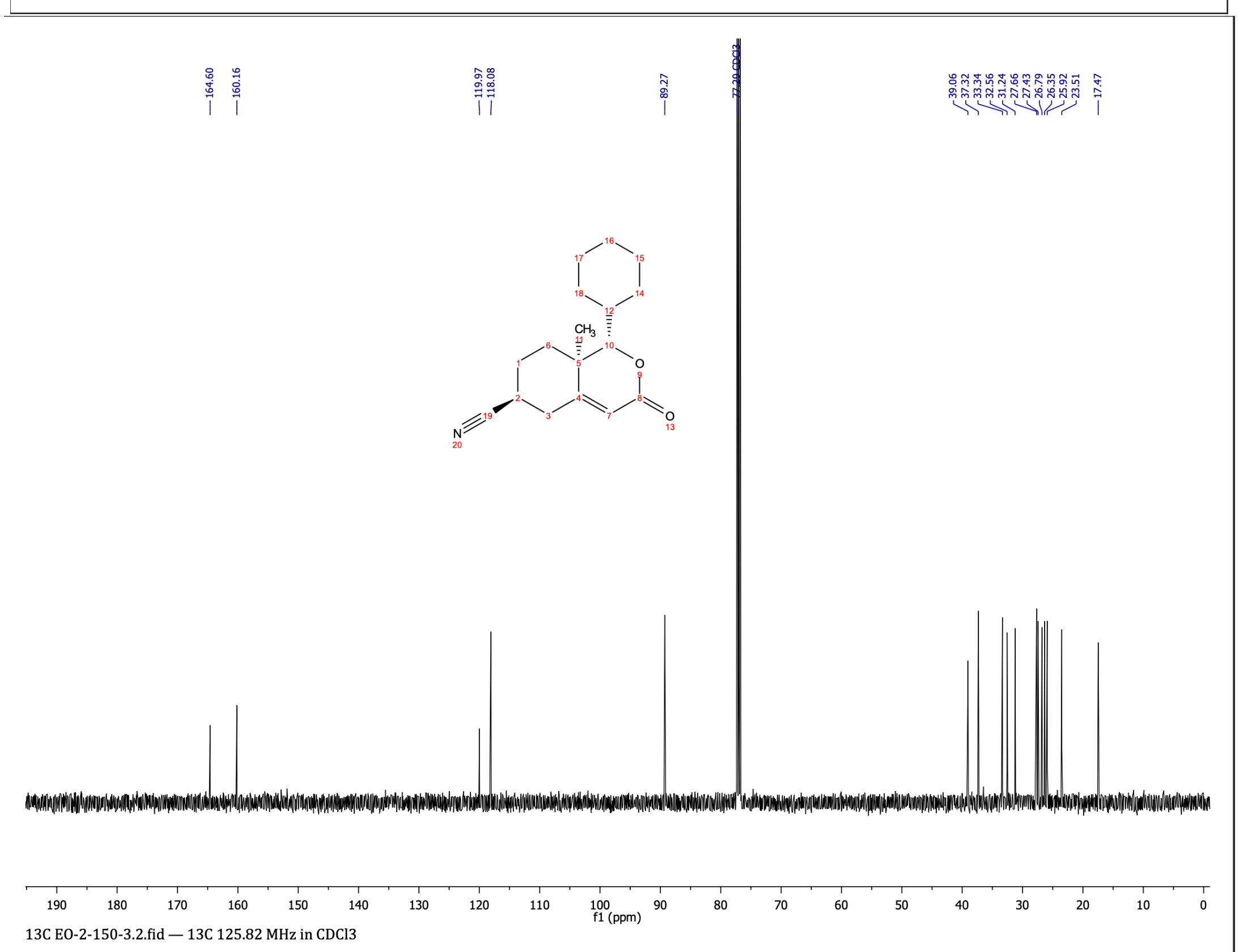

^{1}H NMR (600.21 MHz) and $^{13}C\{^{1}H\}$ NMR (150.94 MHz) for Compound **SI1** in CDCl3

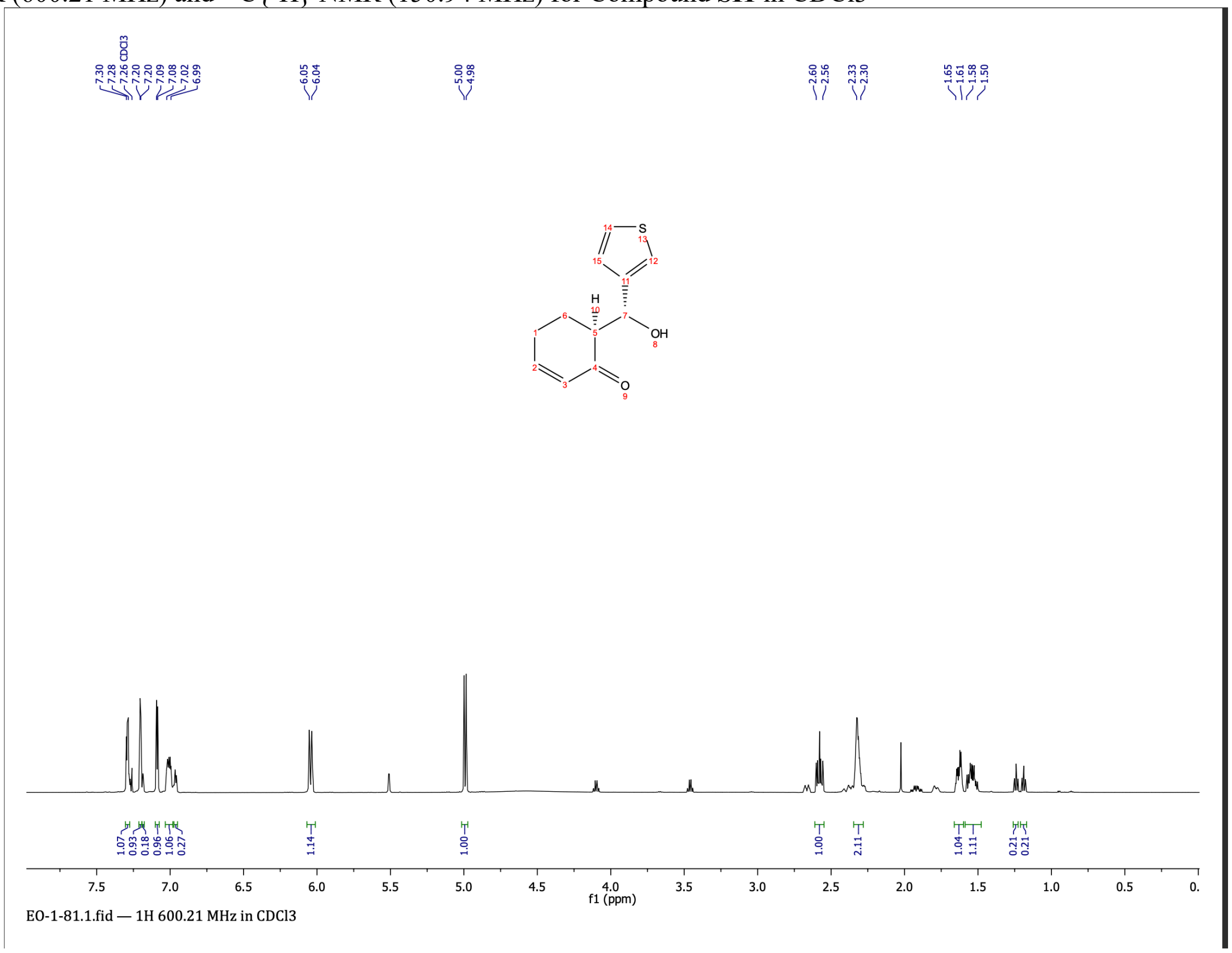

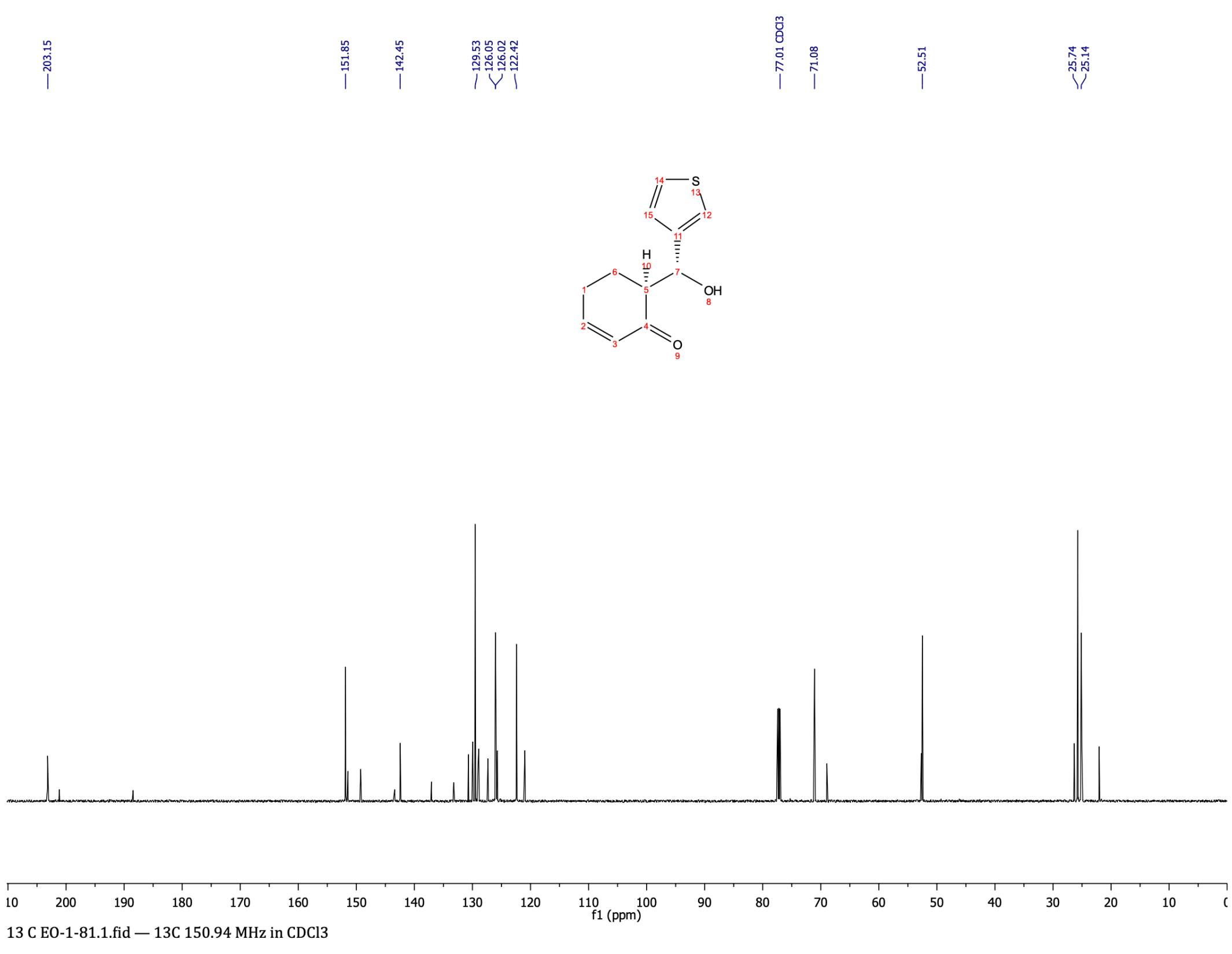

^{1}H NMR (500.30 MHz) and $^{13}C\{^{1}H\}$ NMR (125.82 MHz) for Compound **SI2** in CDCl3

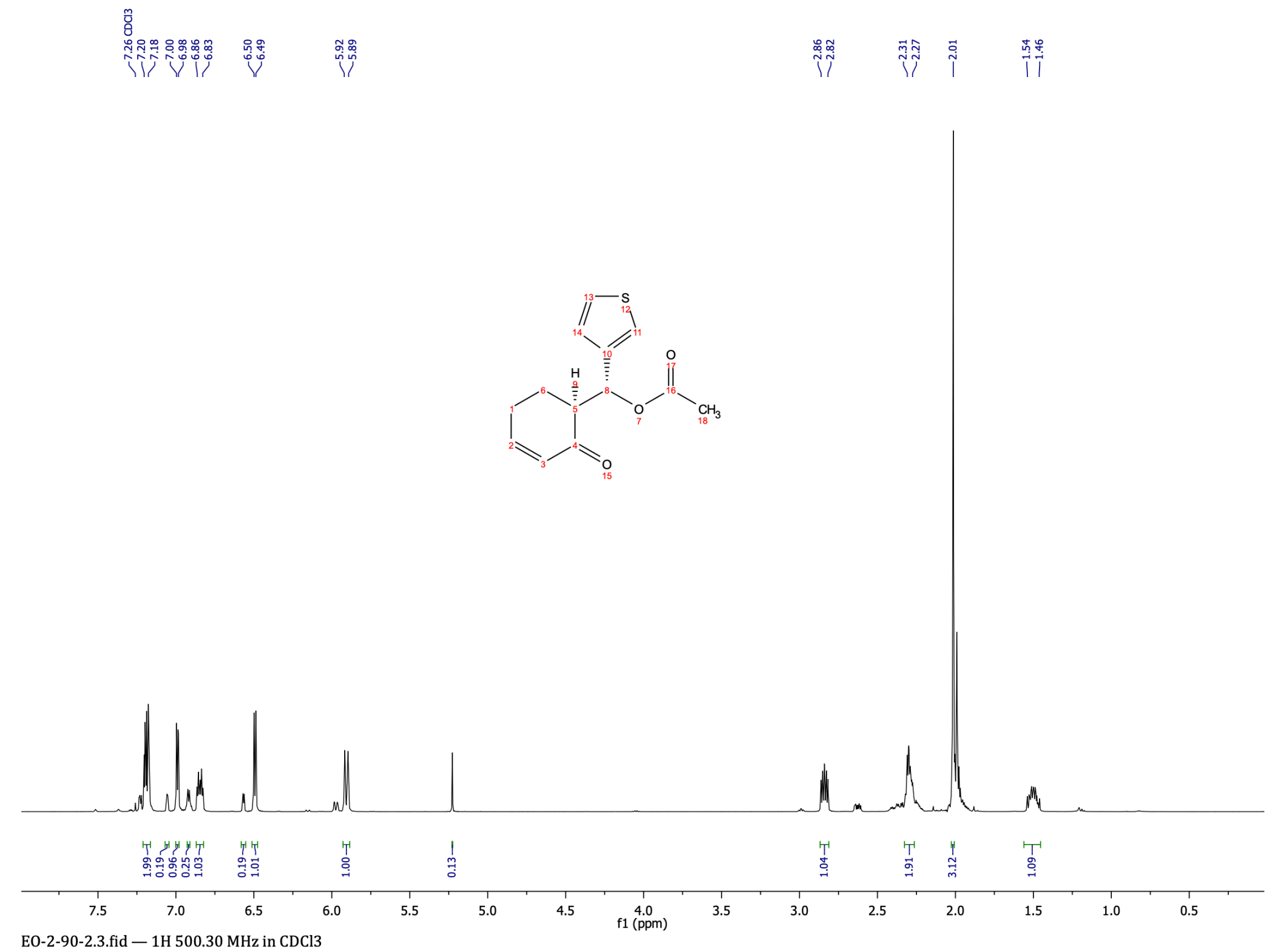

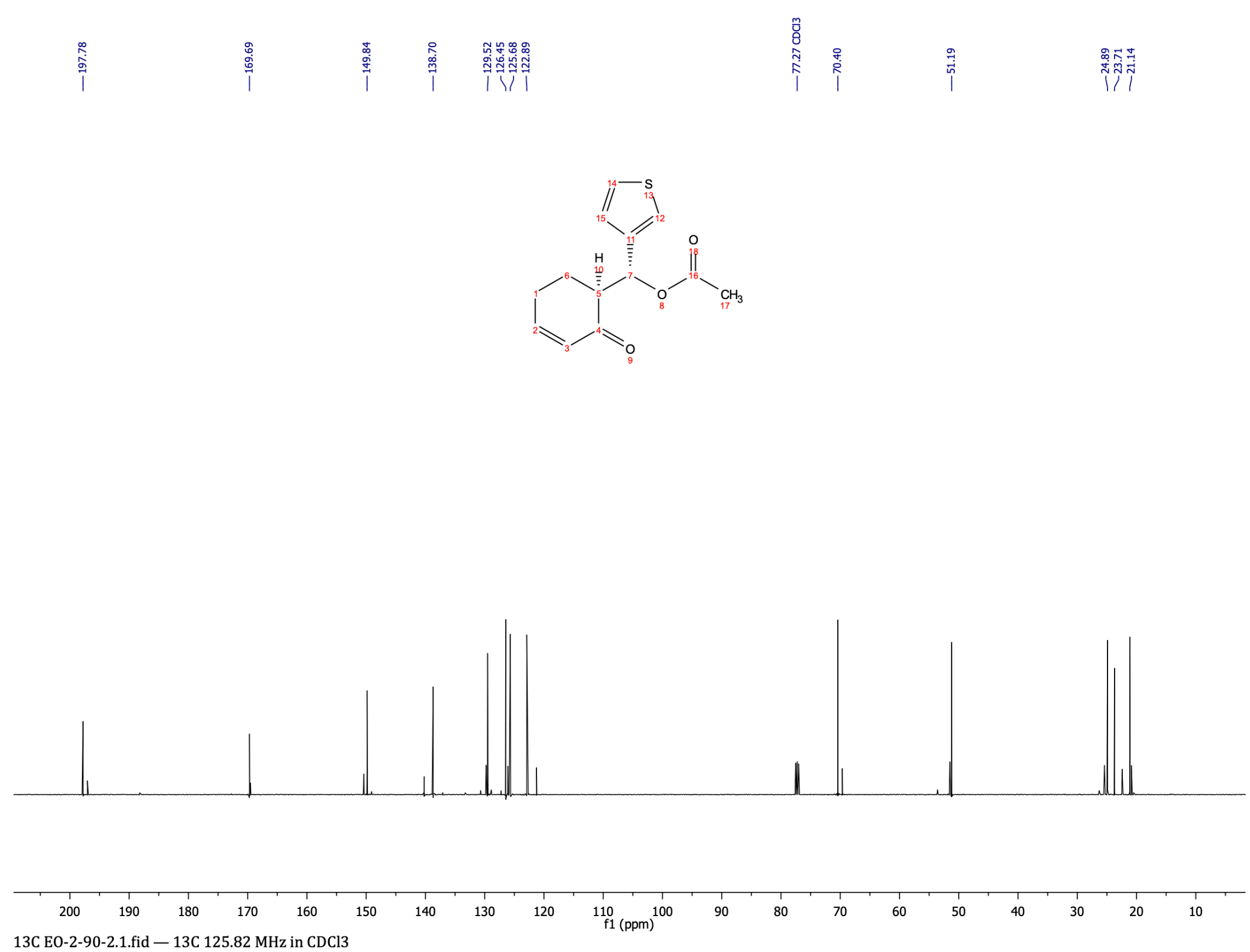

^{1}H NMR (600.21 MHz) and ^{13}C{^{1}H} NMR (150.94 MHz) for Compound **SI3** in CDCl3

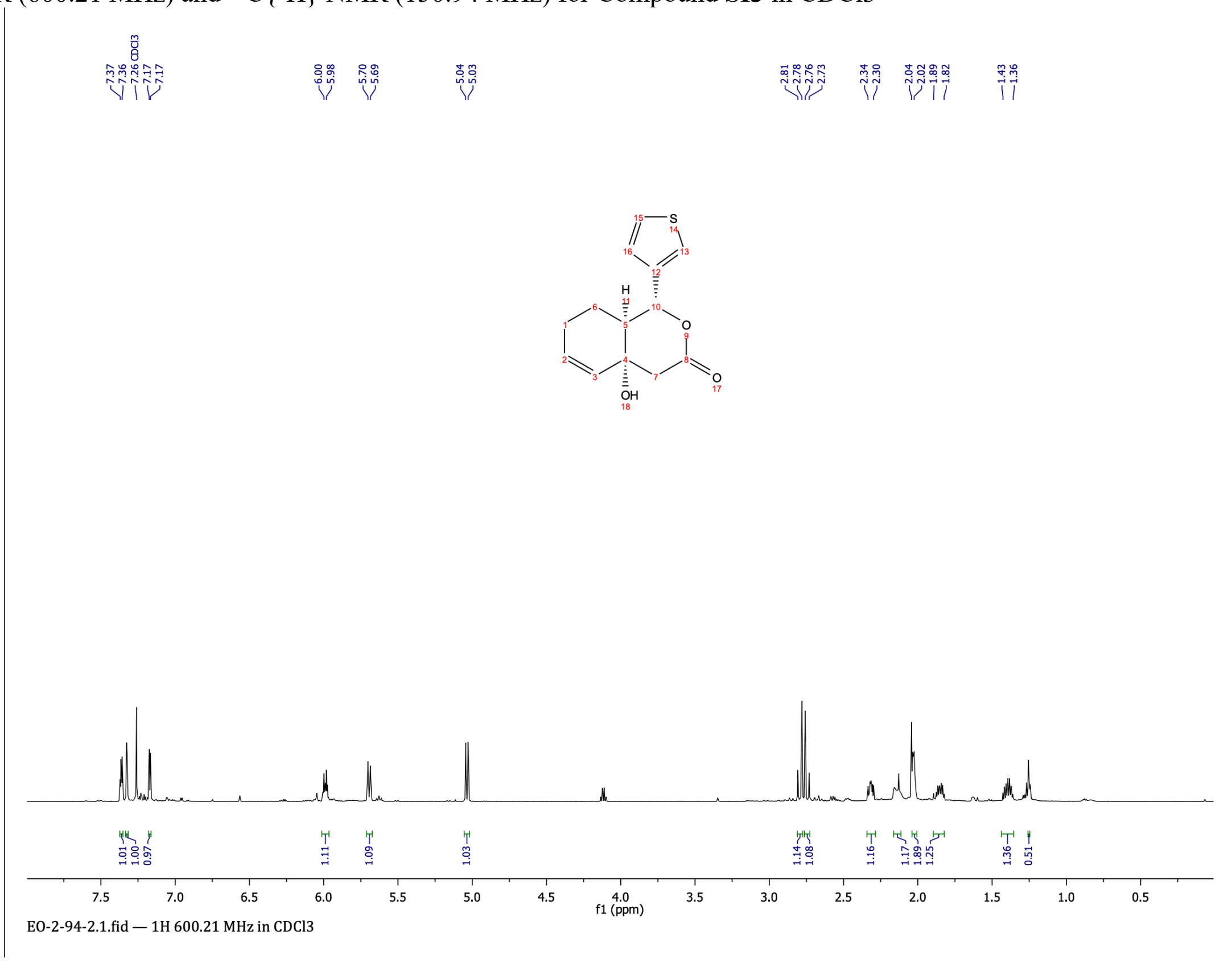

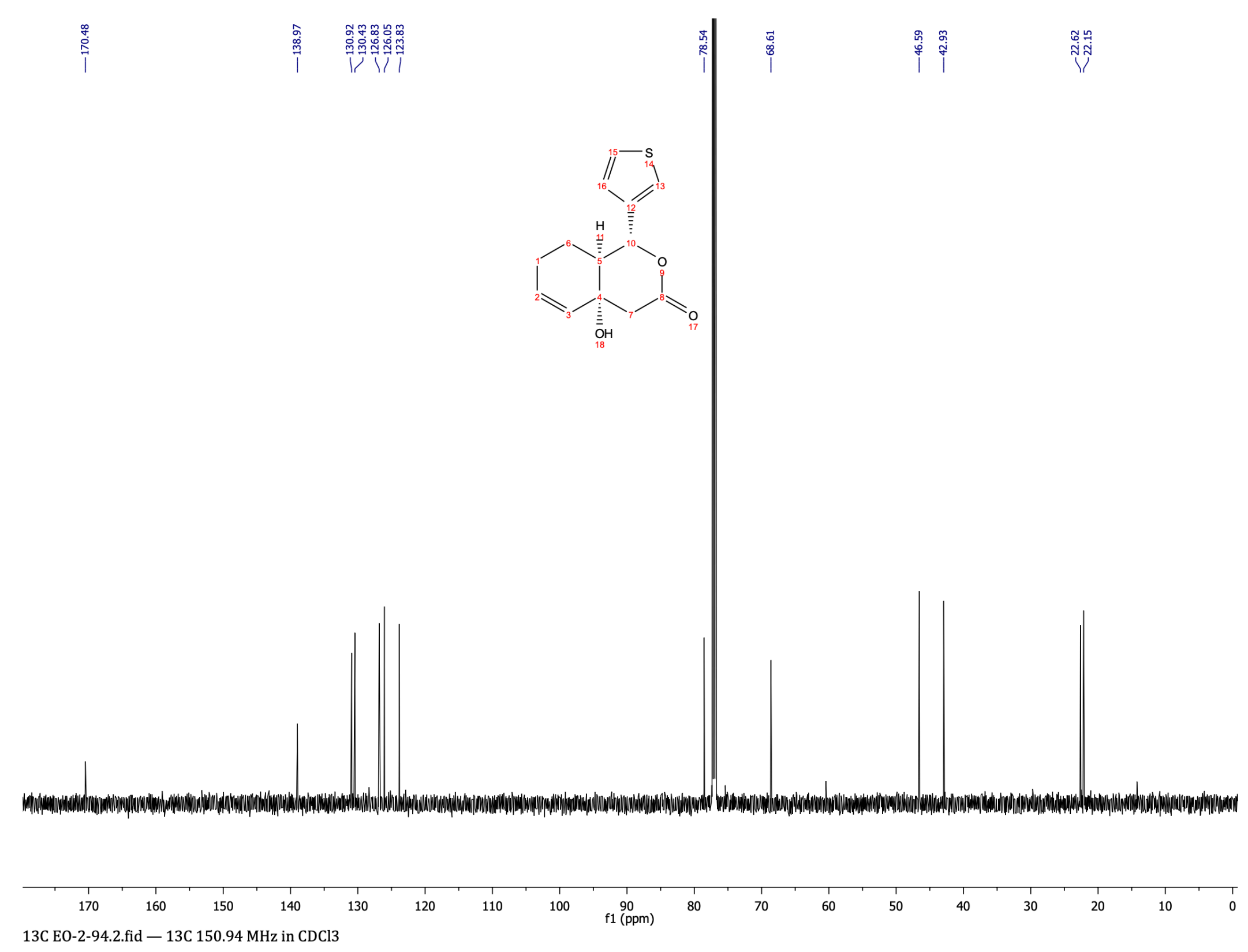

^{1}H NMR (500.30 MHz) and ^{13}C{^{1}H} NMR (125.82 MHz) for Compound **14g** in CDCl3

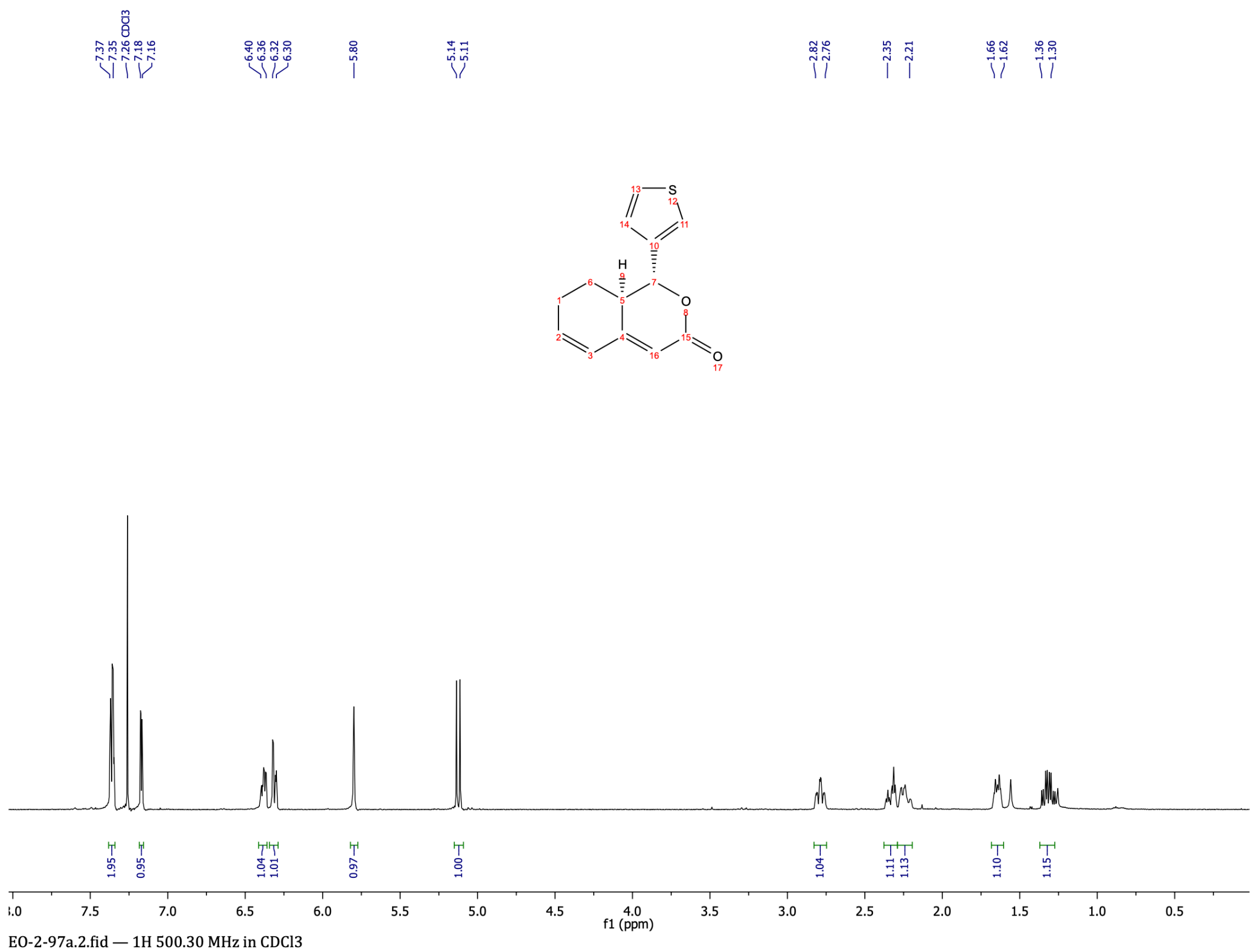

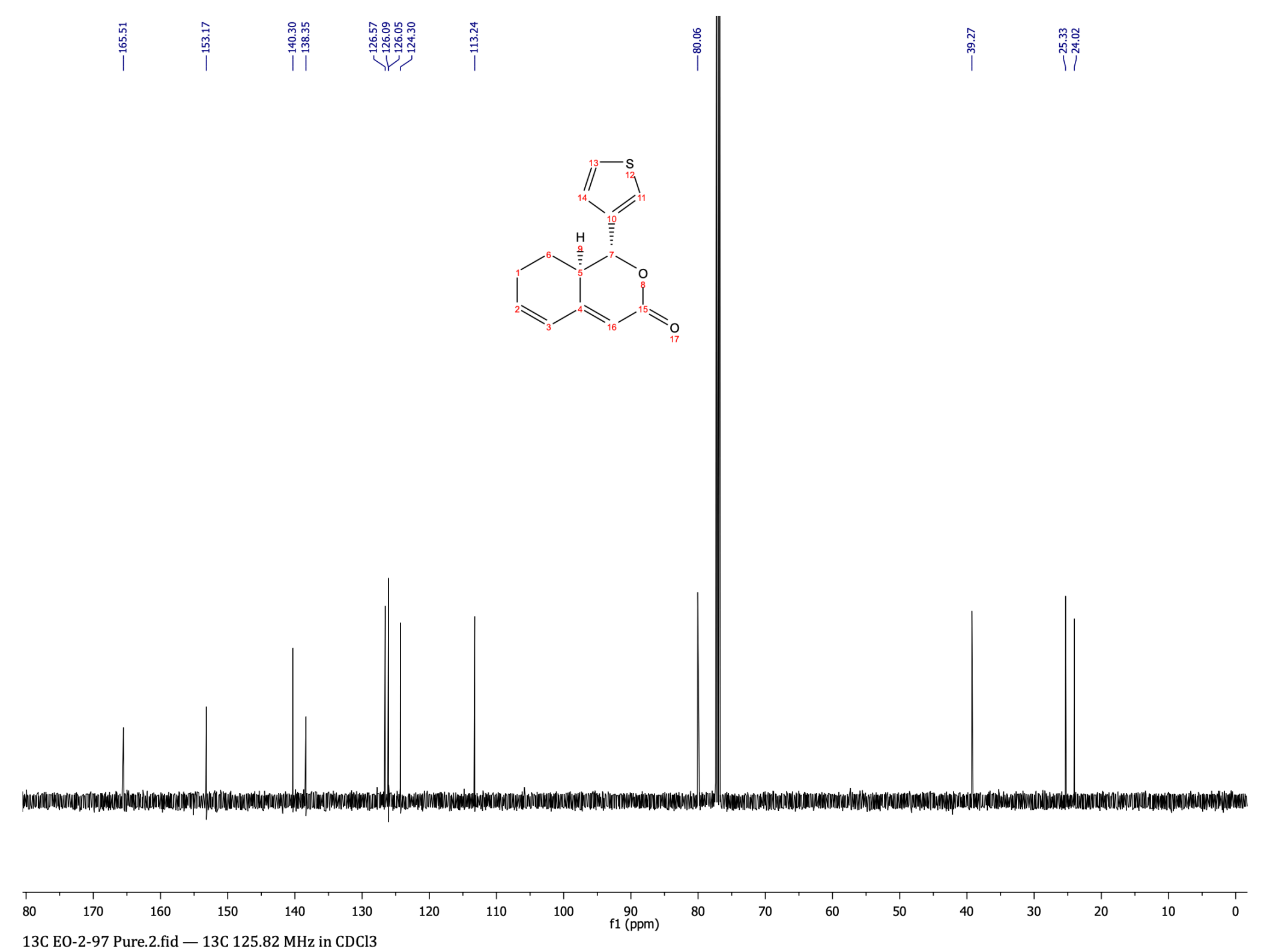

^{1}H NMR (600.21 MHz) and ^{13}C{^{1}H} NMR (150.94 MHz) for Compound **7h** in CDCl3:

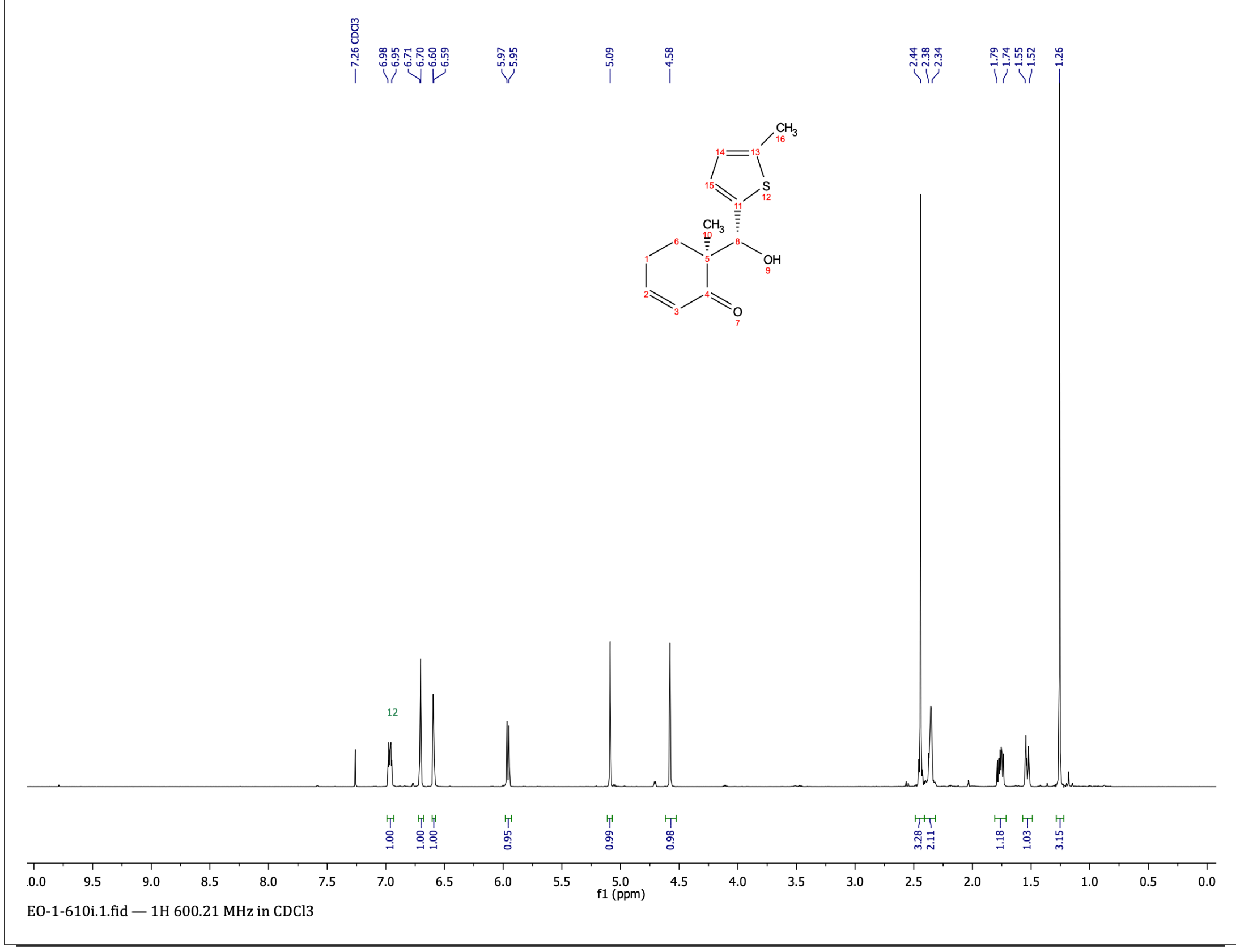

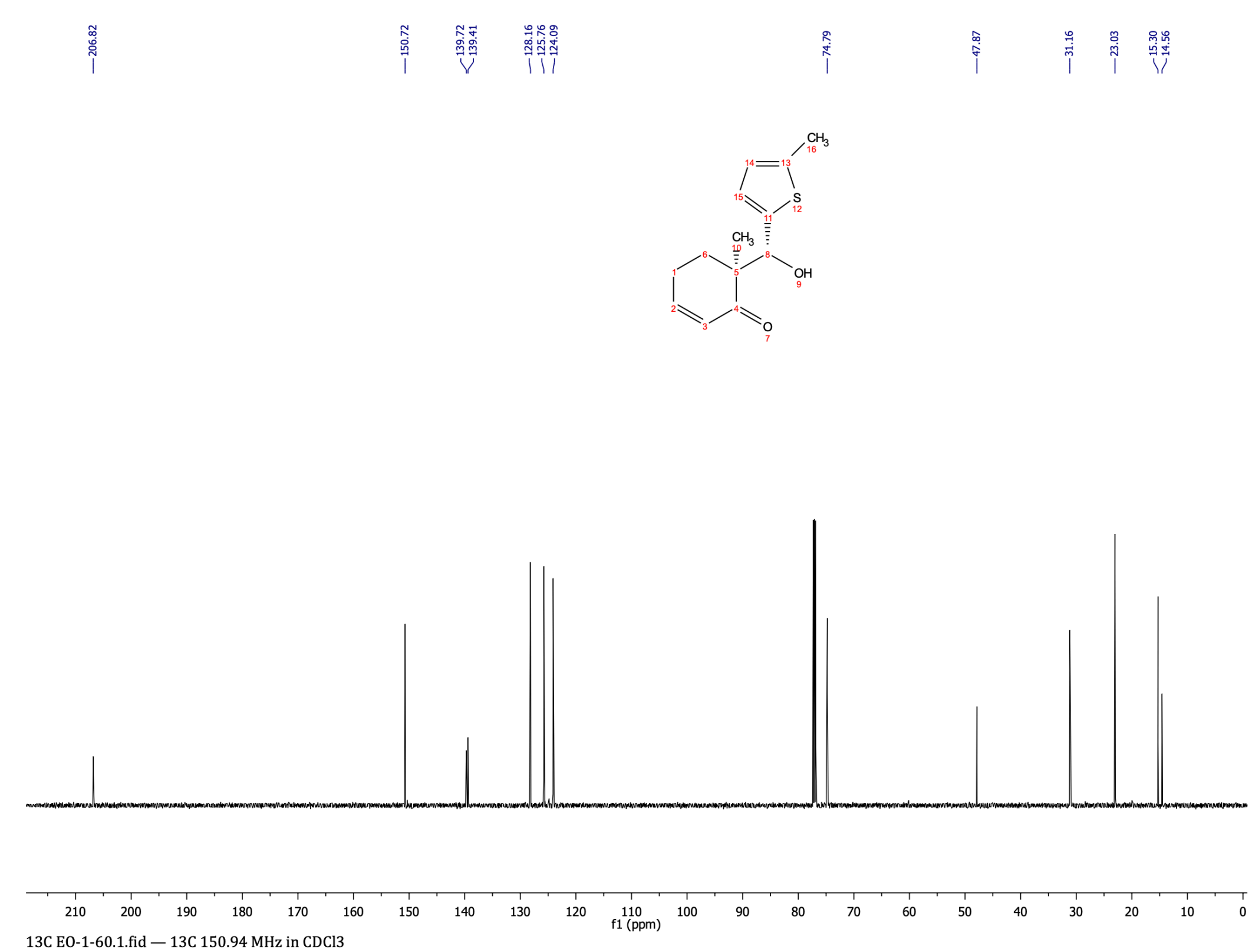

^{1}H NMR (500.30 MHz) and ^{13}C{^{1}H} NMR (125.82 MHz) for Compound **8h** in CDCl3:

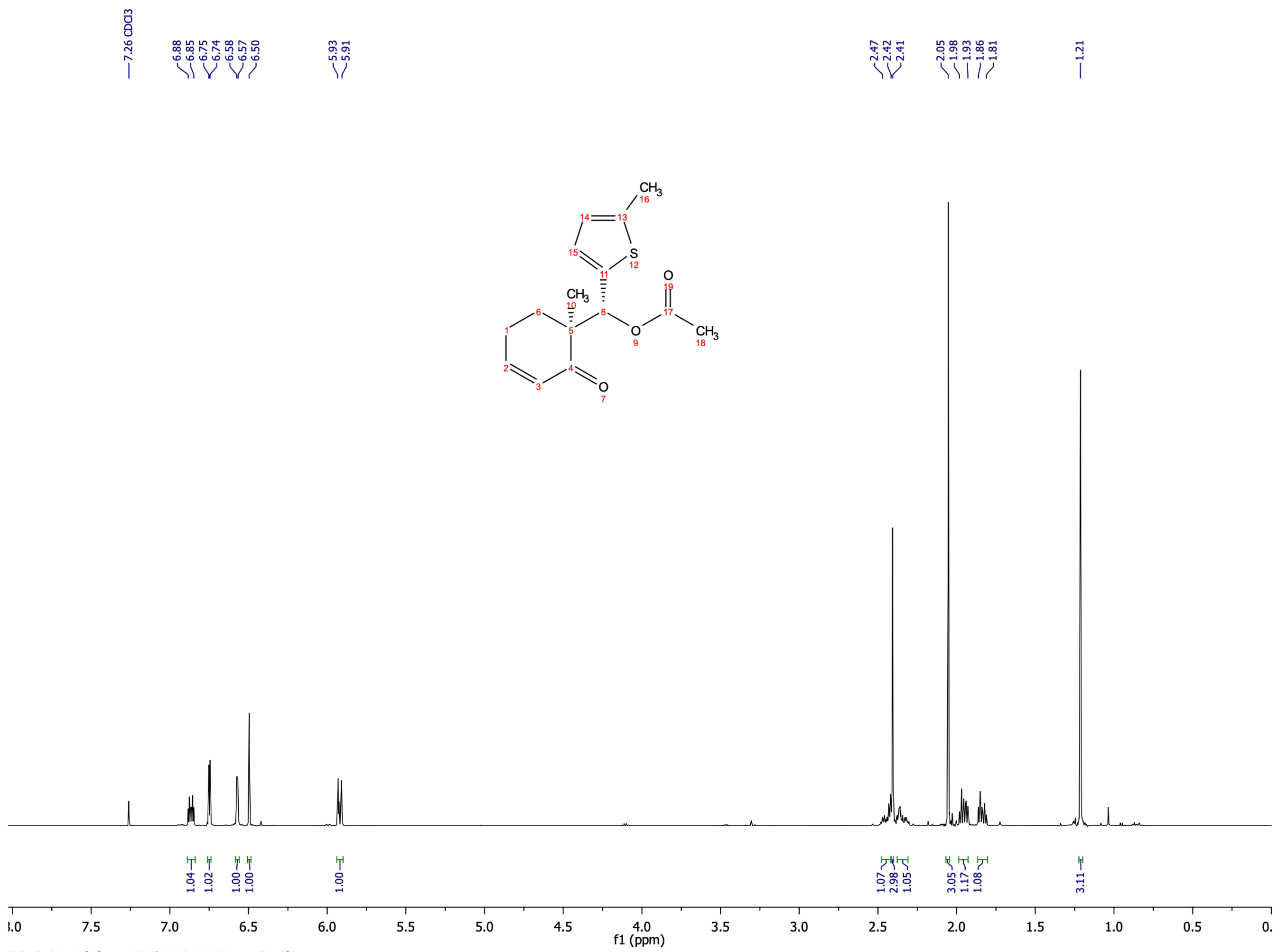

EO-2-89.1.fid — 1H 500.30 MHz in CDCl3

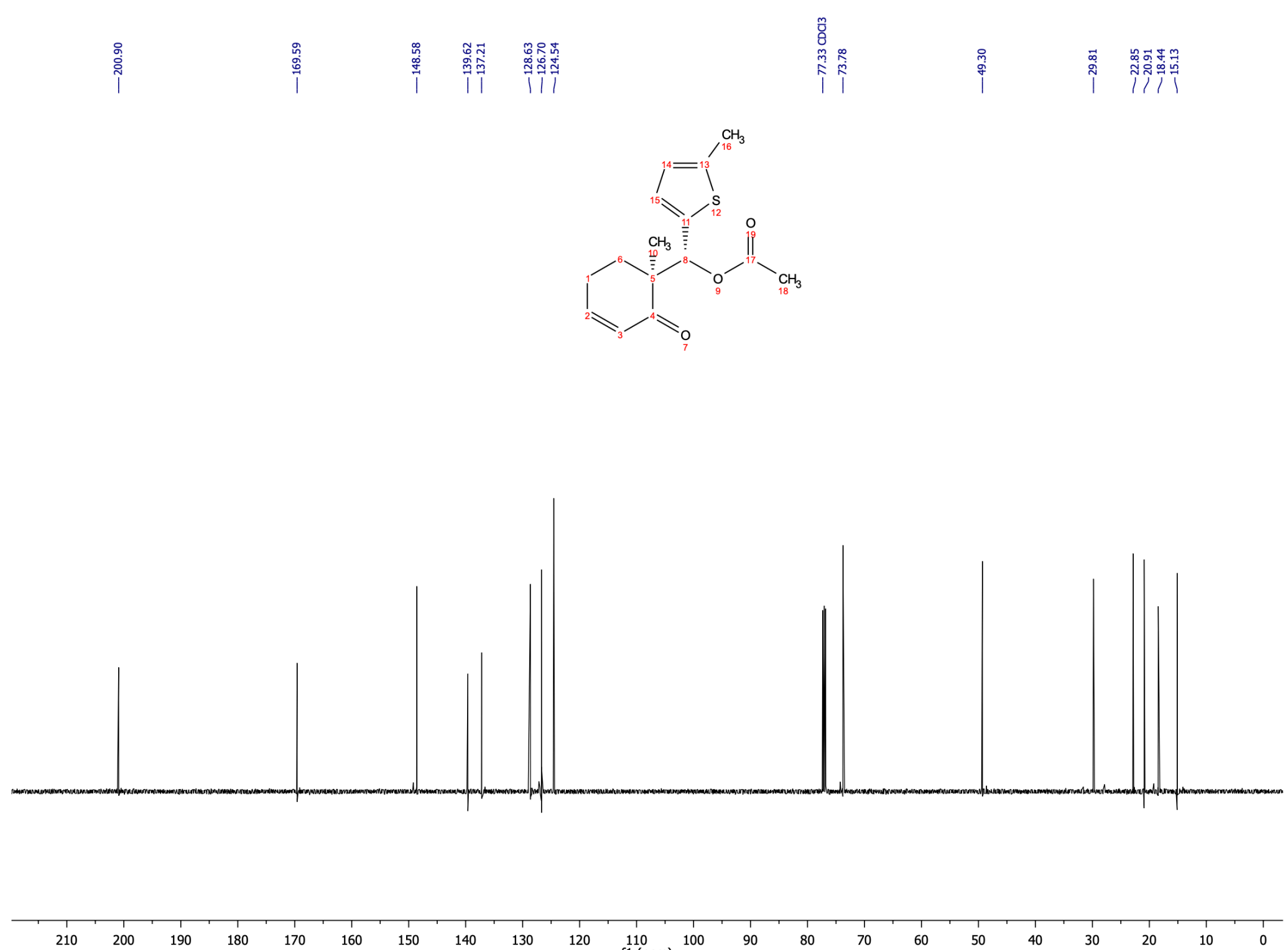

13C EO-2-89.2.fid — 13C 125.82 MHz in CDCl3

^{1}H NMR (500.30 MHz) and ^{13}C{^{1}H} NMR (125.82 MHz) for Compound **9h** in CDCl3:

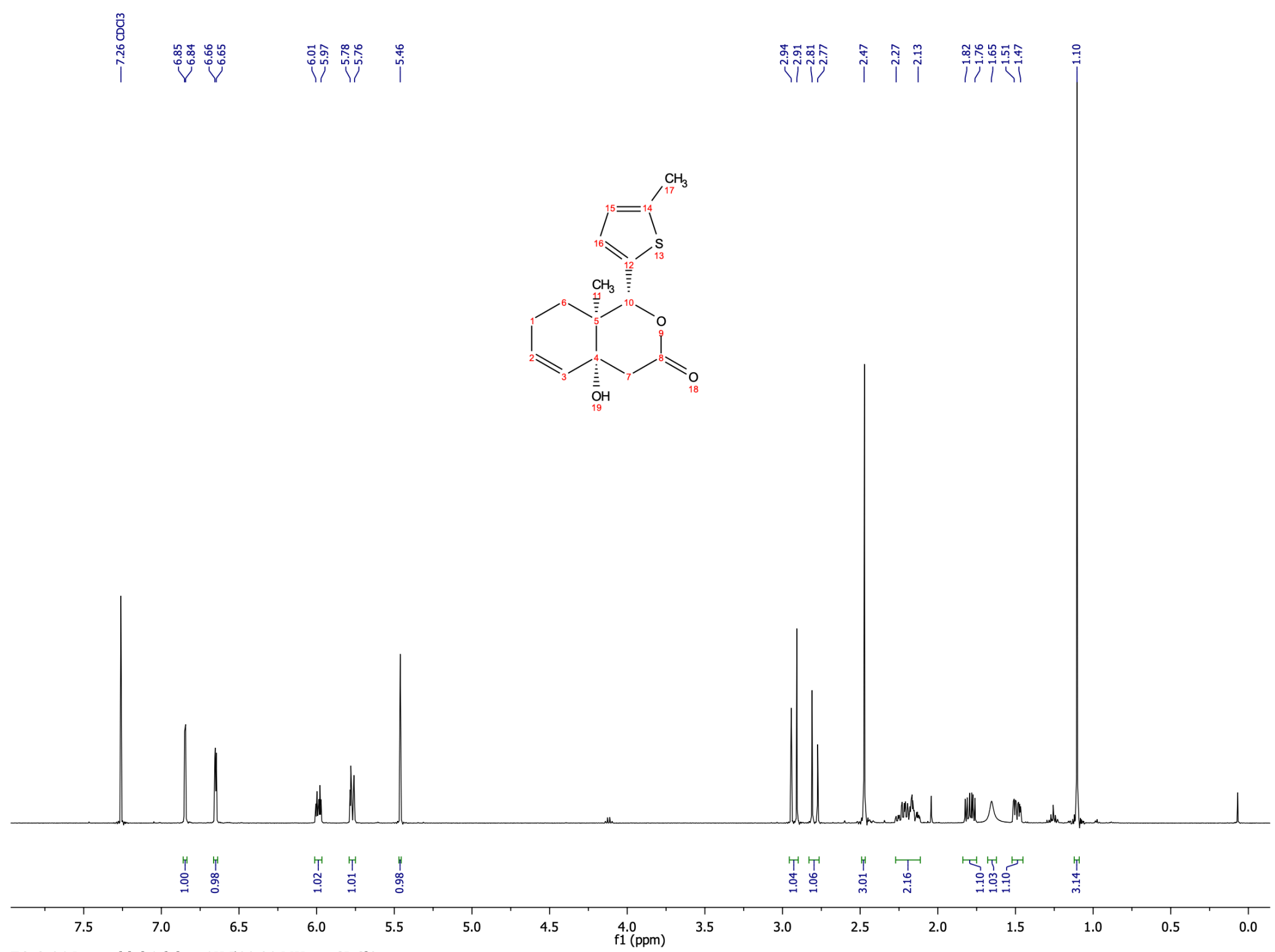

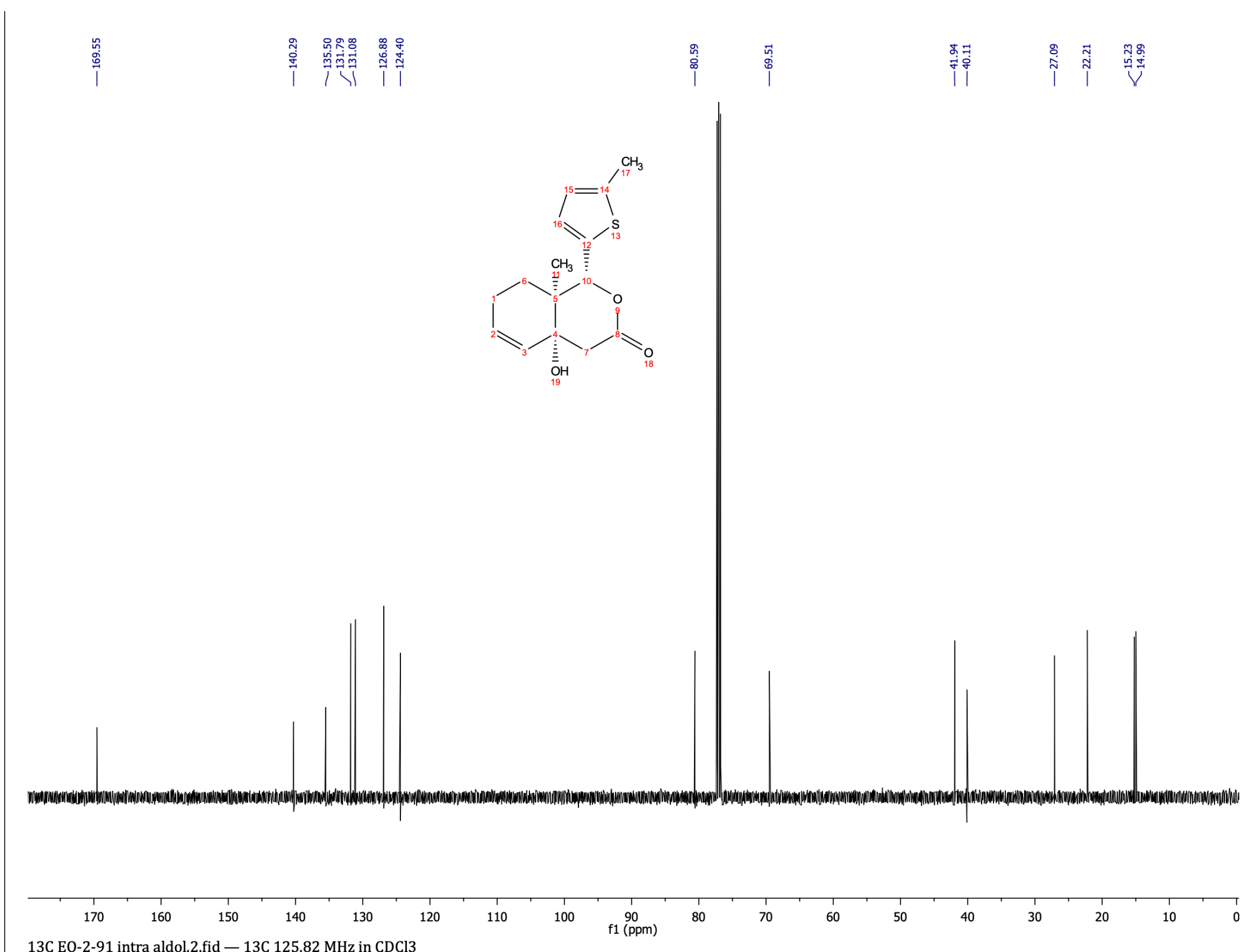

^{1}H NMR (500.30 MHz) and ^{13}C{^{1}H} NMR (125.82 MHz) for Compound **10h** in CDCl3:

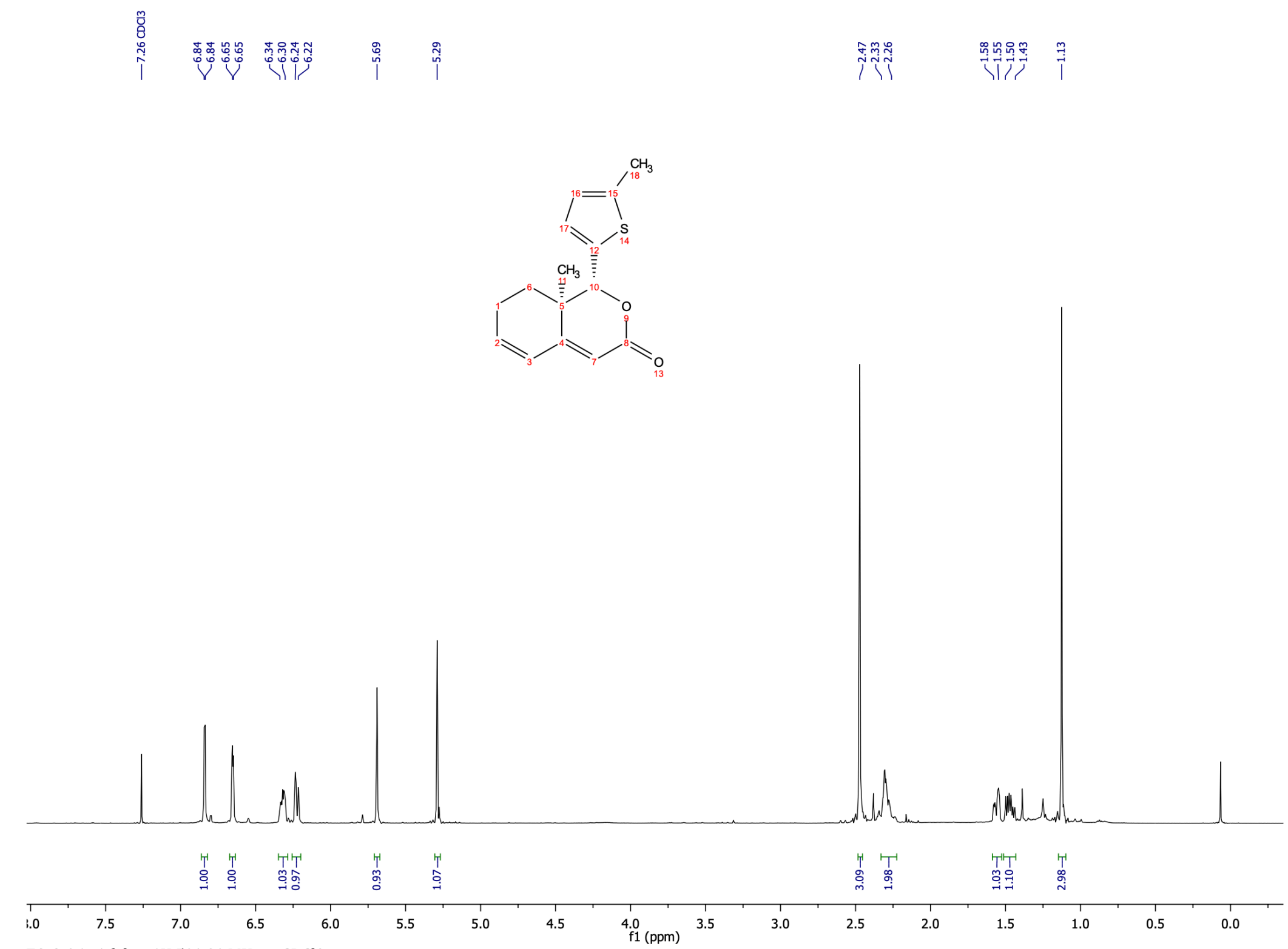

EO-2-96a.1.fid — 1H 500.30 MHz in CDCl3

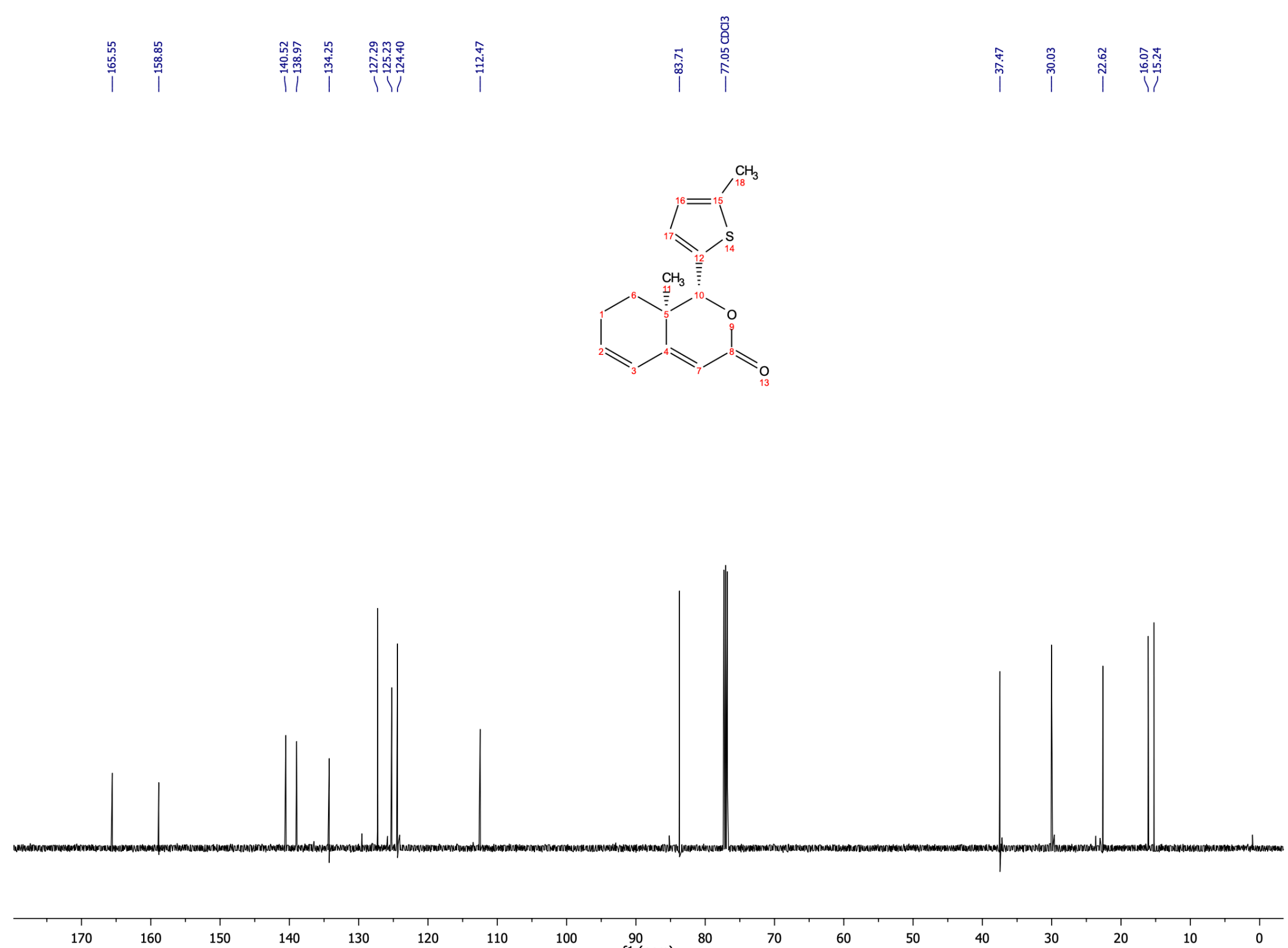

13C EO-2-96.1.fid — 13C 125.82 MHz in CDCl3

^{1}H NMR (500.30 MHz) and ^{13}C{^{1}H} NMR (125.82 MHz) for Compound **15h** in CDCl3:

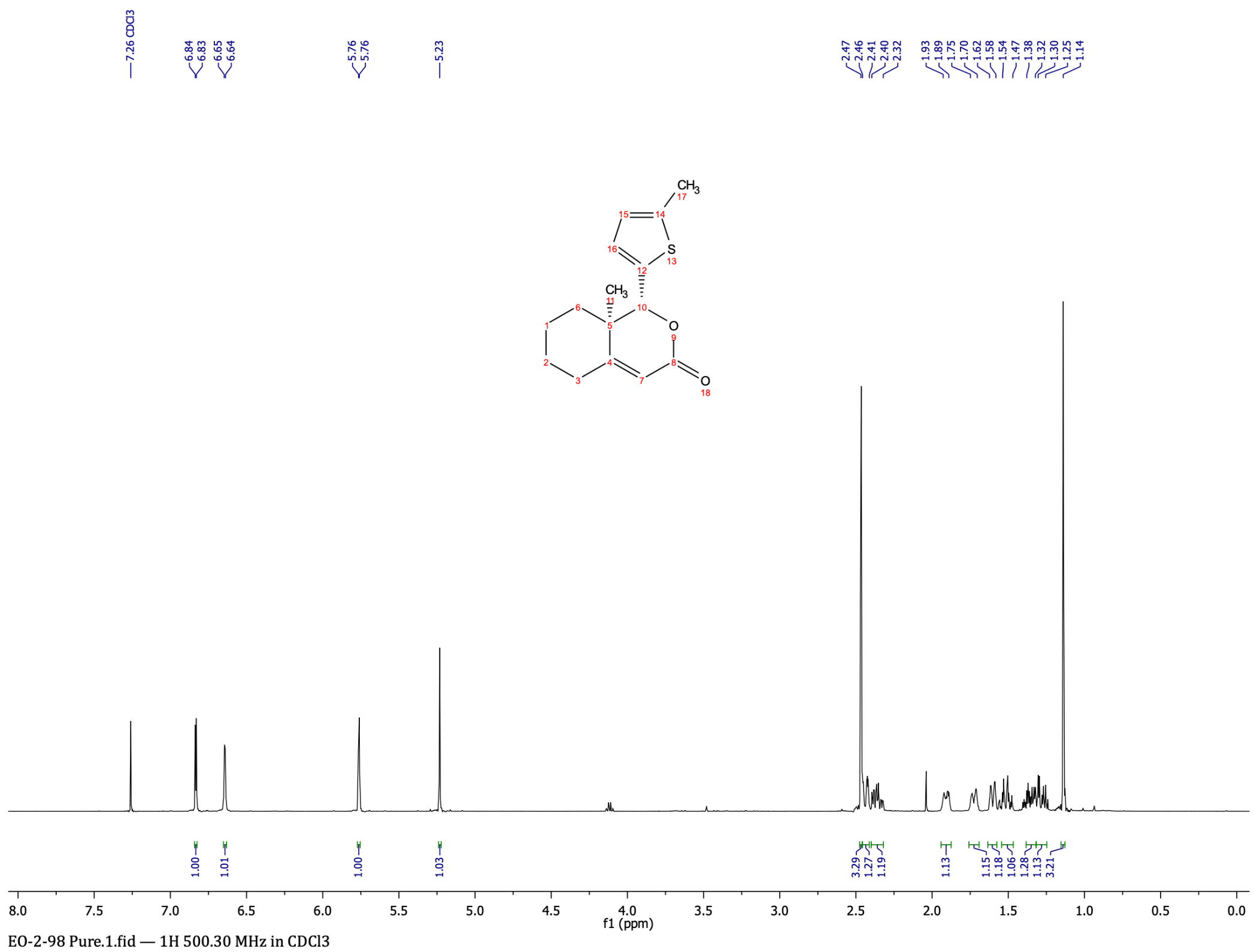

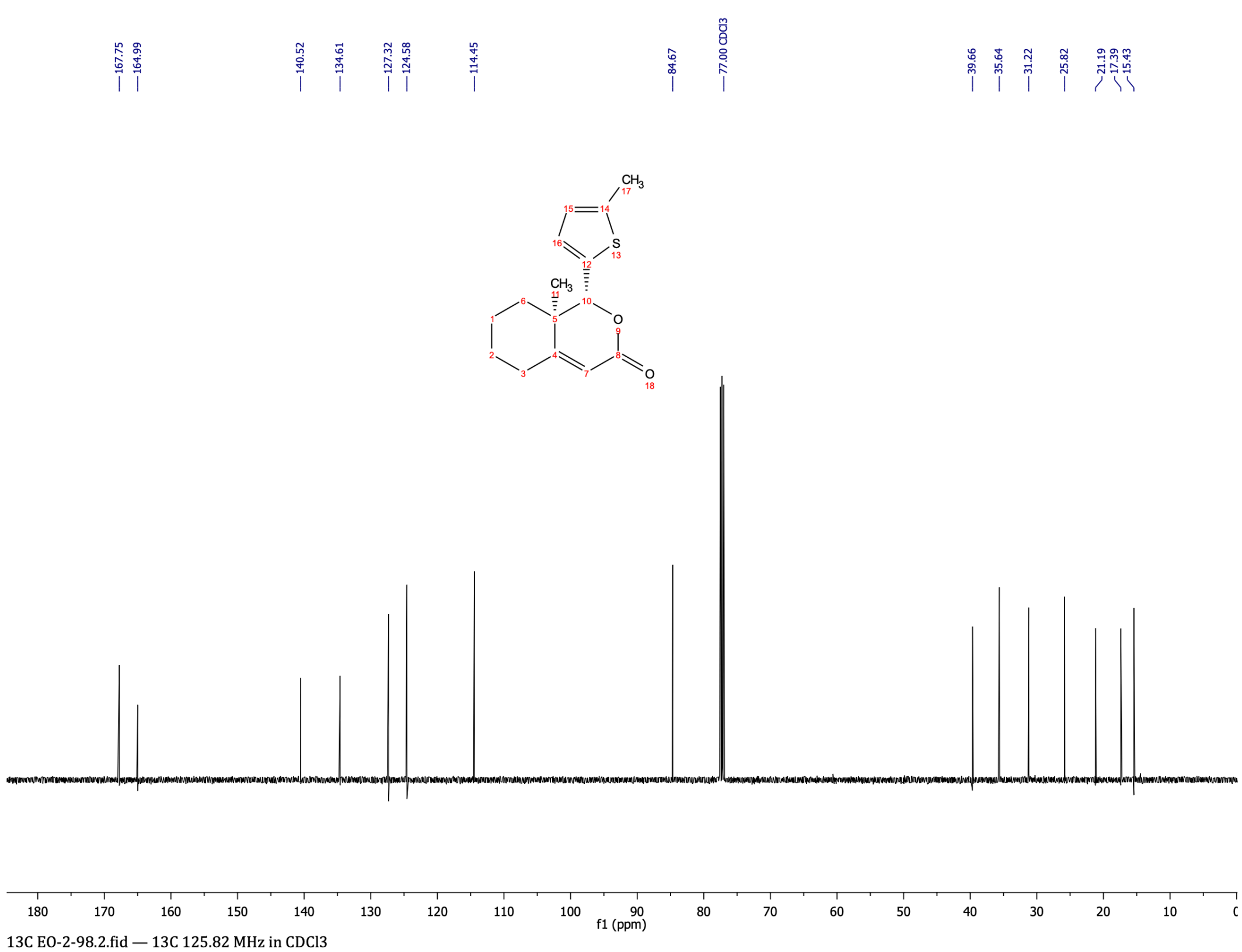

^{1}H NMR (600.21 MHz) and ^{13}C{^{1}H} NMR (150.94 MHz) for Compound **16h** in CDCl3:

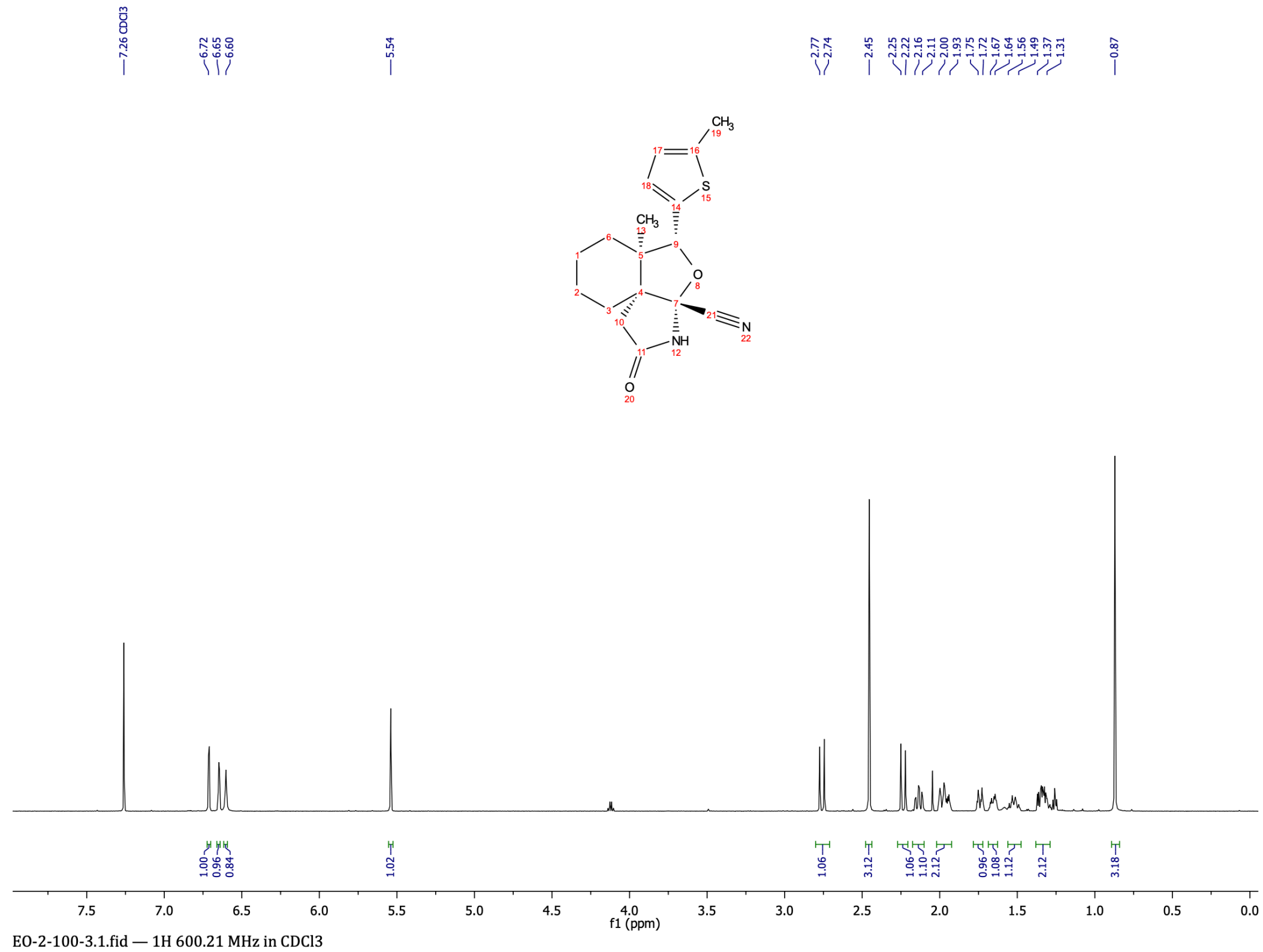

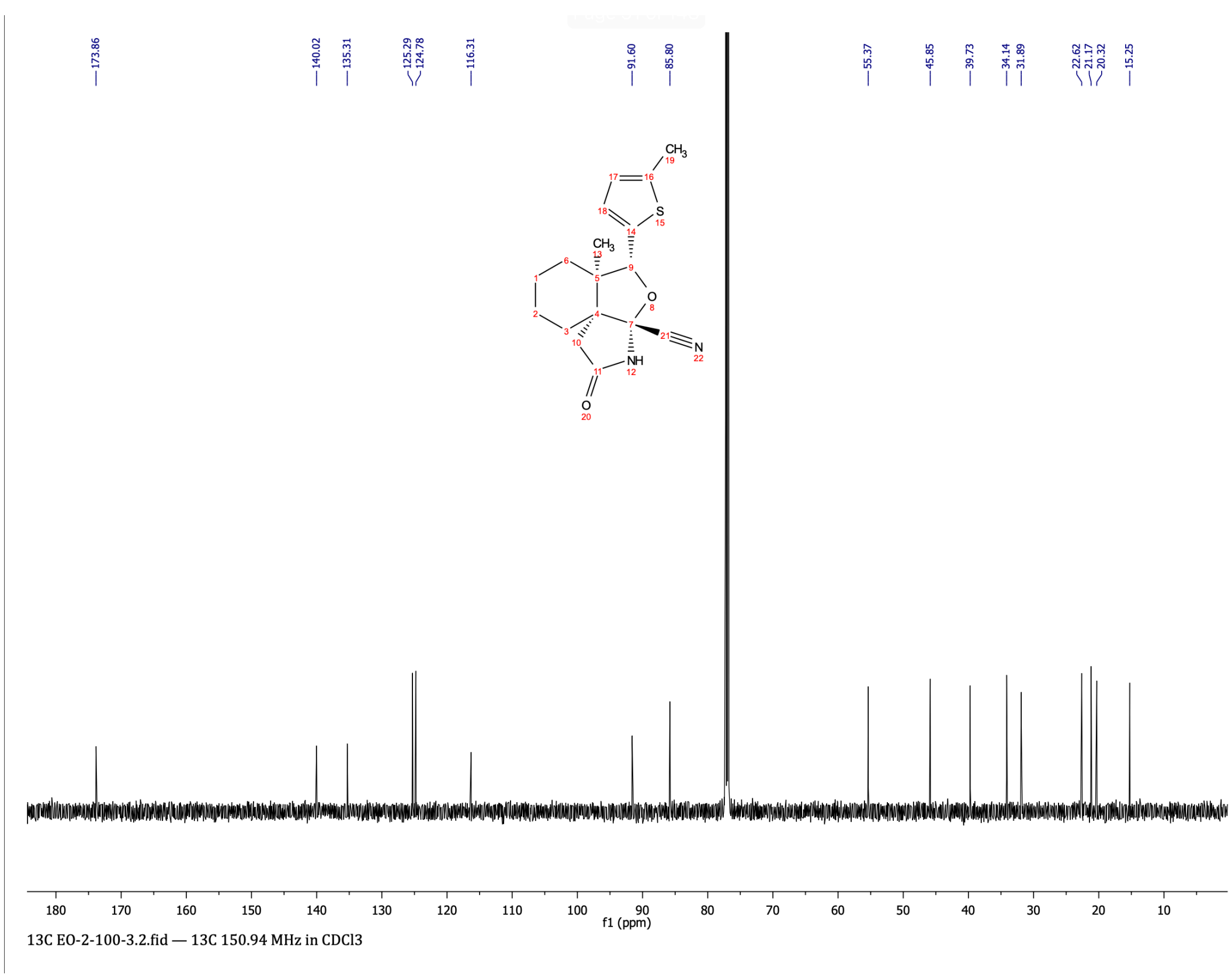

^{1}H NMR (500.30 MHz) and ^{13}C{^{1}H} NMR (125.82 MHz) for Compound **17h** in CDCl3:

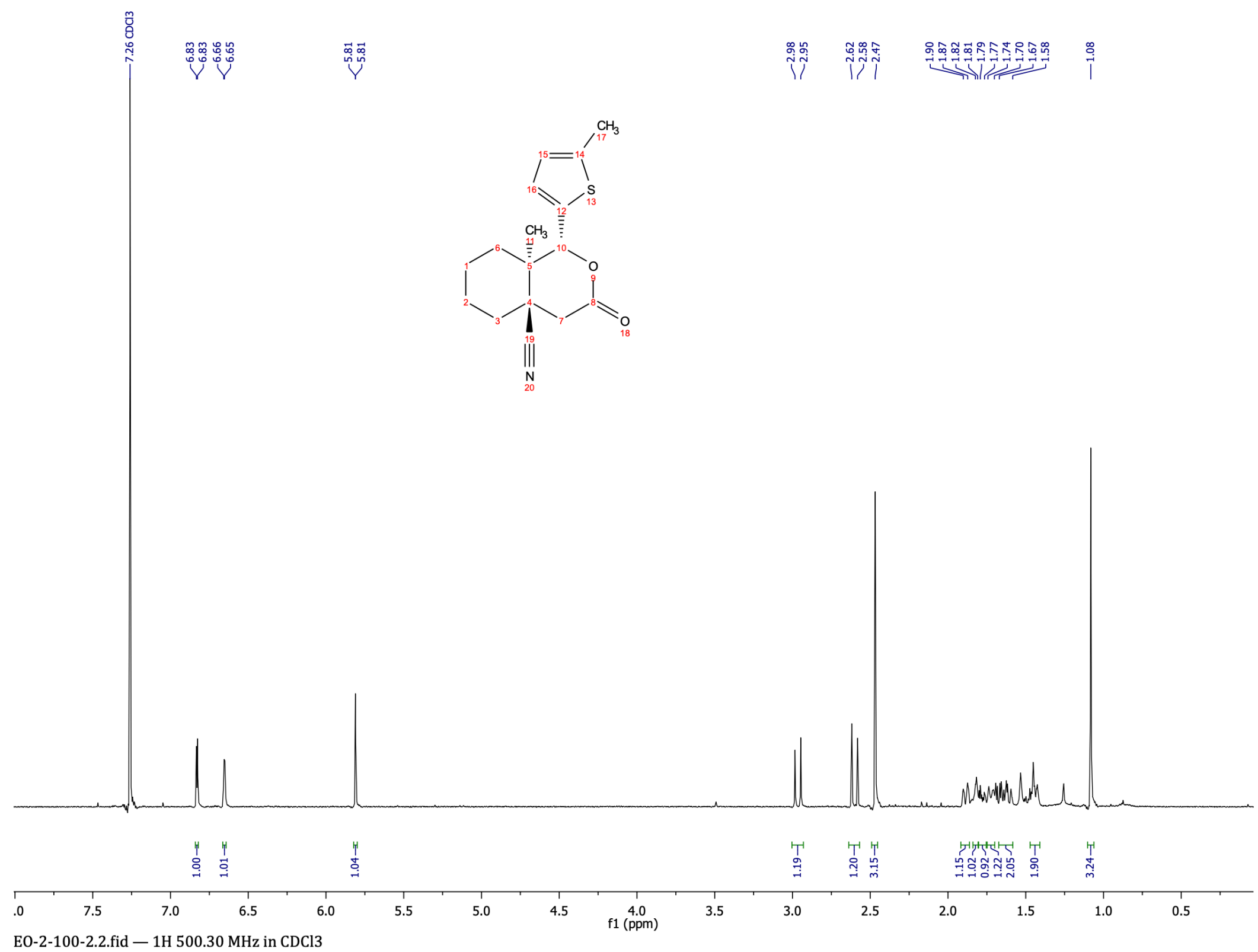

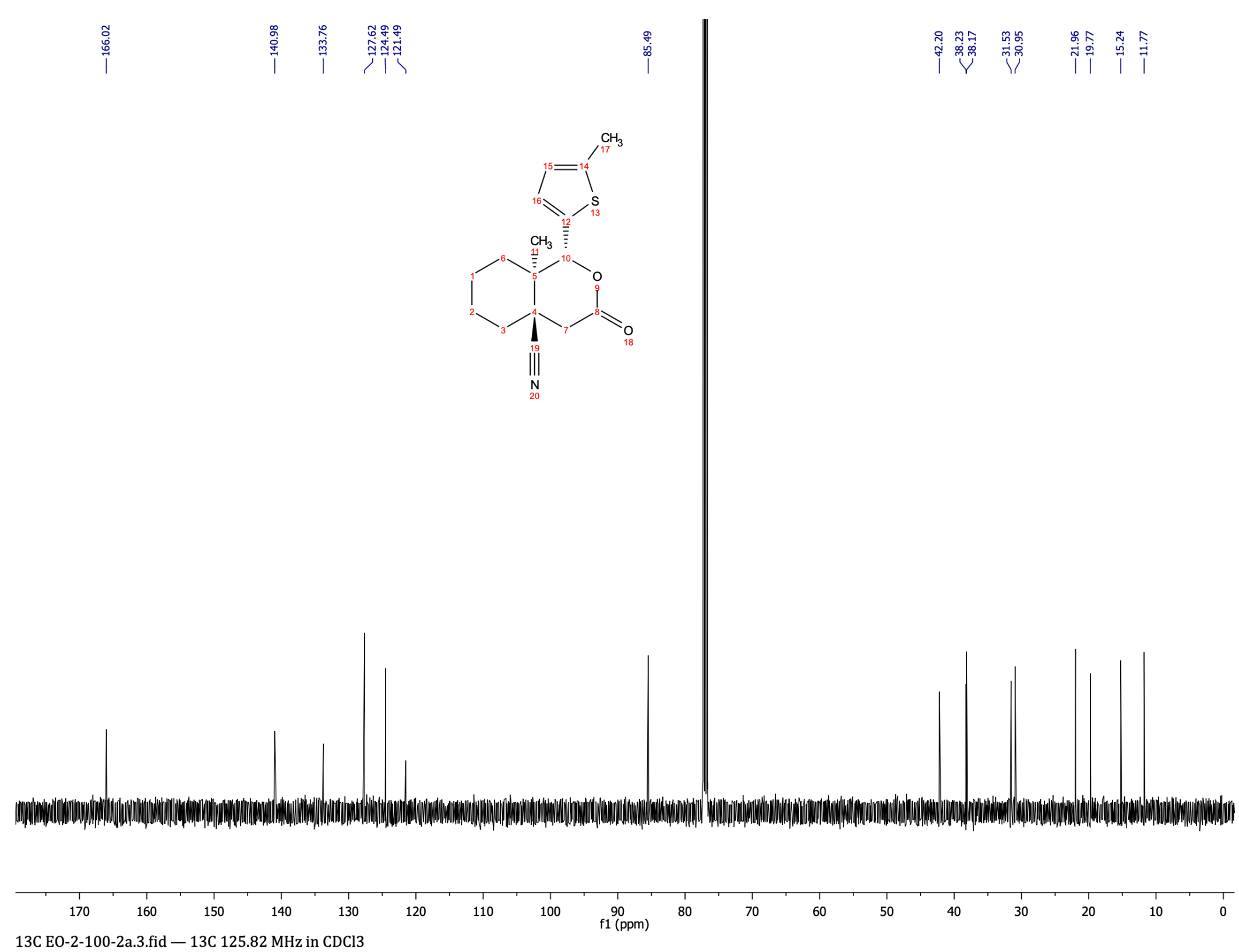

^{1}H NMR (500.30 MHz) and ^{13}C{^{1}H} NMR (125.82 MHz) for Compound **18a** in CDCl3:

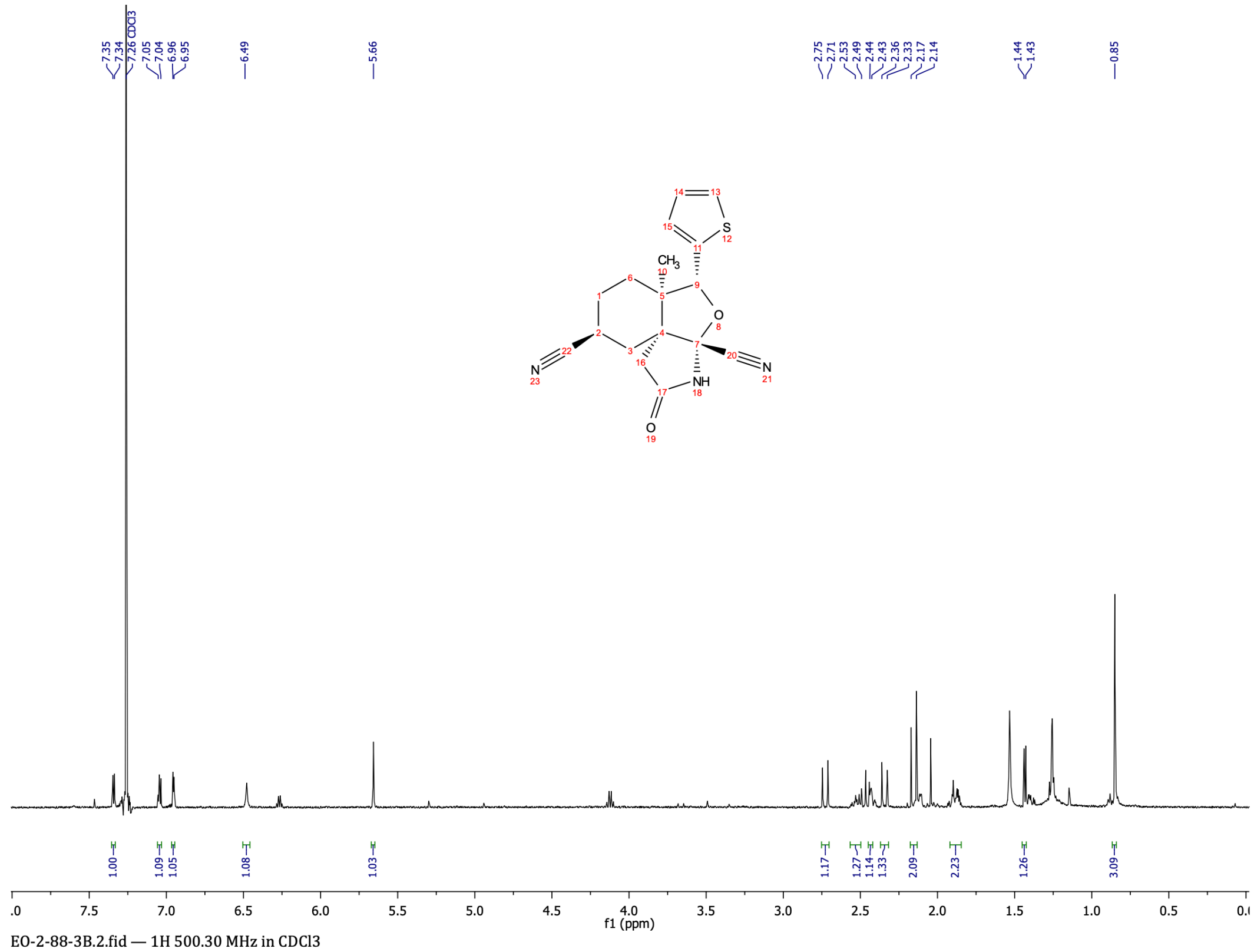

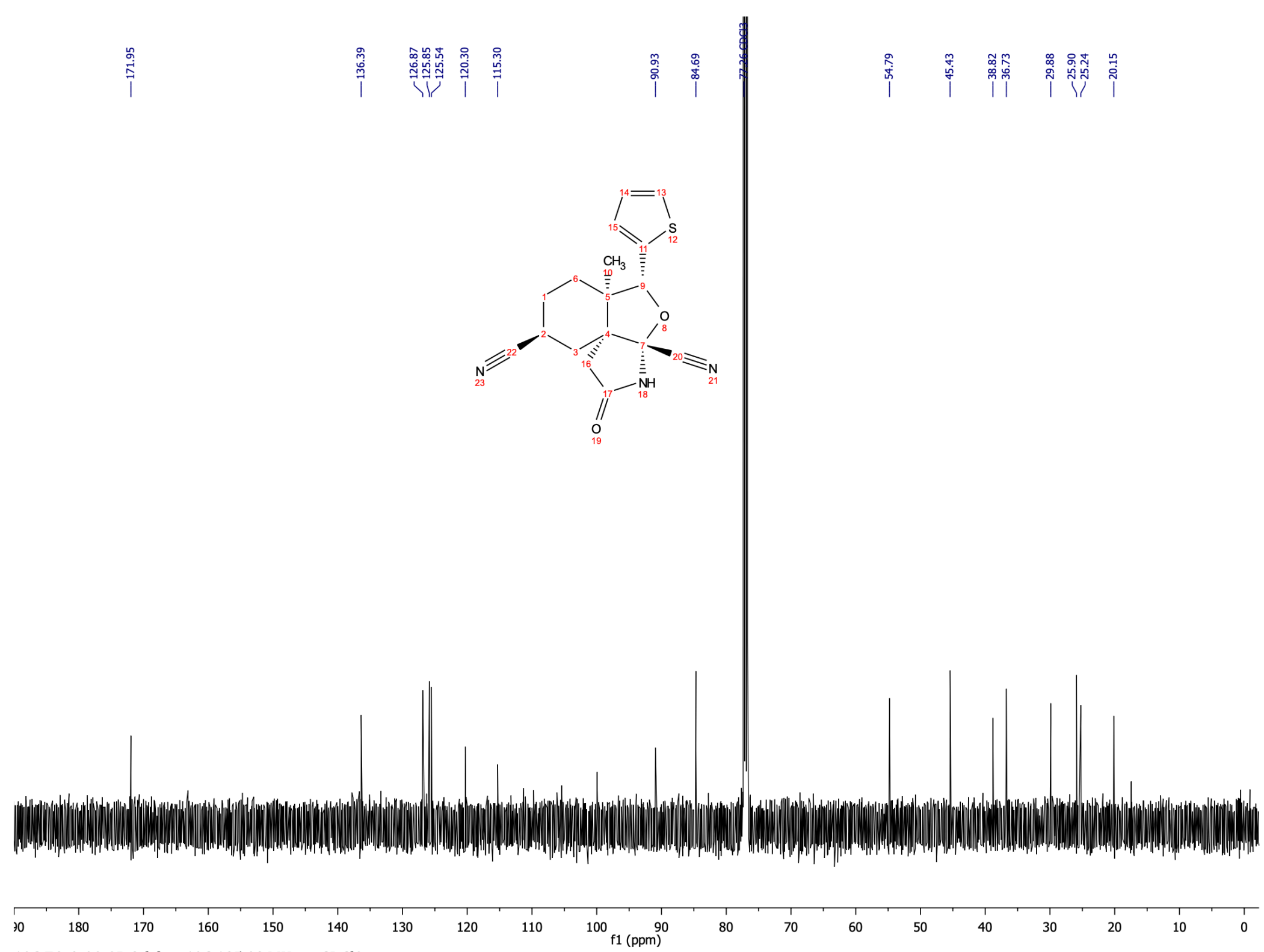

References

1. Bruker (2021). APEX4, SAINT and SADABS. Bruker AXS Inc., Madison, Wisconsin, USA.

2. Sheldrick, G. M. Crystal structure refinement with SHELXL. *Acta Crystallogr*. **2005**, *A71*, 3-8.

3. Sheldrick, G. M. Crystal structure refinement with SHELXL. *Acta Crystallogr*. **2005**, *C71*, 3-8.

4. Dolomanov, O. V.; Bourhis, . L. J.; Gildea, R. J.; Howard, J. A. K.; Puschmann. H. OLEX2: a complete structure solution, refinement and analysis program**.** *J. Appl. Cryst*. **2009**, *42*, 339-341.

5. Marques, F. A.; Lenz, C. A.; Simonelli, F.; Sales Maia, B. H. L. N.; Vellasco, A. P.; Eberlin, M. N. J. Structure Confirmation of a Bioactive Lactone Isolated from Otoba parvifolia through the Synthesis of a Model Compound**.** *J. Nat. Prod.* **2004**, *67***,** 1939–1941.

6. Fernández M. A., De la Fuente Blanco J.A. Synthesis of limonoid model insect antifeedants through stereoselective aldol addition reactions. *J. Org. Chem.* **1991,** *56*, 7084–7092. https://doi.org/10.1021/jo00025a025

7. Vellekoop, A. S; Smith, R.A.J. Steric and complexation effects of 1,4-addition reaction of lithium dimethylcuprate with rigid α,β-unsaturated ketones. *Tetrahedron*, **1998**, *54*, 11971-11994.
